# Dayside Transient Phenomena and Their Impact on the Magnetosphere and Ionosphere

**DOI:** 10.1007/s11214-021-00865-0

**Published:** 2022-06-28

**Authors:** Hui Zhang, Qiugang Zong, Hyunju Connor, Peter Delamere, Gábor Facskó, Desheng Han, Hiroshi Hasegawa, Esa Kallio, Árpád Kis, Guan Le, Bertrand Lembège, Yu Lin, Terry Liu, Kjellmar Oksavik, Nojan Omidi, Antonius Otto, Jie Ren, Quanqi Shi, David Sibeck, Shutao Yao

**Affiliations:** 1 Physics Department & Geophysical Institute, University of Alaska Fairbanks, 2156 Koyukuk Drive, Fairbanks, AK 99775 USA; 2 Institute of Space Physics and Applied Technology, Peking University, Beijing, 100871 China; 3 Polar Research Institute of China, Shanghai, 200136 China; 4 Department of Informatics, Milton Friedman University, 1039 Budapest, Hungary; 5 Wigner Research Centre for Physics, Konkoly-Thege Miklós út 29-33, 1121 Budapest, Hungary; 6 Tongji University, Shanghai, China; 7 Institute of Space and Astronautical Science, JAXA, Sagamihara, Japan; 8 Aalto University, Espoo, Finland; 9 Institute of Earth Physics and Space Science (ELKH EPSS), Sopron, Hungary; 10 NASA Goddard Space Flight Center, Greenbelt, MD 20771 USA; 11 LATMOS (Laboratoire Atmosphères, Milieux, Observations Spatiales), IPSL/CNRS/UVSQ, 11 Bd d’Alembert, Guyancourt, 78280 France; 12 Auburn University, Auburn, USA; 13 Department of Earth, Planetary, and Space Sciences, University of California, Los Angeles, Los Angeles, USA; 14 Birkeland Centre for Space Science, Department of Physics and Technology, University of Bergen, Bergen, Norway; 15 Arctic Geophysics, The University Centre in Svalbard, Longyearbyen, Norway; 16 Solana Scientic Inc., Solana Beach, CA USA; 17 Shandong University, Weihai, China

**Keywords:** Bow shock, Transient foreshock phenomena, Mirror mode, Surface waves, Flux transfer events, Ultra-low frequency waves

## Abstract

Dayside transients, such as hot flow anomalies, foreshock bubbles, magnetosheath jets, flux transfer events, and surface waves, are frequently observed upstream from the bow shock, in the magnetosheath, and at the magnetopause. They play a significant role in the solar wind-magnetosphere-ionosphere coupling. Foreshock transient phenomena, associated with variations in the solar wind dynamic pressure, deform the magnetopause, and in turn generates field-aligned currents (FACs) connected to the auroral ionosphere. Solar wind dynamic pressure variations and transient phenomena at the dayside magnetopause drive magnetospheric ultra low frequency (ULF) waves, which can play an important role in the dynamics of Earth’s radiation belts. These transient phenomena and their geoeffects have been investigated using coordinated in-situ spacecraft observations, spacecraft-borne imagers, ground-based observations, and numerical simulations. Cluster, THEMIS, Geotail, and MMS multi-mission observations allow us to track the motion and time evolution of transient phenomena at different spatial and temporal scales in detail, whereas ground-based experiments can observe the ionospheric projections of transient magnetopause phenomena such as waves on the magnetopause driven by hot flow anomalies or flux transfer events produced by bursty reconnection across their full longitudinal and latitudinal extent. Magnetohydrodynamics (MHD), hybrid, and particle-in-cell (PIC) simulations are powerful tools to simulate the dayside transient phenomena. This paper provides a comprehensive review of the present understanding of dayside transient phenomena at Earth and other planets, their geoeffects, and outstanding questions.

## Introduction

Upstream from Earth’s magnetosphere, many types of transient structures have been frequently observed in or near the foreshock (such as hot flow anomalies (HFAs), foreshock cavities, and foreshock bubbles (FBs)), in the magnetosheath (such as magnetosheath jets), and at the magnetopause (such as flux transfer events and surface waves). They play a significant role in the solar wind-magnetosphere coupling, e.g., by transporting mass, energy, and momentum from the solar wind into the magnetosphere, thereby impacting the whole magnetosphere-ionosphere system.

In the foreshock region, transient structures including HFAs, spontaneous hot flow anomalies (SHFAs), FBs, foreshock cavities, and foreshock cavitons exhibit a common characteristic: low density core regions. In the core regions of HFAs, SHFAs, and FBs, the plasma bulk velocity is significantly deflected. As a result, the dynamic pressure in the core regions of foreshock transients is very low compared to the surrounding solar wind. Since the bow shock location is controlled by the solar wind Mach number and dynamic pressure, when these foreshock transients encounter the bow shock, the local bow shock moves outward resulting in significant perturbations that can propagate into the magnetosheath and disturb the magnetopause (e.g., Sibeck et al. 1999; Archer et al. 2015). Magnetopause perturbations launch field-aligned currents into the magnetosphere that drive traveling convection vortices and plasma flow in the high-latitude ionosphere. Foreshock transients can also transmit compressional waves into the magnetosphere that can excite resonant ultra low frequency (ULF) waves (e.g., Eastwood et al. 2011; Hartinger et al. 2013) and cause particles to scatter into the loss cone and precipitate into the ionosphere, driving transient auroral brightenings (Sibeck et al. 1999; Fillingim et al. 2011).

Jets, localized structures with enhanced dynamic pressure, are often observed in the magnetosheath (e.g., Plaschke et al. 2018a). One explanation is that they are less compressed and thermalized solar wind that penetrates through the rippled bow shock (e.g., Hietala and Plaschke 2013). They are associated with enhanced dynamic pressures that can disturb the magnetopause, resulting in associated geoeffects (e.g., Plaschke et al. 2018a).

At the dayside magnetopause, magnetic reconnection, a fundamental process of energy conversion from electromagnetic fields to charged particles, is the primary process transferring momentum and energy from the solar wind to the magnetosphere. Flux transfer events (FTEs) and their ionospheric signatures play a key role in understanding dayside magnetopause reconnection. On the magnetopause, there are also surface waves (e.g., Hasegawa et al. 2004; Sundberg et al. 2012; Masters et al. 2012). They can be excited by the solar wind pressure variations, transient phenomena near the bow shock, or the Kelvin-Helmholtz instability (KHI) on the magnetopause.

Significant progress has been made on dayside transient phenomena and their impact on the magnetosphere and ionosphere in the past 20 years. This paper provides a comprehensive review of the present understanding of these phenomena. Dayside transient processes occurring at other planets are also discussed. In the following, we start with transient processes in the foreshock, bow shock, and magnetosheath (Sect. 2), then followed by transient dayside magnetopause processes and transport (Sect. 3) and geoeffects of dayside transients (Sect. 4). We discuss some specific outstanding questions at the end of each section. Finally, we summarize the main conclusions of the paper and list some outstanding questions (Sect. 5).

## Transient Processes in the Foreshock, Bow Shock, and Magnetosheath

### Introduction to Earth’s Ion Foreshock

**Fig. 1:**
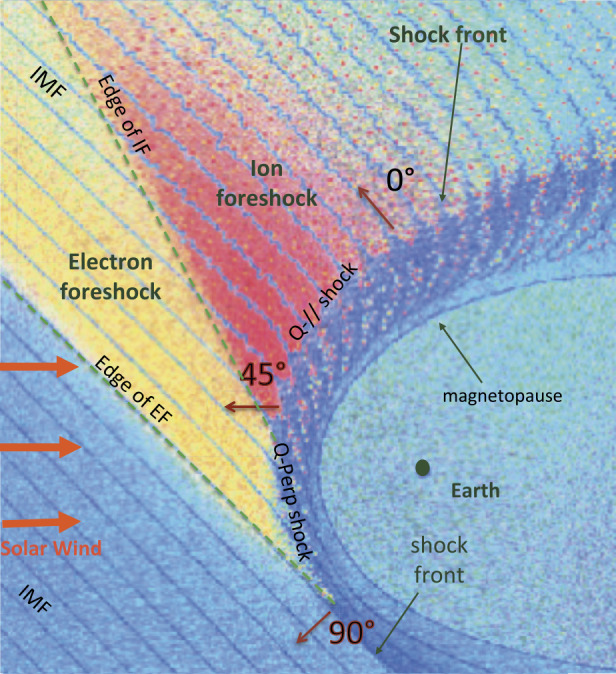
Sketch of the curved terrestrial shock and associated foreshocks; $\Theta _{Bn} = (\mathbf {n}, \mathbf {B}_{0})$ where $\mathbf {n}$ is the local normal to the front. Green dashed lines define the edges of electron and ion foreshock (EF and IF) respectively. (From Tsurutani and Rodriguez 1981)

The terrestrial magnetosphere plays the role of a magnetic obstacle with respect to the continuous and super-fast-magnetosonic flow of the solar wind. A permanent bow shock results, brakes/deviates the flow and allows a transition from the super-fast-magnetosonic to sub-fast-magnetosonic regime. Due to its curved shape, the features of the shock front drastically change according to the local angle ($\Theta _{Bn}$) between the shock normal and the average upstream Interplanetary Magnetic Field (IMF), and are classified into two categories: $90^{\circ } > \Theta _{Bn} > 45^{\circ }$ (so called quasi perpendicular i.e. “Q_⊥_” shock) and $45^{\circ } > \Theta _{Bn} > 0^{\circ }$ (so called quasi parallel i.e. “Q$_{\parallel }$” shock). For “Q_⊥_” shock, when $\Theta _{Bn}$ is large enough ($> 65^{\circ }$, typically), the region upstream from the bow shock is very quiet (solar wind), and the shock transition itself is well defined and presents a sharp and narrow transition between upstream and downstream plasma states. In contrast, the “Q$_{\parallel }$” shock transition is much less clearly defined, and is characterised by an extended and less quiet transition region that extends to distances far upstream from the shock front. It is important to mention that since the Earth’s bow shock is curved, both bow shock types can be found at the same time, adjacent to each other which can have an important implication for ion acceleration mechanism (Lembege et al. 2004; Otsuka et al. 2018; Kis et al. 2018). The foreshock region is often mentioned as a “turbulent area” in the literature. This label is mainly used when comparing it—by contrast—with the (“quiet”) region upstream from Q_⊥_ shocks (Fig. 1). However, thanks to a large number of complementary studies, an improved understanding of the whole foreshock has progressively allowed to identify some distinct wave modes and enhanced power at particular frequencies which emphasizes the point that the foreshock is much more than just a “turbulent” area. In the present context, we will rather use the label ‘nonstationary’ to refer the foreshock regions where important wave activities take place.

Moreover, solar wind particles (electrons and ions) interact quite differently with the shock front and according to which Q_⊥_ or Q$_{\parallel }$ shock region is concerned. Typically, for each species, a large percentage of particles succeed to be directly transmitted, but a certain percentage are reflected by the front and reinjected upstream into the incoming solar wind. More precisely, for $\Theta _{Bn} = 90^{\circ }$, the macroscopic electric field (due to the difference in penetration depth of solar wind ions and electrons) in the front has the appropriate sign to leave electrons passing through the shock front (these are directly transmitted). By contrast, a certain percentage of ions are reflected by the front, suffer a large gyromotion under the combined effect of local macroscopic electric and magnetic fields, gain enough energy during this large gyration and succeed to pass through the front and penetrate downstream; these cannot escape far upstream. Then, the downstream region is composed of both directly transmitted ions (which do not suffer any reflection) and these energetic ions (after being once reflected). The relative percentage of reflected ions strongly depends on the Mach regime of the solar wind and has a strong impact on the microstructures of the shock front itself. Typically, the shock is called supercritical for $M_{\mathrm{{A}}} > 2.5$–3 (with a strong percentage of reflected ions) and is characterized by a foot (upstream of the ramp) and an overshoot (just behind the ramp), both structures being associated with the singly-reflected ions; the percentage does not exceed 20–25% for very high $M_{\mathrm{{A}}}$. In contrast, the shock is called subcritical for $M_{\mathrm{{A}}} < 2$–3 (for a weak percentage of reflected ions, typically a few percents) and presents a laminar profile (i.e. without the presence of a foot or overshoot). For $\Theta _{Bn} = 90^{\circ }$, no particles are reinjected back into the solar wind. However, the situation changes drastically as $\Theta _{Bn}$ deviates from 90°, since the impact of the magnetic field is reduced in terms of controlling the particles’ gyromotion. For each species, an important percentage of particles still succeeds to be directly transmitted but the gyromotion of reflected particles is distorted and some succeed to escape far upstream along the magnetic field lines, depending on the angular deviation of the shock normal as detailed below. In short, the shock front appears as an efficient energy converter (via intricate wave-particles interactions), through which the bulk solar wind energy is transformed into thermal energy, but also as a source of energetic particles backstreaming far upstream. These backstreaming particles are at the origin of the so-called foreshock region, and interact with incoming solar wind to generate various types of microinstabilities which lead to an important upstream wave activity. The features of this wave activity strongly vary according to its origin i.e. in Q_⊥_ or Q$_{ \parallel }$ shocks region, and to the percentage of backstreaming particles. Basically, one identifies two foreshock regions: (i) the “electron foreshock” mainly defined by backstreaming electron beams where the upstream edge is tangent to the curved shock front at $\Theta _{Bn} = 90^{\circ }$ (see Fig. 1). A small deviation from $90^{\circ }$ allows electrons to be easily reflected at the front and escape far upstream, while ions suffer only one or several gyrations forcing these to come back to the front. A much larger deviation from $90^{\circ }$ is necessary for ions to be reflected and escape upstream. (ii) The “ion foreshock” is located deep inside the electron foreshock (see Fig. 1), where its upstream edge starts typically around $\Theta _{Bn} = 62$–$67^{\circ }$ in the examples reviewed by Kucharek et al. (2008) i.e in the Q_⊥_ domain, and is characterised by the presence of backstreaming ion beams (Gosling et al. 1978; Paschmann et al. 1981). In this region, both electron and ion foreshocks coexist. Moreover, experimental observations have evidenced different types of local ion distributions upstream of the front: (a) the so-called “field aligned beam” (FAB) ions (Paschmann et al. 1980, 1981; Thomsen et al. 1983, 1985; Schwartz and Burgess 1984; Oka et al. 2005; Mazelle et al. 2005), (b) the so called “Gyro-Phase Bunched” (GPB) ions (Gurgiolo et al. 1983; Thomsen et al. 1985; Fuselier et al. 1986; Meziane et al. 2001; Mazelle et al. 2003), (c) the “diffuse” ions characterised by a very broad flat energy spectra. The produced beams are considered as the most important source of free energy to generate ULF waves in the ion foreshock (Kucharek and Scholer 1991; Kis et al. 2004; Bonifazi and Moreno 1981a,b), and (d) the “intermediate” ions which are a combination of “FAB” and “diffuse” ions (Oka et al. 2005). FAB ions are characterised by gyrotropic and centered pitch angle distribution, while GPB ions are characterised by non gyrotropic and non-zero centered pitch angle distribution.

Different scenarios have been proposed to account for these various distributions and are summarized in Kucharek et al. (2008) and in Savoini et al. (2013). Kucharek et al. (2004) investigated the origin of FABs and found that they are reflected at the Q_⊥_ side of the bow shock surface. Different mechanisms of FABs formation have been proposed which have been reviewed by Meziane et al. (2005). Paschmann et al. (1981) argued that the initially collimated ion beam can interact with upstream waves and that this interaction leads to the scattering of the beam ions which has been confirmed later by Kis et al. (2007) using data of the Cluster mission. These scattered ions are convected deeper into the foreshock region leading to the appearance of the so-called toroidally gyrating ions, as analyzed by Paschmann et al. (1981) and Thomsen et al. (1985). Gurgiolo et al. (1983) argued that GPB and diffuse ions may be closely related (diffuse ions might directly result from a gyrophase mixing of GPB ions in the upstream region). The diffuse ions present a highly isotropic, doughnut-shaped distribution in velocity space (Paschmann et al. 1981). Trattner et al. (1994) demonstrated, based on a statistical study, that the diffuse ion partial density profile decreases exponentially in the upstream direction along the magnetic field lines. The steepness of the exponential profile depends on the diffuse ion energy. This would suggest that the shock might be the source and the diffuse ions may be indeed shock accelerated particles. The Cluster mission made possible the first direct determination of the diffuse ion spatial profile based on simultaneous multi-spacecraft measurements (Kis et al. 2004). Kronberg et al. (2009) extended this study to higher ion energies. Several numerical simulation/theoretical works have been stimulated in order to account for these different ion populations and for different mechanisms of ion diffusion, which are summarized in Sect. 2.5. Let us specify that “FAB” and “GPB” distributions can be associated with the Q_⊥_ shock, while “diffuse” population more closely associated with the Q$_{\parallel }$ shock, with an intermediate population lying at the transition between Q_⊥_ and Q$_{\parallel }$ shock regions.

Finally, additional boundaries have been identified. Further within the ion foreshock, a third frontier named “ULF waves boundary” (ULFWB) has been evidenced experimentally by Greenstadt and Baum (1986), where ULF waves are generated by the interaction between incoming and backstreaming ion beams (see review of foreshock ULF waves by Wilson (2016)). Throughout this frontier, FAB distributions without ULF waves gradually change to gyrating distributions with the presence of ULF waves. This boundary strongly depends on the IMF cone angle defined between the IMF and the GSE $x$ axis (pointing towards the sun). These observations have been confirmed and analyzed in more detail in later works (Andrés et al. 2015) and supported by theoretical analysis (Skadron et al. 1988). Finally, a fourth boundary named the Foreshock Compressional Boundary (FCB) has been identified consisting of a fast magnetosonic pulse (Sibeck et al. 2008) associated with increased density and magnetic field strength. A deeper investigation of the FCB has been performed by Omidi et al. (2009). All these boundaries are discussed in more detail in Sect. 2.4.4.

### Overview of Transient Phenomena in the Foreshock

This section provides a list and a very short description of foreshock transients based on observations including hot flow anomalies (HFAs), spontaneous hot flow anomalies (SHFAs), foreshock bubbles (FBs), foreshock cavities, foreshock cavitons, foreshock compressional boundaries, density holes, and Short Large-Amplitude Magnetic structures (SLAMs). Table 1 shows a comparison of their characteristics. They are described in detail in Sects. 2.3 and 2.4.

**Table 1:** Comparison of transient phenomena at the bow shock. Durations and scale sizes of the events depend on how they move/convect past a spacecraft in the spacecraft frame. (After Zhang and Zong 2020)

	HFAs	SHFAs	FBs	Foreshock Cavities	Foreshock Cavitons	FCBs	Density Holes	SLAMs
Depletion in n and B	Yes	Yes	Yes	Yes	Yes	Yes on the turbulent side	Yes	No.
Compression in n and B	At edges	At edges	Only on the upstream edge	At edges	At edges	At edges	At edges	Yes
Presence of suprathermal ions	Yes	Yes	Yes	Yes	Yes	Maybe	Yes	Maybe
Significant flow deflection	Yes	Yes	Yes	No	No	No	Sometimes	Sometimes
Significant plasma heating	Yes	Yes	Yes	Modest	No	No	Sometimes	Sometimes
Associated with an IMF discontinuity	Yes	No	Yes	Sometimes	No	No	Yes	No
Duration	10s seconds to minutes	10s seconds to minutes	10s seconds to minutes	10s seconds to minutes	10s seconds to ∼1 minute	10s seconds to minutes	seconds to ∼1 minute	seconds to 10s seconds
Scale size	∼1 to a few $R_{\mathrm{{E}}}$	∼1 to a few $R_{\mathrm{{E}}}$	∼1 to a few $R_{\mathrm{{E}}}$	∼1 to a few $R_{\mathrm{{E}}}$	∼1 to a few $R_{\mathrm{{E}}}$	∼1 to a few $R_{\mathrm{{E}}}$	∼1 to a few $R_{\mathrm{{E}}}$	up to 3000 km
Generation mechanism	Interaction of IMF discontinuities with the bow shock	Interaction of foreshock cavitons with the bow shock	Kinetic interactions between suprathermal, backstreaming ions and incident solar wind plasma with embedded IMF discontinuities that move through and alter the ion foreshock.	Antisunward-moving slabs of magnetic field lines connected to the bow shock that are sandwiched between broader regions of magnetic field lines that remain unconnected to the bow shock.	Nonlinear evolution of ULF waves	Backstreaming ions result in increased pressure within the foreshock region leading to its expansion against the pristine solar wind and the generation of FCBs.	Possibly due to backstreaming particles interacting with the original solar wind	Nonlinear wave steepening

#### Hot Flow Anomalies (HFAs)

**Fig. 2:**
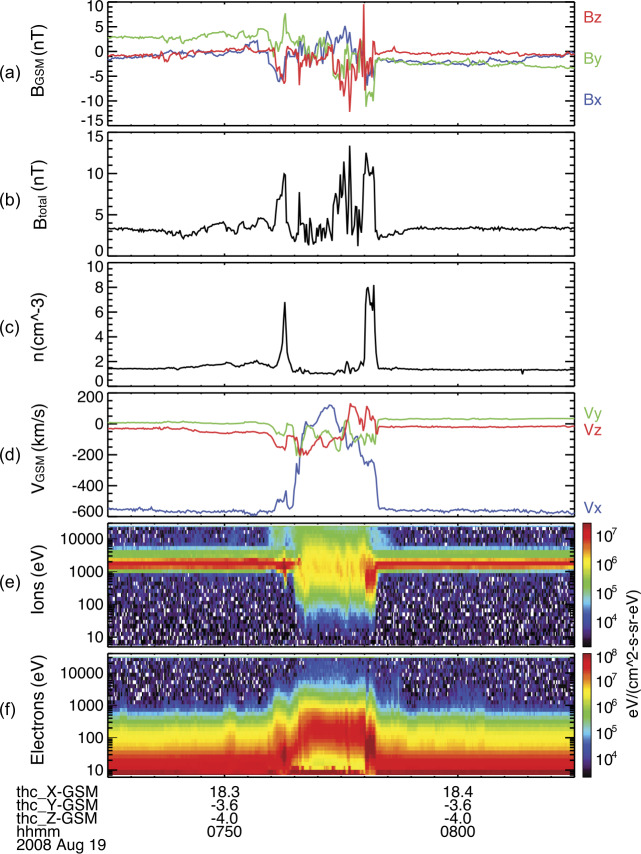
A mature HFA observed by THEMIS C upstream from the bow shock. From top to bottom: (**a**) components of the magnetic field in GSM coordinate system, (**b**) magnetic field magnitude, (**c**) plasma ion density, (**d**) components of plasma flow, (**e**) plasma ion spectrum, (**f**) plasma electron spectrum (from Zhang et al. 2010)

HFAs have been studied for over 30 years. They are characterized by a low field strength and low density core with heated plasma and substantial flow deflection with sizes of several $R_{\mathrm{{E}}}$ (e.g., Schwartz et al. 1985; Schwartz 1995; Schwartz et al. 2018; Thomsen et al. 1986; Lucek et al. 2004b; Facskó et al. 2008; Zhang et al. 2010; Wang et al. 2013b). Figure 2 shows an example of an HFA observed by THEMIS. HFAs are typically driven by a solar wind tangential discontinuity (TD) that intersects the bow shock with solar wind convection electric field pointing inward on at least one side of the TD (e.g., Thomas et al. 1991; Schwartz et al. 2000). Such a TD can locally trap foreshock ions leading to the HFA formation while propagating along the bow shock surface. HFAs may accelerate particles efficiently through Fermi acceleration, i.e., bouncing between the converging HFA boundary and the bow shock (Turner et al. 2018). Lee et al. (2020) provided an alternative explanation for these energetic ions observed upstream from the bow shock and in the magnetosheath. They suggested that the energetic ions have escaped from the outer magnetosphere.

#### Spontaneous Hot Flow Anomalies (SHFAs)

**Fig. 3:**
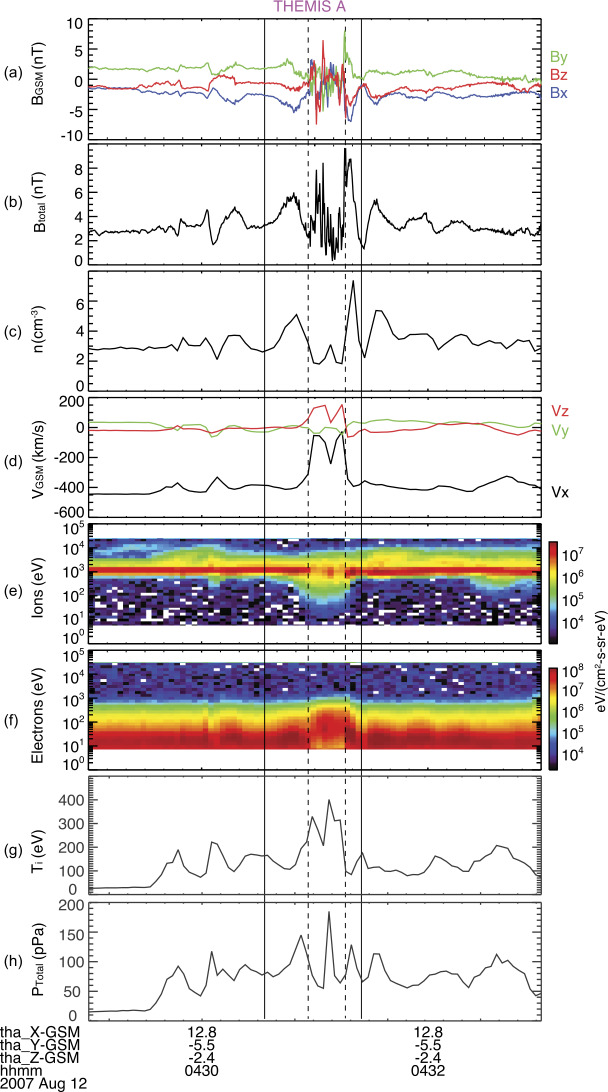
An overview plot of THEMIS A observations of a spontaneous HFA upstream from the bow shock. From top to bottom: (**a**) components of the magnetic field in the GSM coordinate system, (**b**) magnetic field magnitude, (**c**) plasma ion density, (**d**) components of plasma flow in GSM coordinate system, (**e**) plasma ion spectrum, (**f**) plasma electron spectrum with 3 s time resolution (from Zhang et al. 2013)

SHFAs have the same characteristics as HFAs except that they are not associated with any solar wind discontinuities (Zhang et al. 2013). They form intrinsically in the quasi-parallel regime, likely due to the interaction between foreshock cavitons and the bow shock (Omidi et al. 2013b). Figure 3 shows an example of an SHFA observed by THEMIS.

#### Foreshock Bubbles (FBs)

**Fig. 4:**
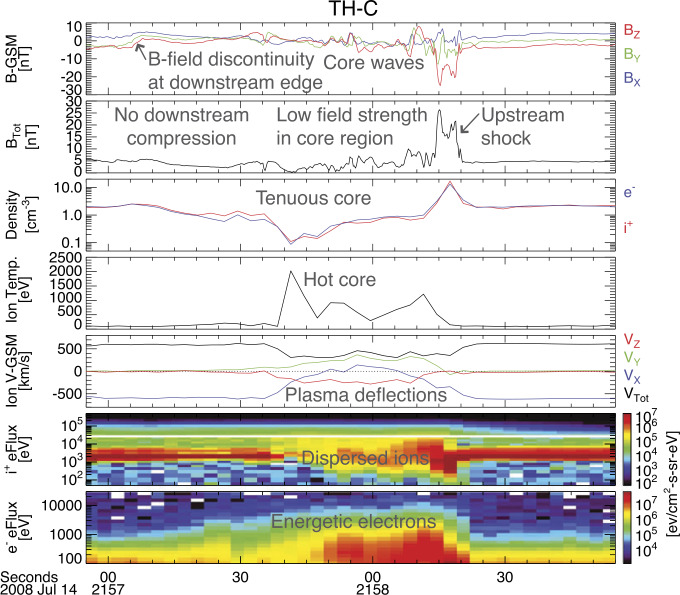
A foreshock bubble observed by THEMIS C upstream from the bow shock. From top to bottom: components of the magnetic field in GSM coordinate system, magnetic field magnitude, plasma ion and electron density, ion temperature, components and magnitude of plasma flow in the GSM coordinate system, plasma ion spectrum, plasma electron spectrum with 3 s time resolution (from Turner et al. 2013)

When backstreaming foreshock ions interact with a solar wind rotational discontinuity (RD) that does not necessarily intersect the bow shock, FBs form upstream of the RD and convecting anti-sunward with it (Omidi et al. 2010, 2020b; Turner et al. 2013). Later observations (Liu et al. 2015, 2016b) and simulations (Wang et al. 2021) found that TDs can also drive FBs. FBs are also characterized by a heated, tenuous core with significant flow deflection (Fig. 4). Different from HFAs and SHFAs, the expansion of FBs is super-fast-magnetosonic and dominantly in the sunward direction. Because of the sunward super-fast-magnetosonic expansion, a shock forms upstream of the core, and the FB size in the expansion direction can reach 5-10 $R_{\mathrm{{E}}}$, larger than typical HFAs and SHFAs. In addition to their significant dynamic pressure perturbations, FBs are also efficient particle accelerators due to the presence of the shock (e.g., shock drift acceleration (Liu et al. 2016a) and Fermi acceleration (Liu et al. 2017d, 2018; Omidi et al. 2021) as the shock converges towards the bow shock).

#### Foreshock Cavities

**Fig. 5:**
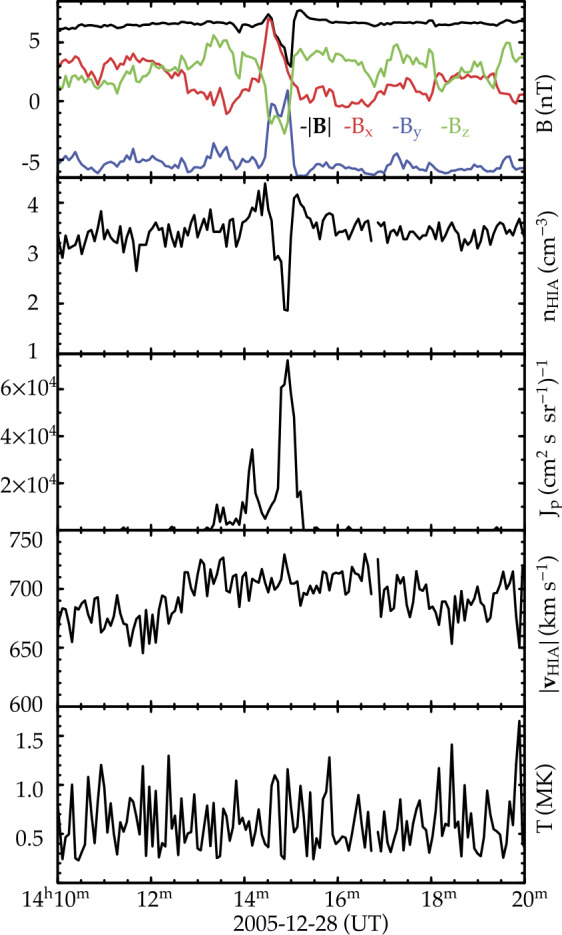
A foreshock cavity observed upstream from the bow shock. From top to bottom: magnitude of the magnetic field and its components in GSE coordinates, density of the thermal ion population, flux of energetic ions (≥27 keV), bulk flow speed, ion temperature (from Billingham et al. 2008)

Foreshock cavities are characterized by low density, low field strength core regions with high density, high field strength compressional boundaries on two sides (e.g., Sibeck et al. 2002, 2004; Schwartz et al. 2006; Billingham et al. 2008). But different from HFAs, the flow deflection inside foreshock cavities is rather weak and plasma heating is not significant (Fig. 5). When slabs of magnetic field lines connected to the bow shock are bounded by broader regions of magnetic field lines that remain unconnected to the bow shock, only the slabs are filled with energized particles reflected from the bow shock. The presence of foreshock particles enhanced the thermal pressure, causing an expansion on two sides. Such an expansion decreases the plasma density and magnetic field strength inside the slabs and increases the density and field strength at two boundaries, i.e., a foreshock cavity forms (Schwartz et al. 2006).

#### Foreshock Cavitons

**Fig. 6:**
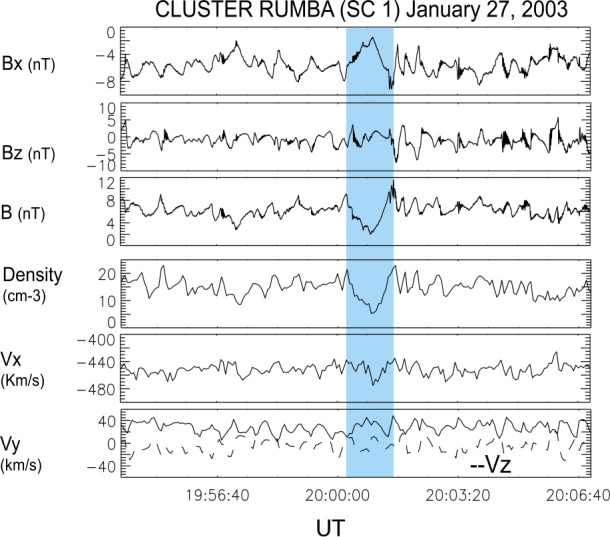
Cluster C1 observations of a foreshock caviton upstream from the bow shock. From top to bottom: components and magnitude of the magnetic field, plasma density, bulk flow velocity (from Blanco-Cano et al. 2009)

Foreshock cavitons are also characterized by a core region with low density and field strength bounded by two boundaries with high density and field strength, without clear heating and flow deflection (Fig. 6). Their sizes are about one $R_{\mathrm{{E}}}$. They form due to the nonlinear evolution of two types of waves: the parallel propagating sinusoidal waves and the highly oblique, linearly polarized, fast magnetosonic waves (Lin 2003; Lin and Wang 2005; Omidi and Sibeck 2007; Blanco-Cano et al. 2009, 2011). Thus, foreshock cavitons are embedded in foreshock ULF waves, whereas foreshock cavities are isolated due to their different formation mechanisms.

#### Foreshock Compressional Boundaries

**Fig. 7:**
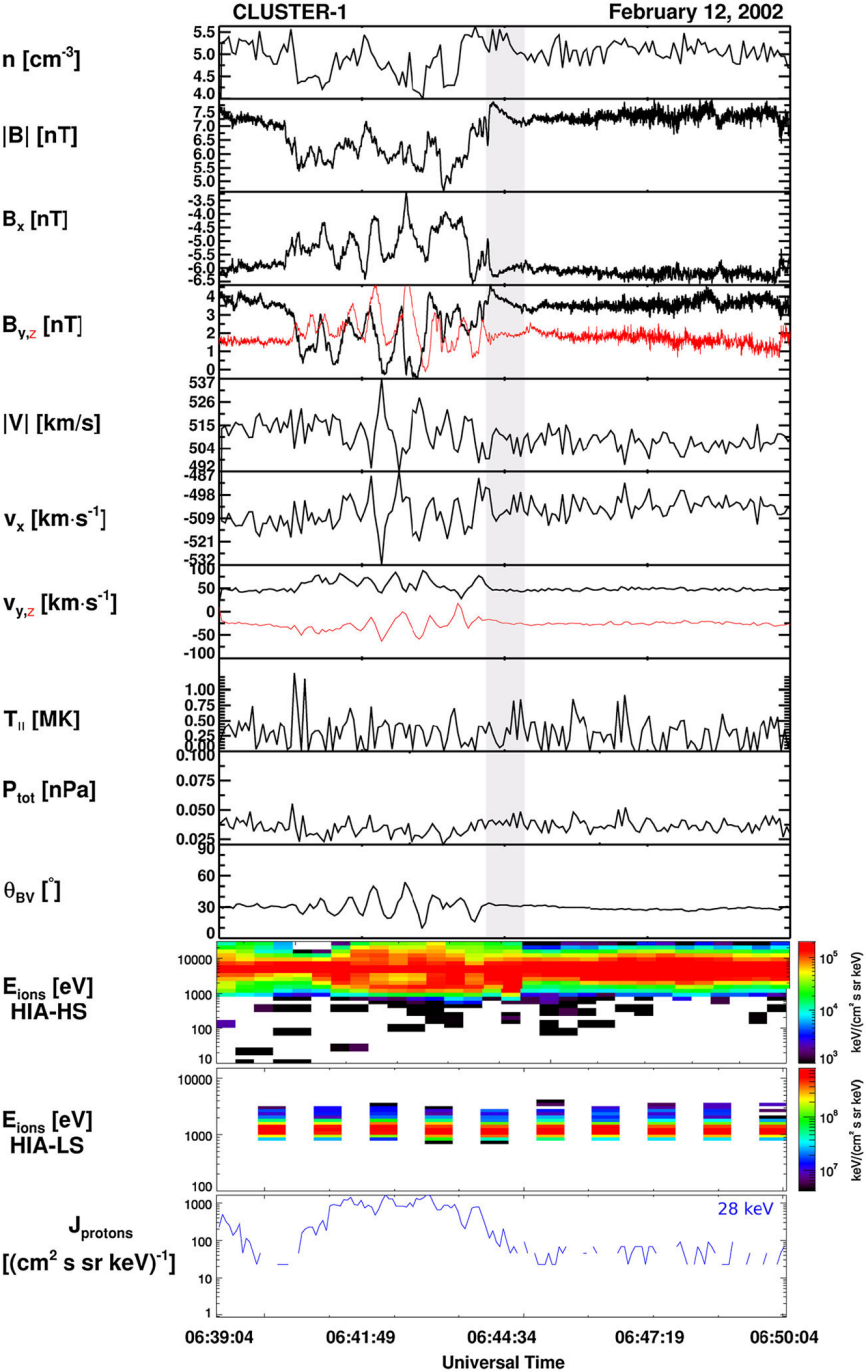
FCB (grey shaded region) observed by Cluster 1 on 12 February 2002. From top to bottom: plasma density, magnitude and components of the magnetic field, magnitude and components of the bulk flow, ion temperature parallel to the magnetic field, thermal plus magnetic pressure, cone angle, Hot Ion Analyzer (HIA)-HS omnidirectional ion energy spectrum, HIA-LS omnidirectional ion energy spectrum, flux of energetic protons (from Rojas-Castillo et al. 2013)

Foreshock compressional boundaries (FCBs) (Sibeck et al. 2008; Omidi et al. 2009) have enhanced density and field strength (Fig. 7). They occur at the boundary between the foreshock and the pristine solar wind. Because of the high thermal pressure due to the presence of foreshock ions, the foreshock region expands into the ambient pristine solar wind, leading to the formation of an FCB. FCBs are sometimes associated with local density and field strength depletion on their foreshock side. FCBs can form under either steady or nonsteady IMF conditions.

#### Short Large-Amplitude Magnetic Structures (SLAMS) and Shocklets

**Fig. 8:**
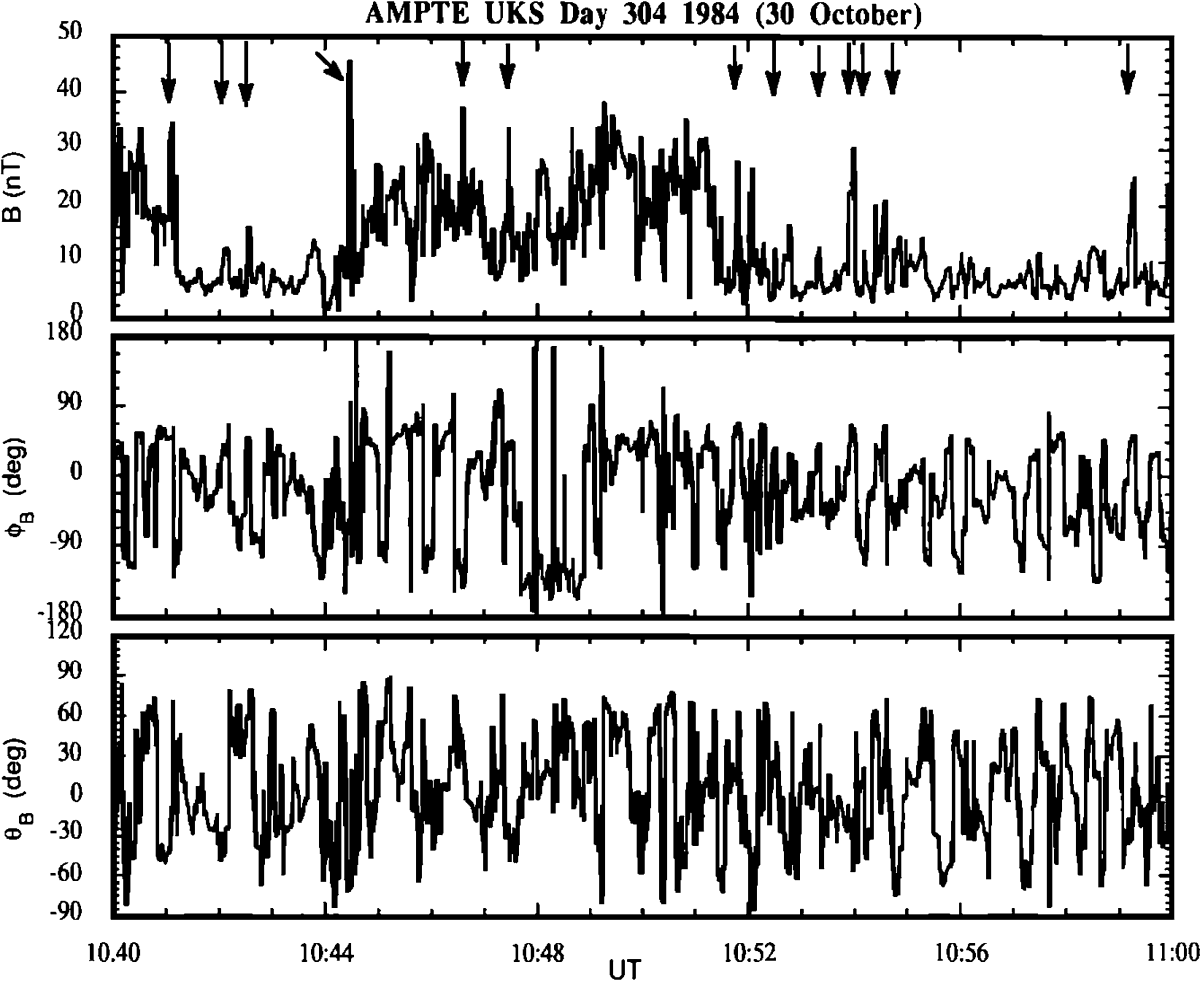
Short Large-Amplitude, Magnetic Structures (SLAMs) observed upstream from the Earth’s bow shock. From top to bottom: the magnitude, azimuth angle, and latitude of the magnetic field (from Schwartz et al. 1992)

SLAMS are magnetic pulsations with amplitudes at least two times the ambient magnetic field strength (e.g., Schwartz and Burgess 1991; Wilson 2016). Two dimensional hybrid simulations showed that SLAMS have typical spatial scales up to a thousand kms and more in the direction parallel to the shock normal and around 3000 km in the direction perpendicular (Dubouloz and Scholer 1995) which are consistent with observations showing scales larger than 1000 km and 3000 km respectively (Greenstadt et al. 1982; Lucek et al. 2004a, 2008) which covers many ion gyroradii (where the thermal ion gyroradius is typically 160 km in the solar wind) and grow rapidly with time scales of ∼seconds (e.g., Lucek et al. 2008). Observational examples of SLAMS are shown in Fig. 8. Shocklets are also magnetic structures (nonlinearly steepened magnetosonic waves), but differ from SLAMS in terms of amplitude, spatial scale, growth rate, and propagation angle (see Table 3). The relationship between SLAMS and shocklets and their relative origin are detailed in Sect. 2.4.5.

**Fig. 9:**
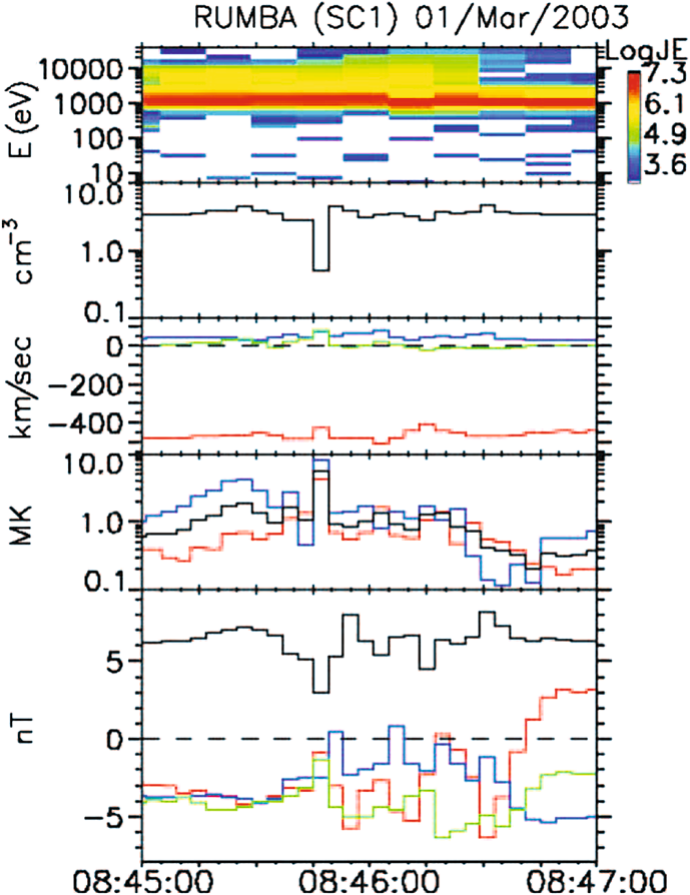
A density hole observed by Cluster upstream from the bow shock. From top to bottom: ion energy spectrum (in eV), ion density (in cm^−3^), components of the plasma flow velocity (in km/s) in GSE coordinates, ion temperature (in MK), the magnitude and components of the magnetic field in GSE coordinates (from Parks et al. 2006)

Density holes are characterized by similarly shaped magnetic holes with enhanced density and field strength at one or both edges (Fig. 9). The definition of density holes overlaps with HFAs, SHFAs, FBs, foreshock cavities, and foreshock cavitons. A recent statistical study (Lu et al. 2022) showed that ∼66% of 411 density holes cannot be categorized by any of these foreshock transient types. Therefore, it is necessary to make density holes a separate category. A better definition of density holes is needed to definitely distinguish them from other foreshock transient types, which requires further studies. The formation could be due to the interaction between backstreaming particles and the original solar wind (Parks et al. 2006).

### Hot Flow Anomalies (HFAs) and Spontaneous Hot Flow Anomalies (SHFAs)

#### HFAs and SHFAs: Universal Phenomena

Numerical simulations (Thomas et al. 1991; Lin 1997; Omidi and Sibeck 2007) and observations (Thomsen et al. 1993) indicate that HFAs form when tangential discontinuities with electric fields pointing towards the discontinuities on at least one side glide slowly along standing shock waves, giving the events sufficient time to form (e.g., Schwartz et al. 2000). Consequently, there is reason to expect them to occur at all of the standing shock waves that occur throughout the heliosphere (Lucek et al. 2004b), namely at each of the planets, in particular the giant planets in the outer heliosphere (Facskó et al. 2008), and at the termination shock.

To the degree that instrument capabilities suffice, there is in fact considerable evidence for this. HFAs are of course well observed and common at Earth in the high cadence plasma and magnetic field observations returned by a host of missions. Here they occur at a rate of several per day (Schwartz et al. 2000), but sometimes more frequently, and have dimensions on the order of 1-2 $R_{\mathrm{{E}}}$ (Facskó et al. 2009). MESSENGER magnetic field and plasma observations provide some evidence for HFA-like magnetic field perturbations associated with suprathermal ions at Mercury (Uritsky et al. 2014). Similarly, Slavin et al. (2009) have presented MESSENGER magnetic field observations of features which resemble HFAs in the foreshock of Venus. Collinson et al. (2012a, 2014, 2017) have reported the results of more extensive surveys of Venus Express observations that provide evidence for the presence of the magnetic field perturbations and heated, deflected plasma expected for HFAs and spontaneous HFAs in the Venusian foreshock. HFA sizes at Venus are large compared to those of the planetary obstacle and events may have an important effect on the solar wind interaction with Venus (Fig. 11). Motional electric fields point inward on at least one side of all events reported at Venus. Omidi and Sibeck (2007) reported the successful simulation of SHFAs in a hybrid code simulation of the solar wind’s interaction with Venus.

Øieroset et al. (2001) reported Mars Global Surveyor magnetic field and electron observations of two hot, diamagnetic cavities that exhibited magnetic field strength and electron density depressions associated with electron heating, in a manner similar to HFAs in the foreshock upstream from Mars. Instrumental limitations prevented identification of flow deflections, if any. The comprehensive instrumentation on MAVEN subsequently permitted Omidi et al. (2017a) to identify all of the features that characterize an SHFA in the foreshock of Mars.

**Fig. 10:**
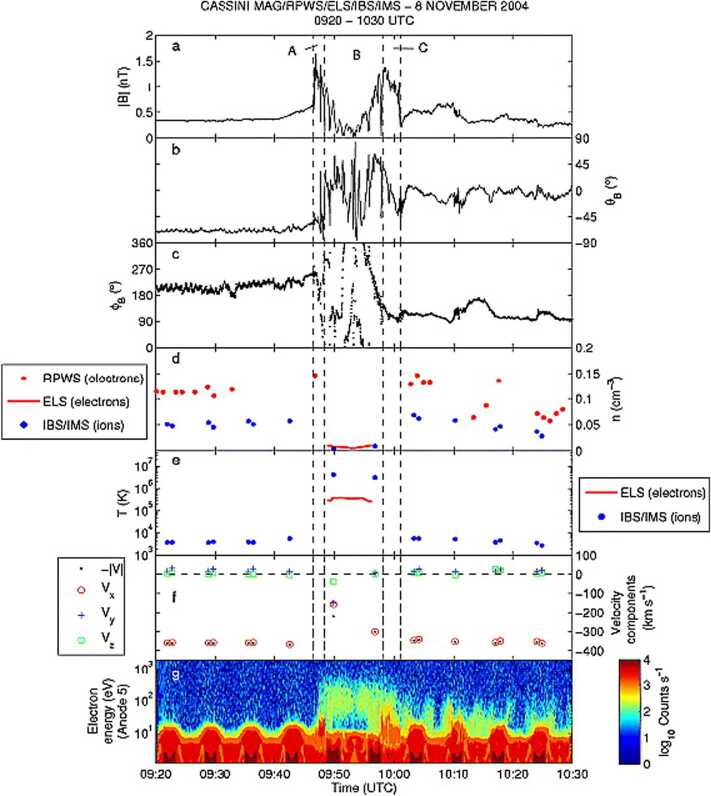
An HFA analogy in the Saturn system. (**a**-**c**) Magnitude and direction of the magnetic field in spherical polar coordinates. (**d**) Electron and ion number densities. (**e**) Election and ion temperatures. (**f**) Components of the flow velocity. (**g**) Time-energy spectrogram of electron count (from Masters et al. 2009b, Fig. 4)

Valek et al. (2017) reported Juno observations of a very large HFA at the intersection of an interplanetary discontinuity with the Jovian bow shock. This event exhibit the density, magnetic field, and flow deflections expected for an HFA. Its dimensions were estimated to be on the order of $2 \times 10^{6}\mbox{ km}$. Masters et al. (2008) reported Cassini electron plasma and magnetic field observations of two HFAs at Saturn. See Fig. 10 for an example. The events occurred at the intersection of IMF discontinuities with the planetary bow shock. By contrast to HFAs observed elsewhere, there were electron density enhancements within the core region of the events. Event dimensions were again large, 2 and 6 Saturnian radii. As shown in Fig. 12, the event dimension at different planets increases with increasing distance from the Sun.

**Fig. 11:**
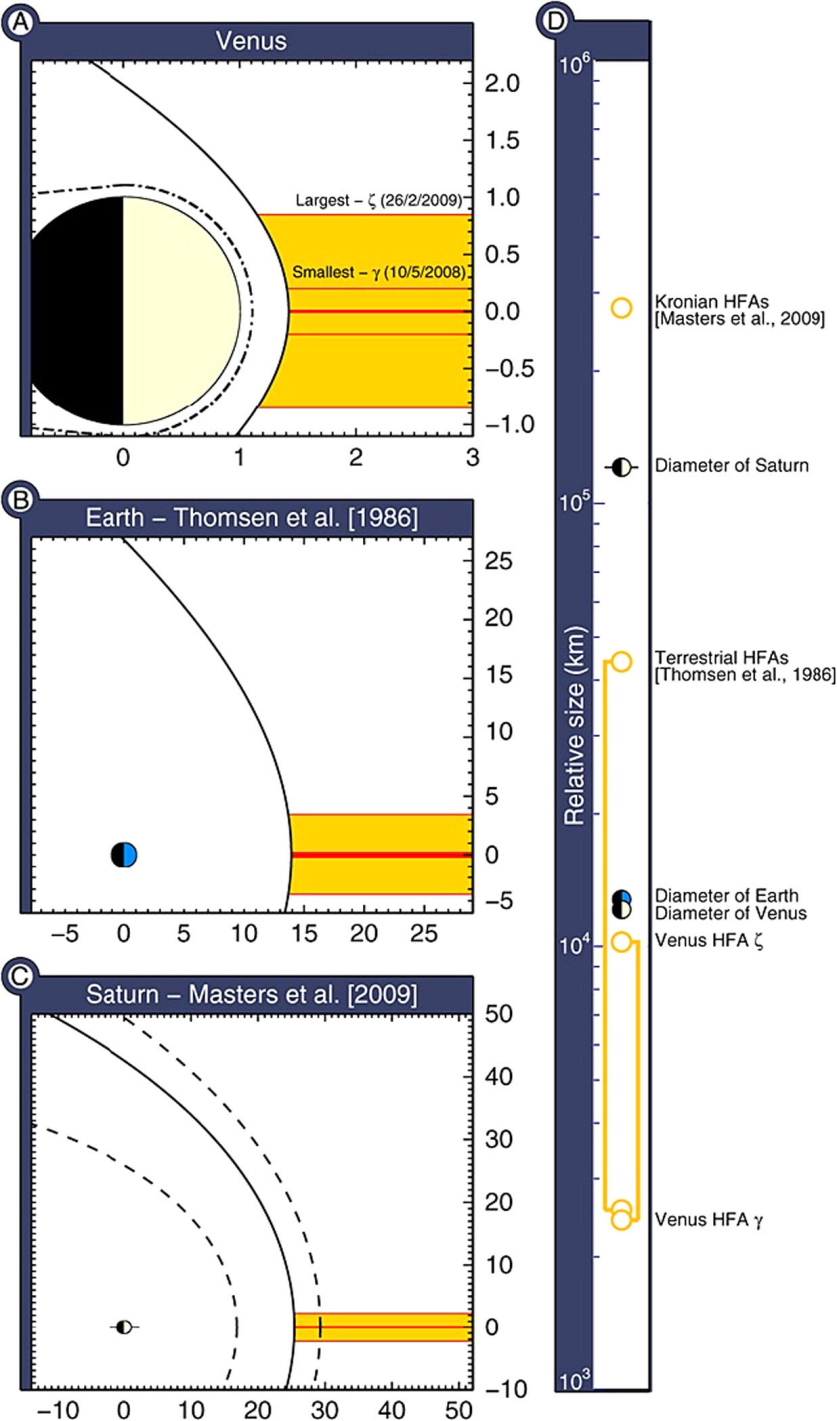
Comparison of HFA sizes relative to their parent magnetosphere in units of planetary radii. Each HFA is artificially moved and rotated to lie along the planet-Sun line. (**a**) Venus (this study); (**b**) Earth according to Thomsen et al. (1986) (**c**) Saturn according to Masters et al. (2009b). (**d**) Scale showing actual diameters (in kilometers) of Venus, Earth, and Saturn, and HFAs at each (from Collinson et al. 2014, Fig. 5)

**Fig. 12:**
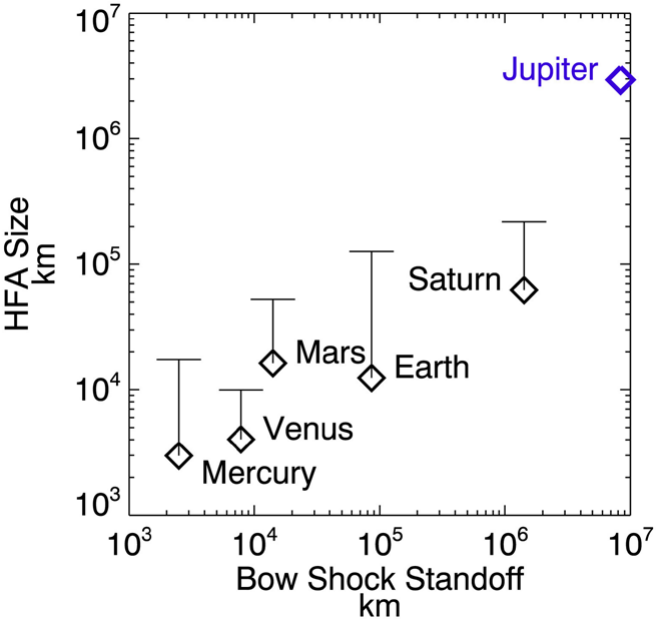
Comparison of typical sizes of HFAs for various planets. Diamonds and bars indicate the typical and largest HFA sizes, respectively. The HFA sizes and planetary bow shock standoff distances are from Uritsky et al. (2014) except for Jupiter. (From Valek et al. 2017, Fig. 4)

Finally, Giacalone and Burgess (2010) presented hybrid code simulations for the interaction of the Heliospheric Current Sheet with the termination shock. They concluded that as the inclination of the current sheet increased relative to the shock normal, the chance of generating a HFA diminished. Since the heliospheric current sheet is highly inclined to the radial direction, they concluded that HFA formation was highly unlikely.

#### Size, Evolution and Propagation Characteristics of HFAs

**Fig. 13:**
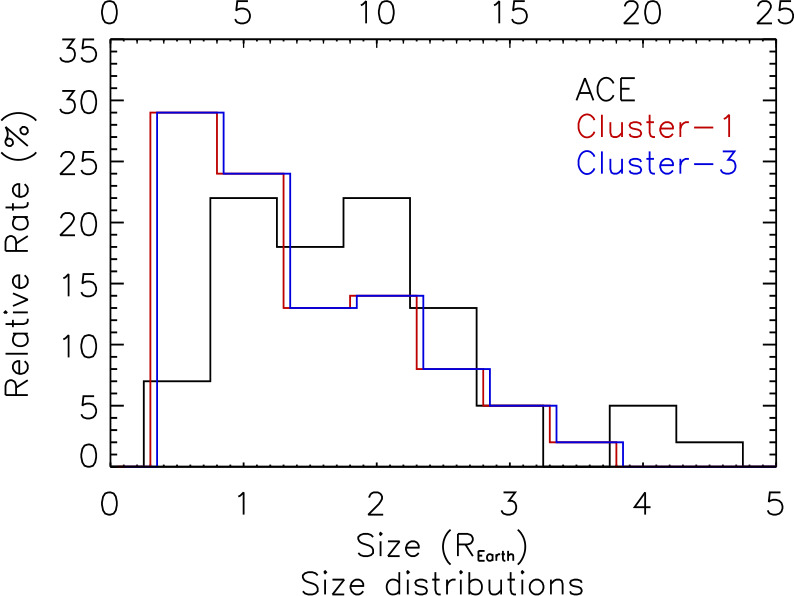
The size distributions of HFAs by assuming that the expansion speed is the Alfvén speed (black line, scale at the bottom) and using the TD transit speed along the bow shock surface from Cluster-1 and -3 CIS HIA measurements (red and blue lines, scale at the top). The average sizes are (1.9±1.0) $R_{\mathrm{{E}}}$, (7.0±4.3) $R_{\mathrm{{E}}}$, and (6.6±4.2) $R_{\mathrm{{E}}}$, respectively. (From Facskó et al. 2009, Fig. 5)

As HFAs are driven by TDs that intersects the bow shock, HFAs convect with the driver TD along the bow shock surface. The size of an HFA can be estimated based on such motion across spacecraft. Schwartz et al. (1985) showed that the thickness of an HFA along the TD normal direction was 2-3 $R_{\mathrm{{E}}}$. Later observations showed similar spatial scales of several $R_{\mathrm{{E}}}$ (e.g., Thomsen et al. 1986; Sibeck et al. 1999). The HFA size in global hybrid simulations performed by Lin (2002) is 1-2 $R_{\mathrm{{E}}}$. Facskó et al. (2009, 2010) determined sizes of HFAs using two different methods based on Cluster observations. In the first method, they assumed that the expansion speed of each boundary is the Alfvén speed and used the expansion speed and the event duration to calculate the size. In the second method, the HFA size along the bow shock surface is calculated using the HFA time interval multiplied by the transit speed of the driver TD (Eq. (1)). The determined sizes were 2-3 $R_{\mathrm{{E}}}$ using the first method, and the second method gave up to 1 $R_{\mathrm{{E}}}$ larger sizes. The differences might be due to the high sensitivity of the methods to the measurement accuracy (Fig. 13). Using MMS observations, Schwartz et al. (2018) calculated the size of an HFA along the bow shock surface as 2.3 $R_{\mathrm{{E}}}$. MMS observed HFAs, SHFAs, or FBs that just started to form with spatial scales of around one foreshock ion gyroradius (1000 to 2000 km) along the solar wind direction (Liu et al. 2020a). ARTEMIS in the midtail foreshock at $X=-40$ to −50 $R_{\mathrm{{E}}}$ observed an HFA with a spatial scale of 1.7 $R_{\mathrm{{E}}}$ along the bow shock surface (Liu et al. 2020e).

To obtain accurate expansion speeds of HFAs, Xiao et al. (2015) used the timing method based on four Cluster observations to obtain the normal directions of two HFA boundaries and speeds along the normal directions. They found that 4 HFAs (out of 21) were contracting at a few tens km/s and 5 HFAs were expanding at a few tens to above one hundred km/s. The remaining 12 HFAs were stable without clear contraction or expansion. They explained that the difference in the sum of magnetic and thermal pressure across HFA boundaries determines whether HFAs contract, expand, or remain stable. Later, Liu et al. (2016b) also used four spacecraft timing method on five THEMIS spacecraft observations to calculate the boundary normal and speed along the normal for 6 HFAs. The HFAs were expanding at several tens to around one hundred km/s, smaller or comparable to the local fast wave speed, consistent with the fact that the HFAs did not have clear shocks as their boundaries. Sizes of the HFAs were 0.5-3 $R_{\mathrm{{E}}}$ based on the expansion speed. For comparison, sizes of 6 FBs in their studies were 2-15 $R_{\mathrm{{E}}}$ with super-fast-magnetosonic expansion consistent with simulation results that FBs are typically larger than HFAs and have shocks as their upstream boundaries (e.g., Omidi and Sibeck 2007; Omidi et al. 2010). In the midtail foreshock, the HFA reported by Liu et al. (2020e) was in a stable state while propagating tailward.

In addition to the spatial scale along the dimension that the spacecraft crossed or along the expansion direction, Zhang et al. (2010) estimated how far HFAs extend upstream from the bow shock. Based on the separation between two THEMIS spacecraft, the HFAs can extend to at least 9 $R_{\mathrm{{E}}}$ away from the bow shock. Similarly, in the midtail foreshock observed by two ARTEMIS spacecraft (Liu et al. 2020e), an HFA extended at least 4.7 $R_{\mathrm{{E}}}$ along the TD surface away from the bow shock. A statistical study by Chu et al. (2017) showed that HFAs were observed within 7 $R_{\mathrm{{E}}}$ upstream from the bow shock. Based on the normal direction of two boundaries, Schwartz et al. (2018) calculated that the two boundaries of an HFA intersected at 1 $R_{\mathrm{{E}}}$ upstream from the bow shock. By calculating the trajectory of ions leaked from HFAs based on dispersed ion distributions, Liu et al. (2017a) diagnosed the curved shape of HFA boundaries in three dimensions. They estimated that the HFA can extend to around 5 $R_{\mathrm{{E}}}$ upstream from the bow shock.

Zhang et al. (2010) compared HFAs in different evolution stages. Proto-HFAs are structures that later develop into HFAs. They exhibit decreases in magnetic field strength and plasma density, moderate plasma heating, and fluctuating, not very deflected ion bulk velocity. Young and mature HFAs are structures that have already become HFAs. The difference is that young HFAs have distinct two ion populations (solar wind and foreshock ions) whereas mature HFAs have one very diffuse ion population inside them (Lucek et al. 2004b). It is likely that the two ion populations release free energy between them and merge into one population, which could explain the increase in the amplitude of magnetic pulsations inside HFAs during the evolution from proto-HFAs ($\delta B/B <50\%$) to young HFAs ($\delta B/B \sim 1$) and to mature HFAs ($\delta B/B \sim 4$). A statistical study by Wang et al. (2013b) demonstrated that in addition to the difference in ion distributions, electron spectra inside young HFAs can be fitted by a single drift$-\kappa $ distribution, whereas in mature HFAs the spectra can be fitted by a combination of a heated population and a drift-Maxwellian distribution with peak energy below 10 eV. Using high resolution observations from the MMS spacecraft in a string-of-pearls formation, Liu et al. (2020a) illustrated the fast evolution of an HFA, SHFA, or FB that just started to form. During the event, two distinctive ion populations were identified, and the foreshock ions were demagnetized, resulting in a Hall current that determined the magnetic field structure. During the evolution, the mass flux of cold plasmas and magnetic flux were transported outward to the boundary. Solar wind ions became more deflected, and more foreshock ions were trapped within the structure.

HFAs have been observed at other planets too. Their size is controlled by the standoff distance of the bow shock and/or local solar wind conditions. Their size relative to the planet is similar to their terrestrial counterpart. At Mercury, the size is thousands of km comparable to 1 Mercury radius (Uritsky et al. 2014). At Venus, the size is 0.4 to 1.7 Venus radii (Collinson et al. 2014). The HFA observed at Mars is 0.66 Mars radii (Collinson et al. 2015). At Jupiter, the size is $2 \times 10^{6}\mbox{ km}$ (Valek et al. 2017). At Saturn, the spatial scale is ∼4.6 Saturn radii (Masters et al. 2009b).

#### Flow Deflection Inside HFAs

**Fig. 14:**
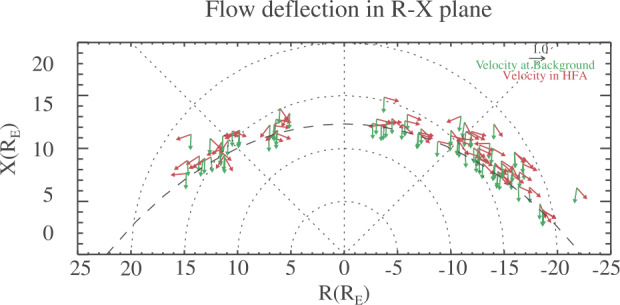
Flow deflections in HFAs. The green (red) arrows represent the background solar wind velocity (flow velocity at the center of HFAs). The length of the arrow represents the ratio between the velocity component in this direction and the speed. The absolute value of R is the distance to the Sun-Earth line, and the sign of R is in the same sense as the sign of GSE Y. The curved dashed line represents the normalized bow shock location. (From Wang et al. 2013a, Fig. 4)

**Fig. 15:**
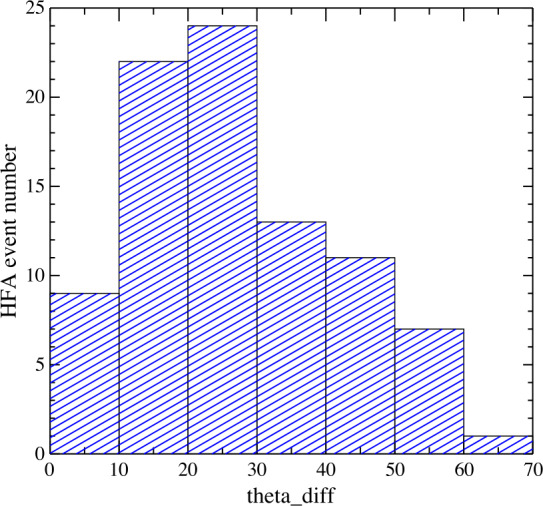
HFA event number distribution for the angle differences ($\theta _{\mathit{diff}}$) between the foreshock ion velocity direction (inferred from CIS data and an assumed foreshock ion density ratio) and the predicted direction of reflected ions based on specular reflection model (from Wang et al. 2013a, Fig. 9)

Wang et al. (2013a) examined 87 HFAs observed by Cluster C1 in which the magnitude of the GSE $V_{y}$ and $V_{z}$ deflections exceeded 200 km/s from 2003 to 2009. They found that the large flow deflections in HFAs strongly depend on the location (Fig. 14), i.e., the velocity direction change is away from the local bow shock normal direction. They interpreted that the deflection is due to the presence of reflected ions (foreshock ions). By assuming that the density ratio of reflected ions to solar wind ions was $20\%$, they estimated the velocity of reflected ions. They showed that the reflected ion velocity direction is close to the predicted near-specularly reflected direction from the bow shock (Fig. 15). They also showed that HFAs closer to the bow shock exhibit more significant $V_{x}$ decreases.

Case studies from THEMIS and MMS observations showed that the ion bulk velocity deflection is due to the presence of foreshock ions and the expansion or outward motion of solar wind ions (Liu et al. 2018, 2020a). Additionally, Liu et al. (2020a) found that the ion bulk velocity becomes more and more deflected during the evolution of a foreshock transient that just formed, and two reasons have been identified. One is that the solar wind ions are decelerated in the spacecraft frame or accelerated in the background solar wind rest frame likely driven by the increasing induced electric field. The other is that the density ratio of foreshock ions to solar wind ions increases, because more and more foreshock ions are trapped within the foreshock transient whereas solar wind ions are transported outward to the boundary (i.e., expansion).

#### Plasma, Magnetic Field, Waves and Turbulence Inside HFAs and SHFAs

Inside HFAs, the magnetic field is usually very turbulent with various wave activities. Inside a young HFA, Tjulin et al. (2008) identified two wave modes using $k$-filtering technique with multi-point Cluster observations. The two wave modes were at around 1 Hz with Poynting vector coinciding with the moving direction of solar wind ions and foreshock ions, respectively. They were interpreted as the combination of inherent fluctuations in the solar wind and foreshock. Because of the free energy between the two ion populations, the ion beam instability could enhance the wave amplitude inside the HFA until the two ion populations become one diffuse population, i.e., a young HFA evolves into a mature one.

From THEMIS observations, Zhang et al. (2010) also identified electromagnetic waves near lower hybrid frequency (e.g., 0.1-1 Hz) inside both young and mature HFAs using wavelet analysis. Lower hybrid waves were considered as one of the possible interpretations, which could contribute to electron heating inside HFAs. In addition, Zhang et al. (2010) examined lower frequency waves (0.03 Hz in the spacecraft frame). They showed that inside a proto-HFA the waves were quasi-parallel propagating (wave normal angle $18.9^{\circ }$) in the sunward direction, left hand polarized in the spacecraft frame, but right hand polarized in the solar wind rest frame. The fluctuations of magnetic field strength and density were correlated with relatively small amplitude ($\delta B/B <50\%$). Similar waves were also observed inside young HFAs but with larger amplitude ($\delta B/B \sim 1$). Inside mature HFAs, magnetic pulsations with very large amplitude ($\delta B/B \sim 4$) were observed. These waves inside proto-, young, and mature HFAs correspond to the early, middle, and late (nonlinear) stages of the right-hand resonant instabilities, which could play a role in thermalizing ions. These “30s waves” are common in Earth’s foreshock (Wilson 2016).

Right outside HFAs, gyrophase-bunched ions with energy dispersion and gyrophase evolution are sometimes observed (e.g., Tjulin et al. 2009; Liu et al. 2017a). Cluster observations (Tjulin et al. 2009) showed that outside the leading boundary of an HFA such ions were accompanied by waves at frequencies varying from 0.5 to 1 Hz propagating obliquely without clear polarization. Outside the trailing boundary without those ions, on the other hand, the waves were at nearly constant 1.5 Hz quasi-parallel propagating with left hand polarization in the spacecraft frame. Since the background conditions on two sides of the HFA are similar, different properties of the two wave modes are possibly caused by the gyrophase-bunched ions outside the leading boundary through the mechanism described in Wong and Goldstein (1987, 1988). The waves outside the trailing boundary, on the other hand, are consistent with “1 Hz waves” commonly observed in Earth’s foreshock (Wilson 2016).

From THEMIS observations, Shi et al. (2020a) identified broadband quasi-parallel propagating whistler waves at around half electron gyrofrequency (several tens to 100 Hz) inside the compressional boundary of HFAs and FBs. The power spectral density contours of electron distributions followed the diffusion surface very well, suggesting that the electrons were efficiently scattered by the whistler waves. The calculated pitch angle diffusion coefficient was large enough compared to the time scale that electrons spent in the compressional boundary (several seconds). The excitation mechanism is that during the formation and evolution of an HFA or FB, the magnetic flux is transported outward resulting in betatron acceleration at the compressional boundary (Liu et al. 2019a, 2020a), which can increase the electron perpendicular anisotropy, leading to the generation of whistler waves.

**Fig. 16:**
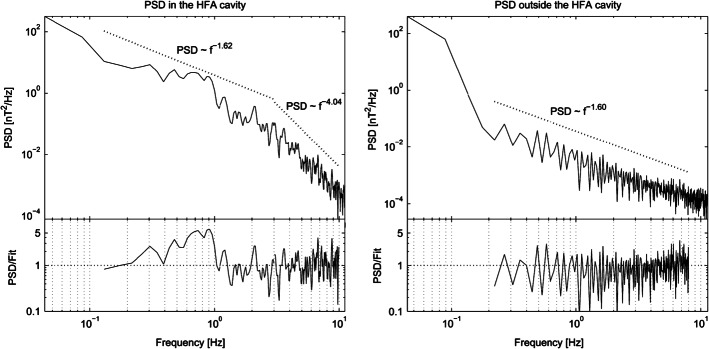
Power spectral density (PSD) of the magnetic field time-series recorded by the Cluster SC2 spacecraft inside and before an HFA. The bottom panels show the differences between the PSD and the fitted power-law curves. (From Kovács et al. 2014, Fig. 6)

Kovács et al. (2014) analyzed turbulence dynamics inside an HFA observed by Cluster. Figure 16 shows the power spectral density of the magnetic field inside and before the HFA. Both show a power-law character with a spectral slope close to that of Kolmogorov spectra, except the spectrum inside the HFA shows a spectral break at around 3 Hz and then steepens dramatically. Inside the HFA, the power-law approach fails to accurately fit the spectrum between about 0.4 and 1 Hz, likely caused by considerable wave activities discussed above and the plasma heating. Considering that the lower hybrid frequency was around 3.2 Hz, lower hybrid waves could play a role in electron heating (Zhang et al. 2010) before the break, whereas beyond the break another cascade process emerges involving energy remnant after the considerable plasma heating.

Kovács et al. (2014) also examined the temporal increments of single-spacecraft and spatial differences of simultaneous multi-spacecraft magnetic field records of the HFA. They employed sliding-window analysis in which moving overlapped sequences of the non-stationary signal were analyzed separately. Due to the presence of the low-frequency and large-amplitude waves, they applied high-pass filtering to minimize the contribution from the waves. They concluded that the filtering considerably enhanced the non-Gaussian character of the HFA magnetic time-series and confirmed the prevailing nature of intermittent multi-scale processes in the HFA. The strongest intermittency appeared at the compressional boundary of the HFA. The high-frequency components of the HFA magnetic fluctuations exhibited spatial coherency among the Cluster spacecraft.

#### Reconnection Inside HFAs

Direct and indirect evidence has shown that magnetic reconnection can occur inside HFAs. A magnetic flux rope was identified inside a magnetosheath HFA observed by Cluster (Hasegawa et al. 2012). Properties of the identified flux rope, including its low velocity with a sunward component, the absence of magnetospheric electrons, and magnetic field variations, indicate that it was created by magnetic reconnection inside the magnetosheath HFA. A flux rope was also identified inside the compressional boundary of an HFA observed by MMS in the foreshock (Bai et al. 2020). Observations by Cluster, THEMIS, and MMS have revealed various types of reconnection induced in the magnetosheath and at the bow shock: reconnection due to compression of a non-reconnecting solar wind current sheet at the bow shock (Phan et al. 2007), reconnection spontaneously generated in the transition region of the bow shock (Wang et al. 2019a; Gingell et al. 2019), reconnection close to the magnetopause due to compression of a magnetosheath current sheet against the dayside magnetopause (Phan et al. 2011), and reconnection in current sheets of the turbulent magnetosheath downstream of the quasi-parallel bow shock (Retinò et al. 2007; Phan et al. 2018). Upstream from the bow shock, reconnection was also identified inside HFAs and FBs from recent MMS observations (Liu et al. 2020d). The reconnection occurred in micro-scale current sheets of thickness comparable to or less than one ion inertial length with a super-ion-Alfvénic electron outflow, positive $\mathbf {j} \cdot \mathbf {E'}$, and the electron temperature increases without clear ion coupling. The possible generation mechanism could be compressed solar wind currents or turbulence in HFAs and FBs, which requires further investigation. Additionally, reconnecting current sheets inside SLAMS have been identified from recent MMS observations (Wang et al. 2020a).

#### Ion and Electron Heating and Acceleration Inside HFAs

By definition, plasma is heated/thermalized inside HFAs (and FBs). As for ions, a statistical study based on Cluster measurements showed a strong correlation between the increase in thermal energy and decrease in kinetic energy, indicating that the thermal energy of HFAs is mainly converted from the kinetic energy of the coupled solar wind and foreshock ions (Wang et al. 2013b). This is consistent with what Thomsen et al. (1988) suggested. A recent PIC simulations by An et al. (2020) examined how foreshock ions couple with solar wind ions, electrons, and the magnetic field through the electric field during the formation process of HFAs/FBs. As foreshock ions gyrate out from a discontinuity, a static electric field arises due to the gyroradius difference between foreshock ions and electrons. Such a static electric field decreases ion energy and increases electron energy. Meanwhile, the evolution of the magnetic structure induces an electric field. The Hall current due to demagnetized foreshock ion motion and magnetized electron motion is against the induced electric field, transferring energy to the magnetic field. The induced electric field also drives the frozen-in plasma to move outward from the HFA/FB core, i.e. expansion, which is one reason for observed plasma deflection. Overall, in the solar wind rest frame, the foreshock ions provide energy to heat and accelerate the solar wind ions and electrons and build up magnetic field structures. Such a process is supported by MMS observations recently reported by Liu et al. (2020a). The statistical study by Liu et al. (2017b) showed that ion energies are generally lower inside than outside HFAs and FBs, also suggesting that the foreshock ions are the energy source. In addition, because of the free energy available between the counterstreaming solar wind ions and foreshock ion populations, Zhang et al. (2010) suggested that right-hand resonant instabilities could occur and thermalize ions, causing two distinct ion populations (young HFAs) to merge into one very diffuse population (mature HFAs). MMS observations by Schwartz et al. (2018) found that the solar wind helium ions also contribute to the ion heating. As for electrons, PIC simulations by An et al. (2020) showed that the electron heating is intrinsic during the formation process caused by the electrostatic field. Based on observed wave activities, Zhang et al. (2010) suggested that lower hybrid instabilities could contribute to the electron heating. Depending on the temperature anisotropy, the firehose instability (Eastwood et al. 2008) or whistler waves (Shi et al. 2020a) can isotropize electrons.

In addition to particle heating/thermalization, particle acceleration is also common for HFAs/FBs. Particle acceleration by foreshock transients was first found at SLAMS (Kis et al. 2013; Wilson et al. 2013). Later, Wilson et al. (2016) identified relativistic electrons inside HFAs/FBs, indicating their significant acceleration ability. A statistical study by Liu et al. (2017b) showed that suprathermal electron energies are greater inside than outside almost all HFAs and FBs and these energies are correlated with solar wind speeds. Some of them can reach 100s of keV. Foreshock ion energies are also greater inside than outside some events, suggesting additional ion acceleration, and the energy is also proportional to the solar wind speed. Multiple acceleration mechanisms have been identified. One mechanism is Fermi acceleration. As the upstream compressional boundary of an HFA/FB convects earthward towards the bow shock, i.e., they are converging, particles can bounce between the bow shock and the inner edge of the compressional boundary to gain energy. Note that if the bouncing is between the boundaries of an HFA/FB, as the boundaries move away from each other, particles lose energy to support the expansion. Using THEMIS observations in comparison with test particle simulations, Liu et al. (2017d) showed the evidence of electron Fermi acceleration. Because of wave-induced pitch-angle scattering, the efficiency of acceleration decreases by 2/3 as the increasing parallel energy is scattered to the perpendicular direction. Using THEMIS observations and particle tracing in a 3D global hybrid simulation, Liu et al. (2018) showed that ions can at least complete one bounce between the bow shock and the upstream boundary. By comparing MMS observations with theoretical prediction, Turner et al. (2018) presented the ion Fermi acceleration process which accelerates protons and heavy ions to energies of around 200 keV and almost 1 MeV, respectively. During the acceleration process, due to their very large gyroradius, some of the accelerated ions leaked out both into the ambient solar wind and magnetosheath. Lee et al. (2020) provided an alternative explanation for these energetic ions observed upstream from the bow shock and in the magnetosheath. They suggested that the energetic ions have escaped from the outer magnetosphere. Liu et al. (2017a) examined the energetic ion leakage from HFAs using THEMIS observations and found that the observations agree well with single particle motion model. Using hyrbid simulations and test particle simulations, Omidi et al. (2021) examined ion acceleration inside FBs and identified second-order Fermi acceleration.

Liu et al. (2019a) employed hybrid simulations and THEMIS observations to show that magnetic flux is transported outward during the formation and expansion process, resulting in a low-field strength core and compressional boundaries. During the magnetic flux transport, electrons can be locally accelerated by betatron acceleration at the compressional boundary. After the acceleration, some of the electrons can move along the field lines into the core region without losing energy but with evolving pitch angles. As the field lines are continuously transported from the core to the boundary, these electrons can experience another instance of betatron acceleration. Such a process can further energize foreshock electrons from 10s of keV to 100s of keV. If considering the wave scattering effect, the acceleration efficiency decreases by 1/3, as the increasing perpendicular energy is scattered to the parallel direction. Such a process can also work for HFAs. From recent MMS observations, Liu et al. (2020a) identified a similar process for thermal electrons during the formation of foreshock transients, which enhances electron perpendicular anisotropy, leading to the excitation of whistler waves that in return decreases the anisotropy (Shi et al. 2020a). If the expansion of an HFA/FB is super-fast-magnetosonic, the compressional boundary can steepen into a shock. Such a shock has been observed to accelerate solar wind ions through shock drift acceleration and form a new foreshock (Liu et al. 2016a). Inside HFAs/FBs, magnetic reconnection has been identified using MMS observations, which energizes electrons through the parallel electric field along the guide field (Liu et al. 2020d).

Particle acceleration by HFAs/FBs could contribute to shock acceleration. One important shock acceleration mechanism is diffusive shock acceleration (Treumann 2009; Lee et al. 2012). However, there are still some open questions. For example, particles need certain initial energy to participate in the acceleration, but the energy source is unknown. In addition, the theoretical acceleration efficiency at Q$_{\parallel }$ shocks is likely underestimated. It is possible that foreshock transients could be one of the energy sources to initiate diffusive shock acceleration. For example, the shocks of FBs and HFAs can first accelerate solar wind particles through shock drift acceleration and bring them towards the bow shock for further acceleration (Liu et al. 2016a). Foreshock transients could also increase the acceleration efficiency at Q$_{\parallel }$ shocks. For example, FBs and HFAs can directly participate in the Fermi acceleration process at the bow shock (Liu et al. 2017d, 2018; Turner et al. 2018; Omidi et al. 2021). They can also further energize particles that are accelerated at the bow shock (Liu et al. 2019a). As foreshock transients are very likely common in the universe, the role of foreshock transients in shock acceleration should be considered.

#### Discovery of SHFAs and Their Parametric Dependencies

**Fig. 17:**
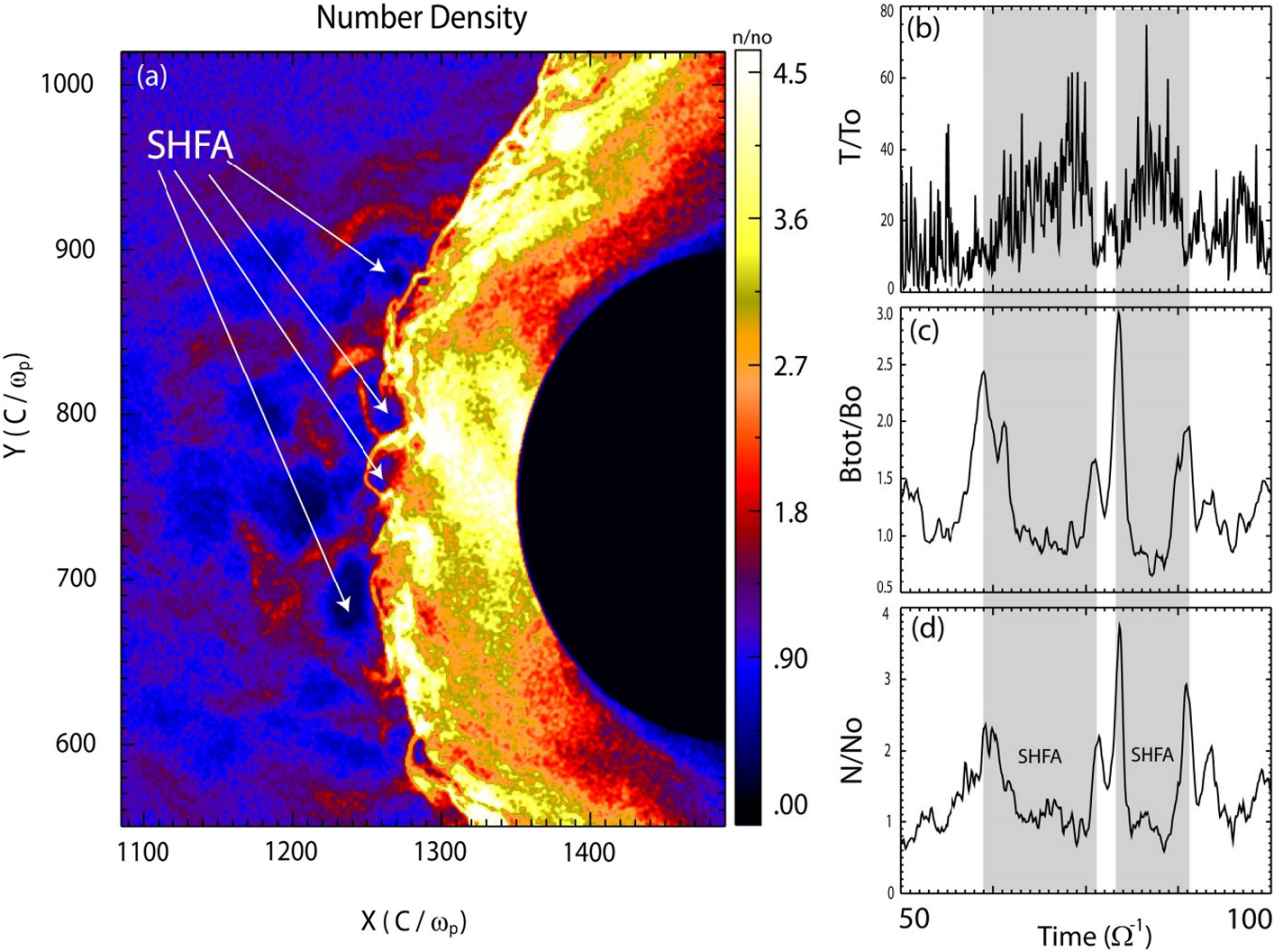
Results of a global hybrid run during radial IMF. The configuration is similar to that in Fig. 2 of Omidi et al. (2020b). (**a**) density normalized by that of the solar wind focused around the quasi-parallel shock and magnetosheath. (**b**)-(**d**) ion temperature, magnetic field strength, and density as measured by a simulated spacecraft located at X= 1250, Y= 800. The shadowed regions correspond to the detection of two SHFAs. (Credit: Nick Omidi)

Omidi et al. (2013b) used global hybrid simulations to demonstrate that the convection of foreshock cavitons and their interaction with the bow shock results in the formation of SHFAs. Figure 17 shows examples of SHFAs formed in a hybrid run with solar wind Alfvén Mach number $M_{\mathrm{{A}}}=$ 7 and radial IMF. Panel (a) shows the density normalized by that of the solar wind focused around the quasi-parallel shock and magnetosheath. Examples of SHFAs characterized by cores of low density and rims of high density are evident in the figure. Panels (b)-(d) show ion temperature, total magnetic field, and density as measured by a simulated spacecraft located at X= 1250, Y= 800 respectively. The shadowed regions correspond to the detection of two SHFAs whose time series signatures resemble that of HFAs. Subsequent studies using global hybrid simulations have demonstrated that SHFAs form at shocks with $M_{\mathrm{{A}}} \geq $3 regardless of the IMF cone angle and that they are an inherent part of ion dissipation processes at the quasi-parallel bow shock with significant impacts on the magnetosheath and the magnetopause such as the formation of Magnetosheath Filamentary Structures (MFS) and sheath cavities (Omidi et al. 2014a,b, 2016). Hybrid-Vlasov global simulations by Blanco-Cano et al. (2018) also showed SHFAs evolved from foreshock cavitons and affected the local bow shock and magnetosheath consistent with Omidi et al. (2013b).

Zhang et al. (2013) reported an SHFA during different evolution stages observed by five THEMIS spacecraft. THEMIS A observed a structure near the bow shock which exhibited a core region with low field strength, low density, strong plasma heating, and significant plasma deflection bounded by two compressional boundaries (Fig. 3). The characteristics are consistent with typical HFAs, except that there is no clear magnetic field direction change across the structure. Therefore, the HFA-like structure was not driven by a discontinuity and must thus be identified as an SHFA. The multiple THEMIS spacecraft also observed the early stage of the SHFA, i.e., a proto-SHFA. Similar to proto-HFAs, the proto-SHFA did not show clear plasma heating and/or significant flow deflection and exhibited two distinctive ion populations (foreshock and solar wind ions) inside it. The proto-SHFA showed depressed magnetic field strength and plasma density, similar to cavitons in hybrid simulations performed by Omidi et al. (2013b). The observations are consistent with the hybrid simulations by Omidi et al. (2013b), confirming that HFAs can spontaneously form at the quasi-parallel bow shock. Collinson et al. (2017) reported ESA Venus Express and NASA Mars Atmosphere and Volatile EvolutioN (MAVEN) observations indicating that SHFAs also exist in the foreshocks of Venus and Mars. These SHFAs form via the same mechanisms as those in the terrestrial foreshock according to 3-D hybrid simulations reported by Omidi et al. (2017a). Statistical studies (Chu et al. 2017; Wang et al. 2013b) showed that there are no significant differences between SHFA and HFA properties and occurrence patterns.

#### When/Where do HFAs Occur—Favorable Formation Conditions

HFAs typically form at the Q$_{\parallel }$ bow shock. For example, global hybrid simulations by Lin (2002) showed HFAs driven by a TD with a Q$_{\parallel }$ bow shock on two sides. Global hybrid simulations by Omidi and Sibeck (2007) used a TD that changed the local bow shock from Q$_{\parallel }$ to Q_⊥_. An HFA formed on the Q$_{\parallel }$ side. Observations show that most HFAs have Q$_{\parallel }$ bow shock on at least one side, and a few HFAs have Q_⊥_ bow shock on both sides (Schwartz et al. 2000; Facskó et al. 2009; Wang et al. 2013b). One possibility is that as the bow shock is curved, HFAs may form in the Q$_{\parallel }$ region and propagate to the spacecraft in the Q_⊥_ region.

Regarding the solar wind conditions, Cluster observations suggest that HFAs occur preferentially during fast solar wind (Facskó et al. 2008, 2009, 2010). Such a formation condition was later confirmed by THEMIS and ARTEMIS observations in the midtail foreshock at $X=-30$ to −50 $R_{\mathrm{{E}}}$. This may also be true for SHFAs and FBs, from both statistical studies (Chu et al. 2017; Liu et al. 2017b, 2021) and case studies (e.g., Turner et al. 2013; Liu et al. 2016b). A possible explanation is that faster solar wind causes higher foreshock ion speed, and the foreshock ion energy is the energy source for the formation process (e.g., An et al. 2020; Liu et al. 2020a). A statistical study using Cluster observations (Facskó et al. 2009) suggested that the dynamic pressure is not an important factor. The solar wind densities before HFAs were slightly lower than the average value of the solar wind density (Facskó et al. 2009). Statistical studies using THEMIS (Liu et al. 2017b) and ARTEMIS (Liu et al. 2021) observations also found that the solar wind density does not affect the HFA, SHFA, and FB occurrence, but that low IMF strengths favor occurrence. High fast magnetosonic Mach number ($M_{\mathrm{{MS}}}$) also favors the formation of HFAs. No events were found below $M_{\mathrm{{MS}}} = 6$ (Facskó et al. 2008, 2009, 2010). Similarly, recent hybrid simulations by Omidi et al. (2020b) showed that when the Alfvén Mach number is larger than $\sim 7$, FBs can form. The statistical study of foreshock transients in the midtail foreshock also showed that high solar wind Alfvén Mach number is a favorable condition (Liu et al. 2021). PIC simulations by An et al. (2020) suggested that high Mach numbers favor the formation and expansion of HFAs and FBs by providing larger foreshock ion energy and greater density ratios of foreshock to solar wind ions. Consistently, THEMIS and MMS observations showed that the expansion speed of FBs is proportional to the solar wind speed and Alfvén Mach number (Liu et al. 2016b; Turner et al. 2020).

**Fig. 18:**
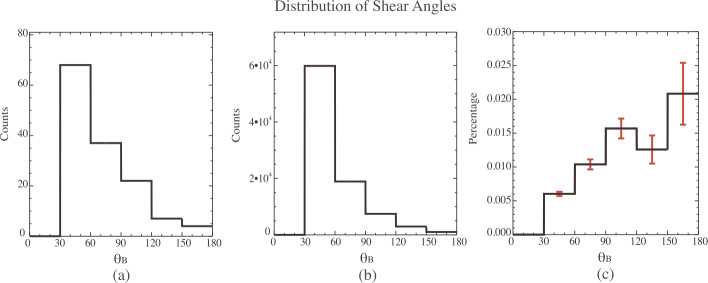
Statistical analysis of shear angle distributions. (**a**) Shear angle distribution of 138 HFA events. (**b**) Shear angle distribution of 90135 discontinuities in the solar wind. (**c**) Normalized shear angle distribution of HFAs by that in the solar wind (from Zhao et al. 2017, Fig. 6)

HFAs are typically driven by TDs that satisfy certain conditions. Schwartz et al. (2000) showed that there is a slight tendency for HFAs to occur preferentially for ∼ 90^∘^ shear angle (the angle between the magnetic field downstream and upstream of the driver TDs). Facskó et al. (2009, 2010) showed that HFAs preferentially occur at large shear angles of ${\sim} 70^{ \circ }$. Later, Zhao et al. (2017) confirmed that large shear angles favor the formation of HFAs (Fig. 18). The statistical study of midtail foreshock transients showed the same preference (Liu et al. 2021). Moreover, Schwartz et al. (2000) showed that a large angle between the TD normal and the Earth-Sun direction is a favorable condition. This was confirmed by Facskó et al. (2008, 2009, 2010) who showed that this angle is larger than $45^{\circ }$ in the majority of HFAs. Schwartz et al. (2000) suggested an explanation by using the following formula to calculate the transit velocity of the TD on the surface of the bow shock:

1 $$ \mathbf {V_{tr}}= \frac{\mathbf {V_{sw}}\cdot \mathbf {n_{cs}}}{\sin ^{2}\theta _{cs:bs}}( \mathbf {n_{cs}}-\cos \theta _{cs:bs}\mathbf {n_{bs}}) $$

where $\mathbf {V_{tr}}$ is the transit velocity, $\mathbf {V_{sw}}$ is the solar wind velocity, $\mathbf {n_{cs}}$ is the normal of the TD, $\mathbf {n_{bs}}$ is the normal of the bow shock, and $\theta _{cs:bs}$ is the angle between $\mathbf {n_{cs}}$ and $\mathbf {n_{bs}}$. When $\theta _{cs:bs}$ is large, the transit velocity is slow meaning that the driver TD has sufficient time to trap foreshock ions to form an HFA. The statistical analysis by Facskó et al. (2009, 2010) confirmed this statement. Hybrid simulations for the heliospheric termination shock by Giacalone and Burgess (2010) showed that large $\theta _{cs:bs}$ favors the formation of HFAs. The statistical study of midtail foreshock transients by Liu et al. (2021) supports this. However, Liu et al. (2021) pointed out that as the midtail bow shock is very tilted, the preference to large $\theta _{cs:bs}$ cannot result in slow $\mathbf {V_{tr}}$ due to the strong tangential component of the solar wind velocity. How exactly TD parameters affect the formation process requires further investigation.

In early studies, Burgess (1989b) used test particle simulations showing convection electric fields pointing towards a TD to show that TDs can trap bow shock-reflected ions along the TD. This was later confirmed by hybrid simulations (e.g., Thomas et al. 1991; Lin 1997) and observations (e.g., Schwartz et al. 2000) that such a field configuration favors the formation of HFAs. However, a statistical study by Wang et al. (2013b) showed that the electric field pointing towards TDs is not a necessary condition for HFA formation. Later, Zhao et al. (2017) showed convection electric fields point inwards toward TD on the leading and trailing sides of 74% and 72% of HFAs, respectively. Decreases in plasma parameters and the magnetic field strength within HFAs exhibiting inward convection electric field on both sides are larger than those with convection electric field inward on only one side. The formation model presented by Liu et al. (2020a) suggested that since the solar wind velocity is always anti-sunward, the direction of the convection electric field relative to the TD normal is determined by the IMF direction relative to the TD normal, which is independent of the frame of reference. When the convection electric field points towards the TD, the corresponding IMF configuration relative to the TD allows the Hall current from demagnetized foreshock ions to increase and thus enables the growth of the magnetic field structure, i.e., the formation of a foreshock transient.

Additionally, Zhao et al. (2017) showed that the formation of HFAs requires the magnetic field on at least one side of the TDs to be connected to the bow shock. They also calculated the thickness of TDs by fitting with the Harris current sheet model. They showed that thicknesses of TDs and HFAs are strongly correlated and thinner TDs form HFAs more efficiently. They also showed that HFAs preferentially form when the calculated specularly reflected flow from the bow shock is along the TD plane.

HFAs and their magnetosheath perturbations (e.g., Eastwood et al. 2008; Hasegawa et al. 2012) have been observed not only on the dayside but also on the nightside. Facskó et al. (2015) reported HFA-like disturbances in the far tail magnetosheath using STEREO magnetic field and electron plasma measurements. Recently, multiple spacecraft observations showed that the disturbances associated with foreshock transient events can propagate to the midtail magnetosheath and affect the nightside magnetopause (Wang et al. 2018a). Using ARTEMIS observations, Liu et al. (2020e) identified HFAs and other types of foreshock transients in the midtail foreshock, which were statistically studied by Liu et al. (2021). Using 3D global hybrid simulations, Wang et al. (2020b) simulated an FB formed in the dayside foreshock and propagated to the midtail foreshock. These results suggest that HFAs and other types of foreshock transients can disturb not only the dayside but also the nightside bow shock, magnetosheath, and magnetopause.

#### Hydromagnetic HFA Formation Mechanism

One of the most remarkable properties of HFAs is the strong deflection of the solar wind bulk flow which can be large enough that inside an HFA the flow can actually show a sunward component. While there is always the presence of back streaming ions in the foreshock region of the Earth’s bow shock, the momentum and energy transport of these foreshock particles is far to small too explain HFA properties for typical conditions at the bow shock. This consideration motivated a very different approach to explain the presence of HFAs at the bow shock. It is straightforward to demonstrate that a transient region of lower density in the solar wind interacting with the fast shock can cause the disruption of the fast shock and leads to a new shock that actually travels into the upstream direction with plasma behind this new shock having a much smaller momentum density and velocity than the original solar wind.

Using two-dimensional MHD simulations Otto and Zhang (2021) modeled such a scenario by assuming the presence of a low density flux tube in the upstream solar wind region. The initial configuration used in these simulations was that of an oblique fast shock and a low density magnetic flux tube in the upstream region that is convected into the fast shock. As soon as the low density flux tube touches the fast shock, a bulge forms at the shock that expands rapidly into the upstream region. It is noted that in one dimension with a simple parallel shock this problem can be solved analytically demonstrating the newly formed fast shock moved fast into the upstream region with a speed that depends on the solar wind speed (or Mach number) and the reduction in density in the depleted flux tube, i.e., the sunward velocity of the newly formed shock is faster for faster solar wind and for stronger reduction in density. For a reduction to about 20% of the original density the speed of the newly formed shock is close to 1/2 of the solar wind speed and the material behind the newly formed shock is stagnant (in the original shock frame).

**Fig. 19:**
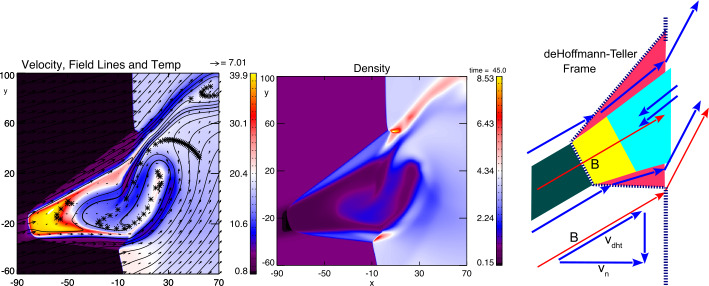
Left: Temperature (color), velocity (arrows), and magnetic field (lines); middle: Density (color); right: Sketch of the different characteristic regions of the HFA-like structure as a result of the interaction of a density depleted flux tube (dark green) with a fast oblique shock (vertical dashed line) for the entropy distribution in our reference case. Here, red indicates leading and trailing shocked plasma regions, yellow indicates the shocked plasma from the density depleted flux tube, and cyan is the inner region of plasma that is a mixture of original magnetosheath material and plasma from the leading edge region which is channeled back into the interior through a vortex motion. (after Otto and Zhang 2021)

Figure 19 illustrates the resulting bulge of the interaction of the low density flux tube with a fast shock about 3 minutes into the simulation. Here the solar wind Mach number is 5, solar wind speed is 500 km/s, density is normalized to 3 cm^−3^, and distances are in units of 300 km. The density plot shows the low density at the outermost edge of the bulge which extends about 4 $R_{\mathrm{{E}}}$ out of the original fast shock with $\Theta _{Bn}=15^{\circ }$.

The figure illustrates a number of typical properties that are consistent with observations of HFAs. The interior of the HFA-like structure is strongly heated and almost stagnant. Note that this simulation is in the de Hoffmann-Teller frame of the original shock in which the HFA is just expanding but not moving along the original shock. In the normal incidence frame the structure is actually moving downward along the shock with a velocity that depends on the solar wind speed and $\Theta _{Bn}$ as indicated in the sketch on the right. This also implies that average interior plasma velocities of HFAs should depend on the inclination of the magnetic field with the shock and the solar wind speed.

Asterisks in the left panel of Fig. 19 show fluid elements that were originally placed along a straight line with uniform spacing. These demonstrate a strong vortical motion which is caused by the flow deflection around the bulge. It is also noted that the leading and trailing edges of the structure show significant density increases consistent with shocks at these boundaries. The pressure in the interior is higher than in the solar wind, leading to the expansion along the original shock, and it is lower than in the adjacent magnetosheath (downstream region) causing a flow of magnetosheath material into the rear part of the HFA-like structure.

The typical regions of this HFA structure are illustrated in the sketch on the right of Fig. 19. These different colored regions and their boundaries are clearly seen in the entropy and density plots in Fig. 19 where, for instance, the leading and trailing shocked regions are dark violet in the entropy and light blue in the density plot. Actual in-situ observation of these structures should show an asymmetry because the trailing edge encounter takes place at a time where the structure is older such that the trailing edge shocked region should be more developed and thicker while the leading edge shock may not have fully developed at the time of the encounter. Also, the interior region is separated from the leading and trailing shocked plasma by tangential discontinuities, and it is a region of strongly varying magnetic field and pressure generating current layers, strong enough to cause magnetic reconnection inside the HFA-like structure in the simulation.

While different solar wind conditions and values of $\Theta _{Bn}$ have an influence on the evolution (faster and hotter for higher solar wind speed) the qualitative structure does not depend on the specific values used for the simulations. An open question for this mechanism concerns the origin of the low density flux tube. However density cavities are fairly frequent in the vicinity of the foreshock region such that it is conceivable that such cavities can interact with the bow shock preferably for fairly radial IMF conditions. The simulations also demonstrate that the HFA-like structure grows much faster for small $\Theta _{Bn}$ consistent with statistical properties of HFA occurrence. Clearly these simulation cannot provide the observed rich kinetic structure of HFAs but they appear to be consistent with the observed bulk properties. It would also be highly desirable to conduct similar studies using kinetic simulation models.

#### Kinetic Formation Model

Hybrid simulations (e.g., Thomas et al. 1991; Lin 2002; Omidi and Sibeck 2007; Omidi et al. 2010) show that HFAs and FBs form when foreshock ions interact with a solar wind discontinuity. The discontinuity can concentrate and thermalize foreshock ions resulting in high thermal pressure, which causes an expansion that piles up plasma outward forming a low density core bounded by compressional boundaries or shocks. To explain how foreshock ions interact with a discontinuity, Archer et al. (2015) proposed that when foreshock ions cross an RD, their parallel speed has to be projected to the perpendicular direction due to the magnetic field direction change. This leads to a conversion from the kinetic energy to the perpendicular thermal energy. Additionally, because of the decrease in parallel speed and conservation of mass flux of foreshock ions across the discontinuity, the density of foreshock ions increases. Both the increases in foreshock ion density and thermal energy result in a large thermal pressure enhancement. Similarly, Liu et al. (2015) proposed that when foreshock ions gyrate across a tangential discontinuity due to their large gyroradius, the tangential discontinuity (under a certain IMF configuration) can also transfer foreshock ion kinetic energy to thermal energy.

**Fig. 20:**
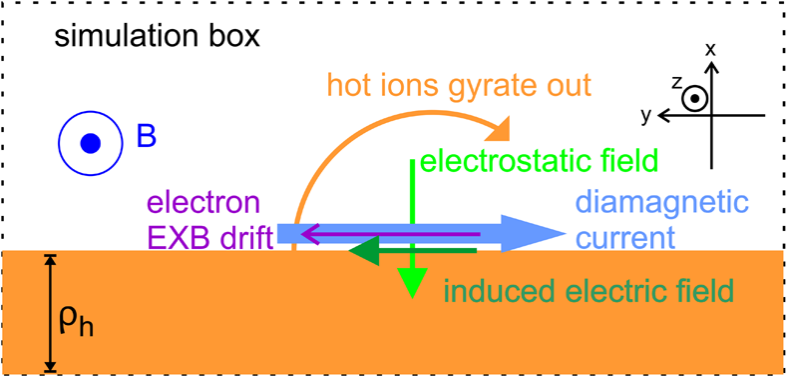
The sketch of formation process. The orange region fills with both foreshock (hot) ions and cold plasma with thickness same as foreshock ion gyroradius ($\rho _{h}$). The white region only has cold plasma. (From An et al. 2020, Fig. 1)

These models, however, are still insufficient. For example, foreshock ion gyroradii can be thousands of km or even several $R_{\mathrm{{E}}}$ in the core region with low field strength, which are larger than or comparable to the discontinuity thickness or HFA/FB spatial scale. The ion gyroperiod (10-20 s) can also be larger than or comparable to the formation/evolution time scale of HFAs/FBs. Therefore, the concept of “thermal pressure” is not suitable to describe foreshock ions and their kinetic effects should be considered. As shown in Fig. 20, greater ion than electron gyroradii cause inward pointing static electric fields to accompany discontinuities (An et al. 2020). Such an electrostatic field drives electrons to ${\mathbf{E}} \times {\mathbf{B}}$ drift, but ions cannot drift because this process happens within one ion gyroperiod. The electron motion together with the partial gyration of foreshock ions results in a Hall current, which decreases the field strength in the core region and increases the field strength at the boundary. Because of the magnetic field variation, there is an induced electric field, which drives cold plasmas to expand outward together with the magnetic field lines. This model provides a more physical description of the formation and expansion process of HFAs/FBs. The energy comes from the foreshock ions through partial gyration against the induced electric field. As a result, higher foreshock ion energy can lead to faster expansion as shown in their parameter scan. Using a more realistic setup, An et al. (2020) showed that when initially field-aligned foreshock ions cross an RD, they cannot immediately change their velocity direction and start to gyrate partially. In addition, because electrons are almost always magnetized and move along the field lines, Hall currents form which determine the basic magnetic profile of an FB.

Liu et al. (2020a) confirmed this model by analyzing MMS observations of foreshock transients that just started to form. They showed that in the background foreshock, foreshock ions move along the field lines with complete gyration and ${\mathbf{E}} \times {\mathbf{B}}$ drift. In the core region, although the magnetic field direction has changed, the foreshock ions still move roughly in the same direction as in the background. As a result, the foreshock ions’ initial parallel speed is projected to the perpendicular direction, i.e., the foreshock ions partially gyrate. Because electrons are almost always magnetized, a Hall current forms. Such a Hall current curves the magnetic field lines, inducing an electric field that drives the cold plasma to ${\mathbf{E}} \times {\mathbf{B}}$ drift outward together with the field lines. As the mass flux and magnetic flux are transported from the core to the boundary, the boundary steepens with enhanced field strength. As a result, more foreshock ions are trapped within the core region leading to a stronger Hall current, which in return further steepens the boundary. This completes a positive feedback loop resulting in a kind of “instability” that enables the structure to grow. As this is an “instability”, the magnetic field variation is nonlinear causing the induced electric field to increase, which drives the cold plasma to move outward faster and faster. This explains how the expansion speed of HFAs/FBs accelerates from 0 to a certain value.

Based on this model, Liu et al. (2020a) suggested that to form an HFA/FB, a certain magnetic field configuration across a discontinuity is needed. For example, when the Hall current can decrease the field strength at the discontinuity, foreshock ions can more easily cross the discontinuity and become more demagnetized due to locally larger gyroradii, which enhances the Hall current, i.e., an “instability” can occur, and an HFA/FB starts to develop. However, if the Hall current increases the field strength at the discontinuity, fewer foreshock ions can cross the discontinuity and become less demagnetized, and a stable solution will be reached, resulting in a static modification to the magnetic profile around the discontinuity. For tangential discontinuities, the two magnetic field configurations have a convection electric field in the bow shock rest frame pointing toward and away from the discontinuity, respectively. Thus, this model can also partially explain why the convection electric field needs to point towards the tangential discontinuity on at least one side to form HFAs (e.g., Schwartz et al. 2000).

### Other Foreshock Transients

#### Foreshock Bubbles

Using the results from global hybrid simulations of solar wind interaction with the magnetosphere, Omidi et al. (2010) predicated the formation of a new non-linear structure named foreshock bubble (FB). Foreshock bubbles form when IMF discontinuities interact with backstreaming ion beams in the foreshock. In contrast to HFAs that form after the interaction of interplanetary discontinuities with the bow shock, FBs can form prior to such an interaction. FB formation is initiated when the discontinuity encounters the backstreaming ion beam resulting in its deflection due to the magnetic field direction change. The deflected beam interacts with the solar wind, resulting in the deceleration of the latter and the formation of a fast magnetosonic shock wave that expands sunward with time. Downstream of the shock wave there is a sheath (shocked solar wind) plasma followed by a core region with low magnetic field strength and containing a hot and tenuous plasma with energetic particles.

**Fig. 21:**
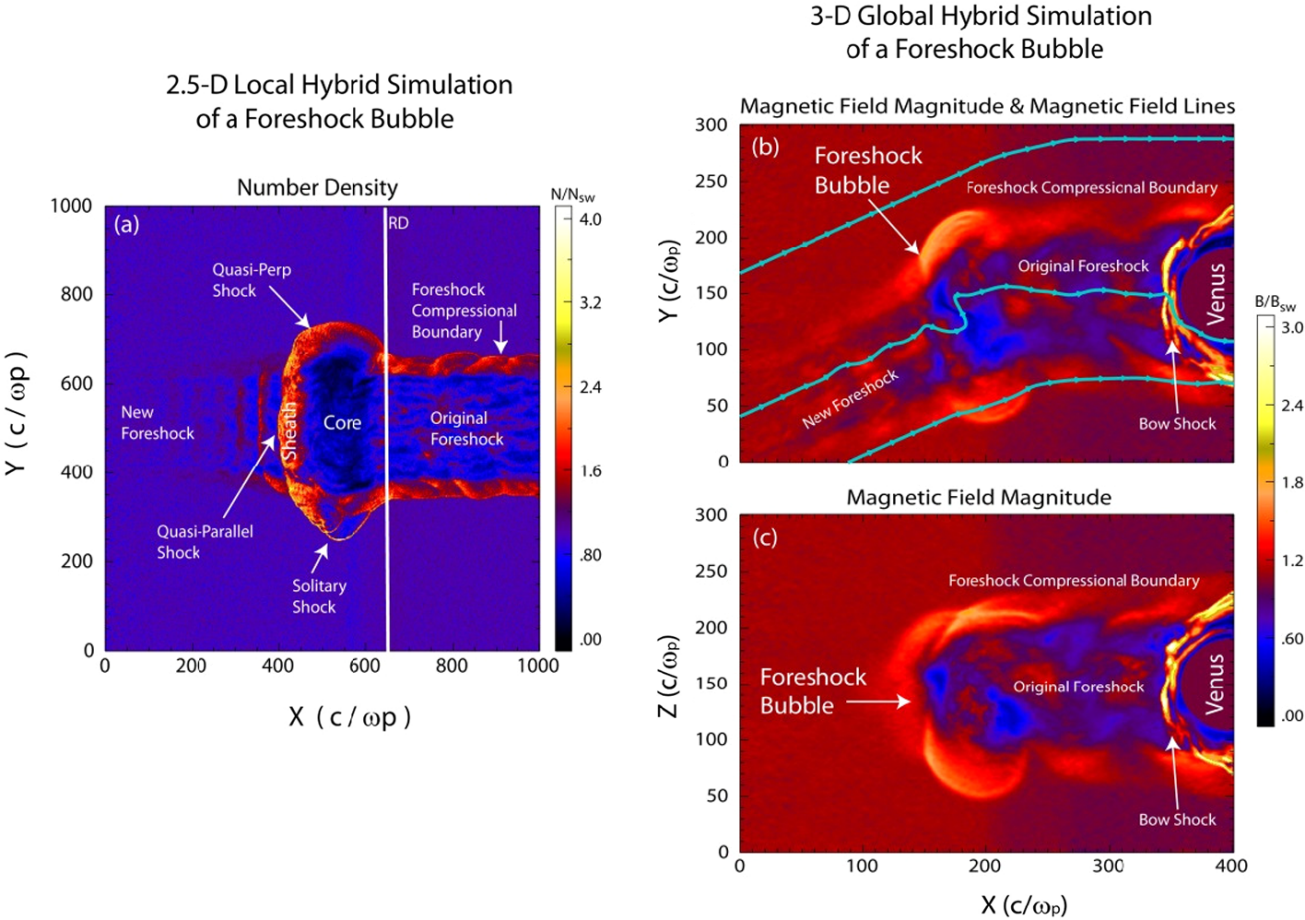
Structure of foreshock bubbles in 2-D and 3-D hybrid simulations. (**a**) density (normalized to solar wind value) from a local 2.5-D hybrid simulation (**b**-**c**) magnetic field strength from a 3-D global hybrid simulation of solar wind interaction with Venus (from Omidi et al. 2010, 2020a)

Panel (a) of Fig. 21 illustrates the structure of a foreshock bubble formed in a local 2.5-D (2-D in space, 3-D currents, fields) hybrid simulation of the interaction between a solar wind RD and a beam of ions. It shows the density (normalized to solar wind value) in a run with the solar wind moving from left to right, interacting with a finite width (in the Y direction) backstreaming ion beam injected from the right hand boundary moving to the left. A foreshock bubble forms on the sunward (upstream) side of the RD embedded in the solar wind. The original foreshock with the associated Foreshock Compressional Boundary lies downstream (to the right) of the RD. The shock wave associated with the foreshock bubble consists of quasi-perpendicular and quasi-parallel geometries and results in the formation of a new foreshock upstream of the FB. It was shown by Omidi et al. (2010) that the width of the FB scales with the width of the ion foreshock and at Earth would correspond to ∼10 $R_{\mathrm{{E}}}$ or more. Foreshock bubbles are carried anti-sunward by the solar wind and depending on the IMF cone angle collide with different parts of the bow shock and magnetosphere.

The discovery of foreshock bubbles was greatly facilitated by simultaneous THEMIS mission observations from far upstream to inside the magnetosphere, making it possible to study the formation, growth and interaction of FBs with the bow shock and the magnetosphere. The use of ground-based magnetometers has allowed us to observe the global impacts of FBs on the ground. The first direct evidence for the existence of foreshock bubbles was presented by Turner et al. (2013). Initial observations of FBs confirmed the model prediction that they form as a result of the interaction between solar wind RDs and backstreaming ions. However, subsequent observations of FBs have shown that tangential discontinuities in the solar wind can also generate foreshock bubbles (Liu et al. 2015, 2016b). Liu et al. (2015) proposed that when foreshock ion gyroradii are larger than the TD thickness, they can gyrate across a TD and form an FB. Recent global hybrid simulation by Wang et al. (2021) generated a TD-driven FB and confirmed this hypothesis. Both global hybrid simulations and spacecraft observations have established foreshock bubbles as highly efficient accelerators of particles. The FB shock can reflect and accelerate solar wind particles through shock drift acceleration forming a new foreshock (Liu et al. 2016a). As the FB shock convects toward the bow shock, electrons and ions can bounce between the bow shock and the FB shock (at the magnetic gradient of the anti-sunward edge) and gain energy through Fermi acceleration (Liu et al. 2017d, 2018; Omidi et al. 2021). Because of the super-fast-magnetosonic expansion, magnetic flux is transported outward very rapidly towards the FB shock. As a result, electrons are observed to be further energized by a factor of 10 up to 100s of keV through betatron acceleration (Liu et al. 2019a).

As discussed in Omidi et al. (2010), upon encountering the bow shock, the lower pressure in the FB core results in the outward motion of the bow shock and sunward flows in the dayside magnetosheath and the outward expansion of the magnetopause and the dayside magnetosphere, which can result in magnetospheric ULF waves as shown in Hartinger et al. (2013). The interaction also results in the injection of high energy particles into the magnetosheath and magnetosphere. Subsequently, the portion of the bow shock colliding with the FB is dissipated and replaced with the FB shock. This results in the return of the anti-sunward flows in the magnetosheath and compression of the magnetopause. Archer et al. (2015) used multi-spacecraft and ground-based observations to demonstrate that FBs have a global impact on the magnetosphere-ionosphere system. Specifically, they showed that measurements in the magnetosheath, magnetopause, the inner magnetosphere and on the ground all show signatures associated with the passage of the FB. They also established that among numerous other foreshock phenomena such as HFAs, foreshock bubbles have the biggest impact on the magnetosphere. Global hybrid simulation by Wang et al. (2020b) shows that FBs can propagate from dayside to the midtail and continuously disturb the local bow shock, magnetosheath, and magnetopause. Much more remains to be understood about the impacts of FBs on the magnetosphere.

In a recent study by Omidi et al. (2020a), 3-D global hybrid simulations and data from Venus Express (VEX) spacecraft was used to investigate the formation of foreshock bubbles at the planet Venus. Panels (b and c) in Fig. 21 show the magnetic field strength from a 3-D global hybrid simulation of solar wind interaction with Venus where an FB has formed upstream of the bow shock due to the presence of an RD in the solar wind. As expected, the size of the FB is similar to the width of the foreshock at Venus which is slightly larger than the diameter of the planet. It is evident from Fig. 21 that the 2- and 3-D structures of FBs are quite similar including the formation of a new foreshock upstream of the bubble in agreement with the observations by Liu et al. (2016a). Examination of VEX data shows ample evidence for the existence of FBs at Venus. Based on the results presented in Omidi et al. (2020a), we expect FBs to have a significant impact on the Venusian ionosphere and loss of planetary ions.

#### Foreshock Cavities

Foreshock cavities are regions with enhanced suprathermal ion fluxes but depressed densities and magnetic field strengths that are bounded by regions without enhanced suprathermal ion fluxes but enhanced densities and magnetic field strengths (Sibeck et al. 2002). They have been detected at Earth, Venus, and Mars (Collinson et al. 2020). At Earth, they typically have durations ranging from one to several minutes, but some can last longer than one hour (Sibeck et al. 2001; Billingham et al. 2008). Event amplitudes decay with distance from the bow shock, and their simultaneous appearances can be very different upstream from the dawn and dusk bow shock (Sibeck et al. 2004). Foreshock cavities tend to occur during intervals of enhanced solar wind velocity (Sibeck et al. 2001; Billingham et al. 2008), which may be equivalent to a statement that they tend to occur during intervals of radial IMF, because the IMF tends to be more radial during intervals of enhanced solar wind velocity.

The cavities are well-explained by numerical simulations for wave particle interactions occurring in spatially limited regions. The heating increases ion thermal pressures and causes regions connected to the bow shock to expand outward at the expense of surrounding regions where no heating is occurring. This results in field-aligned diamagnetic cavities with depressed magnetic field strengths bounded by enhanced densities and magnetic field strengths, both in simplified (Thomas and Brecht 1988) and more realistic geometries (Lin 2003).

Two explanations have been provided for foreshock cavities. In some global hybrid code simulations, the steady-state foreshock that occurs during intervals of nearly radial IMF orientation is bounded by regions of enhanced density and magnetic field strength, namely the foreshock compressional boundaries. The strengths of these boundaries increase with increasing solar wind Mach number, or equivalently velocity (Omidi et al. 2009). These boundaries might sway back and forth across a nearby spacecraft, resulting in transient entries and exits from the foreshock bounded by these enhancements (Sibeck et al. 2008). Alternatively, as argued by Billingham et al. (2008), transient entries into foreshock cavities may simply result from the passage of antisunward-moving slabs of magnetic field lines connected to the bow shock embedded within regions that are not connected to the bow shock.

Simulations and observations indicate that the cavities are readily transmitted across the bow shock and into the magnetosheath (Sibeck et al. 2021), where they can again be recognized on the basis of correlated density and magnetic field strength enhancements bounding heated plasmas accompanied by depressed and fluctuating magnetic field strengths and densities. However, there is one big difference between the boundaries of cavities in the magnetosheath and upstream from the bow shock. The boundaries of cavities in the magnetosheath exhibit flow velocity enhancements or jets relative to the surrounding plasma, where as the boundaries of cavities upstream in the foreshock do not (Kajdič et al. 2021; Sibeck et al. 2021). Foreshock cavities can drive strong pressure variations in the sheath and along the magnetopause, resulting in strong magnetopause motion (Turner et al. 2011).

#### Foreshock Cavitons

**Fig. 22:**
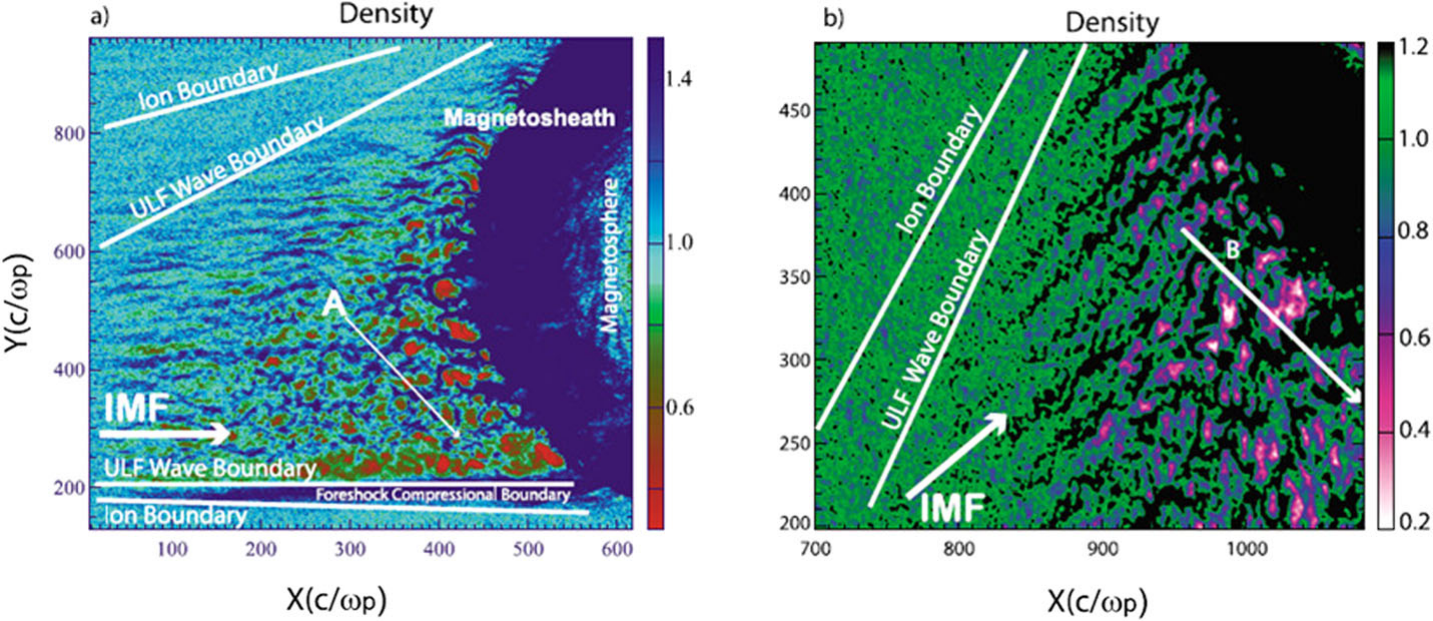
Hybrid simulation results of the ion foreshock and the region filled with waves and cavitons under (**a**) radial and (**b**) oblique IMF orientations. Cavitons appear as red spots in panel (**a**) and pink and white spots in panel (**b**). (Blanco-Cano et al. 2011)

Foreshock cavitons are transient structures with low magnetic field strength and plasma density core bounded by a rim of enhanced magnetic field strength and plasma density, which can develop self-consistently in the foreshock region (Lin 2003; Blanco-Cano et al. 2009). Lin (2003) predicted this type of crater-like foreshock structures using a 2-D hybrid simulation and called them “diamagnetic cavities”. There is no plasma heating or flow deflection inside cavitons. Cavitons are not associated with IMF discontinuities and often surrounded by a train of ULF waves (Blanco-Cano et al. 2009), which are different from isolated cavities with no ULF waves nearby (Schwartz et al. 2006). Lin (2003) suggested that the formation of foreshock cavitons is related to the interaction between the solar wind ions and the backstreaming ion beams. Hybrid simulations (Blanco-Cano et al. 2009, 2011) suggested that the nonlinear interaction of two types of ULF waves generated by backstreaming ions via ion beam instabilities, the parallel-propagating weakly compressive waves and oblique-propagating linearly polarized fast magnetosonic waves, contributes to the generation of foreshock cavitons. Foreshock cavitons can exist in the foreshock region under different IMF orientations and their major features are independent of the IMF orientation. Foreshock cavitons develop into elongated structures along the magnetic field lines under radial IMF conditions, while they are field-aligned filaments or crater-like structures under oblique IMF conditions (Lin 2003). Figure 22 indicates the existence of foreshock cavitons for different IMF geometries in hybrid simulations. Figure 6 shows an example of a foreshock caviton observed by Cluster on 27 January 2003 (Blanco-Cano et al. 2011). Kajdič et al. (2017) found that foreshock cavitons might exist inside the traveling foreshocks bounded by two RDs. Their statistical results showed that the changes in magnetic field magnitude and plasma density are mostly correlated in foreshock cavitons.

The first statistical study based on Cluster observations (Kajdič et al. 2013) showed that distinct cavitons appear under a wide range of solar wind and IMF conditions. The average duration of cavitons is 65 s and their average size is 4.6 $R_{\mathrm{{E}}}$. The depletions in the magnetic field strength and density are greater near the bow shock (Tarvus et al. 2021). Kajdič et al. (2011) analyzed two foreshock cavitons observed by Cluster and found that these two cavitons are highly structured. They also found that these two cavitons were propagating sunward at a speed of 188 km/s and 120 km/s in the solar wind frame. Wang et al. (2019b) studied the propagation properties of twelve foreshock cavitons observed by four Cluster satellites using multi-spacecraft analysis methods, including the timing method, MDD method (Shi et al. 2005) and STD method (Shi et al. 2006, 2019). Their results showed that all cavitons propagate towards the Earth in the spacecraft frame and eleven structures move towards the sun in the solar wind frame. They also found that cavitons with larger sizes move faster in the solar wind frame. The propagation speed of cavitons in the solar wind frame is less than that of SHFAs (Tarvus et al. 2021).

Omidi et al. (2013a) studied the result of foreshock cavitons interacting with terrestrial bow shock using 2.5-D electromagnetic hybrid simulations. Their simulations showed that a new type of transient structures can be generated from this interaction process, i.e., SHFAs, which were observed by THEMIS (Zhang et al. 2013). Ion trapping by foreshock cavitons and Fermi acceleration from the back and forth motion of ions between the cavitons and the bow shock may play an important role in the particle acceleration (Omidi et al. 2013a). Tarvus et al. (2021) used the global hybrid-Vlasov simulation model Vlasiator to investigate caviton-to-SHFA evolution and their properties. They found that a third of the cavitons evolve into SHFAs and SHFAs can form independently near the bow shock.

#### Foreshock Compressional Boundaries

**Fig. 23:**
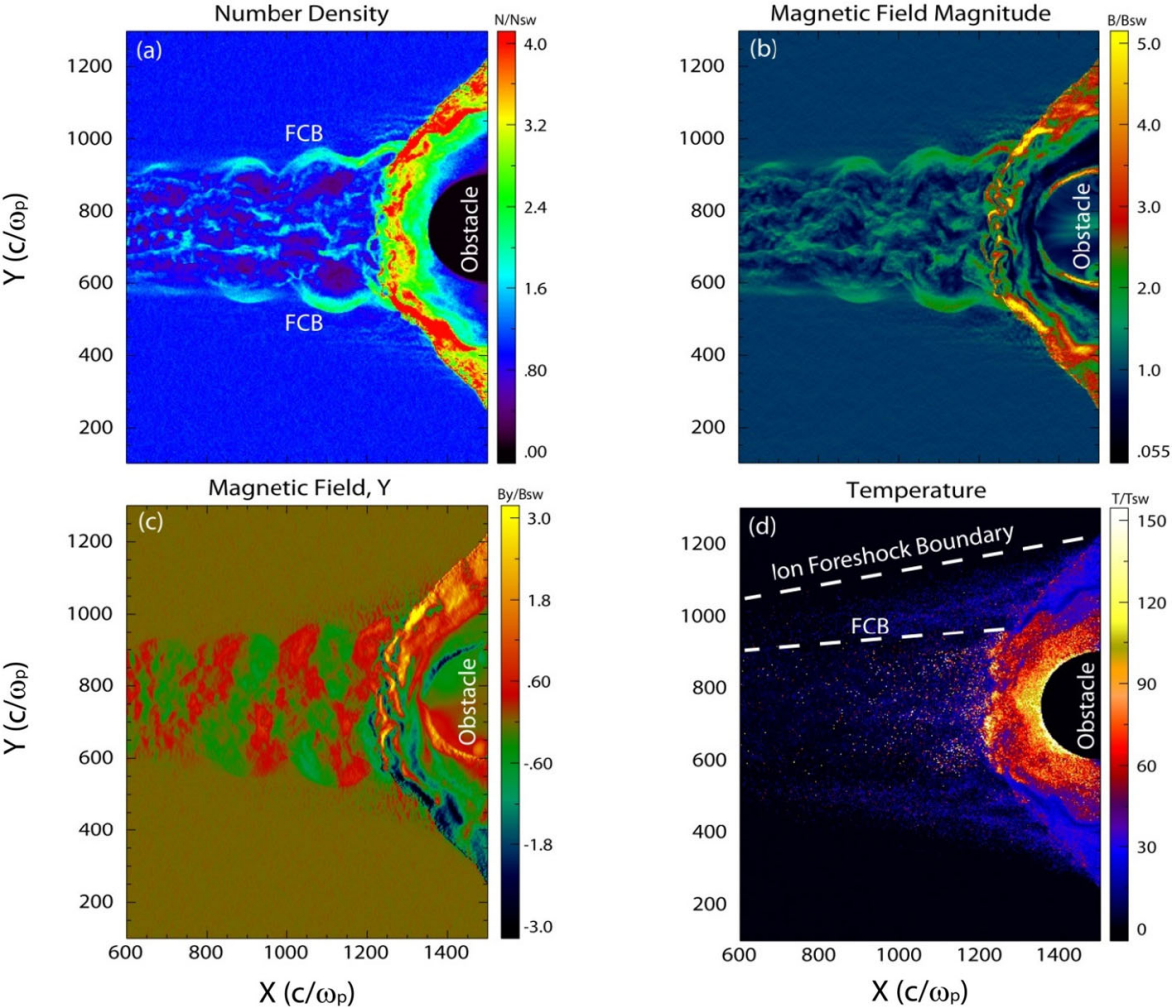
Results of a global hybrid run during radial IMF. The configuration is similar to that in Fig. 2 of Omidi et al. (2020b). (**a**) density (**b**) magnetic field strength (**c**) $Y$ component of the magnetic field (**d**) ion temperature (Credit: Nick Omidi)

The ion foreshock boundary separates the pristine solar wind from a region, upstream of the quasi-parallel bow shock, containing beams of backstreaming ions reflected or leaked from the shock. Additional boundaries have been defined to mark the region containing ion beams and ULF waves (ULF wave boundary) and the region containing compressional ULF waves named the “compressional ULF foreshock boundary” (e.g., Greenstadt and Baum 1986). More recently, Sibeck et al. (2008) identified a new foreshock boundary consisting of a fast magnetosonic pulse in global hybrid simulations of solar wind interaction with the magnetosphere. This was followed by a more detailed investigation of this boundary named the Foreshock Compressional Boundary (FCB) where the effects of solar wind Mach number and the IMF cone angle were examined and an example of this boundary observed by spacecraft was provided (Omidi et al. 2009). Figure 23 shows an example of the FCB formed in a global hybrid simulation with solar wind Alfvén Mach number of $M_{\mathrm{{A}}} =$ 15 and cone angle of 0^∘^ (radial IMF). Panels (a) to (d) in this figure show the density, total magnetic field strength, the $Y$ component of magnetic field, and ion temperature, respectively. The FCB is associated with increased density and magnetic field strength as expected for a fast magnetosonic pulse. Based on the radial nature of the IMF and symmetry around it, the FCB is a cylindrical boundary in 3-D (see 3-D example in Omidi et al. 2017b).

Formation of the FCB is directly tied to the generation of foreshock cavitons and the associated plasma and field excavation that results in the lateral expansion of the foreshock plasma and the drop in average density and magnetic field levels. The interaction between this laterally expanding plasma and the solar wind results in plasma and field pile up forming the FCB. The formation and the presence of cavitons in Fig. 23 can be noted by the associated drops in density and magnetic field in their cores. Given the connection between the FCB and cavitons, the former can be viewed as the boundary between the region in the foreshock where cavitons are formed and the region where they are not. Panel (d) in Fig. 23 demonstrates this point in a clear fashion where the white dashed lines show the locations of the ion foreshock boundary and the FCB. The fact that the two boundaries do not coincide indicates that typically the foreshock can extend beyond the FCB. Examination of panel (c) in Fig. 23 shows that the waves generated between the FCB and the ion foreshock boundary have smaller wavelength (∼10 $c/\omega _{\mathrm{p}}$) as compared to the ones generated in the region where cavitons are formed (∼200 $c/\omega _{\mathrm{p}}$). However, it is also possible that at times the FCB and the ion foreshock boundary may coincide such that it separates the foreshock plasma from pristine solar wind. This result is consistent with the study by Rojas-Castillo et al. (2013) who used spacecraft data to investigate the properties of FCBs. They found that at times the FCB is between the pristine solar wind and foreshock plasma while at other times it falls inside the ion foreshock boundary.

Omidi et al. (2009) examined the impacts of solar wind Mach number on the FCB and showed that the amplitude of the magnetosonic pulse associated with the FCB increases as solar wind Mach number becomes larger. This is tied to the fact that as the solar wind Mach number increases, so do the size and strength of the foreshock cavitons (Omidi et al. 2013a), which enhances the expansion of the foreshock and the strength of the FCB. For sufficiently high Mach numbers, the steepening of the magnetosonic pulse results in FCB being associated with a shock wave. Spacecraft observations confirm this dependence of the FCB strength on solar wind Mach number (Rojas-Castillo et al. 2013). It was also demonstrated by Omidi et al. (2009) that as the cone angle increases to ${\geq} 20^{ \circ }$, the FCB does not form symmetrically around the foreshock and appears on the side that falls deep within the foreshock where cavitons are formed.

As was demonstrated by Omidi et al. (2013a), the FCB is a highly dynamic boundary in that as the lateral expansion of the foreshock due to caviton formation continues the FCB can proceed to move out laterally. In addition, the FCB is continually carried by the solar wind towards the bow shock and into the magnetosheath resulting in FCB footprint in this plasma. For example, note the regions of enhanced density and magnetic field in the sheath that connect to the FCB demonstrating the amplification of the FCB pulse as it enters the magnetosheath. By introducing solar wind discontinuities in the simulations, it was shown by Omidi et al. (2013a) that FCBs are also observed in connection with foreshock cavities. For example, connection of a bundle of IMF lines to the bow shock can result in the formation of traveling foreshocks with FCBs at their edges. Kajdič et al. (2017) used spacecraft data to confirm the formation of traveling foreshocks with FCBs at the edge.

#### ULF Wave Growth/Shocklets/SLAMS in the Dayside and Tail Foreshock

As mentioned in Sect. 2.1, the foreshock region is characterized by the presence of backstreaming particles. The features of the ion interactions with the bow shock strongly vary between Q_⊥_ and Q$_{ \parallel }$ shock regions. For perpendicular shock, the maximum upstream excursion of reflected ions is restricted to the gyromotion that they suffer at the shock front under the effect of the magnetic field; they cannot escape upstream. As the angle between the shock normal and the IMF departs from 90^∘^, this gyromotion is distorted and some reflected ions move upstream and succeed to cover a larger upstream excursion. For Q$_{ \parallel }$ shock, the impact of the magnetic field is strongly reduced in the sense that most reflected ions succeed to escape very far upstream along the magnetic field and are reinjected into the solar wind. As a consequence, a large ion density gradient takes place upstream of the front. An extended ion foreshock forms ahead of the shock front and is connected magnetically to it.

Moreover, earliest works on the upstream region of quasiparallel bow shock have noted that the magnetic field profile often consists of large amplitude “magnetic fluctuations” also named “magnetic pulsations” (Greenstadt et al. 1968a,b, 1977) and what appeared to be nonlinearly steepened ULF waves (e.g., Fairfield 1969; Russell et al. 1971). Further works have identified these nonlinearly steepened ULF waves as magnetosonic whistler modes which have been named “shocklets” since these look like small-shock structures (Hoppe et al. 1981). These shocklets are magnetosonic and some have whistler waves packet attached. Then, the ion foreshock region appears to be also characterized by a large variety of ULF waves (with frequencies much less than the ion gyrofrequency) in addition to various suprathermal ion distributions. These ULF waves also include so called “30 second waves”, “10 seconds waves”, “3 second waves” and “1 Hertz waves” (Burgess 1997; Eastwood et al. 2005). The features of the different magnetic structures and associated ion populations may be found in previous reviews (Greenstadt et al. 1995; Burgess et al. 2005; Wilson 2016).

**Fig. 24:**
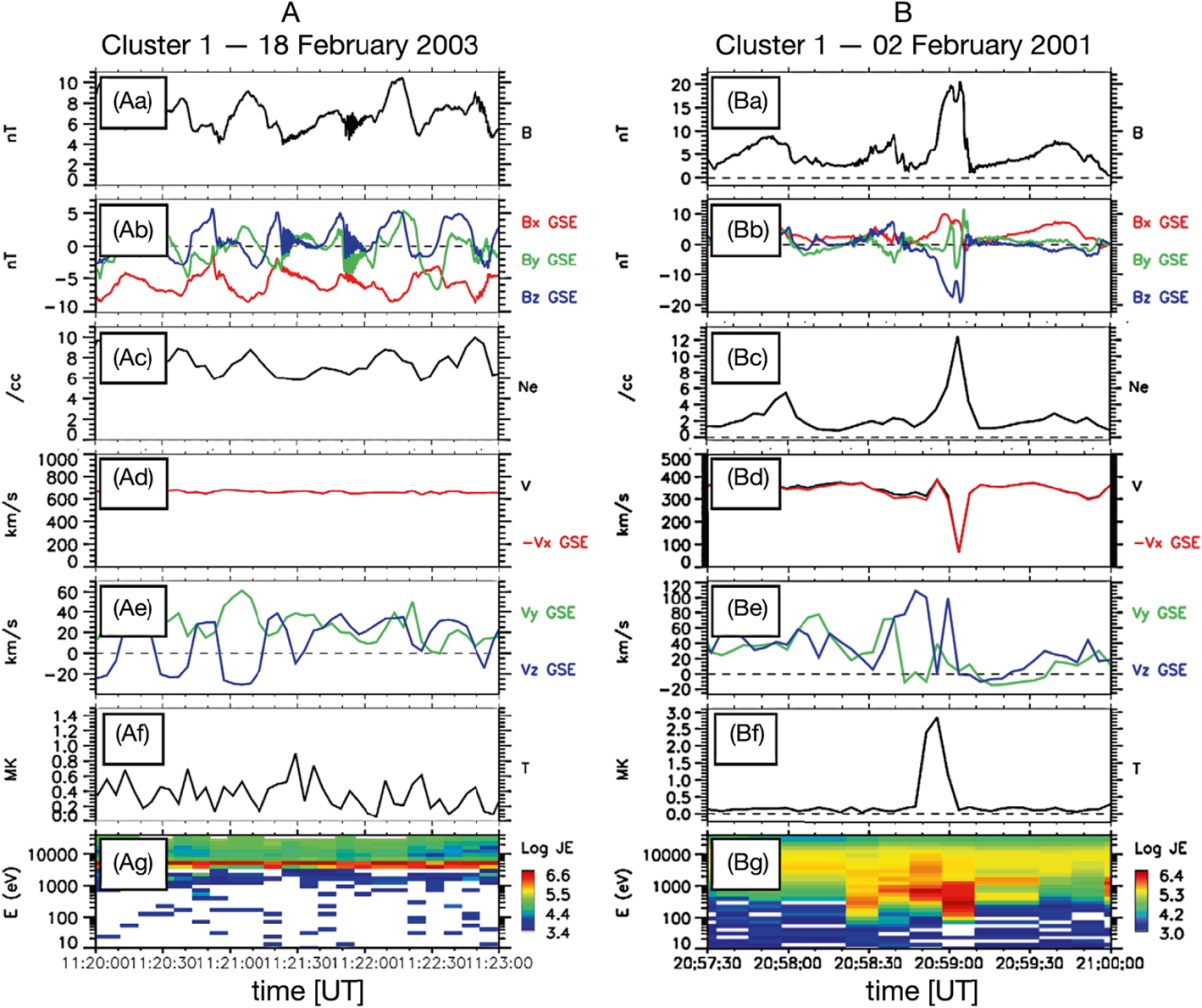
(**A**) Example of “shocklets” observed by Cluster 1 on February 18 2003; (**B**) Example of a “SLAMS” on February 2 2001. In both examples, panels (from top to bottom) show the amplitude of the magnetic field, its components, the plasma density, the bulk velocity (amplitude and its components), the total temperature T (including solar wind and suprathermals), and the proton energy spectrum. (From Plaschke et al. 2018a, Fig. 19)

Later on, magnetic structures with features “apparently” different from “shocklets” have been identified and named SLAMS (Short Large Amplitude Magnetic Structures) (Schwartz et al. 1992). Both structures are illustrated in Fig. 24. However, the over-use of these different labels (sometimes inappropriate) in the literature has led to a certain confusion and raised the following questions: are “shocklets” and “SLAMS” similar or quite different entities? are these issued from similar or different mechanisms? Answering these questions have been clarified with the help of simulations and comparison between experimental and simulations results, and are addressed below.

Let us remind that backstreaming ions can propagate far within the upstream foreshock region and do have quite enough time to interact with the incoming solar wind ions and excite some instabilities (ULF waves). Far from the front (where the ion density gradient is still relatively weak), these ULF waves propagate almost parallel to the magnetic field. One accepted scenario is that these ULF waves suffer progressively a refraction when approaching the shock front where the density gradient becomes very large and their direction changes into parallel to the density gradient i.e. along the averaged shock normal direction (Scholer 1993). During the linear/nonlinear stage, these ULF waves suffer a progressive steepening as these are convected back to the shock front by the solar wind through the ion density gradient, and form the so-called SLAMS (Giacalone et al. 1993, 1994b). Many works based on hybrid simulations (Scholer et al. 1993; Scholer 1993; Dubouloz and Scholer 1993, 1995) and PIC simulations (Scholer et al. 2003; Tsubouchi and Lembège 2004) have confirmed such a scenario. More recently, this scenario has also been confirmed based on both measurement of the MMS mission and PIC simulations by Chen et al. (2021) who brought more precise information on the nonlinear ULF waves development into SLAMS. Three effects contribute: (i) gyro resonance between solar wind ions and right-hand circularly polarized electromagnetic waves results in magnetic field amplifications, (ii) gyro-trapping by the growing magnetic field builds up the plasma density that enhances the current and magnetic field, and (iii) the magnetic field is amplified at discrete isolated locations at the |B| maximal of the wave envelope where the initial density enhancement and longitudinal electric field develop. Typical spatial/time scales of ULF waves/SLAMS/whistler precursor measured during this scenario may be found in Table 2.

**Table 2:** Spatial and time scales of ULF waves, SLAMS and whistler precursor measured in 1D PIC simulation (issued from Tsubouchi and Lembège 2004, Table 2)

	Symmetric SLAMS	Asymmetric SLAMS	Spiky SLAMS
SLAMS			
Spatial extent	$14c/\omega _{pi}$	$14.2c/\omega _{pi}$	$13.2c/\omega _{pi}$
Leading edge:			
Ramp width	$6c/\omega _{pi}$	$2.47c/\omega _{pi}$	NA
Velocity	$4.47v_{A}$	$4.8v_{A}$	$5.25v_{A}$
*δ*B/B	2.66	4.4	3.7
Spiky $E_{lx}$ extent	NA	NA	$0.56c/\omega _{pi}$
Tailing edge:			
Ramp width	$3.54c/\omega _{pi}$	$5.11c/\omega _{pi}$	$6c/\omega _{pi}$
*δ*B/B	2.3	1.6	1.3
“ULF wave growth” phase			
ULF wave			
Wave length *λ*/2	$35c/\omega _{pi}$		
Velocity	$3v_{A}$		
Frequency	$0.5\omega _{ci}$		
“Spiky-SLAMS” phase			
Whistler precursor			
	From new	From the old	
	ramp “N”	ramp “O”	
Wave length *λ*/2	$2.5c/\omega _{pi}$	$2.7c/\omega _{pi}$	
Velocity	$5.6v_{A}$	$3v_{A}$	
Frequency	$13.9\omega _{ci}$	$6.8\omega _{ci}$	

In order to distinguish more clearly shocklet and SLAMS, we need to emphasize these respective features. In short, SLAMS are identified by the following magnetic field signatures: (i) the magnetic field magnitude is enhanced over the undisturbed B field by at least $\delta $B/B$>2-2.5$, with a duration of the order of 10 s in the spacecraft frame; (ii) they have a well defined smooth “near monolithic” profile; (iii) their spatial scales decrease with increasing amplitude (Schwartz et al. 1992; Mann et al. 1994), (iv) these show decreasing convection velocity (in the spacecraft frame) with decreasing distance to the bow shock (Mann et al. 1994); (v) scales sizes parallel to the shock normal and tangential to the shock surface are respectively larger than 1000 km and around 1300 km as observed experimentally by Lucek et al. (2004a, 2008) and retrieved in 2D simulations (respectively >1000 km and 3000 km) by Dubouloz and Scholer (1995); (vi) their gradient scale lengths are around 100 km (Lucek et al. 2004a, 2008). They occur in regions of ULF wave activity (isolated SLAMS) and within regions of stronger magnetic field pulsations associated with decelerated and heated plasma (embedded SLAMS), as well observed in experimental data (Schwartz et al. 1992; Mann et al. 1994; Lucek et al. 2002, 2004a); embedded SLAMS have larger amplitude than isolated SLAMS (Schwartz et al. 1992). They are found to propagate sunward in the plasma frame but are convected anti-sunward by the solar wind. They exhibit mixed polarization, biased towards right-hand polarized signatures (in the spacecraft frame) but often with a remnant of a left-handed (in the plasma rest frame), high frequency whistler wave on the leading edge similar to those at the lower amplitude (Schwartz et al. 1992). One striking feature is that local measurements show that the upstream (steepened) edge of the SLAMS is behaving as a local Q_⊥_ shock (Mann et al. 1994), which has been confirmed by 1D PIC simulation (Tsubouchi and Lembège 2004). Later on, data issued from Cluster multi-spacecraft mission have confirmed the observations of SLAMS in particular at different stages of their formation (Lucek et al. 2002, 2004a; Behlke et al. 2003; Burgess et al. 2005). Note that for large amplitude SLAMS, the amount of reflected solar wind ions reaches values around 30%. Thus, SLAMS represent effective ion reflectors of both solar wind ions and shock-reflected ions and are expected to play an important role in dissipation upstream of and at the Q$_{ \parallel }$ bow shock (Behlke et al. 2003).

Shocklets features differ from SLAMS in several aspects: (i) in terms of their magnetic compression ratio $\delta $B/B $<2$ (e.g., Schwartz et al. 1992), whereas ULF waves typically have $\delta $B/B=1; (ii) scale sizes of shocklets are similar to those of “30 second waves” i.e. up to a few $R_{\mathrm{{E}}}$ (Hoppe et al. 1981; Le and Russell 1994; Lucek et al. 2002); (iii) discrete wave packets associated with shocklets are characterized by wavelengths of 30 km to 2100 km and their propagation angles versus the magnetic field are around $20^{\circ }-30^{\circ }$ (Russell et al. 1971; Hoppe et al. 1981).

For a further comparison, more recent works have focussed on the features of shocklets, SLAMS, associated wave packets and dispersive precursor observed experimentally which are summarized in Table 3. But, the fact that shocklets and SLAMS do not have the same scales/amplitude/time duration does not mean that these are necessarily issued from different origins, in contrast with Wilson et al. (2013) and Wilson (2016) statements. Indeed, despite of their differences illustrated in Table 3 (and which may be found also in references of Wilson (2016)), these present strong similarities in their nature: (i) both are magnetosonic with the density inside them in phase with the magnetic field magnitude; (ii) both propagate sunward in the plasma frame of reference but are carried earthward by the solar wind, as their phase speed is much lower than the solar wind speed; (iii) both can exhibit left-hand polarizations (in the plasma rest frame); (iv) both can dispersively radiate an upstream whistler precursor (directed sunward in the plasma rest frame); (v) both modes contain a low frequency, large-scale compressional pulse (i.e. shocklet and SLAMS) and a higher frequency, smaller wave packet labelled “discrete wave packets” (Russell et al. 1971) which are identified now as a whistler precursor; it is important to remind that not all SLAMS or shocklets have an associated whistler precursor (Hoppe et al. 1981; Schwartz et al. 1992). All these similar features strongly suggest that both magnetic structures may be issued from a same origin as proposed in many previous works.

**Table 3:** Summary of the characteristics of the main different magnetic structures measured experimentally in the ion foreshock region (extracted from Wilson 2016, Table 1)

Frequency (f, *ω*, or *γ*)		Polarization		Amplitude		Angle		Scale or Speed
SCF	PRF	SCF	PRF	*δ*B [nT]	*δ*B/B	$\theta _{kB}$	$\theta _{kn}$	*λ*, *k*, or $V_{ph}$
shocklet (low frequency, magnetosonic, diffuse ions)								
*f* ∼ 10 − 100mHz	$\omega \sim 0.01-0.8 \Omega _{cp}$	L, RH	L, LH, RH	≤5	≤1.5	∼14^∘^ − 40^∘^	∼30^∘^ − 35^∘^	*λ* ∼ 700 − 9000 km
SLAMS (low frequency, magnetosonic, gradients in diffuse ion density)								
*f* ∼ 50 − 250mHz	$\gamma \sim \Omega _{cp}$	LH, RH	N/A	∼10 − 60	≥2 − 6	∼2^∘^ − 75^∘^ (most >30^∘^)	∼1^∘^ − 60^∘^ (most <30^∘^)	*λ* ∼ 1000 km $V_{ph}/V_{A}\sim 3.58\pm 1.04$
“Discrete Wave Packet” (RH, shocklet whistler precursor, dispersive radiation)								
*f* ∼ 0.1 − 3.0 Hz	$\omega \sim 0.2-38 \Omega _{cp}$	LH, RH	RH	≤5	≤1	∼10^∘^ − 60^∘^ (most ≤45^∘^)	∼20^∘^ − 75^∘^	*λ* ∼ 30 − 2000 km $V_{ph} \sim 70-100 \mbox{ km}/s$
whistler precursor (RH, shock whistler precursor, dispersive radiation and/or shock reflected ion instabilities)								
*f* ∼ 0.007 − 10.0 Hz	*f* ∼ 0.5 − 11 Hz $\omega \sim 4-27 \Omega _{cp}$	LH, RH	RH	≥1 − 30	∼0.01 − 2.1	∼10^∘^ − 88^∘^	∼3^∘^ − 90^∘^	*λ* ∼ 15 − 1600 km (most <300 km)

In order to support this idea, a more precise approach of the scenario looks necessary as summarized as follows. Let us remind that SLAMS have smaller scale sizes than shocklets and ULF waves (Lucek et al. 2002), and the amplitude of SLAMS is higher than those of shocklets and ULF waves. When an original upstream ULF wave is convected back to a region of increasing diffuse ion density upstream of the shock, the wave steepens, becoming a pulsation-like wave packet (as for so called “shocklets”). Then, a shrinking takes place so that their spatial scale decreases while their amplitude increases. But, one additional condition is necessary for the wave to grow and reach the “ultimate” stage of a pulsation-like structure (as for so called “SLAMS”): the characteristic scale length of the ion density gradient has to be of the same order as the wavelength of the original ULF wave as mentioned originally in hybrid simulations (Scholer 1993) and later on confirmed in PIC simulations (Tsubouchi and Lembège 2004). Let us complete with one additional point: this wave shrinking and steepening (at the leading edge) may continue until nonlinear effects reach a limit. This limit takes place as nonlinear effects are balanced by dispersive effects (emission of a whistler precursor) and/or dissipation effects (local ion reflection as in Tsubouchi and Lembège (2004)), and/or ion trapping within the emitted whistler wave train (as in Scholer et al. 2003); a SLAMS forms at this “ultimate” stage. This is coherent with the fact that the structure/dynamics of the leading edge of the SLAMS behaves as a Q_⊥_ shock front (Mann et al. 1994; Tsubouchi and Lembège 2004). Scholer et al. (2003) have shown that at some time of the simulation, the SLAMS consists of two regions with different distributions: in the upstream part, the distribution is decelerated and adiabatically heated, and in the whistler wave train the distribution has a high energy tail. In the extreme case where dispersive effects are very important (emission of a strong whistler precursor), these can refrain or even stop the local steepening, and the “ultimate” stage of SLAMS formation could be hardly or could not be reached. The impact of this competition between these different effects may require a deeper investigation.

In addition to the conditions mentioned above, this scenario can be refined with the following reminder: the upstream region of a Q$_{\parallel }$ shock is strongly nonstationary (in time) and inhomogeneous (in space), which will impact the local plasma conditions of the wave refraction/shrinking. The relative occurrence of shocklet and SLAMS in time and space may also result from an “interplay” between the local strength of the convection velocity and the amplitude of the local ion density gradient through which the plasma is convected to the shock front. If the local ion density gradient is finite but relatively weak, the growth of shocklets from the ULF can take place but its steepening may not be strong enough during the convection to reach the ultimate stage and to be categorized into a SLAMS: then, signature of shocklets may be dominant. In contrast, if the ion density gradient is high, the steepening of the growing shocklet may be strong enough to reach the ultimate stage of a SLAMS which will be the final dominant signature. Moreover, let us precise that how quick shocklets or SLAMS are formed may depend on the convection velocity of the plasma within the ion density gradient. If the local convection is high, the signature of an “intermediate” shocklet may not be clearly evidenced during the rapid growth/steepening of the SLAMS. The dependence of this scenario versus local varying plasma conditions may account for a certain range of spatial scales/amplitude/duration of the magnetic structures as illustrated in Table 3 for shocklets and SLAMS. In summary, each magnetic structure results from an “interplay” between different effects: wave refraction/shrinking conditions, local steepening versus dispersion/dissipation effects, and impact of local varying plasma conditions (nonstationary upstream region). This interplay will give birth—both in time and space—to the possible emergence of shocklets or SLAMS, keeping in mind that large amplitude SLAMS correspond to the “ultimate” stage of the scenario. A combination of global and local 2D/3D simulations could be helpful for refining these different stages which contribute to the “patchwork” of magnetic signatures initially proposed by Schwartz and Burgess (1991).

This scenario supports well old and recent experimental and simulation results but may not be unique. Let us remind a “variante” to this scenario proposed by Scholer and Burgess (1992). The main difference is that SLAMS can be formed not from the steepening of nonlinear ULF waves originally issued from the interaction of incoming solar wind and reflected gyrating ion beam. Instead, by using 1D hybrid simulations, Scholer and Burgess (1992) let a magnetic field perturbation (convected with the solar wind) interact with a finite length beam (without a shock). The beam ions are deflected by the magnetic field perturbation. Depending on the angle $\Theta _{bi}$ between the magnetic field (in the perturbation) and the direction of the reflected ion beam, the local beam density increases, which in turn increases locally the magnetic field. This then may also lead to large-amplitude magnetic field structures. The idea is that during small $\Theta _{bi}$ the beam of more or less specularly reflected ions can travel far upstream where it encounters a magnetic field perturbation with a locally large $\Theta _{bi}$. There, the feedback loop occurs and the SLAMS rises up. But, at that time, the authors did not focus on possible raise/identification of “shocklet” before the “SLAMS” stage is finally reached. This point needs to be clarified. In summary, the main difference is that SLAMS can build up from (i) either the steepening of ULF waves originally self-generated by the shock front itself (common scenario), (ii) either the steepening of an external magnetic perturbation carried by the solar wind. This last variante could be analyzed in more detail when applied to some transient structures reviewed in the present document (see Sect. 2.3.10).

##### ULF Waves/Shocklet/SLAMS Versus Self-Reformation/Nonstationarity of the Shock Front:

**Fig. 25:**
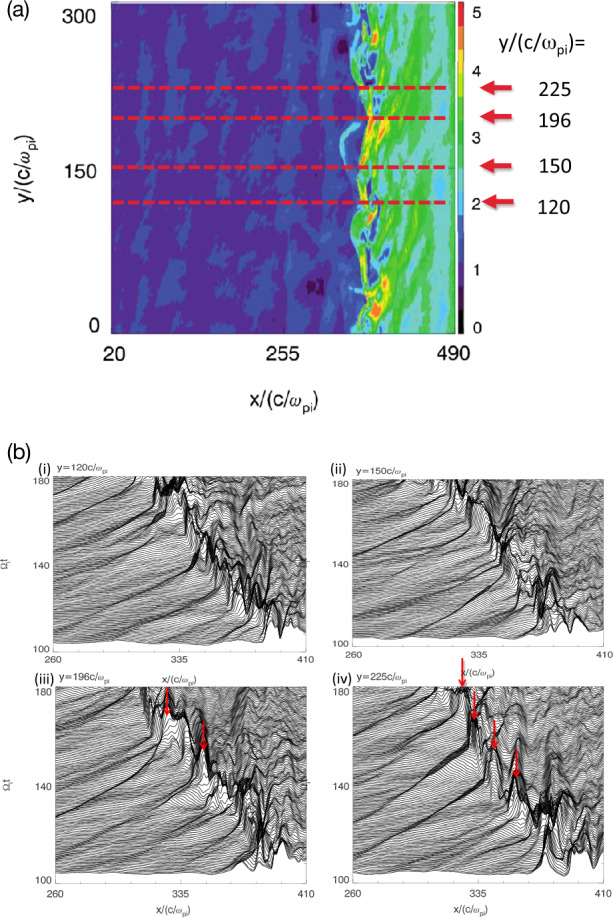
(**a**) Isocolors of the total magnetic field B(x,y) amplitude measured at late time of the 2D hybrid simulation; (**b**) Time variation of the total B field when measured at the four fixed locations y= (i) 120, (ii) 150, (iii) 196, et (iv) 225 c$/\omega _{pi}$ indicated by horizontal dashed lines in plot (**a**); red arrows indicate the cyclic self-reformation of the shock front when possible to identify it. (Issued from Hao et al. 2016)

Another confusion may also appear due to the over-use of the “self-reformation” label in the literature. As well known, nonlinear structures/shocklets/SLAMS have a strong impact on the shock front itself. While convected back to the shock front, these interact/merge with it and are responsible for the strong inhomogeneity (front ripples) and nonstationarity of the front. For clarifying, we need to distinguish “nonstationary” effects and “self-reformation” processes. Let us remind that both 1D hybrid simulations (Burgess 1989a; Scholer and Burgess 1992) and 1D PIC simulations (Scholer et al. 2003; Tsubouchi and Lembège 2004) have shown that this nonstationarity leads to a self-reformation of the shock front (characterized by a cyclic time period). But, the situation strongly differs as shown in more recent 2D hybrid simulations (Hao et al. 2016, 2017). Indeed, because of the presence of large amplitude ripples at the shock front, the self-reformation cannot be synchronous along the whole front; only “local” self-reformation (i.e. over a small part of a ripple) can take place. Moreover, this “local” self reformation is only “intermittent” since the features/locations of the ripples vary in time as upstream nonlinear ULF waves/SLAMS are continuously convected back by the solar wind and hit the shock front at different locations (Hao et al. 2016). This is illustrated by red arrows in Fig. 25, which shows that the self-reformation strongly depends on the y-locations. The shock front is nonstationary in plots (i) and (ii) but no cyclic self-reformation can be clearly identified. However, the self-reformation can be identified at some other y-locations but is intermittent (i.e. not continuous in time) as in plots (iii) and (iv). In other words, a crossing of a multi-satellites set moving along a local shock normal can identify a local self-reformation during a certain time range. In contrast, a similar satellites crossing performed at different y-locations (if local $y$-axis is assumed along the shock front) may confirm that the shock front is nonstationary but no local self-reformation can be identified.

#### Transient, Local Ion Foreshocks

Omidi et al. (2013a) used several global hybrid simulation runs for steady and time-varying IMF conditions to study the dynamics of the FCB and examine the relationship between the FCB and foreshock cavities generated. In one of their runs, two discontinuities were launched convecting along the bow shock surface. The IMF was quasi-parallel and perpendicular to the bow shock normal between and outside two discontinuities, respectively. As a result, a local foreshock formed between two discontinuities bounded by two FCBs. Such a local foreshock was called the traveling foreshock as it traveled with the IMF between two discontinuities. As for the regular foreshock, its boundary is due to the curved bow shock surface that varies $\Theta _{Bn}$ to be quasi-perpendicular. In their another run, when the IMF was time varying, the boundary of the foreshock oscillated back and forth. If there is spacecraft close to the boundary, it will temporarily enter the foreshock from the solar wind and exit resulting in an observation signature that is consistent with foreshock cavities.

Kajdič et al. (2017) used observations from five THEMIS spacecraft that were in a string-of-pearls formation to distinguish the two scenarios described in Omidi et al. (2013a). They found a foreshock bounded by two FCBs. Based on the time sequence of multiple spacecraft observations, they determined that the foreshock was traveling with two discontinuities rather than oscillating back and forth. This foreshock was thus identified as a traveling foreshock. Kajdič et al. (2017) suggested that isolated foreshock cavities could be a subset of traveling foreshocks.

**Fig. 26:**
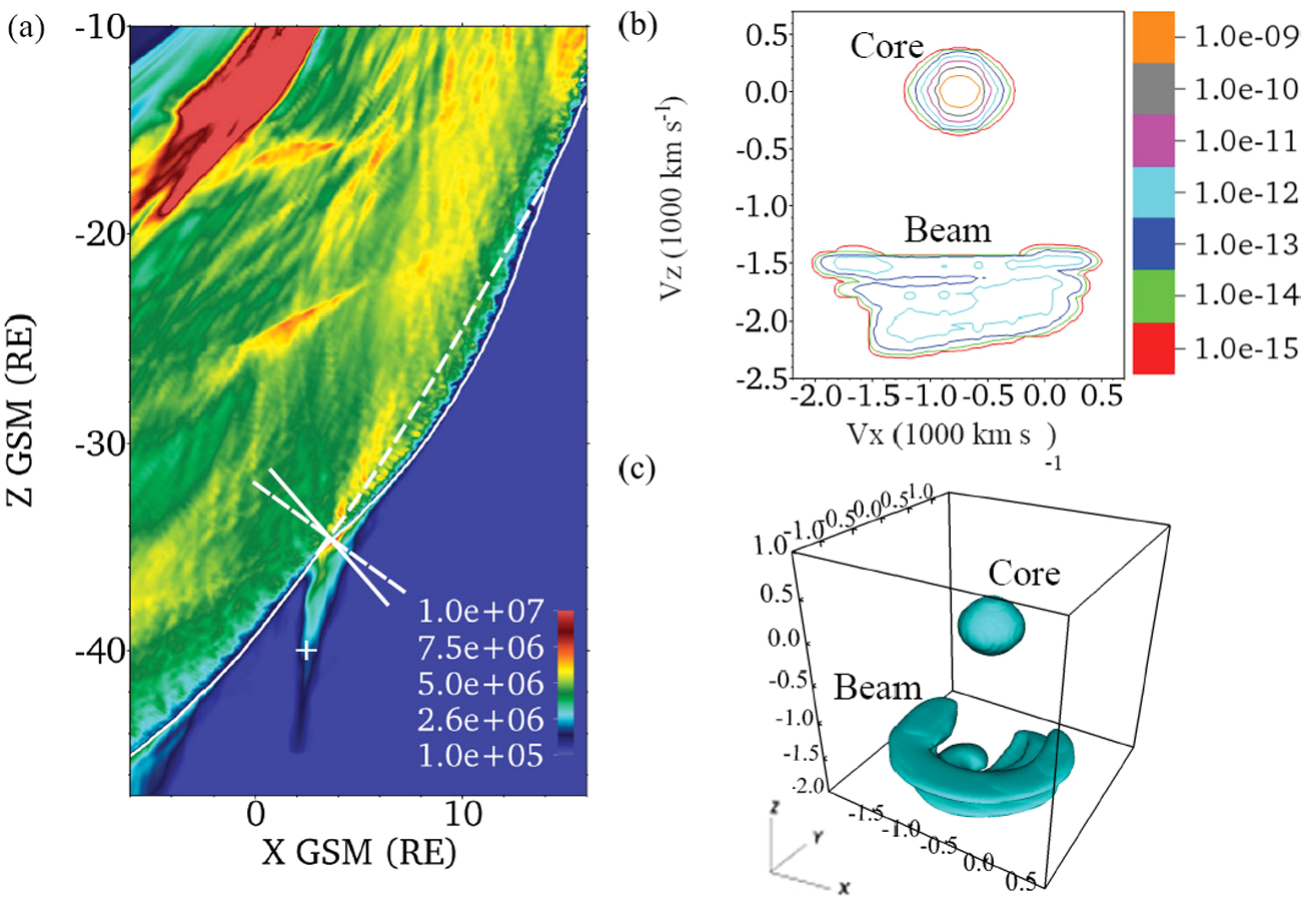
(**a**) A local foreshock in the simulation with color indicating the parallel temperature that is sensitive to the presence of an ion beam. The solid white curve shows the bow shock location based on the density contour. The dashed white curve illustrates the approximate bow shock location without perturbations in the magnetosheath. The solid and dashed segments indicate the direction normal to each curve ($\Theta _{Bn}$: 41^∘^ and 54^∘^). (**b**) 2-D projected contour and (**c**) 3-D contour plots of the ion velocity distribution (PSD in s^3^ m^−6^; 3-D contour at $1\times 10^{-15}~\mbox{s}^{3}\,\mbox{m}^{-6}$) at the white cross in panel (**a**). Both distributions show the field-aligned beam that is directly comparable to Figs. 2 and 6 in Kempf et al. (2015). (From Pfau-Kempf et al. 2016, Fig. 5)

Pfau-Kempf et al. (2016) presented a scenario resulting in time-dependent behavior of the bow shock and transient, local ion reflection under steady solar wind conditions. Dayside magnetopause reconnection creates FTEs which drive fast-mode wavefronts in the magnetosheath. These fronts create a bulge on the bow shock surface because of their large downstream pressure. The resulting bow shock deformation leads to a configuration favorable to localized ion reflection. This process has been identified in 2D global Vlasiator simulations (Fig. 26). Pfau-Kempf et al. (2016) also presented observational data showing the occurrence of dayside reconnection and FTEs at the same time as Geotail observed transient foreshock-like field-aligned ion beams.

### Understanding Foreshock Effects in Simulations

As noted in Sect. 2.1, the foreshock is the region with magnetic field lines connected to the bow shock and filled with particles backstreaming from the bow shock. Among the different types of backstreaming ions, the formation mechanisms for field aligned beams (FABs) and gyro-phase bunched (GPB) backstreaming ions are still under active investigation. Different “scenarios” have been proposed (Möbius et al. 2001; Kucharek et al. 2004), each of which has some drawbacks. Some scenarios are based on the guiding center approximation and rough conservation of the magnetic moment, i.e. adiabatic reflection (Sonnerup 1969; Paschmann et al. 1980; Schwartz et al. 1983; Schwartz and Burgess 1984). Others invoke simple geometrical considerations consistent with specular reflection (Gosling et al. 1982; Paschmann et al. 1982; Meziane et al. 2001; Yamauchi et al. 2011). Yet others invoke leakage of some magnetosheath ions to produce low energy FABs (Edmiston et al. 1982; Tanaka et al. 1983; Thomsen et al. 1983). Particle diffusion processes have also been invoked to explain the diffusion of reflected “gyrating ions” by upstream magnetic fluctuations (Giacalone et al. 1994a) and the ion diffusion that takes place within the shock ramp due to pitch angle scattering during the reflection process (Möbius et al. 2001; Kucharek et al. 2004; Bale et al. 2005). Understanding the origin of the GPB population poses an even greater problem. These ions are observed near the quasi-parallel shock front (Gosling et al. 1982; Meziane et al. 2004) after undergoing specular reflection. However, they can also be observed at some distance from the shock front (Thomsen et al. 1985; Fuselier et al. 1986). These synchronized nongyrotropic distributions can be explained by different processes: (i) trapping by low-frequency monochromatic waves (Mazelle et al. 2003, 2005, 2007; Hamza et al. 2006), or (ii) beam-plasma instabilities (Hoshino and Terasawa 1985) which trap ions and can create the GPBs, or (iii) short time interaction with the macroscopic electric and magnetic fields at the front (Savoini and Lembège 2015). However, it is difficult to distinguish between these different possibilities which may or may not be present simultaneously.

The different ion populations within the ion foreshock exhibit spatial distributions whose characteristics depend upon distance from the bow shock, the length of time period when the field lines have been connected to the bow shock, and bow shock curvature (Eastwood et al. 2005). This point has stimulated several global foreshock simulations, still few in number in contrast to experimental studies. Three different global approaches are under use: (i) 2D/3D Hybrid-PIC, (ii) 2D Hybrid-Vlasov, and (iii) 2D Full PIC simulations.

**Fig. 27:**
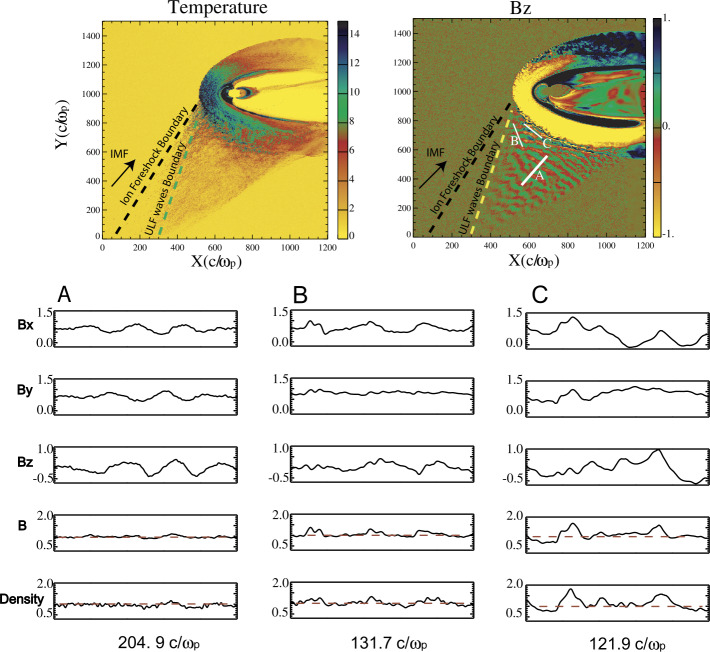
(Top) Temperature and $B_{z}$ magnetic field component within the simulation box. The ion foreshock boundary, the ULF waves boundary (so called ULFWB in Sect. 2.1), and the direction of the IMF are indicated. (Bottom) Waves profiles along three trajectories within the foreshock. Cut A shows sinusoidal waves, while cuts B and C illustrate compressive wave profiles. The number below cuts A-C corresponds to the extension of each trajectory expressed in ion inertial length c/$\omega _{pi}$. (From Blanco-Cano et al. 2006)

a) Two dimensional Hybrid-PIC simulations (kinetic ions and fluid electrons) have been performed by different authors for the full range of Q_⊥_ and Q$_{\parallel }$ foreshock regions, i.e. for the full range of angles between the IMF and the normal to the bow shock (Karimabadi et al. 2014; Blanco-Cano et al. 2006). In particular, Blanco-Cano et al. (2006) have analyzed coupling between the solar wind and the terrestrial magnetosphere for magnetic dipoles with different strength and consequences of these choices on the foreshock morphology and associated wave activity. Basically, they find two types of ULF waves excited by kinetic instabilities generated by two different ion populations (Fig. 27): (i) sinusoidal almost parallel propagating waves generated by FABs of backstreaming ions via the right-hand (RH) resonant instability, and (ii) highly compressive obliquely propagating fluctuations near the shock generated by gyrating ion beams closer to the front. A comparison of these results with observations show that the features of the sinusoidal waves (i) resemble the properties of 30-sec sinusoidal quasi-monochromatic waves in the foreshock. By contrast, the compressive waves exhibit properties similar to the observed right-handed steepened fluctuations. Results have also been compared with previous experimental work obtained from the Cluster mission (Mazelle et al. 2003, 2005; Meziane et al. 2004).

**Fig. 28:**
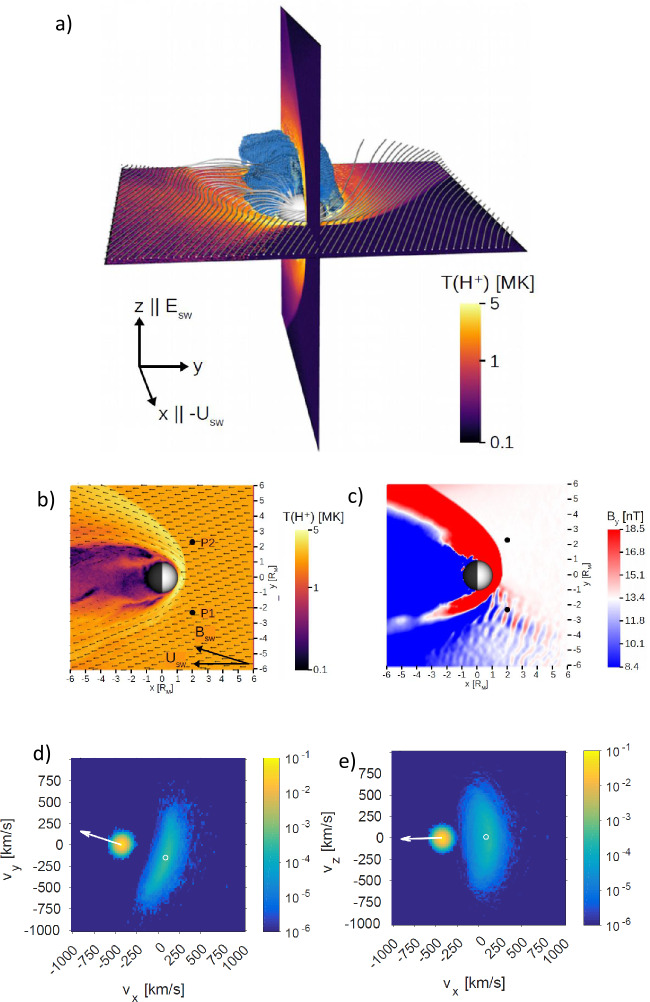
Examples of parameters from a global 3D hybrid-PIC simulation of the Mercury-solar wind interaction and the ion foreshock. (**a**) The magnetic field lines near Mercury. The solar wind is flowing along the -x direction. The temperature of the solar wind protons is shown in equatorial ($x-y$) and meridian ($x-z$) planes. (**b**) The density of the solar wind protons in the equatorial plane at $z = 0$. The black arrows show the solar wind bulk velocity. (**c**) The $B_{y}$ component of the magnetic field in the equatorial plane at $z = 0$; (**d**, **e**) The velocity distribution function of H^+^ ions measured on the point P1 shown in panel (**b**). In (**d**) the 3D velocity distribution function is projected on the $V_{x}$-$V_{y}$ plane and in (**e**) on the $V_{x}$-$V_{z}$ plane. The white arrow shows the direction of the average magnetic field vector. (Jarvinen et al. 2019)

The challenge in the three dimensional (3D) hybrid-PIC simulation of the Earth’s foreshock is their high computational costs because of the large spatial scale compared with ion’s kinetic plasma scales (ion gyroradius, ion skin depth). However, computational cost can be reduced by investigating Mercury-solar wind interaction where the size of the bow shock and the magnetosphere is smaller than at Earth because of Hermean small intrinsic magnetic field compared to Earth. Recent 3D hybrid-PIC Mercury simulations suggest the planet has a dynamic and well-developed ion foreshock and a suprathermal, and partly backstreaming, foreshock ion population, which is associated with coherent, large-scale ULF waves at ∼5 s period (Jarvinen et al. 2019). This recent work shows that local ion distribution within the foreshock has a crescent shape resembling the intermediate distribution in the terrestrial ion foreshock, away from the bulk solar wind population (Figs. 28d and 28e).

However, some phenomena seen at the Earth’s foreshock may not correspond to Mercury’s foreshock for several reasons. First, Mercury does not have an ionosphere through which magnetospheric currents can close. The electric current can, however, close through planetary interior provided that the conductivity of the planet is large enough (Janhunen and Kallio 2004). Second, Mercury’s intrinsic magnetic field is weak, only about 190 nT at the magnetic equator (Anderson et al. 2011; Hauck et al. 2013), compared with the IMF so that part of the planetary surface is continuously magnetically connected to the solar wind (Kallio and Janhunen 2003). Therefore, the Hermean magnetosphere is a “pocket size magnetosphere” and consequently, also the time scales of plasma physical processes are smaller than in the Earth’s magnetosphere. Third, the plasma parameters at Mercury’s orbit at 0.31-0.47 AU differs considerably from plasma parameters at the Earth distance. Especially, the average Parker spiral angle is much smaller at Mercury than at Earth resulting in different parallel-perpendicular bow shock conditions at Mercury compared with the Earth’s bow shock.

**Fig. 29:**
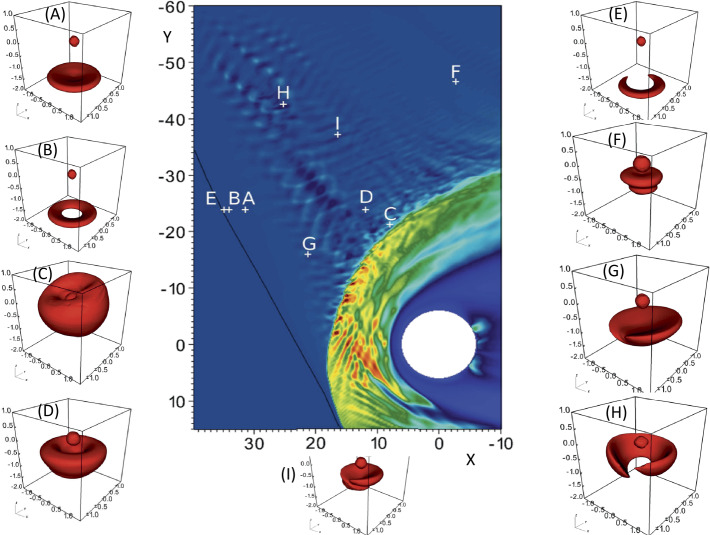
Central plot represents the ion number density in the ion foreshock and magnetosheath regions of the global 2D Hybrid Vlasov simulations; Color code (not shown here) extends from low (blue: 6.50 e+04) to high (red: 5.50 e+06) values of the ion number density. Black line represents the ion foreshock edge upstream of which there is only Maxwellian distribution of solar wind ions; this edge starts in the Q_⊥_ shock region at angle lower than 90^∘^ from the front (not to be confused with the location of the electron foreshock edge excluded in this simulation). White plus signs and letters A-I represent the location of the ion distribution functions which are shown outside the central plot following: A for FAB; B for ring beam; C for diffuse distribution; D for intermediate; E for partial ring beam; F for multicap distribution; G for lightly disturbed cap; H for strongly disturbed cap; I for spiral distribution respectively. Coordinates XGSE-YGSE plane are scaled in Earth radii ($R_{\mathrm{{E}}}$). (Inspired from results of Kempf et al. (2015))

**Fig. 30:**
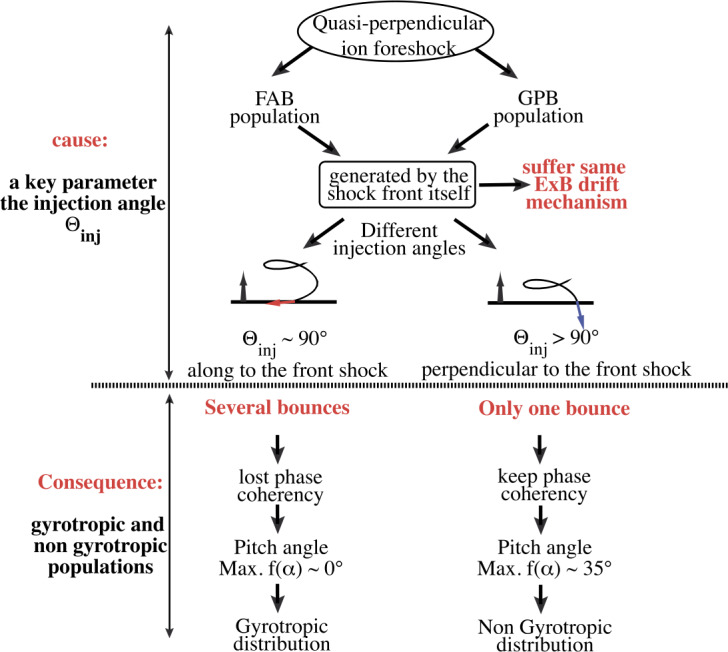
Schematic diagram of the scenario of processes involved in the formation of the quasi-perpendicular ion foreshock (from Savoini and Lembège 2015)

b) Two-dimensional “global” Hybrid-Vlasov simulations (Vlasov ions and fluid electrons) performed by Kempf et al. (2015) reproduce the well known foreshock ion distributions very well and have been compared with observations from the THEMIS mission. The simulations reproduce two important features: (a) the decrease of the backstreaming beam speed with increasing radial distance from the edge of the foreshock, and (b) beam speed increases and density decreases with increasing radial distance from the bow shock. More precisely, FAB and ring beam are seen at the upstream edge of the foreshock; in contrast, deeper within the foreshock (further from the edge of the foreshock) intermediate distributions dominate which eventually get disrupted by ULF waves to become gyrophase-bunched distributions. This supports the scenario proposed by Meziane et al. (2001), in which the foreshock waves are the consequences of the ion beam activity generated by backstreaming ion populations and the GPB ion distributions associated with strong ULF waves result from beam disruption by the waves. Figure 29 summarizes the locations of the different ion distributions in the ion foreshock in results from the 2D Hybrid-Vlasov simulations (Kempf et al. 2015).

c) Two-dimensional “global” PIC simulations (fully kinetic ions and electrons) of the curved shock and associated ion foreshock region have been performed by Savoini et al. (2013) and Savoini and Lembège (2015). Because of computational constraints, the analyses were restricted to the Q_⊥_ region ($90^{ \circ } > \Theta _{Bn} > 45^{\circ }$). These studies concentrated on GPB and FAB ion distributions observed near the front as ULF waves did not have time to reach substantial amplitudes. Both populations have been identified as occurring self-consistently and spatial mapping of local FAB and GPB distribution functions show that these can be observed simultaneously. One striking feature is that these distributions result only from their interaction with the macroscopic fields at the front; no waves or instabilities are necessary. Both statistical study and time trajectory analysis have shown that these populations can be differentiated from each other solely by the time spent within the shock front, from a short time (involving Fermi type acceleration) to a long time (with a mechanism still under investigation). The access to these different time ranges depends primarily on the so-called “injection angle” defined between the ion gyrating velocity vector and the local shock normal at the time the ion hits the front; no specific initial conditions need to be satisfied for the incoming solar wind ions in terms of energy, velocity distributions or pitch angle. In addition, these studies show that the nonstationarity of the shock front may have a strong impact on the backstreaming particles, since incoming particles will see different shock profiles when they strike the front and during their interaction time (short or long) with the front (Fig. 30).

Recently, Savoini and Lembège (2020) have analyzed more deeply the formation processes of backstreaming ions into the upstream ion foreshock after interacting with a quasi-perpendicular curved front by using two different approaches of test particle simulations: (i) a fully consistent expansion (FCE) of the front which includes all self-consistent shock profiles at different times (time dependence included), and (ii) an homothetic expansion (HE) model where shock profiles are chosen at certain fixed times and are artificially expanded in space (time dependence excluded); in both cases, the shock profiles are issued from a previous full 2D-PIC simulation. For each case, particles have been released from different boxes initially located along the curved front within the shock angular range ($90^{\circ } > \Theta _{Bn} > 45^{\circ }$) in order to determine the impact of the front curvature. These combined approaches have allowed to analyze the origin and formation of backstreaming ions under angular and/or temporal dependences separately. Main results show that the formation of ion foreshock is not a continuous process but is time dependent which leads to bursty emissions of backstreaming ions. Moreover, four different processes contribute to the formation of backstreaming ions: (i) the electrostatic $E_{l}$ field component which has a strong impact particularly for high $\Theta _{Bn}$ angles (i.e. when approaching the edge of the ion foreshock), (ii) the magnetic field B (via the magnetic mirror reflection) whose impact is particularly relevant for lower $\Theta _{Bn}$, (iii) the $E_{t} \times B$ drift in the velocity space mainly supported by the convective $E_{t}$ electric field which is necessary to generate both FAB and GPB populations (Savoini and Lembège 2015), and (iv) a comparison of results obtained between the simulations indicates that fields’ time variations (FCE case) are much more efficient at diffusing particles than the fields’ spatial variations (HE case). A deep impact of shock front non-stationarity is more difficult to analyze for two reasons: a) the time-of-flight effects mix reflected ions issued from different shock profiles (met at different times and at different locations of the curved front); (b) some shock profiles have been shown to be more efficient than others (in time) for reflecting ions, but the differences of their respective impacts rapidly disappear since they are being blurred out by the impact of less efficient profiles on particles as time evolves.

**Fig. 31:**
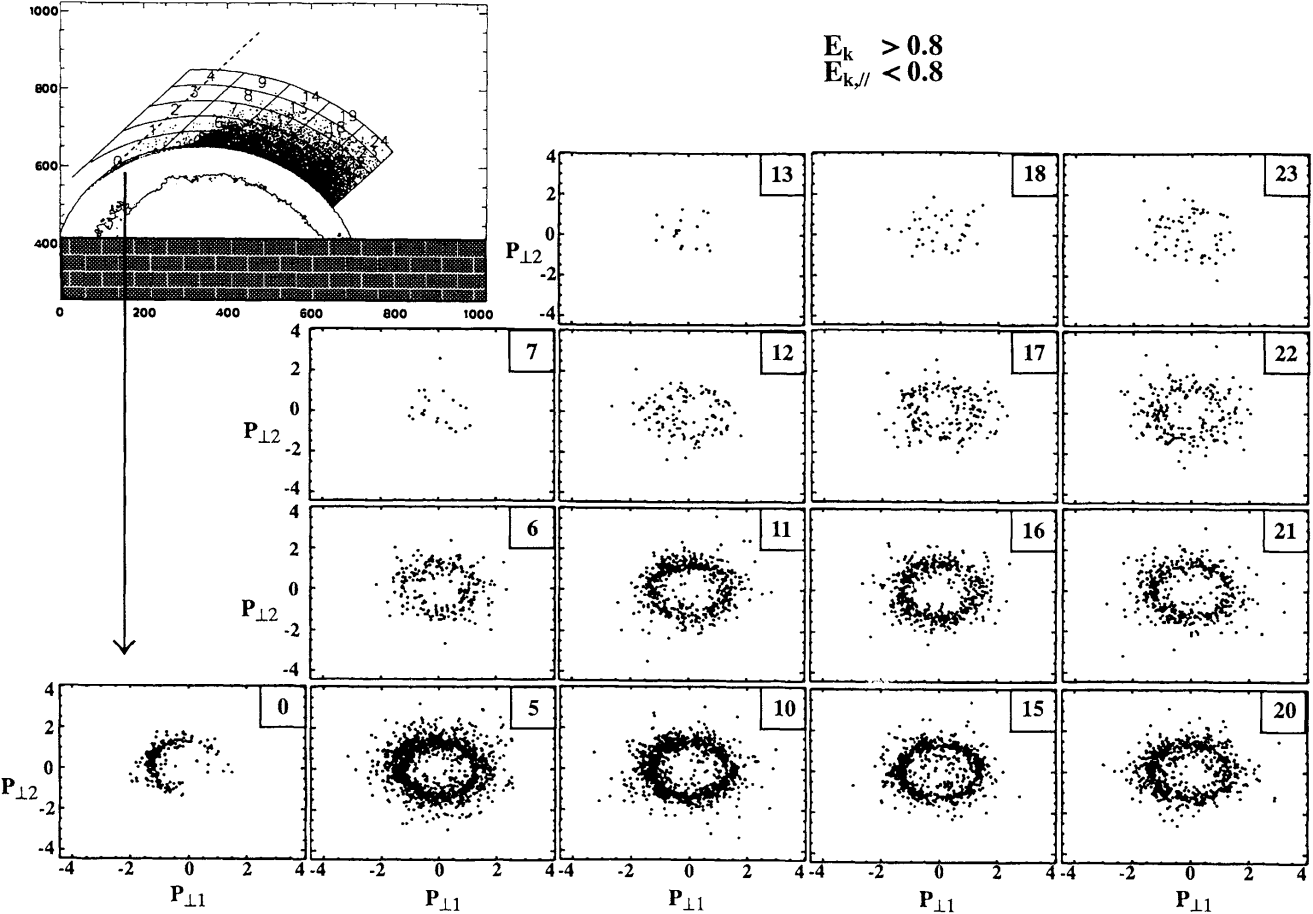
Local electron perpendicular momenta space of backstreaming electrons selected with low parallel kinetic energy only, represented respectively within each sampling box labelled from 0 to 24. For reference, the left-hand top panel illustrates locations of the curved shock front, of the backstreaming electrons, and of the sampling boxes within the simulation plane; the foreshock edge is indicated by the projected magnetic field line tangent to the curved front (dashed line). (From Savoini and Lembège 2001, Fig. 9b)

At last, by using 2D large scale PIC simulations, the electron foreshock has been analyzed self consistently by Savoini and Lembège (2001) who recovered local electron distribution functions and velocity space in good agreement with local experimental observations (Feldman et al. 1983; Fitzenreiter et al. 1990). Basically, three different types of electron distributions have been identified according to their penetration depth within the front (Savoini and Lembege 2010): (i) the “magnetic mirrored” ones which only suffer one specular reflection at the front; (ii) the “trapped” ones which suffer a local trapping within the parallel electrostatic potential at the overshoot, and (iii) the “leaked” electrons which penetrate even more deeply into the downstream region before being reinjected back upstream. The low energy part electrons (i) are characterized by a loss cone distribution (and a ring in the perpendicular velocity space), while the high energy part electrons ((ii) and (iii)) contribute to the bump-in-tail part of the electron distribution (Fig. 31).

In summary, “global” hybrid simulations allow us (i) to cover the full range of ion foreshock shock normal angles and up to large distances from the shock front, but exclude consideration of the electron foreshock and (ii) to analyze any ion wave activity, but (iii) are often restricted by the limited spatial resolution which impacts shock front and upstream wave steepening (and in return local ion interactions). By contrast, current “global” 2D PIC simulations are restricted to short distances from the shock front (a few Earth radii only) and to Q_⊥_ shocks at present time. As a consequence, they predicted no ion wave activity and as yet unstudied electron wave activity, but did (i) reproduce the spatial ranges and kinetic features of both the ion and electron foreshocks (for both statistical and time trajectory analysis), (ii) provide access to shock front scale sizes smaller than those associated with ions (in particular the shock ramp and/or steepened precursor) which can affect particle acceleration processes at the front, and (iii) include some nonstationary processes of the front self-consistently (see review by Lembege et al. (2004)) on spatial scales smaller than those associated with ions which can also impact local acceleration processes. Consequently, hybrid and PIC simulations play complementary roles. As a final reminder, one must keep in mind that backstreaming particles represent only a small percentage of incoming solar wind particles, and a significant number of particles are needed to obtain satisfactory results for backstreaming populations within the foreshock.

### Magnetosheath Transients

The bow shock may be considered as both an energy converter (transforming the bulk motion of the upstream solar wind plasma into heated plasma in the downstream region) and a source of energetic particle acceleration in upstream region. But, signatures of energy conversion are very different between Q_⊥_ and Q$_{\parallel }$ regions. For Q_⊥_ region, a large part of incoming plasma is directly transferred to downstream region while a certain percentage is reflected by the shock front. Depending on the angle between the shock normal and the upstream IMF, reflected ions suffer one large gyromotion under the magnetic field before penetrating the downstream region (for a ${\Theta _{Bn}}=90^{\circ }$ shock) or suffer a distorted gyromotion and a part of reflected ions flows back upstream along the magnetic field (as $\Theta _{Bn}$ departs from 90^∘^). Then, different instabilities can take place in downstream region which have been analyzed by different authors: (i) relaxation of the ion ring formed by the gyrating ions, (ii) strong temperature anisotropy driven instabilities (so called the mirror-type instability and the Alfvén ion cyclotron instability) since ions suffer an important perpendicular heating at the front and downstream, which results in a noticeable temperature anisotropy (Winske and Quest 1988; Shoji et al. 2009).

The situation strongly differs for Q$_{\parallel }$ shocks which are much less quiet than Q_⊥_ shocks. Surprisingly, some filamentary structures have been identified (under different labels within the last two decades) in experimental results. It was effectively a surprise, since these structures are mainly observed in the downstream of Q$_{\parallel }$ shock, have an apparent well coherent structure and have been shown to persist far downstream in the magnetosheath. These structures have been well retrieved in simulations and have stimulated many efforts (still in course) in a detailed comparison between experimental/numerical simulation analyses.

In addition to these transients inherent to the dynamics of the shock front itself, some structures/transients are directly carried by the solar wind itself, interact with the shock front, and succeed to penetrate the magnetosheath. The sections below illustrate the diversity of these transients. The present main goal is to reach a synthetic view and to evidence that many of these are related to each other.

#### Magnetosheath Jets/Magnetosheath Filamentary Structures

Two decades ago, observations from Interball-1 and Magion-4 satellites (Němeček et al. 1998) have evidenced the presence of “enhanced structures” more precisely of “transient flux enhancements” in dynamic pressure within the downstream region of Q$_{\parallel }$ shocks; these structures are typically caused by increases in plasma velocity. Similar structures have also been identified by Savin et al. (2008) with Interball-1 and Cluster satellites, which have been named “high kinetic energy jets” and later “high speed jets”, “HSJs”, or “magnetosheath jets”. Later on, statistical studies have identified more clearly the main features of magnetosheath jets: (a) magnetosheath jets are defined as intervals when the plasma dynamic pressure along the anti-sunward direction in the subsolar magnetosheath is greater than half of the upstream solar wind value; (b) relative to the ambient magnetosheath, magnetosheath jets exhibit some enhancements of both density and magnetic field intensity but lower and more isotropic temperatures; (c) magnetosheath jets occur predominantly downstream of Q$_{ \parallel }$ shocks (i.e. when IMF cone angles are low), but jet occurrence is only very weakly dependent on other upstream plasma conditions or solar wind variability; (d) typical duration and recurrence times are from a few seconds to several minutes or have a spatial scale less (or sometimes higher) than one $R_{\mathrm{{E}}}$ (Archer et al. 2012; Hietala et al. 2012; Plaschke et al. 2013); and (e) magnetosheath jets are almost always super-Alfvénic and often even super magnetosonic. Consequently, magnetosheath jets are likely to have important effects on the magnetosphere and ionosphere if these impinge on the magnetopause. Based on Cluster observations, Hietala et al. (2012) have shown that magnetosheath jets with very high dynamic pressure can perturb the local magnetopause; during the interval of jet observations, irregular pulsations were observed at the geostationary orbit and localized flow enhancements were detected in the ionosphere, suggesting that magnetosheath jets can cause inner magnetospheric phenomena (see Sect. 3.3).

Different scenarios have been proposed in order to identify the source mechanisms of these magnetosheath jets: (i) early works of Němeček et al. (1998) and later Savin et al. (2008) did not identify a clear source mechanism for the magnetosheath jets but only invoke a local reconnection process; (ii) Lin et al. (1996a,b) have analyzed the interaction of the bow shock with an RD moving in the IMF by using hybrid and magnetohydrodynamic models; (iii) Savin et al. (2004, 2008, 2012) have proposed that HFAs may locally trigger magnetosheath jets by keeping the local flux balance due to the bow shock deformation by HFAs; (iv) based on Cluster experimental observations, Hietala et al. (2009, 2012) have proposed a scenario in which magnetosheath jets are due to local ripples inherent to a quasi-parallel shock (which cause the local curvature variations in the shock front) combined with the nonstationarity of the shock front. This scenario invokes the presence of a secondary shock as a super-fast-magnetosonic jet encounters the magnetopause; then, the jet is not decelerated (or very weakly) but only deviated by this secondary shock provided that the local angle between the upstream bulk velocity and local shock normal is large (due to the local large scale front rippling). Through a quantitative comparison study between magnetosheath jets and quasi-parallel bow shock ripples, Hietala and Plaschke (2013) concluded that 97% of the observed jets can be generated by local ripples.

A very detailed analysis of the internal structure of a special leading “jet” has been recently performed by Plaschke et al. (2017) based on observations made by the MMS mission, which reveal large amplitude density, temperature and magnetic field variations inside the jet, over small scale/short periods of time. These structures mainly convect with the jet plasma; the leading jet is the strongest and exhibits the largest dynamic pressure. Then, during an interval when repeated jets were observed, the plasma velocity varies significantly including sunward plasma flows in the subsolar magnetosheath: in other words, within the jet, structures propagate forward in the jet’s core region and backward outside of that region. Unfortunately, due to the short interdistance between the MMS spacecraft, it was not possible to confirm that the sunward flows were caused by the nearby passage of magnetosheath jets. Recent statistical work of Plaschke and Hietala (2018) based on 662 events observed by two THEMIS satellites (one observing the jet, the other one providing observations of nearby plasma to uncover the flow patterns in and around the magnetosheath jet), has clarified this point: (a) along the jet’s path, slower plasma is accelerated and pushed aside ahead of the fastest core jet plasma; (b) behind the jet core, plasma flows into the path to fill in the wake. This plasma motion affects the ambient magnetosheath close to the jet’s path. Diverging and converging plasma flows ahead and behind the jet are complemented by plasma flows opposite to the jet’s propagation direction in the vicinity of the jet. In the frame of reference of the background magnetosheath flow, the plasma clearly performs a vortical motion. This vortical motion leads to a deceleration of the ambient plasma when a jet passes by.

All these observations have strongly stimulated numerical works in terms of global and local simulations. Global 2D hybrid simulations of Karimabadi et al. (2014) have observed downstream structures similar to magnetosheath jets and indicated that it is possible to form a bow wave/shock ahead of a super-fast-magnetosonic magnetosheath jet during its propagation deeper into the magnetosheath with a typical Mach number $M_{\mathrm{{A}}}=$ 8, to be compared with the experimental conditions ($M_{\mathrm{{A}}}=$ 12) of Hietala et al. (2009, 2012). Other global 2D hybrid simulations (Omidi et al. 2014a) have focussed on the main features of downstream filamentary structures and shown that these form over a wide range of solar wind $M_{\mathrm{{A}}}$ and IMF cone angles. The formation of these structures is connected to the existence of localized regions with increased ion temperature at and upstream of the Q$_{ \parallel }$ shock. Upon injection of these energetic ions into the downstream region, they follow the field line and the enhanced pressure in flux tubes containing the shock accelerated ions depletes the thermal plasma in these flux tubes and enhances density in the surrounding flux tubes without energetic ions. As a consequence, an anticorrelation is observed between plasma density and ion temperature within the filamentary structures.

**Fig. 32:**
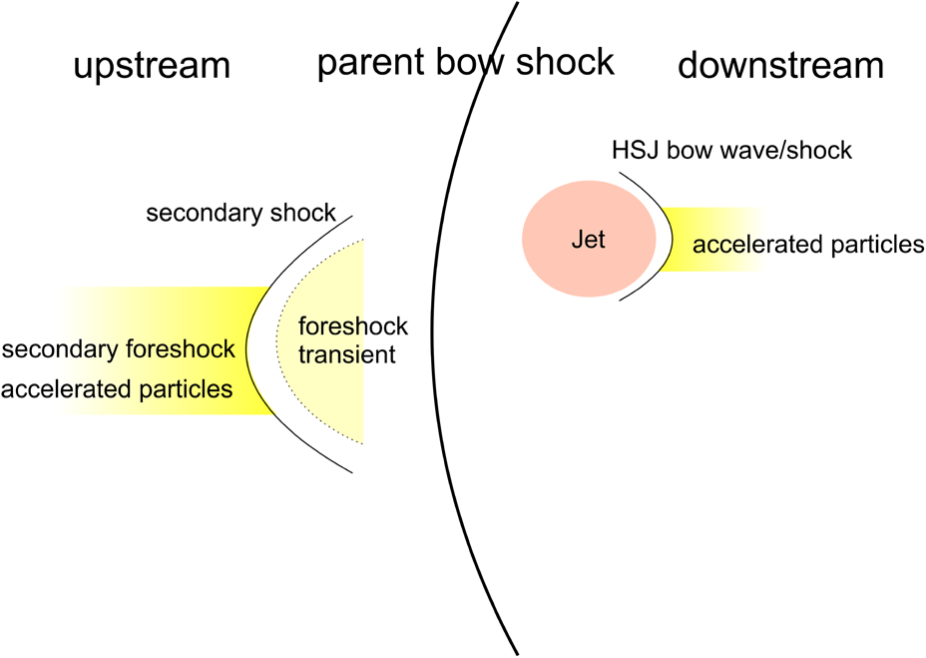
A sketch illustrating the presence of nonlinear transient structures, such as foreshock transients and magnetosheath jets, both upstream and downstream of the primary shock. They can form a secondary bow wave/shock which can accelerate particles and contribute to particle acceleration at the primary shock. HSJ holds for high speed jet. (From Liu et al. 2020b)

Recent THEMIS observations identify that when a magnetosheath jet is super-fast-magnetosonic relative to the ambient magnetosheath flow, a bow wave forms ahead of it (Liu et al. 2019b) consistent with simulations by Karimabadi et al. (2014). Case studies show that ions/electrons are accelerated at magnetosheath jet-driven bow waves likely through grad-B drift along/against the convection electric field (Liu et al. 2019b, 2020c). A statistical study by Liu et al. (2020b) shows that it is common for the bow waves to accelerate particles and large solar wind dynamic pressure, large Mach number, and large solar wind plasma beta favor the occurrence of the bow waves. These results suggest that under favorable upstream conditions, magnetosheath jets can contribute to particle acceleration, especially at high Mach number shocks. In Sect. 2.3.6, foreshock transients are shown to accelerate particles, which implies that nonlinear structures both upstream and downstream of shocks should be included in shock acceleration models (Fig. 32).

**Fig. 33:**
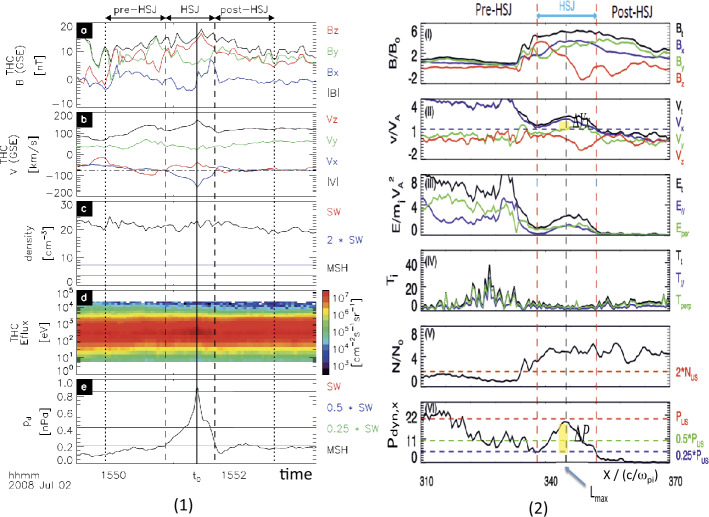
Comparison of the main signatures of one magnetosheath jet measured (1) in experimental observations of THEMIS (Plaschke et al. 2013) and (2) in 2D local hybrid simulations (Hao et al. 2016). From the top to bottom of Panel (1), (plots **a** to **e**): magnetic field and ion velocity measurements in GSE, ion density, ion energy flux density and dynamic pressure. (**c**) shows magnetosheath (MSH) measurements in black and the corresponding solar wind (SW) ion densities (n_SW_ and 2⋅n_SW_) are shown in red and blue respectively. (**e**) shows the GSE $x$ component dynamic pressure (P$_{\mathrm{{d,msh,x}}}$) in black; corresponding SW data (P$_{ \mathrm{{d,sw}}}$, P$_{\mathrm{{d,sw}}}/2$ and P$_{\mathrm{{d,sw}}}/4$) are shown in red, blue and green. From the top to bottom of Panel 2 (plots **i** to **vi**): spatial profiles of magnetic field and ion velocity measurements, kinetic energy, ion temperatures, ion density, and $x$ component (solar wind direction) of the dynamic pressure. Caution: the $x$ axis is respectively the UT in Panel (1), and the distance X normalized with respect to the ion inertial length ($c/\omega _{pi}$) in Panel (2)

More recently, using 2D local hybrid simulations of a typical quasi-parallel shock where the angle between the normal to shock front and the IMF is $\Theta _{Bn}=30^{\circ }$, Hao et al. (2016) have analyzed the link between the time dynamics of the shock front and the formation of the associated magnetosheath jets. As well known, the Q$_{\parallel }$ shock front is strongly nonstationary. Some upstream nonlinear ULF waves structures/SLAMS form after the interaction of backstreaming ions and incoming ions and are convected back by the solar wind to the shock front; when interacting with the front, these structures contribute to both its strong spatial inhomogeneity (front rippling which generates strong local curvatures) and to its time nonstationarity (see Sect. 2.4.5). Simulations of Hao et al. (2016) clearly confirm the scenario of Hietala et al. (2009, 2012) and typical features/scales of magnetosheath jets observed experimentally (Fig. 33). However, some differences appear in the formation of the secondary shock. In Hao et al. (2016), since the upstream flow is lower at $M_{\mathrm{{A}}}=$5.5, a secondary shock forms shortly due to the pile-up of nonlinear upstream structures at the front; it is located just downstream of the main shock front and does not propagate deeply within the magnetosheath.

All scenarios (i) to (iii) of magnetosheath jets formation summarized above require the presence of an external structure carried by the solar wind and interacting with the bow shock to generate magnetosheath jets. In contrast, one striking feature of the last scenario (iv) (Hietala et al. 2009, 2012), confirmed by hybrid simulations of Karimabadi et al. (2014) and Hao et al. (2016), is that the front of quasi-parallel shock which is strongly inhomogeneous and nonstationary, succeeds to generate self-consistently well coherent and structured flows persisting downstream even if their occurrence can take place anywhere within the Q$_{\parallel }$ shock domain. The generation of these magnetosheath jets does not need any “external” upstream pressure fluctuations/discontinuities in the solar wind.

#### Mirror Mode/Magnetic Holes/Peaks in the Magnetosheath

Magnetic holes, also termed magnetic cavities, magnetic dips, or depression structures, have an observable magnetic field decrease in a short time span and have been widely observed in the solar wind plasmas (e.g., Turner et al. 1977; Winterhalter et al. 1994; Zhang et al. 2008; Xiao et al. 2010, 2011, 2014), comet magnetospheres (Russell et al. 1987; Plaschke et al. 2018b), terrestrial/planetary magnetosheaths (e.g., Tsurutani et al. 1982; Bavassano Cattaneo et al. 1998; Joy et al. 2006; Lucek et al. 1999; Balikhin et al. 2003, 2009; Walker et al. 2002, 2004; Soucek et al. 2008), magnetospheric cusps (Shi et al. 2009), and the magnetotail (Ge et al. 2011; Sun et al. 2012) since 1970s. Here we mainly focus on linear magnetic holes across which the magnetic field direction does not change significantly. Magnetic peaks, which are sudden enhancements of magnetic strength, are often observed in the magnetosheath of Earth and other planets. Magnetic peaks observed in the magnetosheath are often flux ropes (see Sect. 3.2) or magnetic mirror mode structures.

Magnetic mirror mode structures or mirror waves include both magnetic holes and magnetic peaks. In the downstream of the Q_⊥_ bow shock, ions are mainly heated in the perpendicular direction, creating an anisotropy in the ion temperature with $T_{\perp }>T_{\parallel }$. In this region, the mirror instability can be easily excited, generating mirror mode structures. The threshold of mirror instability in a proton-electron plasma with cold electrons (Hasegawa 1969; Southwood and Kivelson 1993) can be written as

2 $$ R>\frac{T_{\perp }/T_{\parallel }}{1+1/\beta _{\perp }} $$

where $\beta $, $T_{\perp }$ and $T_{\parallel }$ denote the plasma beta, perpendicular and parallel ion temperature, respectively. If this condition is satisfied, non-propagating (in the plasma frame) quasi-sinusoidal compressional waves grow in the linear stage of the mirror instability. This is consistent with some of the structures observed in the magnetosheath, which are usually characterized by a series of magnetic holes (dips and troughs) and peaks (humps). Embedded in the ambient plasma flow, these structures exhibit anti-correlated magnetic field strength and plasma density (e.g., Horbury et al. 2004; Soucek et al. 2008; Yao et al. 2019b), i.e., they are non-propagating and pressure balanced. Generation of magnetic holes by the mirror instability in high $\beta $ anisotropic magnetosheath plasmas was first proposed by Kaufmann et al. (1970). This explanation was later applied to magnetic holes in the solar wind (Tsurutani et al. 1982; Winterhalter et al. 1994; Stevens and Kasper 2007).

**Fig. 34:**
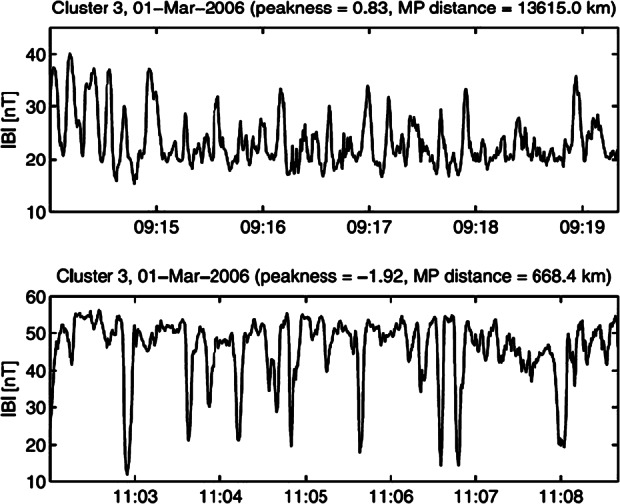
Cluster observation of magnetic peaks (upper panel) and holes (lower panel) in the magnetosheath which were interpreted as mirror mode structures by Soucek et al. (2008)

However, isolated structures observed by satellites in space plasmas often appear as large amplitude magnetic dips or peaks in the magnetosheath where the mirror instability condition described by linear instability theory (Bavassano Cattaneo et al. 1998; Soucek et al. 2008; Génot et al. 2009) is far from satisfied. The mirror instability in the nonlinear stage is likely the source of these structures. These nonlinear effects during this later stage were described theoretically using kinetic approaches (Kivelson and Southwood 1996; Kuznetsov et al. 2007; Pokhotelov et al. 2005, 2008). Numerical simulations suggested that the large-amplitude structures can be stable enough to survive for an extended period of time under mirror stable conditions (Baumgärtel et al. 2003; Califano et al. 2008). Detailed analysis by Balikhin et al. (2009) using THEMIS data showed that the plasma inside magnetic mirror holes is unstable to the linear mirror instability although the ambient plasma is mirror-stable. This observation has been predicted by Pokhotelov et al. (2008) using the nonlinear theory, in which the mirror structures can be stabilized by ions trapped inside them. After tracing the evolution of mirror modes in Saturn’s magnetosheath, Bavassano Cattaneo et al. (1998) found that mirror structures evolve from quasi-sinusoidal waves to non-periodic structures in the form of magnetic peaks and dips, as they propagate from the Q_⊥_ shock to the magnetopause, and finally become dips when close to the magnetopause (Fig. 34). Similar trends were found in the magnetosheath of Jupiter (Joy et al. 2006) and Earth (Soucek et al. 2008; Génot et al. 2009). The nonlinear magnetic peaks and dips and their relationship with the nonlinear stage of the mirror instability are still areas of active research.

Magnetic peaks and holes have also been interpreted as solitary waves, with bright solitons corresponding to magnetic peaks and dark solitons corresponding to magnetic holes. A soliton model based on the Hall-MHD theory was proposed and tested using hybrid simulations (Baumgärtel 1999; Baumgärtel et al. 2003). This model was used to explain some structures observed in the magnetosheath (Stasiewicz 2004a,b). One of the main differences between the mirror mode mechanism and the soliton approximation in observation is that the mirror mode-produced magnetic holes are frozen in the background plasma, while the solitons can propagate in the plasma. Examinations of more magnetic structures detected by four MMS spacecraft are needed to determine their generation mechanisms.

**Fig. 35:**
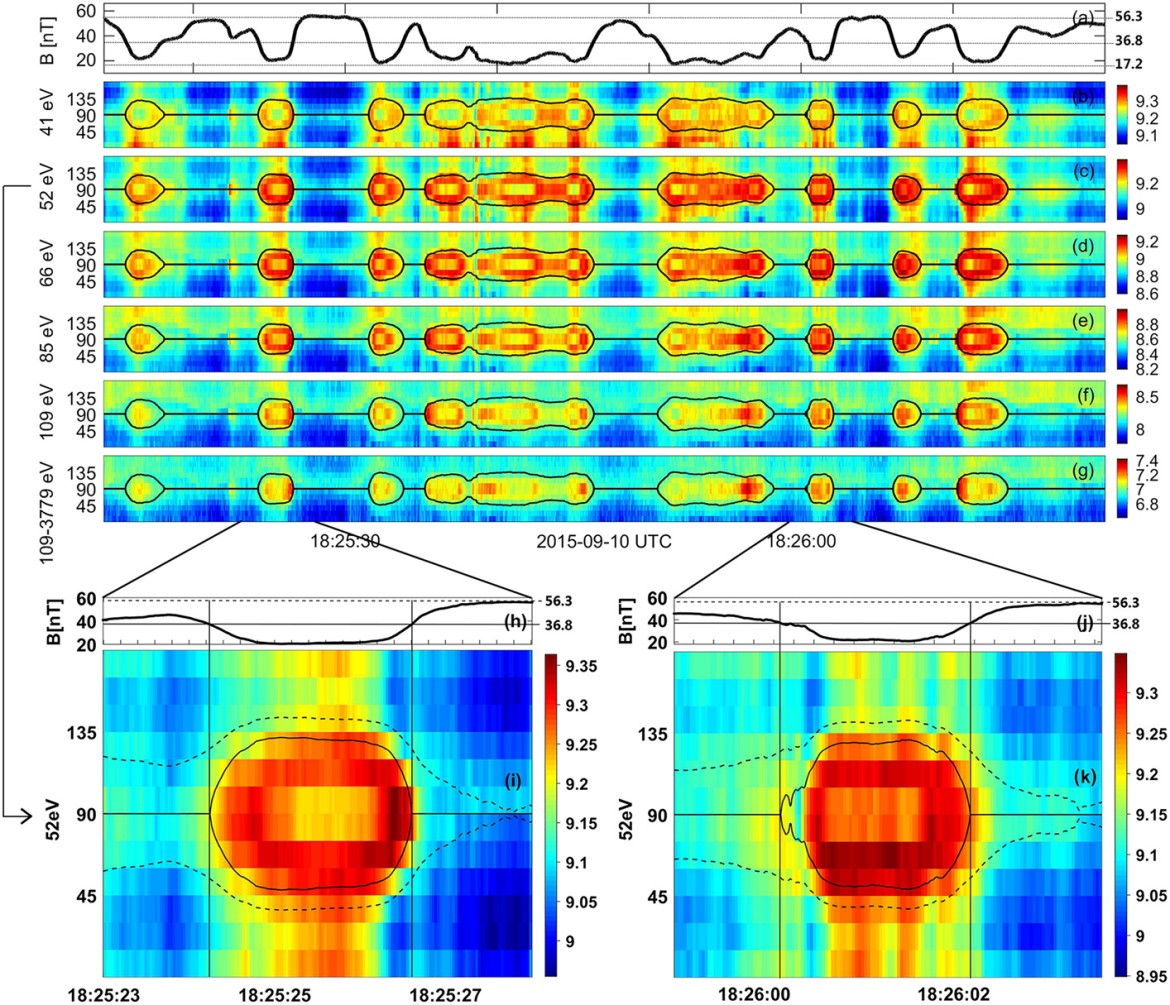
Electron pitch angle distributions from MMS1. (**a**) Magnetic field strength. The three horizontal gray lines mark the maximum, mean, and minimum magnetic field strength in the interval. (**b**-**g**) Electron pitch angle distributions. The black lines represent the local cones. (**h**-**k**) The zoom-in pitch angle spectra of 52 eV electrons with magnetic field strength on the top. The black solid and dashed lines indicate the local cones determined from the mean and maximum magnetic field strength, respectively. (From Yao et al. 2018b)

Particle behaviors in the nonlinear stage of the mirror mode were investigated by Kivelson and Southwood (1996). They pointed out that, during the formation of the mirror mode structure, the trapped ions lose or gain energy by Fermi deceleration or acceleration, respectively. The deeply trapped particles are further decelerated by the betatron mechanism when the magnetic troughs get deeper (e.g., Konjukov and Terietskij 1958; Liu et al. 2017c; Northrop 1963). As a result, particles are decelerated at close to $90^{\circ }$ pitch angles and accelerated at intermediate pitch angles. Chisham et al. (1998) studied the behaviors of the mirror-mode electron distribution in the terrestrial magnetosheath and showed that the deeply trapped electrons are cooled and the shallowly trapped electrons are heated with respect to the rest of the electron velocity distributions. Using high-resolution data from the MMS mission, Yao et al. (2018b) found that most electrons are trapped inside the mirror-mode troughs and exhibit donut-shaped pitch angle distributions (Fig. 35). They demonstrated that donut-shaped distributions are due to betatron deceleration and the spatial dependence of electron pitch angle distributions in these structures. The characteristic donut-shaped distribution of electrons is considered to be related to whistler waves by Ahmadi et al. (2018).

**Fig. 36:**
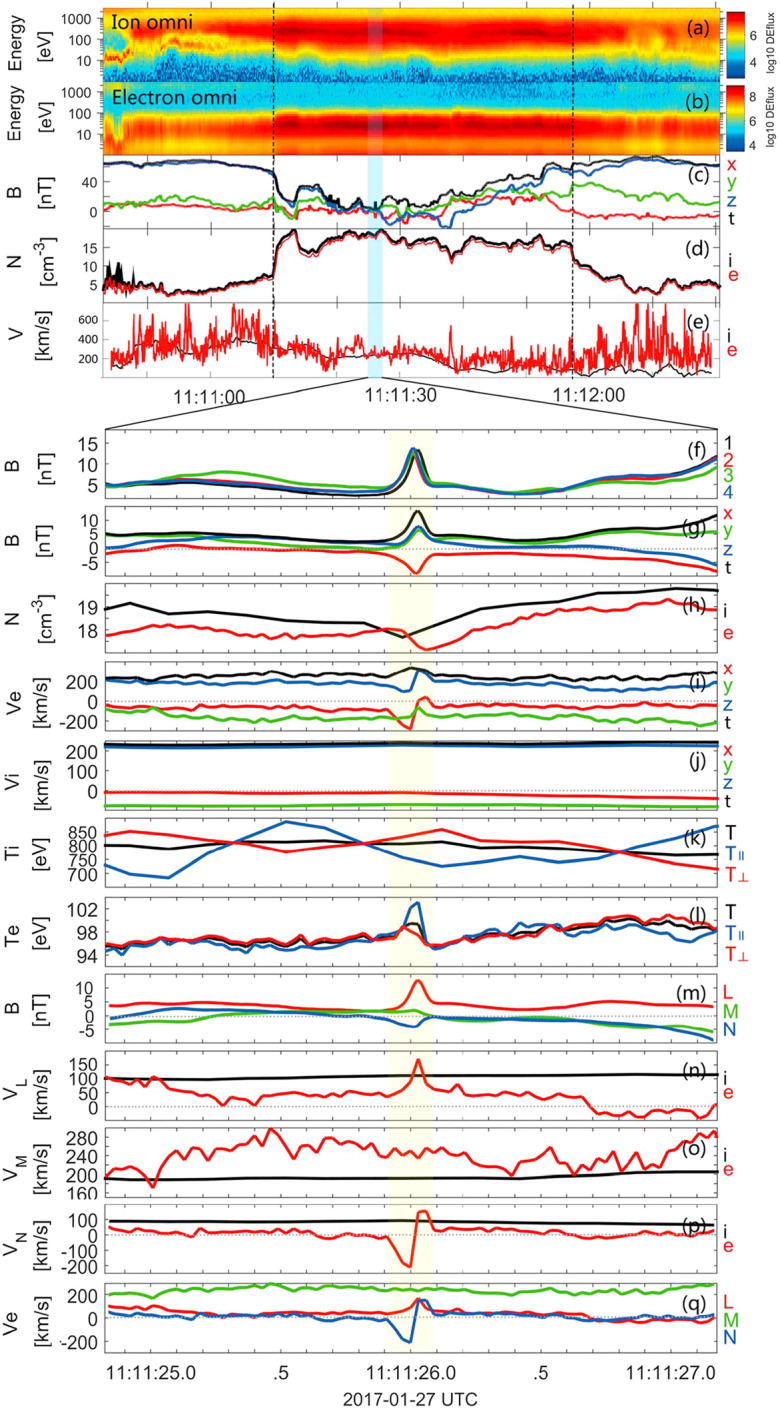
A kinetic scale magnetic peak observed by MMS in the magnetosheath. (**a**, **b**) Ion and electron differential energy fluxes (**c**) Magnetic field strength and components in GSE coordinates (**d**) Ion and electron number density (**e**) Ion and electron bulk velocity (**f**) Magneticfield magnitude of MMS1-MMS4. MMS1 observations of magneticfield and plasma data. (**g**) Magneticfield strength and components in GSE coordinates (**h**) Ion and electron number density (**i**, **j**) Ion and electron bulk flow velocity in the in GSE coordinates (**k**, **l**) Ion and electron temperature (**m**-**q**) Magnetic field and ion and electron bulk flow velocity in LMN coordinates. (From Yao et al. 2018a)

Before MMS, the mirror mode/magnetic dip/peak structures observed in the magnetosheath are on the MHD scale, from tens to thousands of proton gyroradius, corresponding to temporal scales of seconds to tens of minutes. Yao et al. (2018a) reported an electron scale magnetic peak observed by MMS in the Earth’s magnetosheath, with a scale of ∼7 electron gyroradii and a duration of ∼0.18 s (Fig. 36). An electron vortex is found in the plane perpendicular to the magnetic field line and is self-consistent with the magnetic peak. A technique was developed to distinguish flux ropes and mirror mode peaks which both have bipolar magnetic signatures. The analysis result shows that this small scale magnetic peak is not a flux rope which generally has a toroidal bipolar magnetic field in the plane perpendicular to the rope axis, but rather a magnetic bottle like structure which has a radial bipolar magnetic field in the plane perpendicular to the bottle axis. The mechanism generating the electron scale magnetic bottle like structure is still unclear (e.g., Treumann and Baumjohann 2018; Yao et al. 2019a) and new theories need to be developed to understand such small-scale phenomena.

**Fig. 37:**
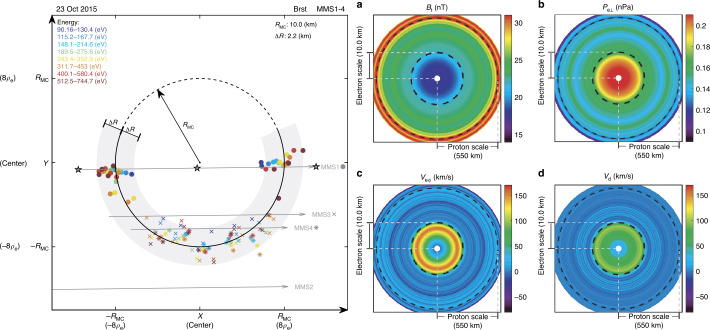
(Left) Cross-section of the electron scale magnetic cavity obtained using the sounding technique. (Right) The electron scale magnetic cavity is embedded in a proton scale magnetic cavity. (From Liu et al. 2019c)

Yao et al. (2017) presented a series of kinetic scale magnetic holes (KSMHs) observed by MMS in the magnetosheath, which exhibit diamagnetic electron vortices in the plane perpendicular to the magnetic field (see also Huang et al. 2017). The scale size of KSMHs is tens of electron gyroradii, similar to that of magnetic peaks. Liu et al. (2019c) reported a KSMH (which they called a magnetic cavity) embedded in a larger scale magnetic hole (Fig. 37). Using particle sounding technique on the KSMH, they found that it has a circular cross section and is a magnetic bottle in 3D.

KSMHs have been observed not only in the magnetosheath but also in the plasma sheet (Ge et al. 2011; Gershman et al. 2016; Sun et al. 2012; Sundberg et al. 2015; Yao et al. 2016; Zhang et al. 2017). KSMHs in the plasma sheet also have a spatial scale similar to that of magnetic peaks. Additionally, KSMHs in the plasma sheet have properties similar to that of magnetic peaks, i.e., electron vortices (Gershman et al. 2016; Zhang et al. 2017) and propagation relative to the plasma flow (Yao et al. 2016). Therefore, magnetic peaks and KSMHs could be physically related structures. Using a PIC simulation, Haynes et al. (2015) reported KSMHs within decaying turbulence showing an azimuthal diamagnetic current associated with the magnetic field depression. An Electron MagnetoHydroDynamics (EMHD) model of the slow-mode soliton has been developed to explain the formation of KSMHs (Ji et al. 2014; Li et al. 2016b), and the observed amplitude, size, and propagation velocity agree well with the theory of EMHD solitons (Yao et al. 2017). A detailed comparison among theories, simulations, and satellite observations is required to fully understand the physics of KSMHs.

**Fig. 38:**
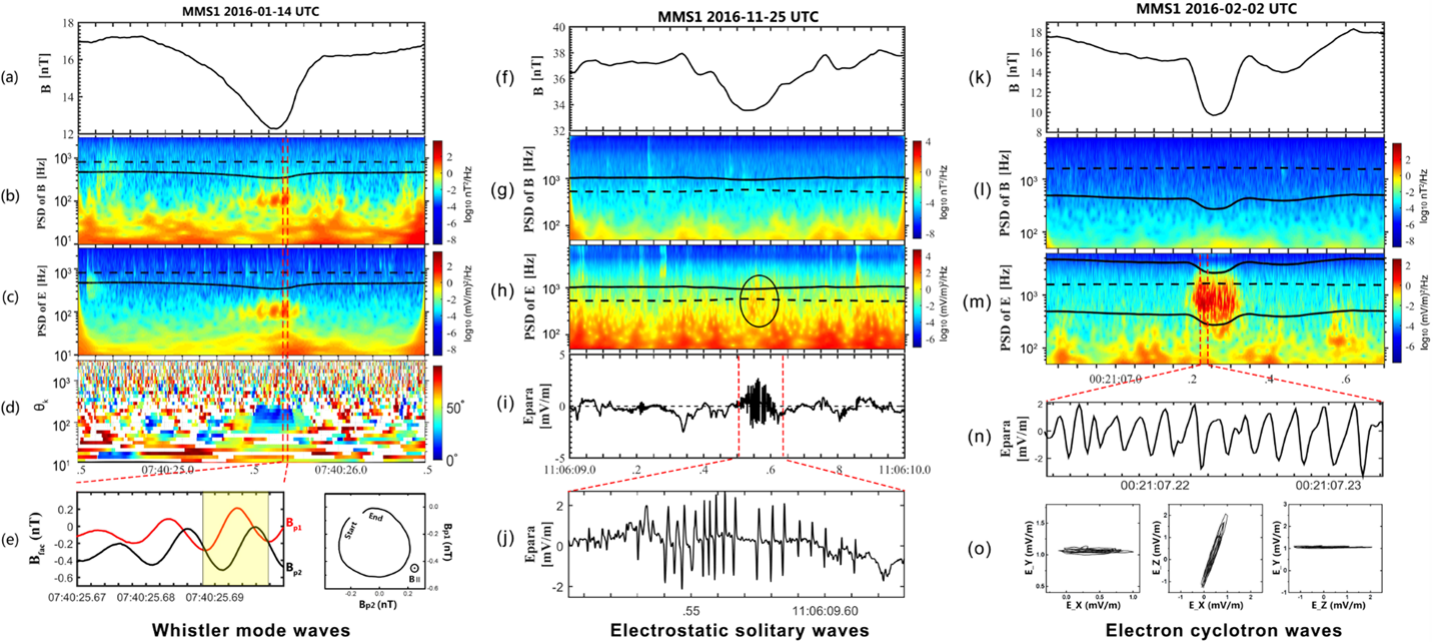
Three type of waves in kinetic scale magnetic holes in the magnetosheath. (**a**) Magnetic field strength (**b**, **c**) Power spectral densities of the electric and magnetic fields overplotted with the electron cyclotron frequency (solid line) and ion plasma frequency (dashed line) (**d**) Wave normal angle (**e**) Magnetic field in field-aligned coordinates and their hodograph during the time interval marked by two vertical dashed lines in above panels. BP1 and BP2 are the magnetic field in two perpendicular directions. (**f**-**h**) Same as (a-c) (**i**, **j**) Parallel electric field and an expanded view of the parallel electric field (**k**-**m**) Same as (a-c). The black solid lines in (**m**) indicate the electron cyclotron frequency and 3 times of the electron cyclotron frequency. The black dash line is the ion plasma frequency. (**n**) Parallel electric field (**o**) Hodographs of the electric field (From Yao et al. 2019b)

Such small structures have been found to contain a rich set of exciting physical processes, including different kinds of ion and electron distributions, electron or ion vortices, various types of waves, and even particle acceleration and declaration. For example, Yao et al. (2017) found that the flux of lower energy particles decreases in the center of the structure while the higher energy particle flux increases, indicating a non-adiabatic behavior. Liu et al. (2020f) found that these structures can shrink due to increases in the surrounding magnetic field strength and this shrinkage can induce an electric field. They suggested that this non-adiabatic behavior of the particles is related to the shrinking of the structure while propagating to the magnetopause where the magnetic pressure is higher. Qualitatively distinct from adiabatic acceleration mechanisms (e.g., betatron and Fermi acceleration), this process indicates a new type of non-adiabatic acceleration, and has been confirmed by the observed electron distributions and test particle simulations (Liu et al. 2020f). This discovery in space physics also has implications for understanding energy conversion in astrophysical plasmas, the origin of cosmic high-energy particles, and plasma turbulence. Large scale mirror mode structures in the magnetosheath often contain intense bursts of narrow-band whistler mode waves with a center frequency of ∼100 Hz, called “lion roar” (Smith et al. 1969; Smith and Tsurutani 1976; Giagkiozis et al. 2018), which are characteristic high-frequency waves in the magnetosheath. The growth of whistler waves generated in mirror mode structures was examined by PIC simulations (Ahmadi et al. 2016) and was further compared with MMS observations (Ahmadi et al. 2018). On kinetic scales, Yao et al. (2019b) presented observations of whistler mode waves, electrostatic solitary waves, and electron cyclotron waves inside KSMHs in the magnetosheath (Fig. 38). They suggested that the free energy to excite these waves comes from electron temperature anisotropy or beams in KSMH structures. Additionally, a higher wave occurrence rate at the center of the magnetic dips than at their edges indicates that these waves may originate from KSMHs. These observations suggest that electron scale magnetic peaks (Stawarz et al. 2018; Yao et al. 2020a) and KSMHs (Yao et al. 2019b, 2020b; Liu et al. 2020f) may play important roles in transporting particles and dissipating energy in turbulent plasmas.

### Transient Processes in the Foreshock, Bow Shock, and Magnetosheath at Other Planets

The transient phenomena discussed in the previous sections were discovered at the terrestrial bow shock, and some of them have also been observed at other planets. Table 4 summarizes the transient phenomena observed at various solar system bodies. Based on our current understanding of the formation mechanisms of these phenomena, we expect them to exist at all planets. ULF waves have been observed at Mercury (Russell 1989), Venus (Greenstadt 1986; Orlowski et al. 1995), Mars (Delva and Dubinin 1998), Jupiter (Khurana and Kivelson 1989), Saturn (Orlowski et al. 1995), comets Halley and Giacobini-Zinner (Le et al. 1989). SLAMS have been detected at Venus (Collinson et al. 2012b), Jupiter (Tsurutani et al. 1993), and comet Giacobini-Zinner (Tsurutani et al. 1993). Despite the known physical processes and the easy identification of these events, many fields in the rows of Table 4 for SLAMS, ULF waves, and mirror mode structures are still empty.

**Table 4:** Transient phenomena observed at various solar system bodies. “+”: observed phenomenon

	Mercury	Venus	Earth	Mars	Jupiter	Saturn	Comets
HFAs	+	+	+	+	+	+	
SHFAs		+	+	+		+	
Foreshock cavitons		+	+				
Foreshock compressional boundaries		+	+				
SLAMS		+	+		+		+
Foreshock bubbles			+				
Foreshock cavities		+	+	+			
Transient, local ion foreshock			+				
Density holes			+				
Magnetosheath jets/magnetosheath filamentary structures			+				
ULF waves	+	+	+	+	+	+	+
Mirror mode structures in the MSH/magnetic holes/peaks		+	+		+	+	

As mentioned in Sect. 2.3.1, HFAs have been observed at all planets in the solar system. Signatures of SHFAs have been recorded at Venus, Mars, and Saturn. It is important to note that these phenomena were observed at the bow shock of both magnetized and unmagnetized planets. Hence, these phenomena are universal. We expect the other transient events to be universal phenomena as well. Furthermore, the foreshock ULF waves, SLAMS, foreshock cavitons, and SHFAs are related phenomena (Schwartz and Burgess 1991; Kajdič et al. 2011; Omidi et al. 2013b). The foreshock ULF waves have been observed at all planets in the solar system. Hence, we expect the related events to exist at other planets too. Despite this expectation, there is no observation of density holes, foreshock bubbles, transient, local ion foreshock, foreshock cavitons, or foreshock compressional boundaries at other planets yet (Table 4). Fortunately, simulations can help to predict the occurrence pattern and properties of these structures at other planets. For example, Omidi et al. (2017b) performed hybrid simulations of the solar wind-Venus interaction and theoretically predicted the occurrence of foreshock cavitons and foreshock compressional boundaries.

**Fig. 39:**
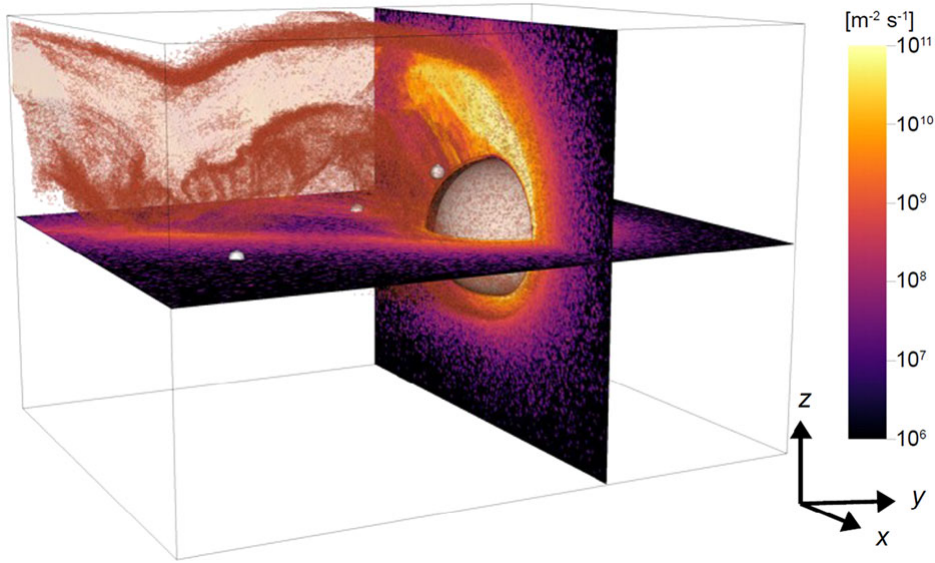
Escaping O^+^ ions from Venus modulated by the ULF waves. The color on the $z=0$ and on the $y=0$ planes show the oxygen bulk particle flux. The region where the particle flux exceeds $10^{9}$ m^−2^ s^−1^ is also shown by a partially transparent three-dimensional volume rendering. In the simulation the interplanetary magnetic field is [-8.09, 5.88, 0] nT and the solar wind flows to the $-x$ direction. (Jarvinen et al. 2020)

Venus and Mars provide another type of foreshock region because these planets do not have a notable global intrinsic magnetic field. These planets do not have intrinsic magnetospheres but, instead, the flow of the solar wind is stopped by the so-called magnetic barrier. In such, so called “Venus-like” interaction, a strong magnetic field region above the planets is filled with IMF which is “piled up” against the ionosphere (e.g., Luhmann et al. 2004). Therefore, the morphology of the magnetic field near Venus and Mars differs from the magnetized planets Mercury, Earth, Jupiter, and Saturn. Moreover, the bow shock at Mars and Venus is much closer to the planet than that at Earth (e.g., Slavin and Holzer 1981) and, consequently new planetary ions can be formed near the planet and at planetary foreshock from the hydrogen corona and hot oxygen corona (e.g., Lammer et al. 2008) forming a multi-ion species plasma. Despite these differences between magnetized and non-magnetized planets, the foreshock of the non-magnetized Venus seems to have many features similar to terrestrial foreshock according to 3D simulations, namely heated plasma and backstreaming ions (Jarvinen et al. 2020). Moreover, at Venus ULF waves cause periodic changes to the acceleration of escaping planetary ions (Fig. 39).

Bow shocks can form around comets too. However, it is difficult for foreshock transients to form due to the low strength of the shocks. In addition, due to their small sizes, usually there is not enough time to develop transient phenomena such as HFAs, SHFAs, FBs, foreshock cavitons, foreshock cavities, magnetosheath jets. Thus, the chance to observe these events seems to be low (Table 4). However, if these events were observed, their size would be small and their relative size compared with the comet would be large (Sect. 2.3.1). Hence, their influence on the cometary magnetosphere would be strong.

These phenomena very likely exist at all planets because their physical background is similar. Observations are needed to fill the empty slots in Table 4. Hence, Table 4 gives the future direction of research on the transient events. Studying these events at various planets and comparing their features with their terrestrial counterpart will dramatically improve our understanding of them. The following factors may affect the properties of the transients at different planets. a) Size of the bow shock. b) The spiral angle increases with heliocentric distance. A change in the spiral angle will change the foreshock geometry. c) The plasma $\beta $ is expected to maximize near Mars. d) The magnetosonic Mach number is expected to increase with heliocentric distance. Stronger shocks will result in stronger fluxes of backstreaming particles due to leakage of magnetosheath particles and reflection of solar wind particles (Russell et al. 2016).

### Outstanding Questions

About foreshock:

- Different scenarios have been proposed for the formation of GPB and FAB ion populations in the ion foreshock but one still ignores which processes may or may not be present simultaneously, which ones may be dominant and at what foreshock locations.
- 2D-PIC (global) simulations have allowed to recover simultaneously both ion and electron foreshocks and to analyze the local signatures of backstreaming particles functions but are limited to relatively short distances from the curved front and within the angular range ($90^{\circ } > \Theta _{Bn} > 45^{\circ }$) up to now. A further extension of the analysis, in particular to (partial or full) Q$_{\parallel }$ domain, should be quite instructive in order to analyze the coupling/continuity of the processes supporting these signatures between Q_⊥_ and Q$_{\parallel }$ shock for each population.

About ULF waves/shocklets/SLAMS structures:

Formation mechanisms and dynamics of ULF waves /shocklets /SLAMS have been mainly analyzed upstream of Q$_{\parallel }$ shock domain with the help of 1D and 2D (global and local) hybrid simulations, and a few 1D-PIC simulations. Several questions persist:

- What is the impact of the spatial scales of these structures versus the scales of shock rippling, as these hit the front?
- How do these sizes impact the formation and the survival time of magnetosheath jets/filamentary structures as these propagate within the downstream region?
- How does the persistence of ULF wave/shocklets/SLAMS vary upstream when approaching Q_⊥_ shock domain and how do their properties vary?

About transient phenomena at Earth’s foreshock, bow shock, and magnetosheath:

- How do foreshock cavitons interact with the magnetopause after entering the magnetosheath and cause magnetospheric and ionospheric disturbances? Foreshock cavitons have density and magnetic field perturbations, which are widely spread in the foreshock regions and may generate localized dynamic pressure and field perturbations. Understanding whether or how these perturbations affect the magnetosphere and ionosphere will be helpful for our understanding of the localized space environment variations.
- What mechanisms other than the mirror instability can generate magnetic holes and peaks in the magnetosheath? Solitary waves and the tearing mode instability (Balikhin et al. 2012) have also been proposed to contribute to the formation of magnetic holes, especially in mirror stable environments. In addition, the sub-ion scale structures cannot be directly generated from MHD-scale mirror instability. How they are generated and evolving is an outstanding question.
- Waves and particle generation in kinetic-scale magnetic holes. Various kinds of waves were found inside different scale magnetic holes. How they are generated inside or propagate into the structure needs further investigation.
- The mechanism to generate the electron scale magnetic bottle-like structures is still unclear, suggesting that a new theory needs to be developed to understand such small-scale phenomena. Are they generated from small scale solitary waves, electron mirror instability, or shrinking from larger scale mirror structures? Is there any new theory that can explain their formation? These questions raise new challenges in basic plasma physics.
- Downstream phenomena, such as FTEs generated by reconnection at the magnetopause, have been shown to temporarily deform the local bow shock. Such bow shock deformation could affect the upstream dynamics, e.g., by forming a transient foreshock. How downstream phenomena in return affect the upstream processes needs further investigation.
- Our current understanding of the formation mechanisms of foreshock transients is still qualitative and mostly limited to Earth’s bow shock environment. More efforts on quantifying and generalizing their formation processes are needed in order to predict their geoeffectiveness and investigate their existence, properties, and effects in various shock environments.

About foreshock phenomena at other planets:

- How do the strength of the planet’s intrinsic magnetic field and ionosphere affect its foreshock phenomena? This question arises already when magnetized planets Earth and Mercury are compared because weak Hermean magnetic field is permanently connected to the IMF. However, this question becomes more prominent when the foreshock of non-magnetized planets Venus and Mars are investigated because these planets do not have a traditional magnetosphere but their foreshock region can be magnetically connected to the planetary upper ionosphere.
- Can the dynamics in Venus and Mars foreshock propagate into the magnetic barriers and, finally, into the ionosphere along the draped magnetic field lines and what might be their ionosphere effects?
- When the non-magnetized planets Mars and Venus are considered, how do the planetary ions from the hot neutral corona and from the upper atmosphere affect the foreshock phenomena and vice versa?

## Transient Dayside Magnetopause Processes and Transport

### Surface Waves/Instabilities

Magnetopause surface waves can be generated by the solar wind dynamic pressure variations, transient phenomena near the bow shock, or the KHI on the magnetopause. The KHI can grow along the magnetopause because there exists a velocity shear between the anti-sunward streaming plasma in the magnetosheath and nearly stagnant magnetospheric plasma. The magnetopause KHI could play a key role in the transport of mass, energy, and momentum from the solar wind into the magnetosphere: the formation of the low-latitude boundary layer (Newell and Onsager 2003) and/or the cold-dense plasma sheet (e.g., Terasawa et al. 1997) often observed under northward IMF conditions, driving turbulent convection in the magnetosphere, the excitation of ULF waves in the inner magnetosphere, etc. Since extensive reviews on the magnetopause KHI have been given by Hasegawa (2012), Johnson et al. (2014), Faganello and Califano (2017), Masson and Nykyri (2018), here we only focus on recent progress on the magnetopause KHI. See, in particular, Johnson et al. (2014) for the fundamental physics underlying why and under what conditions the KHI can grow. We also point out that while spacecraft signatures of the KHI look transient because they are observable only when the boundary conditions are suitable for the KHI and spacecraft is near the KH unstable interface, the KHI itself is not necessarily a transient process. This is because KH surface waves or vortices can be generated continuously on the dayside magnetopause and advect tailward if the solar wind and magnetospheric conditions are stable and favorable for the KHI.

#### Recent Results on Large-Scale Properties

Kavosi and Raeder (2015) conducted a survey of the magnetopause KHI using seven years of data from the THEMIS spacecraft. They found that KH waves are present at the magnetopause approximately 19% of the time regardless of the solar wind conditions. They also showed that the occurrence rate of KH waves increases with the solar wind speed, Alfvén Mach number, and number density, but is mostly independent of the IMF magnitude. They also found that although the occurrence rate of KH waves under southward IMF is significantly higher than previously detected, it is still approximately four times less than the occurrence rate under northward IMF. Most of the events during southward IMF are irregular, short and polychromatic as compared to regular, long lasting and monochromatic waves under northward IMF.

Lin et al. (2014) showed, using a smaller number of KH wave events seen by THEMIS, that the KH wave period $T_{\mathrm{{KH}}}$ is shorter for higher solar wind speed $V_{\mathrm{{SW}}}$. This is a trend expected when the KH wavelength $\lambda _{\mathrm{{KH}}}$ is roughly constant because the advection speed of KH waves would be proportional to the solar wind speed, i.e., $T_{\mathrm{{KH}}} \propto \lambda _{\mathrm{{KH}}}/V_{\mathrm{{SW}}}$. They also found that the KH wave period/wavelength tends to be longer for larger IMF clock angles (smaller IMF $B_{z}$). This is a counterintuitive result because the latitudinal range where the KHI can be unstable would be narrower and thus the allowed maximum KH wavelength is smaller for larger clock angles (Farrugia et al. 1998). The puzzling result may be related to how the KHI or surface waves are excited for different IMF directions. We also note that the KH wave period is often not in the Pc 5 (>150 s) range (Fig. 6 in Lin et al. 2014) as often assumed in the literature on ULF waves, but in the Pc 4 (45-150 s) range.

KH waves have been observed in the mid-tail as well. Wang et al. (2014) reported bursty enhancements of hot (>0.5 keV) electrons observed by the ARTEMIS spacecraft in the tail magnetosheath at lunar distance, in association with quasi-periodic plasma and field fluctuations likely due to KH surface waves. Based on these observations, they suggested that the KHI can play a role in the loss of magnetospheric particles into interplanetary space. A similar study based on MMS data for the dayside magnetosheath has been conducted by Cohen et al. (2017) which found that quiet time events peaked strongly away from local noon on the morning and afternoon sides indicating KH waves as the probable cause. Based on global MHD and energetic test-particle computations, Sorathia et al. (2017) indeed found that energetic particles escape into the magnetosheath from both dawn and dusk sides of the magnetosphere, and the KHI enhances the losses, particularly for smaller gyroradius particles such as electrons.

Ling et al. (2018) reported simultaneous ARTEMIS and Geotail observations of KH waves in the mid-tail and the near-Earth region and suggested that the KH wavelength increases as they propagate along the boundary layer toward the tail. An increasing wavelength from the subsolar region has been predicted by Otto and Fairfield (2000) based on the increasing growth time for longer wavelength or coalescence, and by Li et al. (2012) in global simulations.

Walsh et al. (2015) showed observations of KH waves along the dayside magnetopause measured by THEMIS and Saskatoon HF radar. These observations occurred during a time period when a cold, dense plasmaspheric plume contacted the dayside magnetopause in the dusk sector. Theoretical calculations and observations indicate that the dense plume plasma lowered the KH unstable threshold and permitted the waves to form on a region of the magnetopause that is closer to the subsolar point than typically observed.

Identifying vortices from in-situ measurements is not a trivial task, although a number of methods to identify and/or analyze magnetopause vortices exist and were reviewed by Hasegawa (2012). Recent advances are testing and validating techniques to detect and characterize vortices, which are standard tools in the field of fluid dynamics but are not well known in the space physics community. Collado-Vega et al. (2018) applied one of such vortex identification algorithms (VIAs) to velocity data from global MHD simulations of the magnetosphere driven by real solar wind conditions. Cai et al. (2018) reviewed three VIAs and applied to magnetic field data taken by the four Cluster spacecraft during a KH vortex event at the duskside magnetopause (because not all four Cluster spacecraft made sufficiently accurate velocity measurements), and detected a roughly equal number of clockwise and counterclockwise magnetic field rotations.

#### Magnetic Reconnection Associated with KH Waves

KH waves have been largely associated with northward IMF because most observational evidence is for northward IMF. However, there is no fundamental reason that KH modes should not occur for southward IMF as well. In fact, Hwang et al. (2011) identified intermittent KH waves in a comparison of global simulations and Cluster observations. These findings were confirmed and specified by Yan et al. (2014) in THEMIS observations on the duskside flank. For southward IMF, both, magnetic reconnection and the KH instability can occur simultaneously at the Earth’s magnetopause. Ma et al. (2014a,b, 2016a) discussed the interaction of the KHI and reconnection for large magnetic shear. In particular, each strongly impacts the other, with the KHI limiting the reconnected flux and modifying the dissipation region structure. It is also demonstrated that the reconnection rate maximizes for conditions that allow a strong nonlinear evolution of KH waves, i.e., fast shear flow and limited guide magnetic field (field component along the X line). However, signatures can be reminiscent of FTEs such that an identification of KH waves for southward IMF can be more difficult.

The occurrence of KH waves is not limited to the vicinity of the equatorial plane. In comparison with global MHD simulations, Hwang et al. (2012) reported the first observations of KH waves at high latitudes during strongly dawnward IMF conditions. Similarly, based on mesoscale 3D simulations, Ma et al. (2016c) re-interpreted a high latitude event, which was originally thought to represent cusp reconnection, as a boundary modulation caused by a KH wave.

Originally, KH waves have only been associated with viscous transport (Miura 1984) and not with magnetic reconnection because they were regarded as ideal instabilities. For northward IMF, first in two dimensions it was found that for an inclined magnetic field across a boundary reconnection in the twisted magnetic field has to be expected (Otto and Fairfield 2000; Nykyri and Otto 2001; Nakamura and Fujimoto 2005). Subsequently, there have been many studies using different geometries, and plasma approximations to examine two and three-dimensional properties of this vortex reconnection. Nakamura et al. (2013) performed first three-dimensional (3-D) fully kinetic simulations of secondary reconnection induced as a consequence of the KHI. They demonstrated that in the presence of guide field a number of meso-scale magnetic flux ropes can be generated along current layers compressed and thinned around the periphery of the vortex, because secondary reconnection can occur at many flux surfaces in 3-D. These flux ropes are carried by the shear flow and are entrained into the vortex. In the final stage, merging of the vortices occurs and leads to the formation of new compressed current sheets and flux ropes.

For northward IMF and three dimensions, it was realized that twisting the magnetic field in a limited region close to the equatorial plane would generate strong current layers particularly at the southern and northern boundaries of the KH unstable region. Unless these vortices unwind, reconnection was required to separate the twisted part of the flux tube from the IMF. Consequently, pairs of magnetic reconnection sites have to form, allowing the exchange of part of magnetic flux tubes between the magnetosheath and magnetosphere and thus the capture of the magnetosheath plasma (Faganello et al. 2012; Takagi et al. 2006). More recently, Borgogno et al. (2015) examined this process in three-dimensional two fluid simulations. Ma et al. (2017) showed that this double mid-latitude reconnection for northward IMF can efficiently transport the plasma with a diffusion coefficient of $1\times 10^{10}$ m^2^ s^−1^. Note that this is considerably higher than estimated for reconnection within the KH vortex (Nykyri and Otto 2001). For IMF directions tilted from due north, the KHI and mid-latitude reconnection grows asymmetrically with respect to the equatorial plane and, interestingly, vortex-driven reconnection is limited to the region from the equatorial plane to mid-latitudes in the hemisphere that turns out to be less KH unstable (Fadanelli et al. 2018).

#### Results from the Magnetospheric Multiscale Mission

**Fig. 40:**
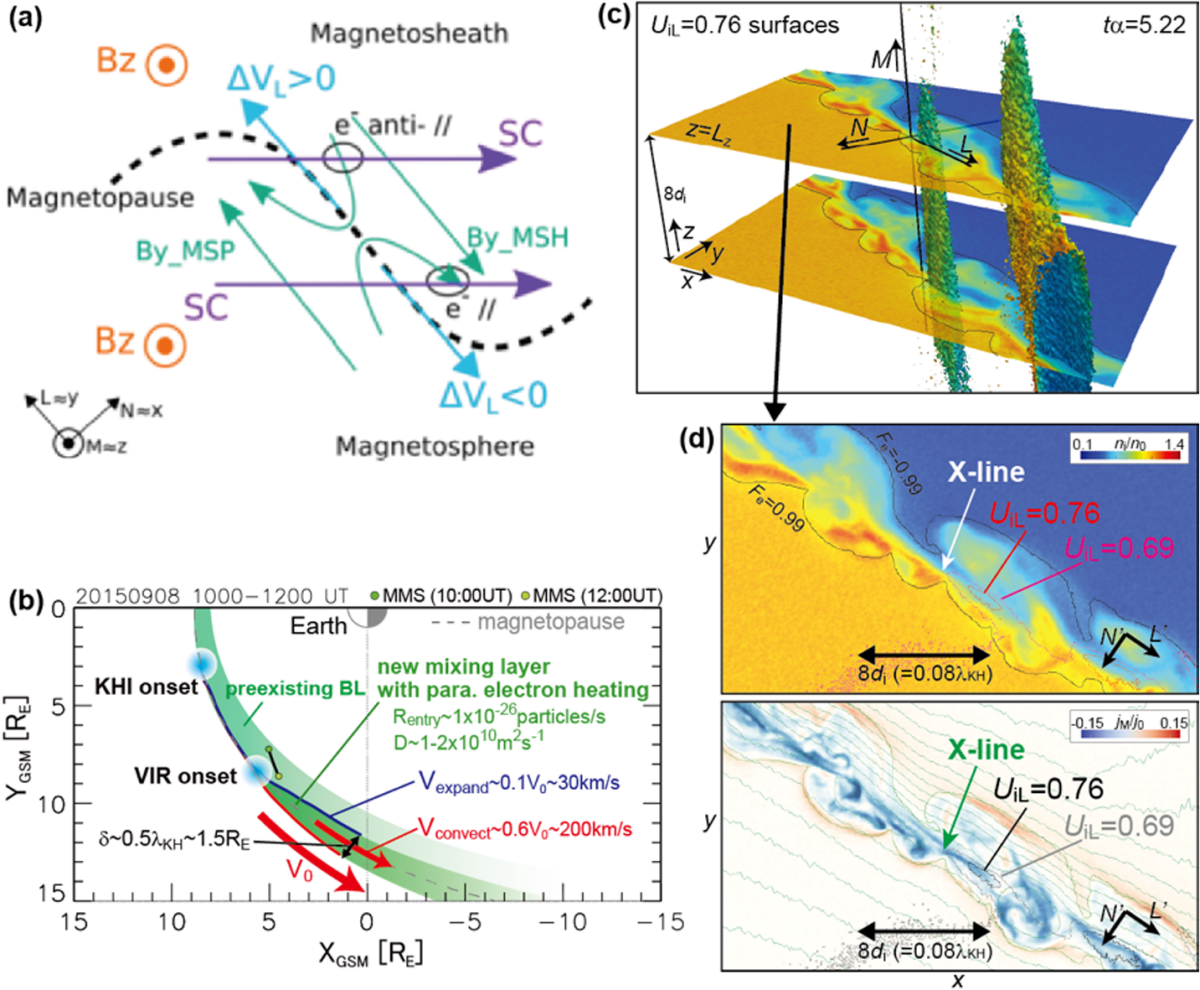
(**a**) Schematic of magnetic reconnection observed at the trailing (sunward) edge of a Kelvin-Helmholtz surface wave observed by MMS on 8 September 2015 (from Vernisse et al. 2016). Purple arrows show the direction of the spacecraft motion in the KH-wave rest frame. (**b**) The MMS location for 1000-1200 UT on 8 September 2015 when the KH vortices were detected, and parameters such as the particle entry rate R_entry_, diffusion coefficient D, and thickness $\delta $ of the mixing layer derived from comparison between MMS observations and three-dimensional (3-D) fully kinetic simulations of the KHI (Nakamura et al. 2017a). (**c**) 3-D view of ion-scale vortices formed at the KH wave trailing edge through vortex induced reconnection at the early nonlinear stage of the KHI (Nakamura et al. 2017b). 3-D surfaces show ion jets from the vortex induced reconnection with large velocity components in the $L$ direction. (**d**) Density and out-of-plane current density maps at $z=L_{z}$. The black curves in the top panel show the mixing surface defined as $F_{e}=((n_{1}-n_{2} ))/((n_{1}+n_{2} ) )= \pm 0.99$ where $n_{1}$ and $n_{2}$ are the initial electron densities on the magnetosheath and magnetospheric sides, respectively, with $n_{2}/n_{1} =0.3$. In the bottom panel, the black and gray curves show contours of the L component of the ion bulk velocity, and green lines show the in-plane magnetic field lines

Unambiguous evidence of magnetic reconnection driven by the KHI has been identified from the MMS mission, while earlier missions did not have plasma instruments with such high time resolutions that can dissect thin current sheets formed through the KHI. Eriksson et al. (2016) reported the first direct evidence of reconnection exhausts associated with KH waves at the postnoon magnetopause observed on 8 September 2015. The evidence consists of ion jets and Hall magnetic and electric fields in agreement with strong guide-field reconnection at the trailing surfaces of the KH waves. Using the same MMS event, Li et al. (2016a) reported kinetic evidence of vortex driven reconnection, such as low-energy (<100 eV) magnetosheath electrons entering the magnetosphere and magnetospheric electrons escaping into the magnetosheath through reconnected field lines. Vernisse et al. (2016) further investigated magnetic topologies of magnetosheath boundary layers observed outside the KHI-corrugated magnetopause surface (Fig. 40a), and found that particle leakage features are consistent with not only local vortex induced reconnection but also mid-latitude reconnection as simulated by Borgogno et al. (2015). These results demonstrate that the 3-D development of the KHI can induce plasma entry through reconnection at both mid-latitude and equatorial regions already sunward of the dawn-dusk terminator where the KHI is in its early nonlinear phase.

Nakamura et al. (2017b) conducted a large-scale fully kinetic 3-D simulation of the KHI specifically set for the KHI event observed by MMS on 8 September 2015, and found that ion-scale jets from vortex induced reconnection rapidly decay through self-generated 3-D turbulent reconnection. More efficient plasma transfer than previously thought can occur because the surface area of the interface between the magnetosheath and magnetosphere increases as a consequence of the formation of not only MHD-scale KH vortices but also ion-scale vortices resulting from vortex induced reconnection (Figs. 40c-d). Nakamura et al. (2017a) further compared the 3-D simulation and MMS observations and suggested that in addition to a pre-existing boundary layer produced by high-latitude reconnection, the KHI can form a new mixing layer with a thickness of ∼1.5 $R_{\mathrm{{E}}}$ near the terminator (Fig. 40b).

MMS observations have also revealed properties of turbulence and electrostatic waves presumably generated in association with the KHI. Stawarz et al. (2016) demonstrated that spectra of electromagnetic fluctuations and ion and electron velocities observed in the event on 8 September 2015 were all characterized by power law, consistent with developed turbulence despite the fact that the KHI was still in an early nonlinear phase. Wilder et al. (2016) reported large-amplitude (up to 100 mV/m), parallel, electrostatic waves in the middle of a KH vortex, whose excitation appears to require mixing of multiple particle populations including cold electrons with energies <10 eV.

### Flux Transfer Events

Flux transfer events (FTEs) observed by a spacecraft situated near the magnetopause are characterized by bipolar variations of the magnetic field component normal to the nominal magnetopause surface (Russell and Elphic 1978) and an enhancement of the field intensity at the center or a depression in the circumferential region in the case of a “crater” FTE (e.g., Sibeck et al. 2008). FTEs are believed to form through some form of time-dependent magnetic reconnection on the magnetopause and contribute to the transport of magnetic flux from the dayside magnetosphere into the magnetotail.

Various fairly idealized mechanisms have been proposed for the generation of FTEs (see Scholer (1995) and Raeder (2006) for reviews of FTE models), but they can be categorized into three types: localized and transient reconnection (Russell and Elphic 1978), extended but transient reconnection (Southwood et al. 1988; Scholer 1988), and multiple X line reconnection (Lee and Fu 1985), and multiple patches (Nishida 1989). The first two types are similar in that a symmetric pair of reconnected flux bundles are generated, one on each outflow side of the reconnection line. The difference lies in the size and/or dimensionality of the reconnected flux tubes; the first type produces a 3-D elbow-shaped flux tube with a relatively small azimuthal extent and a bend near the portion where the reconnected flux tube crosses the magnetopause from the magnetosheath to the magnetosphere (in the northern hemisphere) or vice versa (in the southern hemisphere). The second type produces an azimuthally extended bulge in the magnetopause and thus is essentially two-dimensional. The third type is unusual in its ability to create a single magnetic flux rope when only two X lines are present, so that there can be a hemispheric or dawn-dusk asymmetry in the occurrence frequency of FTEs if there is a preferred direction in which FTEs move. The last type is a distribution of different size reconnection patches and, in principle includes the other FTE models. A concise but helpful review of FTEs has been given by Paschmann et al. (2013), thus here we focus only on recent advances in FTE studies.

The different FTE reconnection models address mostly the question of in-situ FTE signatures but not issues such as the length of X lines or the total magnetic flux transport where ground observations provide further information (see Sect. 4.2.4).

As a variant of the multiple X line reconnection model for the FTE formation, sequential multiple X line reconnection (SMXR) was proposed by Raeder (2006) based on global MHD simulations of the solar wind-magnetosphere interaction under dominantly southward IMF. In their simulations, an initial X line forms in the magnetopause current layer on the winter hemisphere side of the point where the magnetosheath flows stagnate (see Hasegawa (2012) for a discussion about why there may be a tendency that the initial reconnection line forms on the winter hemisphere side). The X line then moves toward the winter hemisphere because there is a flow directed away from the stagnation point. Subsequently, a second X line forms in the region near the point of the initial X line formation, the result being the generation of a flux rope between the first and second X lines. The resultant flux rope further moves toward the winter hemisphere, and thus Raeder (2006) suggested that more FTEs would be encountered in the winter hemisphere. The above series of processes is repeated, thus called the SMXR model. Fear et al. (2012b) studied the seasonal control of the FTE location using hundreds of FTEs encountered by Cluster. They found that more FTEs tend to be observed in the winter hemisphere, although FTEs can occur even when the geomagnetic dipole tilt is negligibly small. Their results are consistent with results based on Interball-1 spacecraft observations of FTEs (Korotova et al. 2008), and partially support Raeder’s SMXR generation model.

Trattner et al. (2012) analyzed several cusp crossings to search for the signature of FTEs in the energy distribution of downward precipitating ions. They found several cusp events which show an energy overlap for parallel-streaming precipitating ions, that is consistent with field lines experiencing reconnection more than once. They suggested that this condition might be caused by reopening an already reconnected field line, forming a flux rope at the magnetopause.

Using a 2-D global hybrid-Vlasov simulation of the solar wind-magnetosphere interaction, Jarvinen et al. (2018) showed that fast-mode bow waves form ahead of poleward propagating FTEs in the magnetosheath. Their analysis demonstrates that protons can be energized up to 30 keV by a shock drift-like acceleration process and suggests that the resulting velocity distributions may account for energy-time dispersed ion injections observed by Cluster in the magnetosheath (Louarn et al. 2003).

Small spacecraft separations and unprecedentedly fast plasma measurements of the MMS mission have allowed in-depth investigations of the structure and evolution of not only typical FTEs (durations >1 min or diameters >1 $R_{\mathrm{{E}}}$) but also smaller ones. Hwang et al. (2016) analyzed one of the first FTEs encountered by MMS and revealed multi-layered structures and multiple field line topologies/connectivities of the FTE, consistent with but far more clearly than earlier observations (Pu et al. 2013; Varsani et al. 2014; Zhong et al. 2013). Zhao et al. (2016a) used magnetometer and fast plasma instrument measurements from the four MMS spacecraft to calculate the magnetic and plasma forces in FTEs. Their analysis shows that some but not all FTEs are force-free structures in which the magnetic pressure force balances the magnetic curvature force. Eastwood et al. (2016) reported MMS observations of two small-scale flux ropes with diameters of ∼10 ion inertial lengths, which are seen to be force-free and not growing. They suggested that the observed flux ropes are consistent with generation on the magnetopause by secondary magnetic reconnection in the vicinity of a preexisting, primary X line (e.g., Daughton et al. 2011); secondary reconnection can be another mechanism for the FTE generation. On the other hand, Dong et al. (2017) reported a series of ion-scale FTEs that appeared to be evolving to those of a typical size following the creation by secondary reconnection. Hasegawa et al. (2016) also showed, based on simultaneous Geotail and MMS observations at different latitudes, that meso-scale FTEs with durations less than 1 min (tens of ion inertial lengths) can decay in the course of poleward propagation during quasi-continuous, spatially extended magnetopause reconnection. Hwang et al. (2018) further suggested that ion-scale FTEs could be formed by the tearing instability in the reconnection exhaust region as a consequence of the elongation of the current sheet when two X lines are being separated in the jet direction.

**Fig. 41:**
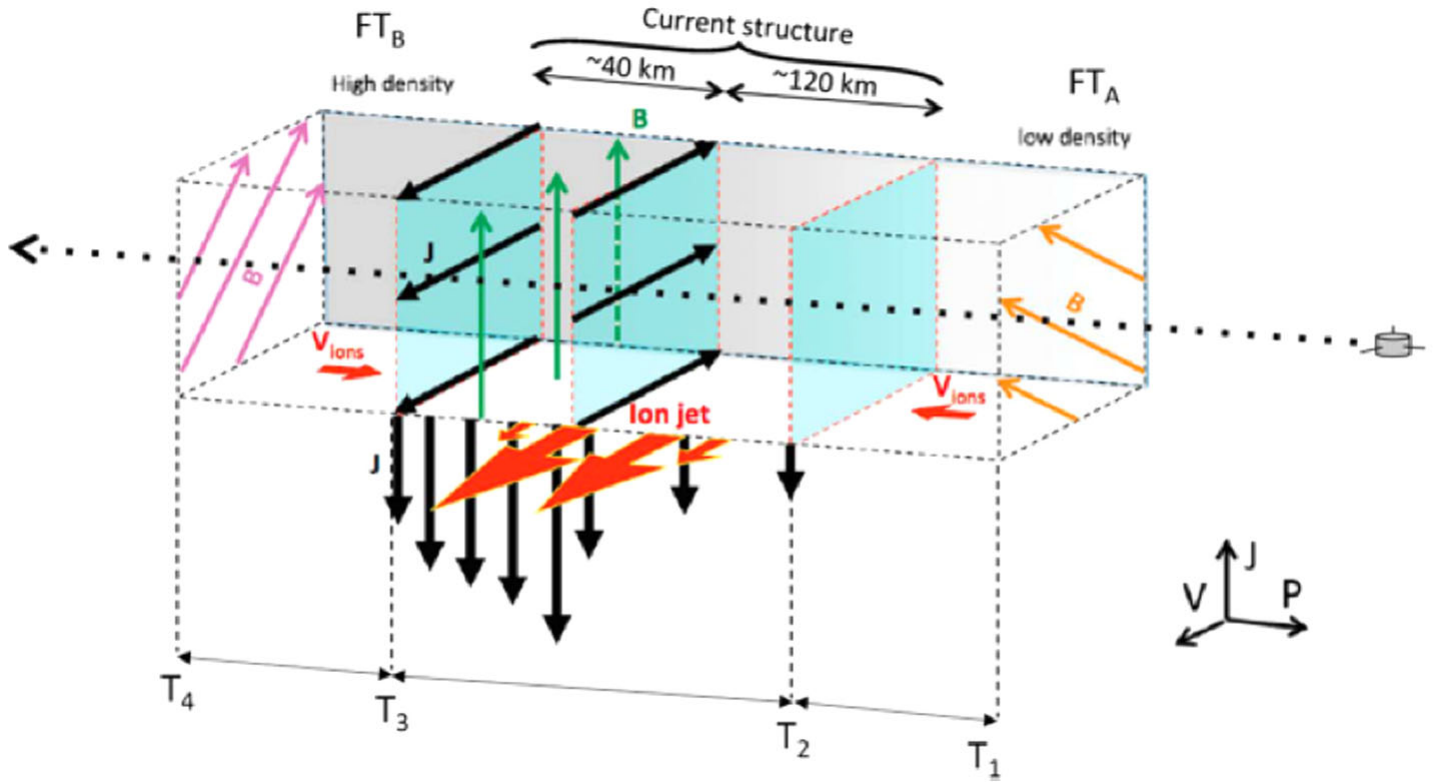
Schematic of a thin current sheet observed by MMS between two interlaced magnetic flux tubes (FTA and FTB) constituting an event that looks like a flux transfer event. An ion jet detected inside the current sheet is consistent with magnetic reconnection likely driven by flows toward the current sheet (red arrows in FTA and FTB). The P, J, and V axes are along the propagation direction of the structure, anti-parallel to the electric current in the current sheet, and along the reconnection jet, respectively. (From Kacem et al. 2018)

MMS observations also detected magnetic reconnection in a current sheet formed through the interaction of different flux tubes constituting an FTE or FTE-like structure. Such an interaction can occur when oppositely directed reconnection jets from multiple X lines collide at the center of a flux rope (Nishida 1989; Øieroset et al. 2014; Otto 1995) or when reconnected flux tubes collide with those preexisting in the current sheet with a guide field. Øieroset et al. (2016) reported the first direct evidence of guide field reconnection in a thin (2.5 ion inertial lengths) current sheet at the center of a magnetopause flux rope identified by MMS. Kacem et al. (2018) analyzed another MMS event observed in the vicinity of the magnetopause that, at first glance, appears to be a typical FTE, and found that it consisted of two distinct flux tubes with different magnetic topologies or connectivities that were separated by an ion-scale reconnecting current sheet at its center (Fig. 41). Such complex magnetic structures indicate that the three standard FTE generation mechanisms cannot simply account for the event, and suggest a 3-D interaction of reconnected field lines in the magnetopause boundary layer, as simulated by Cardoso et al. (2013) and Farinas Perez et al. (2018). Note that magnetic structures suggestive of such a 3-D interaction in the magnetopause have been reported earlier by Louarn et al. (2004) and Hasegawa et al. (2015) based on Cluster and THEMIS observations, respectively, and have been predicted by theory and simulation (Nishida and Maezawa 1971; Nishida 1989; Otto 1995). Øieroset et al. (2019) further analyzed a total of four such reconnection events in the current sheet of 3-D interlaced flux tubes, and suggested that magnetic pile up upstream of the reconnecting current sheet leads to enhanced magnetic shear and decrease in $\beta $ jump across the current sheet, both being the conditions favorable for reconnection.

The MMS mission would also be able to identify coalescence of magnetic flux ropes, but unambiguous evidence of such a process at the magnetopause remains to be reported, while Wang et al. (2016) showed Cluster observations consistent with magnetic islands coalescing in the magnetotail. In fact, Zhou et al. (2017a) showed MMS observations near the subsolar magnetopause of an FTE that they claimed are consistent with the coalescence of macroscopic flux ropes. However, the reported reconnecting current sheet was observed in a region where the GSM $B_{z}$ component was significantly positive, suggesting that the spacecraft were not at the center of the magnetopause current sheet, that is the expected location of the coalescence, but rather on the magnetospheric side. It is possible that what they reported are signatures of secondary reconnection, as simulated by Daughton et al. (2011), that can occur in regions somewhat away from the center of the current sheet where there exists weak but finite magnetic shear in the presence of significant guide field.

### Effects of Impulsive Events at the Magnetopause

#### Foreshock/Bow Shock/Magnetosheath Transients and Magnetopause Surface Waves

Foreshock transients, including HFAs, SHFAs, FBs, foreshock cavitons, and foreshock cavities, have a core with very low dynamic pressure bounded by one or two boundaries with enhanced dynamic pressure. Therefore, as they contact the bow shock, the bow shock together with the magnetopause can locally deform. For example, using multi-spacecraft observations, Sibeck et al. (1999) found that the magnetopause and magnetospheric plasma moved outward into an HFA across the original magnetosheath. Using data from multiple THEMIS spacecraft, Jacobsen et al. (2009) showed that due to an HFA the magnetopause deformed with an inward bulge and an outward bulge, which were propagating tailward with the HFA. Later, Archer et al. (2015) reported magnetopause outward motion driven by an FB. Based on observations, Archer et al. (2014) found that the pressure gradient in the magnetosheath due to a foreshock transient can accelerate magnetosheath plasma causing fast magnetosheath flow, which can approximately keep the deformed magnetopause bulge in pressure balance.

**Fig. 42:**
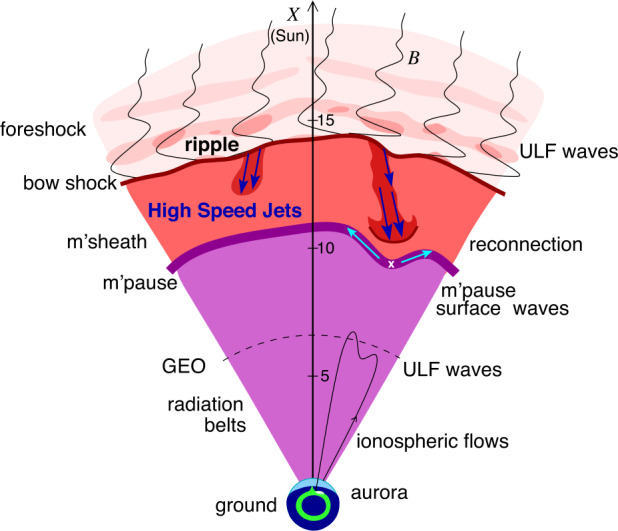
Sketch illustrating magnetosheath high-speed jets which originate from the bow shock and their possible effects (Plaschke et al. 2018a)

As illustrated in Fig. 42, magnetosheath jets with high dynamic pressure can penetrate deep into the magnetosheath and impinge on the magnetopause. Since magnetosheath jets are local, transient structures with typical scale sizes of 1.34 $R_{\mathrm{{E}}}$ (azimuthal) and 0.71 $R_{\mathrm{{E}}}$ (radial direction) (Plaschke et al. 2016, 2018a), localized boundary indentations with large amplitudes can be produced on the magnetopause (Shue et al. 2009).

**Fig. 43:**
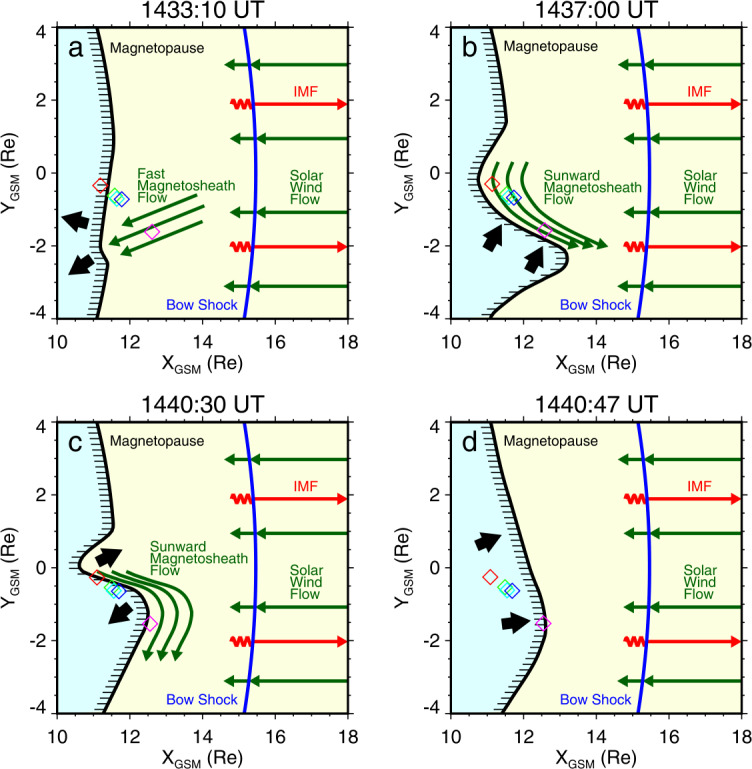
Schematic diagrams for a magnetosheath jet and distorted magnetopause observed by different THEMIS probes (colored diamonds). The black arrows indicate the directions of the magnetopause motion. (**a**) The small-scale, magnetosheath jets (fast anti-sunward magnetosheath flow). (**b**-**c**) The magnetopause is distorted locally. (**d**) The distortion is relaxing gradually. (Shue et al. 2009)

The schematic diagrams in Fig. 43 illustrate the magnetopause distortion due to a magnetosheath jet. A magnetosheath jet generated an initial disturbance on the magnetopause (Fig. 43a). The distortion expanded in both azimuthal and radial directions with a maximum scale size of 2 $R_{\mathrm{{E}}}$ wide and 1 $R_{\mathrm{{E}}}$ deep (Fig. 43b). The flow direction of the magnetosheath jet can be reversed to sunward by the magnetopause rebound. After the flow direction change, the magnetopause gradually returned to the normal shape (Figs. 43c, d).

The dayside magnetopause can excite and trap surface waves with its own eigenmode impulsively. The magnetopause surface waves can be a standing wave pattern that is formed by the interference of surface waves propagating along the magnetospheric magnetic field lines and reflecting in the northern and southern ionospheres (Plaschke et al. 2009b,a; Plaschke and Glassmeier 2011; Archer and Plaschke 2015). The radial oscillation of the Earth’s magnetopause field lines can further excite ULF waves predominantly with distinct, often called “magic”, frequencies in the magnetosphere (Plaschke and Glassmeier 2011; Archer et al. 2013).

Hartinger et al. (2015) investigated the magnetospheric response to a solar wind dynamic pressure pulse using BATS-R-US global MHD simulations. The simulation results show persistent monochromatic magnetopause surface eigenmodes at a frequency of 1.8 mHz, implying that magnetopause surface eigenmodes can be a potential source of monochromatic compressional ULF waves below 2 mHz in the dayside magnetosphere and provide a seed perturbation for the tailward propagating surface waves via the KHI.

ULF waves in the magnetosphere are often observed on the ground with persistent discrete frequencies (around 0.7, 1.3, 1.9, 2.6, 3.3, and 4.8 mHz) known as “magic” frequencies (Menk 2011). Different from other standing ULF waves in the magnetosphere, there is no eigen-frequency gradient for the magnetopause standing waves (Plaschke and Glassmeier 2011).

**Fig. 44:**
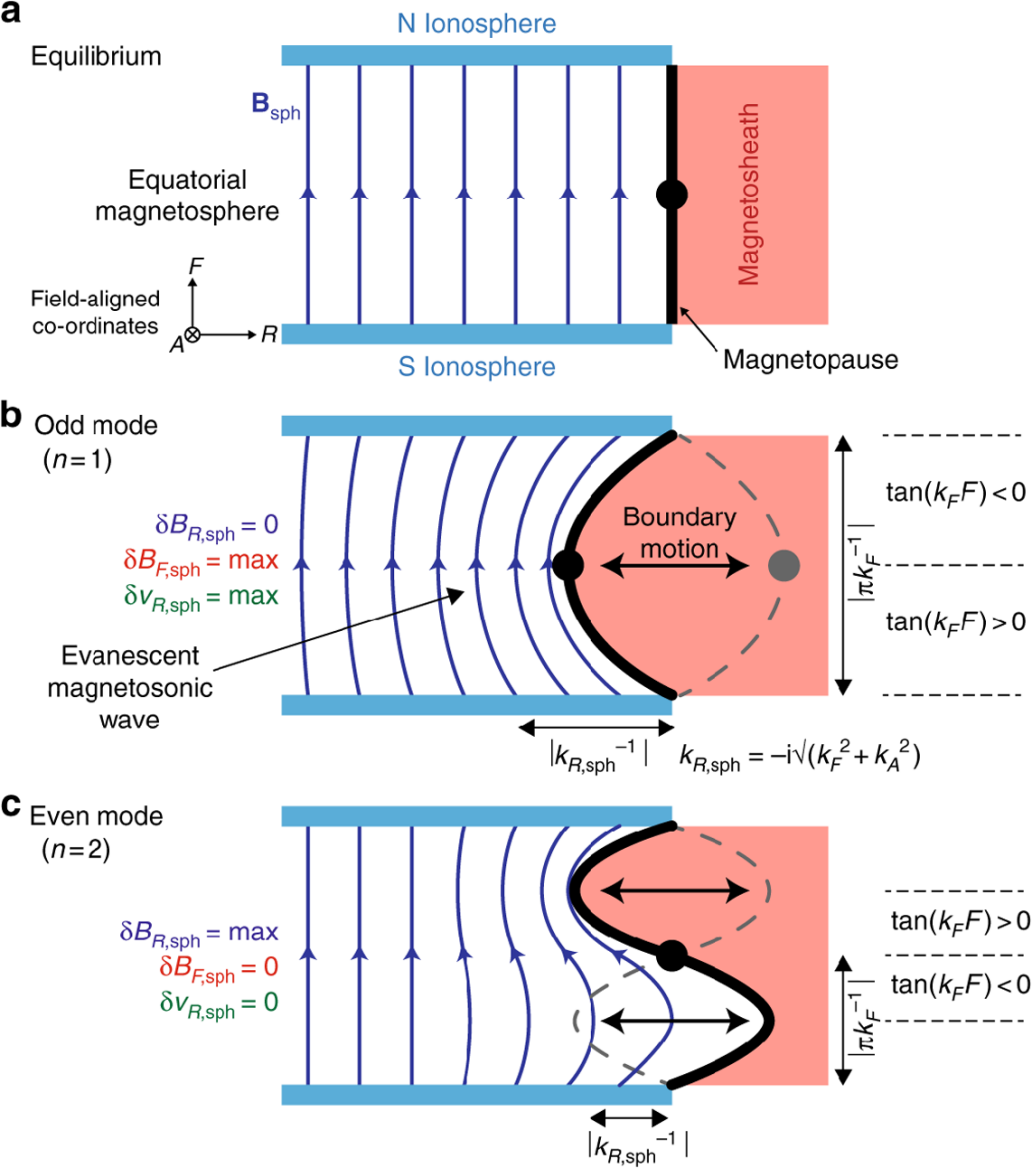
Diagram of the magnetopause surface eigenmode in a box model. (**a**) Box model equilibrium showing the magnetopause (black) which separates the magnetosheath (red) and the magnetosphere. Dark blue arrows denote the magnetic field lines between the northern and southern ionospheres (colored light blue). (**b**) $n=1$ (fundamental mode) (**c**) $n=2$ (second harmonic mode). The midpoint of the phase is denoted by the black dot, corresponding to the location of the magnetopause surface eigenfrequency $n=1$ antinode and $n=2$ node. (Archer et al. 2019)

There are many possible drivers for magnetopause surface eigenmodes including interplanetary shocks, both positive or negative solar wind dynamic pressure pulses, and magnetosheath jets. Using a simplified box model shown in Fig. 44, Archer et al. (2019) suggested that the magnetopause surface eigen-frequency should be around 1.7, 1.8 and 3.3 mHz for $n=1$ (fundamental mode) and $n=2$ (second harmonic mode) respectively. In addition, they provided direct observations of magnetopause eigenmodes during a magnetosheath jet event. These observations confirm that the dayside magnetopause can trap surface waves with its own eigen-frequency.

#### Mode Conversion at the Magnetoapause

*Introduction*

Wave perturbations generated from the bow shock, e.g., the foreshock of the quasi-parallel shock, can propagate to the magnetopause, which in turn excite shear Alfvén waves in the magnetosphere through a wave mode conversion process in the magnetopause boundary layer (Lee et al. 1994; Johnson and Cheng 1997), in which the incoming compressional mode waves are converted to shear Alfvén mode waves through excitation of kinetic Alfvén waves (KAWs) (Hasegawa and Mima 1978; Lee et al. 1994; Johnson and Cheng 1997; Chen 1999; Johnson et al. 2001; Chaston et al. 2008; Lin et al. 2010). The KAWs excited at the magnetopause may cause a cross-field diffusive transport across the magnetopause, since the waves carry kinetic-scale electric and magnetic field variations that can break the frozen-in condition (Lee et al. 1994). Such mode conversion may, therefore, facilitate effective processes for plasma heating and transport at the magnetopause (Hasegawa and Mima 1978; Johnson and Cheng 1997; Chen 1999; Johnson and Cheng 2001; Chaston et al. 2008).

For a closed dayside magnetopause (under a northward IMF), wave-particle diffusive processes in large-amplitude ULF waves, as frequently observed at the magnetopause boundary, provide a source for plasma entry from the solar wind (Anderson et al. 1982; Tsurutani et al. 1982; Rezeau et al. 1986; LaBelle and Treumann 1988; Rezeau et al. 1989; Engebretson et al. 1991b,a; Lin et al. 1991; Takahashi et al. 1991; Rezeau et al. 1993; Song et al. 1993a,b,c; Anderson et al. 1994; Song 1994; Song et al. 1994), in addition to the mechanisms of transport through high-latitude reconnection (Song and Russell 1992; Raeder et al. 1997; Le et al. 1996; Lavraud et al. 2006b) and Kelvin-Helmholtz vortices. Near the magnetopause boundary, a sharp transition is frequently found in wave polarization from predominantly compressional waves in the magnetosheath (e.g., due to foreshock waves of the quasi-parallel bow shock) to transverse in the boundary layer (Song et al. 1993a; Rezeau et al. 1989; Chaston et al. 2008). It has been suggested that the mode conversion from compressional to Alfvén modes provides a source of Alfvén waves at the magnetopause (Lee et al. 1994; Johnson and Cheng 1997), which can efficiently transport plasma across the magnetopause boundary (Hasegawa and Mima 1978; Lee et al. 1994; Johnson and Cheng 1997; Chen 1999; Johnson et al. 2001; Chaston et al. 2008). Multipoint measurements have verified that the dispersion of the broadband waves is consistent with the kinetic Alfvén waves (Chaston et al. 2007, 2008). Numerical simulations have been performed to investigate such mode conversion process (Lin et al. 2010, 2012; Shi et al. 2013, 2017a), including the subsequent generation of field line resonance (FLR) associated with toroidal Alfvén waves in the dayside magnetosphere (Shi et al. 2021).

In this section, we present a review of the theoretical studies, numerical simulations, and space observations of the mode conversion process.

*Theoretical studies*

Based on the MHD linear dispersion relation in a uniform plasma, there are three fundamental modes, including the fast/slow magnetosonic mode and the shear Alfvén mode (intermediate mode). In a nonuniform plasma, however, the MHD modes are coupled. In a plasma with a one-dimensional (1-D) inhomogeneity along the direction perpendicular to the background magnetic field, the linearized MHD equations can be expressed in terms of $\delta p_{1} = B_{0} \delta B_{\parallel }+ \delta p$ and $\delta B_{\perp }$ by a coupled system of equations (Lin et al. 2010),

3 $$ - i k_{\parallel }\left (1 - \frac{k_{\parallel }^{2} C_{s}^{2} }{\omega ^{2}} \right ) \delta p_{1} = \left (1 + \frac{C_{s}^{2} }{V_{\mathrm{{A}}}^{2}} - \frac{k_{\parallel }^{2} C_{s}^{2} }{\omega ^{2}} \right ) B_{0}^{2} \nabla _{\perp }\cdot \left (\frac{ \delta {\mathbf{B}}_{\perp }}{B_{0} }\right ), $$

and

4 $$ (\omega ^{2} - k_{\parallel }^{2} V_{\mathrm{{A}}}^{2} ) B_{0} \delta {\mathbf{B}}_{\perp }= i k_{\parallel }V_{\mathrm{{A}}}^{2} \nabla _{\perp }\delta p_{1}, $$

with $\omega $ being the wave frequency, $V_{\mathrm{{A}}}$ the Alfvén speed, and $k_{\parallel }$ the parallel wave number. Singular behavior occurs at either the Alfvén resonance (Tamao 1965; Uberoi 1972; Chen and Hasegawa 1974; Southwood 1974; Hasegawa et al. 1983), $\omega ^{2} = k_{\parallel }^{2} V_{\mathrm{{A}}}^{2}$, or at the sound resonance, where the compressional wave couples with the Alfvén or sound wave. In higher frequency cases, the Alfvén resonance condition is modified as $\omega ^{2} = k_{\parallel }^{2} V_{\mathrm{{A}}}^{2}(1-\omega ^{2}/\Omega _{i}^{2})^{2}$ due to the finite ion Larmor radius effects (Stix 1992), where $\Omega _{i}$ is the ion gyrofrequency. The Alfvén resonance singularity can be removed by including non-MHD effects such as electron inertia or ion Larmor radius corrections in Eq. (4). Near the magnetopause, electron inertial effects are typically not important.

This Alfvén resonance singularity can be removed by including kinetic effects (Chen and Hasegawa 1974; Southwood 1974; Hasegawa and Chen 1976a,b; Johnson and Cheng 1997; Johnson et al. 2001; Chen 2008; Lin et al. 2010), and the wave coupling is through the mode conversion to kinetic Alfvén waves (Hasegawa and Mima 1978; Hasegawa and Chen 1976a,b; Lee et al. 1994; Johnson and Cheng 1997) for cases with an electron $\beta _{e} > m_{e}/m_{i}$. As a result, mode conversion occurs where the frequency matches the continuous spectrum. For magnetospheric plasma, the KAWs are low frequency ($\omega < \omega _{i}$, where $\omega _{i}$ is the ion cyclotron frequency) Alfvén mode with $k_{\perp }\rho _{i} \sim k_{\perp }\rho _{s} \sim 1$, where $\rho _{i}$ is the ion Larmor radius, $\rho _{s} = \sqrt{T_{e}/T_{i}} \rho _{i}$, $T_{e}$ and $T_{i}$ are the electron and ion temperatures, respectively, and $k_{\perp }$ is the perpendicular wave number. On the short wavelength scale, ion motion decouples from the electron motion. KAWs can develop parallel electric fields $E_{\parallel }$ through charge separation, which facilitates particle heating and transport on scales where the ion motion decouples from the field lines due to polarization drift (Johnson and Cheng 1997).

Typically, the Alfvén velocity across the magnetopause increases by a factor of 10 such that an entire decade (in frequency) of wave power can be captured by the mode conversion process and localized in the boundary layer leading to massive particle transport. At the Alfvén resonance point, transversely polarized waves are expected to be generated and radiate away from the mode conversion location.

Analytical linear theories have been derived for mode conversion at the magnetopause (Johnson and Cheng 1997; Johnson et al. 2001) based on a kinetic-MHD model, which uses Ohm’s law and the momentum equations obtained from the gyrokinetic equations. Based on the quasilinear theory, the diffusion coefficient for the KAWs under typical magnetopause conditions is on the order of $D_{\perp }\sim 10^{9-10} m^{2}/s$ (Johnson and Cheng 1997). Theoretically, ions may be nonlinearly heated by resonance between the polarization drift and gyro-motion above a threshold wave amplitude (Johnson et al. 2001; Chen et al. 2001). The effects of IMF orientation have been calculated by Johnson and Cheng (1997). While the KAWs radiate back into the magnetosheath from the Alfvén resonance location under a northward IMF, they can propagate through the $k_{\parallel }=0$ location due to the existence of magnetic shear, which may lead to magnetic islands (Johnson and Cheng 1997).

Johnson et al. (2001) carried out a theoretical investigation of ion motion in the presence of large-amplitude KAWs with wavelength on the order of $\rho _{i}$ and demonstrated that for sufficiently large wave amplitude the ion orbits become stochastic. As a result, low energy particles in the core of the ion distribution can migrate to higher energy through the stochastic sea leading to an increase in the perpendicular ion temperature and a broadening of the distribution.

*Observations of mode conversion and associated KAWs*

Large-amplitude Alfvén waves have been observed around the magnetopause (Anderson et al. 1982; Song 1994). Cluster multipoint measurements have found the dispersion of broadband waves consistent with KAWs (Chaston et al. 2007). The deposition of energy into the auroral ionosphere from KAWs in the cusp and low-latitude boundary layer has been reported (Chaston et al. 2005), which results in precipitating electron fluxes sufficient to drive bright aurora and cause outflows of energized electrons and O^+^ ions from the ionosphere.

Kinetic Alfvén waves also provide a natural explanation for the observed dawn-dusk asymmetry in plasma entry during northward IMF (Hasegawa et al. 2003; Wing et al. 2005) because they result from mode conversion of compressional foreshock waves, which typically bathe the dawn flank for the typical Parker spiral configuration. The nonlinear development of the magnetopause spectrum observed by Cluster (Chaston et al. 2007) and THEMIS (Chaston et al. 2008; Yao et al. 2011) has shown intriguing features. The global distribution indeed indicates that the spectral energy densities across the magnetopause are larger on the dawn than dusk side (Yao et al. 2011). The THEMIS observations (Chaston et al. 2008) show direct evidence of a turbulent spectrum of KAWs at the magnetopause with sufficient power to provide massive particle transport. The diffusion coefficients based on the observed spectra are about $D_{\perp }\sim 10^{9-10} m^{2}/s$, consistent with the estimates of Johnson and Cheng (1997) based on the quasilinear theory. By examining the global distribution and properties of kinetic-scale magnetopause waves observed by THEMIS, Yao et al. (2011) have shown that the KAW wave activity increases with the magnetic shear across the magnetopause, under both southward and northward IMF. Such observation is consistent with the quasi-linear theory by Johnson and Cheng (1997), although it is still unclear whether the observed electromagnetic fluctuations are resulting from KAWs due to mode conversion or KAWs that are generated by magnetic reconnection (Chaston et al. 2009; Gershman et al. 2017).

Heated ions are commonly observed at the magnetopause in conjunction with waves that satisfy the threshold condition for chaotic ion heating (Chaston et al. 2008). THEMIS events show strong evidence of perpendicular heating of the magnetosheath ions through interaction with the KAWs near the dayside and flank magnetopause (Chaston et al. 2014). The wave spectra ($B^{2}$) associated with KAWs at the magnetopause typically exhibits a breakpoint around the ion gyroradius (Chaston et al. 2008), where the spectrum becomes more electrostatic.

*Local and global simulations of mode conversion and associated KAWs*

**Fig. 45:**
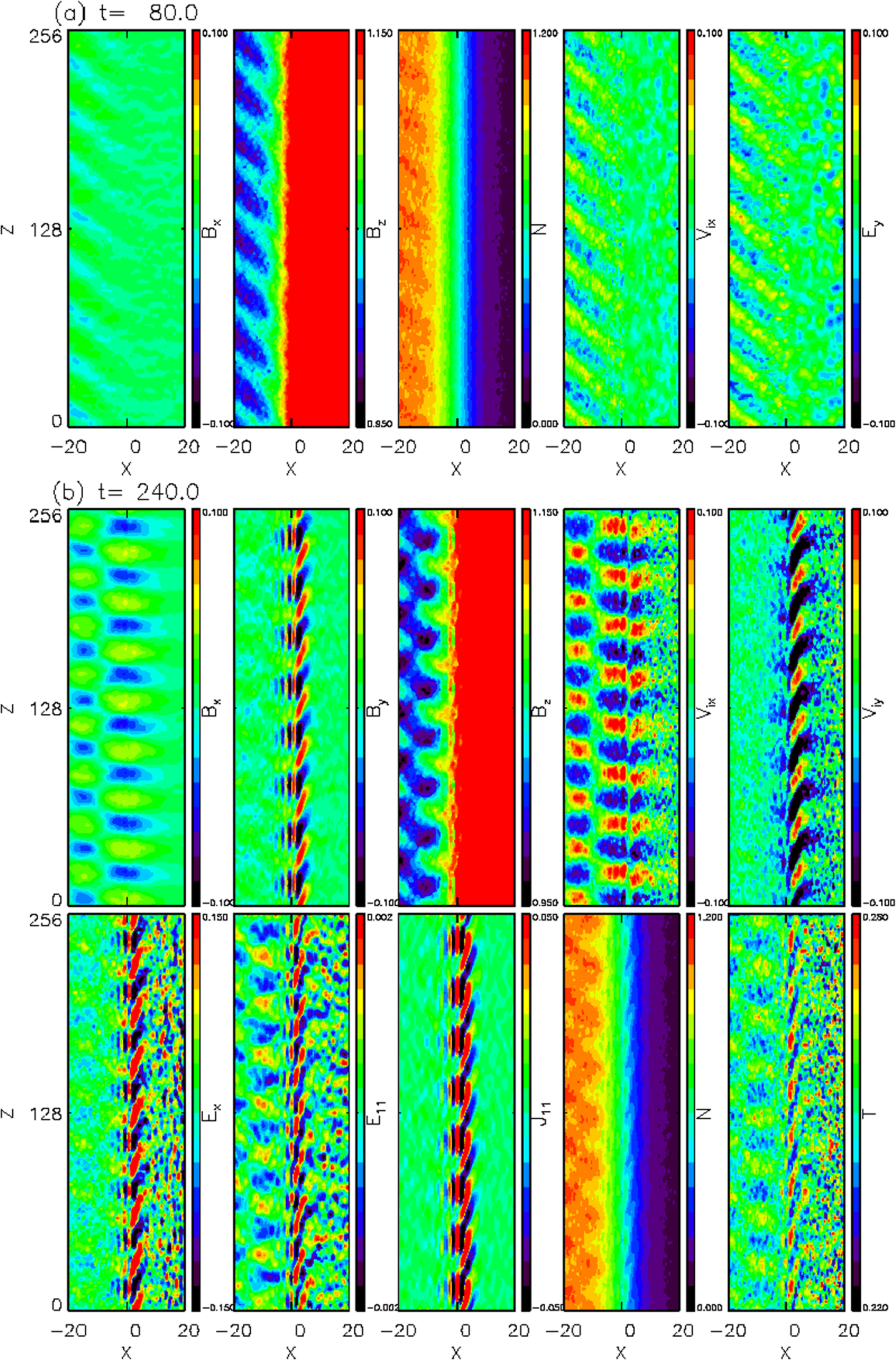
Contours of various quantities around the magnetopause boundary layer obtained from hybrid simulation in slab geometry at (**a**) an early time $t = 80$ and (**b**) $t = 240$. Short wavelength KAWs are generated by interaction between the incident wave and the magnetopause. (Lin et al. 2010)

The mode conversion process and associated KAWs have been tackled with kinetic simulations. Lin et al. (2010) performed a 2-D hybrid simulation for a current sheet in slab geometry to investigate how monotonic fast-mode compressional waves incident on a magnetopause current layer mode convert to short wavelength ($k_{\perp }\rho _{i} \sim 1$) kinetic Alfvén waves near the Alfvén resonance surface, under a northward IMF. Structure of the mode conversion was examined for various driver wave frequency $\omega _{0}$, wave amplitude, wave vector, magnetopause half-width $D_{0}$, ion $\beta $ in the magnetosheath, and electron-to-ion temperature ratio. Figure 45 depicts contours of various quantities around the magnetopause, obtained by Lin et al. (2010) in a case with a northward IMF. The simulation is performed in the $xz$ plane, where $x$ is normal to the magnetopause and the magnetosheath field ${\mathbf{B}}_{0}= B_{0} {\mathbf{z}}$. At an early time, $t=80$ (normalized to the inverse of the magnetosheath ion gyrofrequency), the incident compressional wave has just reached the magnetopause boundary from the magnetosheath (left side). The incident wave has wave numbers $k_{x0}=k_{\perp 0}=0.262$ and $k_{z0}= k_{\parallel 0} = 0.196$ (normalized to the inverse of the ion inertial length). At later time $t=240$, larger-amplitude, short perpendicular wavelength KAWs are generated in the transition layer by mode conversion, where the background density and magnetic field have a large gradient. In these waves, the shear Alfvénic components $B_{y}$, $E_{x}$, ion flow $V_{iy}$, and the parallel current density $J_{\parallel }$ are well correlated. Strongly enhanced parallel electric field $E_{\parallel }$ is present in the resulting waves. Meanwhile, wavy perturbations in local ion temperature $T_{i}$ are also observed, correlated with the variations in $B_{y}$. On the right edge of the KAWs, the enhanced perpendicular wave vector has reversed direction to $k_{x}<0$, as seen from the tilt angle of the wave fronts, where the KAWs radiate back to the magnetosheath. The dependence of the compressional wave absorption rate on $\omega _{0}$, $\theta $, and $D_{0}$ was obtained and compared with the linear cold-fluid theory. The absorption rate $A$ was found to have a sensitive dependence on $\omega _{0}$ and the orientation of the magnetosheath ${\mathbf{B}}_{0}$. Because the group velocity of these waves is much smaller than the compressional wave, the amplitude of the transverse fluctuations is typically much larger than the amplitude of the compressional driver, consistent with magnetopause observations (Johnson et al. 2001). Although the 2D mode conversion is predominantly of linear physics, nonlinear harmonic generation was also found. As the incident wave amplitude is increased, harmonics of $\omega _{0}$ are generated, leading to the broadening of the mode conversion region and its spectral width. The presence of the higher frequency harmonic modes may significantly lower the threshold of the stochastic ion heating due to KAWs (Johnson and Cheng 2001).

**Fig. 46:**
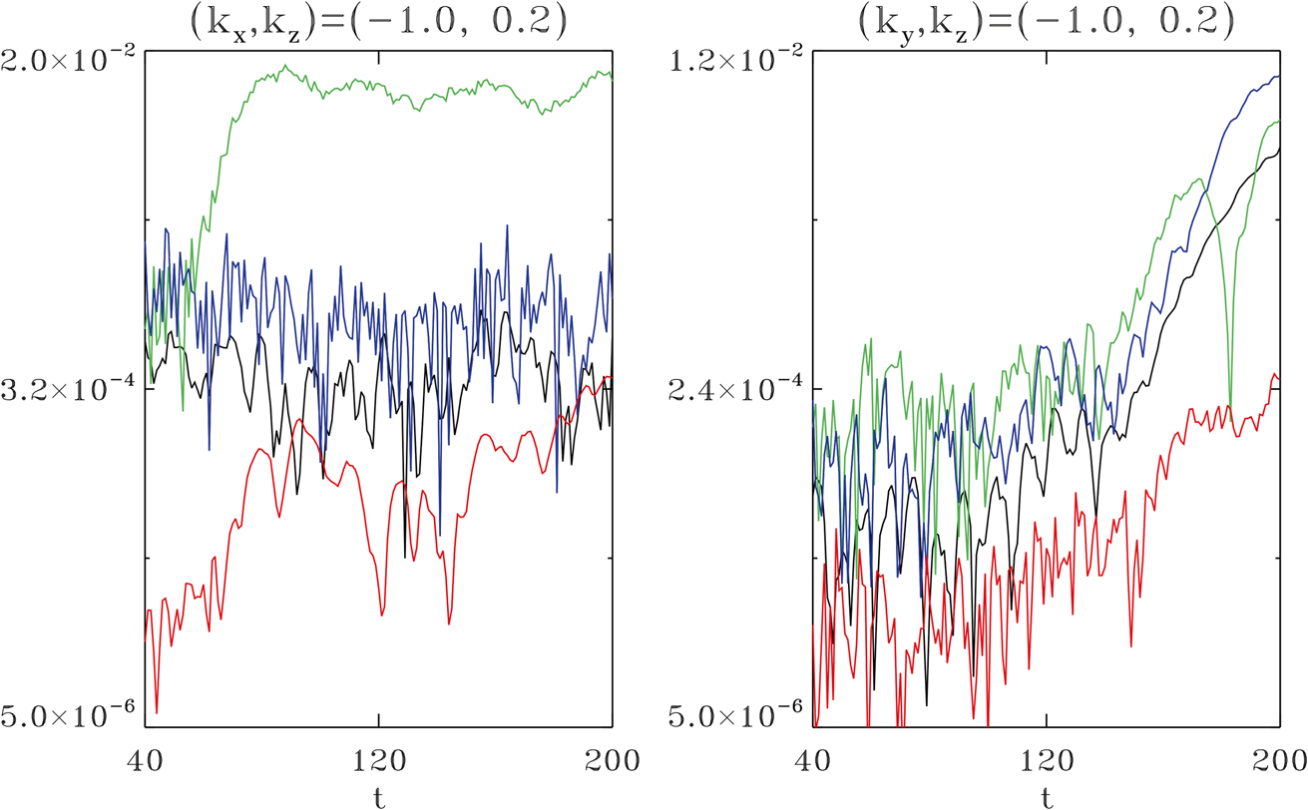
3-D physics of mode conversion: Time evolution of $B_{x}$ (black), $E_{x}$ (green), $E_{y}$ (blue), and $E_{\parallel }$ (red) for the KAWs modes dominated by $k_{x}$ (left column) and for those dominated by $k_{y}$ (right column) during the mode conversion in the inhomogeneous magnetopause (Lin et al. 2012)

When extending the hybrid simulation of Lin et al. (2010) to 3-D, Lin et al. (2012) found that the 3-D, nonlinear physics is fundamentally important to the mode conversion process. The simulation showed that following a stage of mode conversion to KAWs with $k_{x} \rho _{i} \sim 1$ (dominated by the linear physics), the growth of KAW modes with $k_{y} \rho _{i} \sim 1$ (with $k_{y}$ being perpendicular to both the background ${\mathbf{B}}_{0}$ and the wave vector ${\mathbf{k}}_{0}$ of the incident compressional wave) was observed in the nonlinear stage. Figure 46 shows the time evolution of the modes dominated by $k_{x}$ (left column) and modes dominated by $k_{y}$ (right column), for the same case shown in Fig. 45 but run in 3-D. The first-stage growth of KAWs from $t=40$-80, shown in the $k_{x}$ dominant mode, is due to the linear physics of mode conversion from the fast wave to KAWs, which results in the growth of $E_{x}$ and $E_{\parallel }$ when the incident fast wave reaches the magnetopause boundary. The later stage of evolution in $t>80$ is due to the nonlinear physics in which the mode coupling to $k_{y}$ plays a significant role. In $t=80$-145, the strong growth in $B_{x}$, $E_{y}$, and $E_{\parallel }$ is consistent with KAWs that are dominated by $k_{y}$, which nonlinearly co-exist with the KAWs dominated by $k_{x}$. Finally for $t>145$, the wave perturbations of $k_{x}$ and $k_{y}$ modes become isotropic with both $E_{x}$ and $E_{y}$ growing at the same rate. The generation of the perpendicular and azimuthal $k_{y}$ spectrum results when the amplitude of KAWs generated by linear mode conversion becomes large enough to drive a nonlinear parametric decay process, accompanied by a simultaneous excitation of zonal flow modes with similar large $k_{y}$. Since the diffusion coefficient across the gradient is proportional to the azimuthal $k_{y}^{2}$ (Johnson and Cheng 1997; Chen 1999), the KAWs generated in the nonlinear stage may lead to massive particle cross-field line transport.

**Fig. 47:**
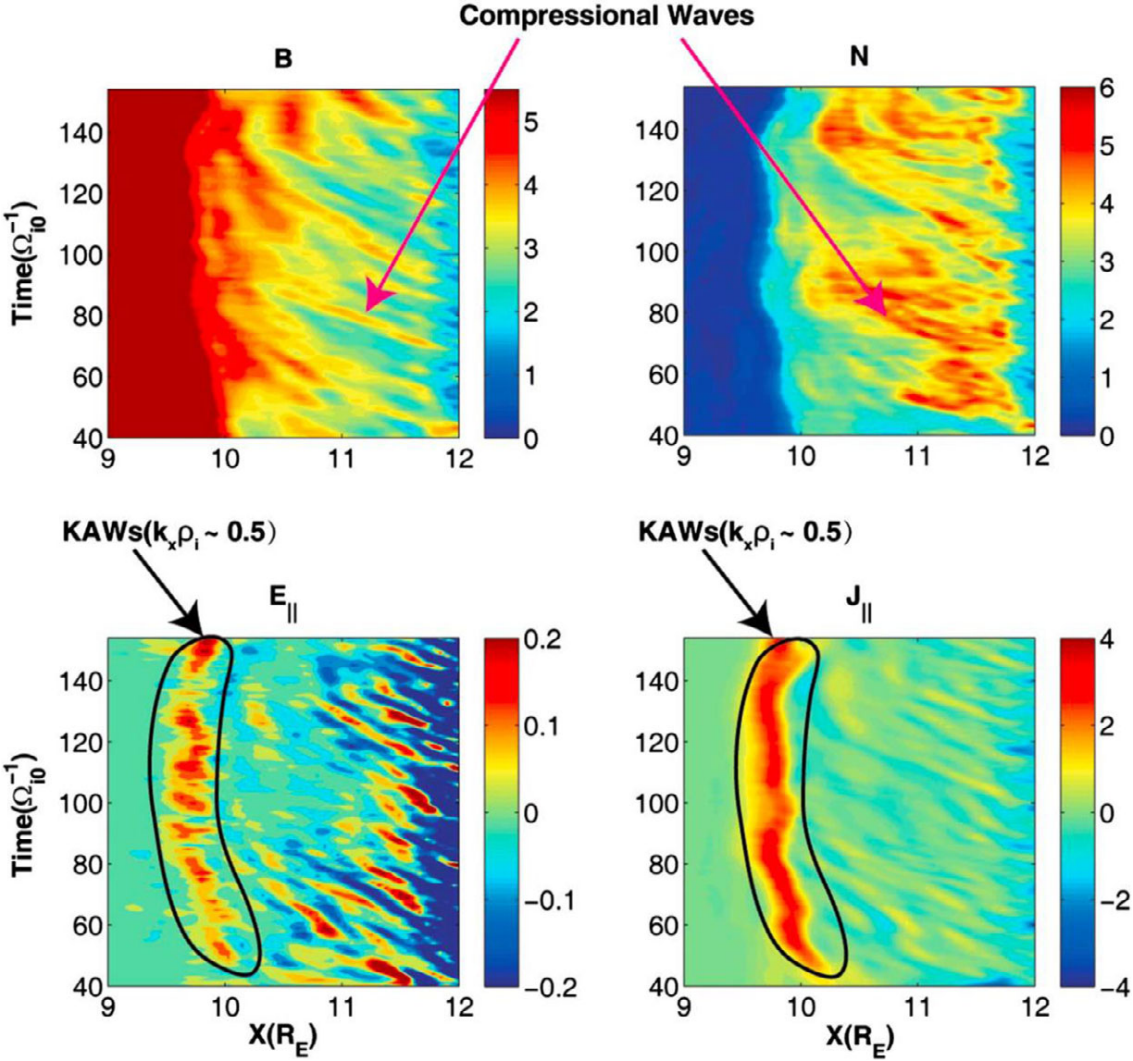
Time evolution of spatial profiles of various quantities along the Sun-Earth line in a case of 3-D global hybrid simulation with an oblique IMF shows the interaction between compressional waves from the foreshock and the magnetopause, with the circles highlighting the areas of the KAW structures with locally excited $E_{\parallel }$ around the MPBL. (Shi et al. 2017a)

**Fig. 48:**
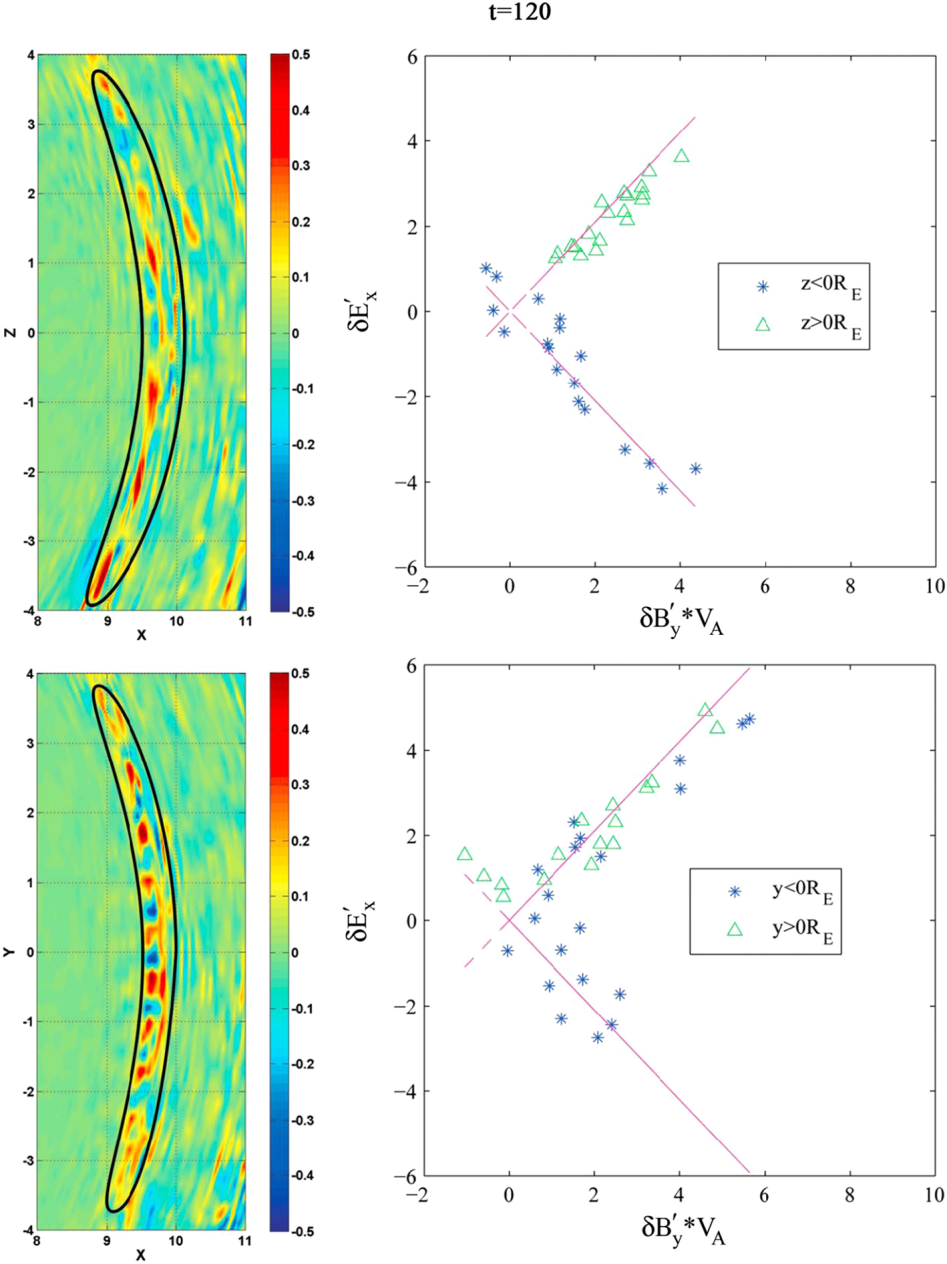
Global hybrid simulation results for the case shown in Fig. 47. (Top left) Spatial contours of $E_{\parallel }$ around the MPBL in the noon-meridian plane. (Top right) Corresponding polarization relation in the circled regions in the top left plot showing Alfvén modes propagating in different directions along the field lines. (Bottom row) Similar to the top row, except for the equatorial plane. The violet dashed lines in the right column represent the theoretically predicted polarization relation of KAWs. (Shi et al. 2017a)

The self-consistent interaction of foreshock waves with the dayside magnetosphere and the associated mode conversion process have been investigated by Lin and Wang (2005) and Shi et al. (2013, 2017a) using 3-D global-scale hybrid simulations. In the case with a radial IMF (Lin and Wang 2005; Shi et al. 2013), the quasi-parallel shock dominates the dayside. Large-amplitude compressional waves of the foreshock lead to the magnetopause surface perturbations. The results show that as the compressional pulses/packets, with a broadband frequency, propagate from the magnetosheath to the magnetopause, short wavelength structures of $k \rho _{i} \sim 1$ with enhanced parallel electric field $E_{\parallel }$ are excited in the magnetopause boundary layer (MPBL). The wave phase relationship between the magnetic field and the density changes from in-phase in the magnetosheath to anti-phase in the short-wavelength MPBL perturbations. The wave polarization is predominantly compressive in the magnetosheath, whereas strong transverse wave powers appear abruptly around the MPBL. The mode conversion from the compressional pulses to KAWs is identified around the predicted Alfvén resonance points in the MPBL (Shi et al. 2013). The short-wavelength KAWs are identified by the sharp increases in $E_{\parallel }$ and the transverse electromagnetic field polarization relation of Alfvén mode. These KAW perturbations propagate poleward into the cusps along the MPBL, carrying Poynting fluxes and field-aligned currents. Excitation of the azimuthal wave numbers following the growth of KAWs was also shown. The global hybrid simulation was further performed for cases with various IMF and solar wind conditions (Shi et al. 2017a). Figure 47 shows the spatial profiles of various quantities along the Sun-Earth line in a time sequence of $t = 40-150$ (normalized to inverse of the solar wind ion gyrofrequency) obtained from a case with an oblique IMF in the $B_{x}$-$B_{y}$ plane (cone angle of $30^{\circ }$) and Mach number $M_{\mathrm{{A}}}=5$ obtained from the 3-D global hybrid simulation of Shi et al. (2017a). Incoming foreshock compressional wave pulses characterized by the perturbations in magnetic field $B$ and ion density $N$ repeatedly propagate onto the magnetopause from the bow shock. The stronger wave pulses also lead to the generation of strong perturbations of $E_{\parallel }$ and $J_{\parallel }$. Correspondingly, the wave polarization relation is shown in Fig. 48 by plotting the relation between the transverse electric field and magnetic field for locations in the MPBL where $E_{\parallel }$ is enhanced. The results are nearly consistent with the theoretical prediction for KAWs. Around the subsolar region at the magnetopause, it can be distinguished that two wave branches propagate northward and southward, separated around the equatorial plane. For cases with an oblique IMF, the amplitude of magnetosheath compressional waves is larger at the quasi-parallel shock than at the quasi-perpendicular shock. The KAWs generated from the magnetopause mode conversion can be carried to the regions downstream of the quasi-perpendicular shock owing to the flow convection at the magnetopause. The KAWs are more intense in the downstream of quasi-parallel shocks than downstream of quasi-perpendicular shocks. It was also found that under a reduced, near-critical Mach number, KAW structures due to mode conversion exhibit a feature of broader excitation regions in the MPBL.

The mode conversion process has been suggested as a directly-driven mechanism for the generation of the frequently observed discrete harmonic frequencies of shear Alfvénic field line resonances in the dipolar field region (Engebretson et al. 1991b; Chi et al. 1994; Clausen et al. 2009; Wang et al. 2018c), including kinetic FLRs (Chaston et al. 2014). As shown in the global hybrid simulation of Lin and Wang (2005), in addition to the resonance absorption at the magnetopause, part of the energy will be transmitted into the magnetosphere. As predicted by theories discussed above, a standing wave pattern of shear Alfvén waves is developed, leading to the resonance of closed field lines. A fundamental odd resonance mode is obtained.

**Fig. 49:**
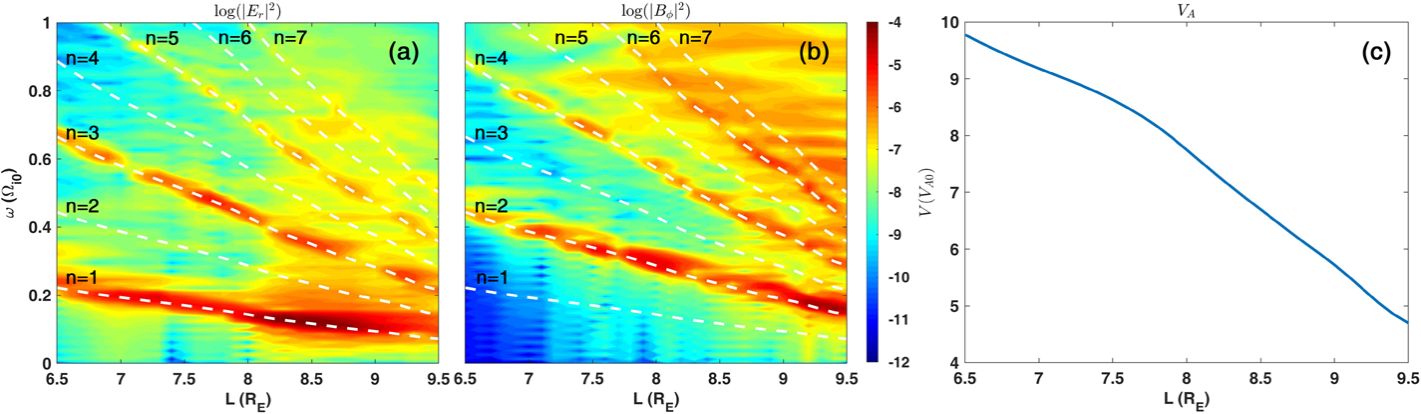
Generation of standing toroidal Alfvén modes in the magnetosphere by foreshock perturbations obtained from 3D global hybrid simulation: Wave power spectra of (**a**) radial electric field $E_{r}$ and (**b**) azimuthal magnetic field $B_{\phi }$ as a function of $L$ in the noon-meridian plane, and (**c**) the corresponding equatorial Alfvén speed. The white dashed lines mark the first 7 harmonic modes of the FLRs. (Shi et al. 2021)

Using coordinated observations in the foreshock and the magnetosphere, Wang et al. (2018c) found direct evidence of Pc 5 field line resonances driven by the foreshock cavity. ULF waves excited in the magnetosphere have a frequency similar to that of the foreshock pressure pulses. The toroidal modes are consistent with the shear Alfvénic FLRs, while poloidal modes are also present. A pair of traveling convection vortices and auroral brightening were generated in the ionosphere correspondingly. Recent 3D global-scale hybrid simulations of Shi et al. (2021) assuming a radial IMF illustrated for the first time the properties of self-consistently generated field line resonances through direct mode conversion in magnetospheric response to the foreshock disturbances. It was found that the foreshock wave spectrum covers a wide frequency range and matches the band of FLR harmonics. The fundamental harmonic of field line resonances dominates and has the strongest wave power, and the wave power decreases with the harmonic order. The eigenstructures of the harmonic modes along dipole-like field lines were obtained. The power of the toroidal Alfvén waves is steady under the steady solar wind inputs, suggesting that the FLRs are part of the steady-state magnetosphere. At the equator, an alternating feature of toroidal Alfvén wave components $E_{r}$ (radial) and $B_{\phi }$ (azimuthal) is present. As the Alfvén speed increases inwards in the magnetosphere, the frequencies of toroidal Alfvén harmonic modes increase accordingly. Figure 49 depicts the power spectral density (PSD) as a function of $L$ for $E_{r}$ (Fig. 49a) and $B_{\phi }$ (Fig. 49b) at the equator in the noon-meridian plane. The time-averaged equatorial Alfvén speed is plotted in Fig. 49c. The white dashed lines mark the first 7 orders of $n$-harmonic modes of the FLRs. The enhanced odd harmonic numbers in $E_{r}$ and even-harmonic numbers in $B_{\phi }$ are features of standing toroidal Alfvén waves. In addition, field-aligned currents in the cusp region indicative of possibly observable aurora were found to be a result of magnetopause perturbations caused by the foreshock disturbances. There exists a low $m$-number ($m \sim 5$) azimuthal mode in the dayside driven by the FLRs, and the motion of these FAC structures is both poleward and azimuthal. The simulation also showed that when the solar wind Mach number decreases, the strength of both FLRs and field-aligned currents decreases accordingly.

#### Triggering of Magnetopause Reconnection

It has been reported that a local magnetic reconnection can be triggered (Hietala et al. 2018; Ng et al. 2021), or an impulsive penetration may occur when a magnetosheath jet impinges on the magnetopause (Dmitriev and Suvorova 2015).

**Fig. 50:**
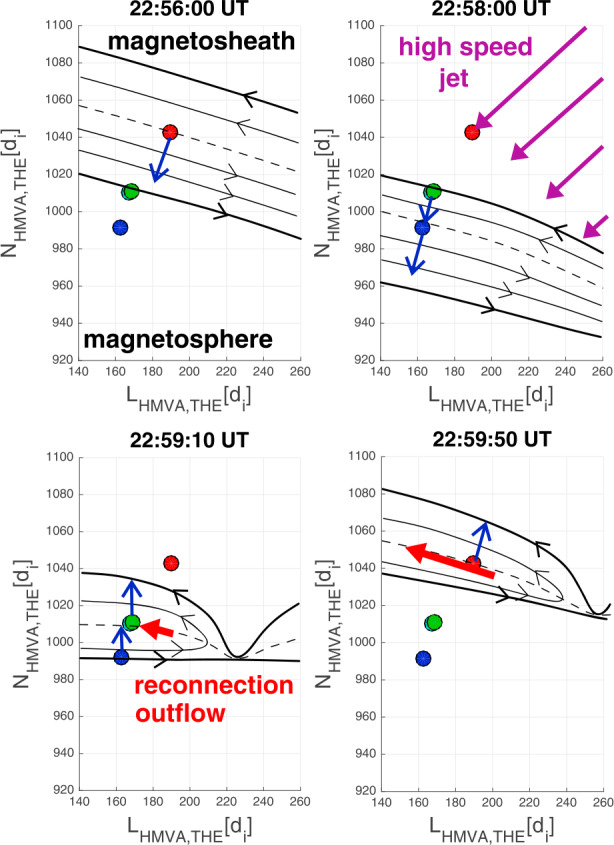
Sketches illustrating the magnetosheath jet triggering the magnetopause reconnection. Black lines indicate the magnetopause current layer, with the current sheet center marked by the dashed line and their normal vectors by blue arrows. Purple arrows indicate the magnetosheath jet and the red arrows the reconnection outflow. (After Hietala et al. 2018)

As shown in Fig. 50, the magnetopause impact by a magnetosheath jet has been examined by Hietala et al. (2018). Before the magnetosheath jet impact, the magnetopause current layer was thick (60–70 $d_{i}$) with a high magnetic shear of 140^∘^–160^∘^, without reconnection signatures. Shortly after the magnetosheath jet impact, reconnection outflows were observed within the magnetopause layer while the magnetic shear did not change significantly from pre-impact to post-impact. Thus, it is suggested that the observed reconnection at the magnetopause has been triggered by the magnetosheath jet’s compression on the magnetopause current sheet. These observations suggest that magnetosheath jet impacts may trigger magnetopause reconnection.

**Fig. 51:**
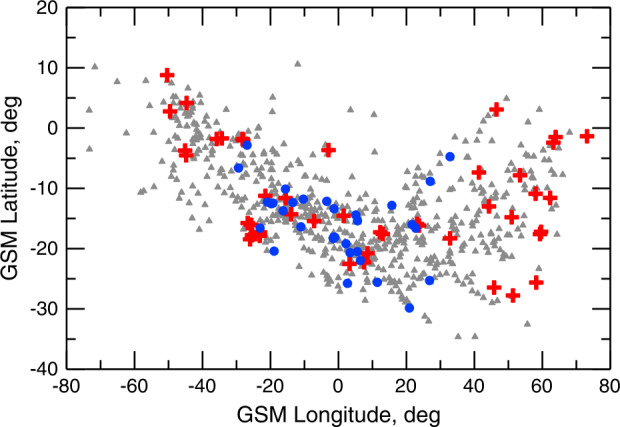
GSM location of 646 magnetosheath jets (gray triangles) observed by THEMIS satellites from 2007 to 2009. The red crosses indicate the jets, which result in plasma penetration across the magnetopause based on multi-spacecraft observations. The blue circles indicate the jets, which are not accompanied by the plasma penetration. (Dmitriev and Suvorova 2015)

The interaction of magnetosheath jets with the magnetopause has been revealed from a sudden inward motion of the magnetopause and an enhancement in the geomagnetic field. The magnetosheath plasma penetration was determined as appearance of the magnetosheath plasma against the background of the hot magnetospheric particle population (Dmitriev and Suvorova 2015). A total of 646 magnetosheath jets have been identified using THEMIS data from 2007 to 2009. The jets were identified as the ratio $R$ of the total energy density of a jet to that of the upstream solar wind to be larger than 1 for $\Delta T = 30\mbox{ s}$ and longer. As shown in Fig. 51, the spatial range of magnetosheath jets location was rather scattered. Hence, it seems that the magnetosheath plasma penetration does not depend on the magnetosheath jets location. Apparently, not every magnetosheath jet was accompanied by a plasma penetration in the magnetosphere. It is found that 44 out of 76 jets (almost 60%) were accompanied by the plasma penetration. The other 32 jets were not accompanied by the penetration.

### Transient Dayside Magnetopause Processes at Other Planets

Observations of transient dayside magnetopause processes at other planets have been reported. KH instabilities have been observed at Mercury (Slavin et al. 2008), Venus (Wolff et al. 1980), Mars (Penz et al. 2004), Saturn (Pu and Kivelson 1984), and cometary ion tails (Ray 1982). FTEs are prevalent at Mercury’s magnetopause with a typical duration of ∼1 s and spacing of 4 s (Russell and Walker 1985; Sun et al. 2020). They have been observed during both northward and southward IMF conditions with a higher occurrence rate during southward IMF. FTEs in the shower events (≥10 FTEs in a magnetopause crossing) carry most of the magnetic flux that drives Mercury’s Dungey cycle (Sun et al. 2020). At giant planets, in contrast, observations of FTEs are rare. Fourteen possible FTEs were found at Jupiter (Walker and Russell 1985) and only one FTE at Saturn was reported (Jasinski et al. 2016).

The giant planet magnetospheres contain significant local time asymmetry. These pronounced dawn-dusk asymmetries indicate that the solar wind must, fundamentally, play an important role (Hill et al. 1983; Khurana 2001). For instance, Jupiter’s magnetodisc is highly stretched in the midnight to dawn sector, and more dipolar in the pre-noon to dusk sector. Even deep in the inner magnetosphere, a persistent dawn-dusk (east-west) asymmetry of the UV emissions from the Io plasma torus (Sandel and Broadfoot 1982) led two teams, Barbosa and Kivelson (1983) and Ip and Goertz (1983), to propose that flow down the magnetotail could impose weak stresses on the Io plasma torus, consistent with a dawn-to-dusk electric field (few mV/m) that would displace plasma orbits toward dawn. Delamere and Bagenal (2010) further suggested that the electric field could be imposed by a strong viscous interaction starting at the dayside magnetopause boundary. Jupiter and Saturn both exhibit significant local time asymmetry, providing compelling evidence that the solar wind interaction must be at the root of this problem.

There has been some debate regarding the nature of the solar wind interaction with the giant planet magnetospheres. Following the New Horizons excursion down Jupiter’s magnetotail, McComas and Bagenal (2007) suggested that Jupiter’s interaction with the solar wind was fundamentally different from Earth due to the lack of a well-defined and tailward-extending plasma sheet. Much of the subsequent debate is summarized by Delamere (2015), comparing the relative importance of large-scale reconnection (Dungey 1961), a viscous-like interaction (Axford and Hines 1961), and the internally-driven Vasyliunas cycle (Vasyliunas 1983). Delamere and Bagenal (2010), Delamere and Bagenal (2013), and Masters (2018) argued that the viscous-like interaction may play a dominant role in the solar wind interaction due, in part, to low reconnection rates and to the large sheared flows (maximum on the dawn flank, minimum on the dusk flank) that exist on the dayside magnetopause boundaries, leading to the KH instability.

**Fig. 52:**
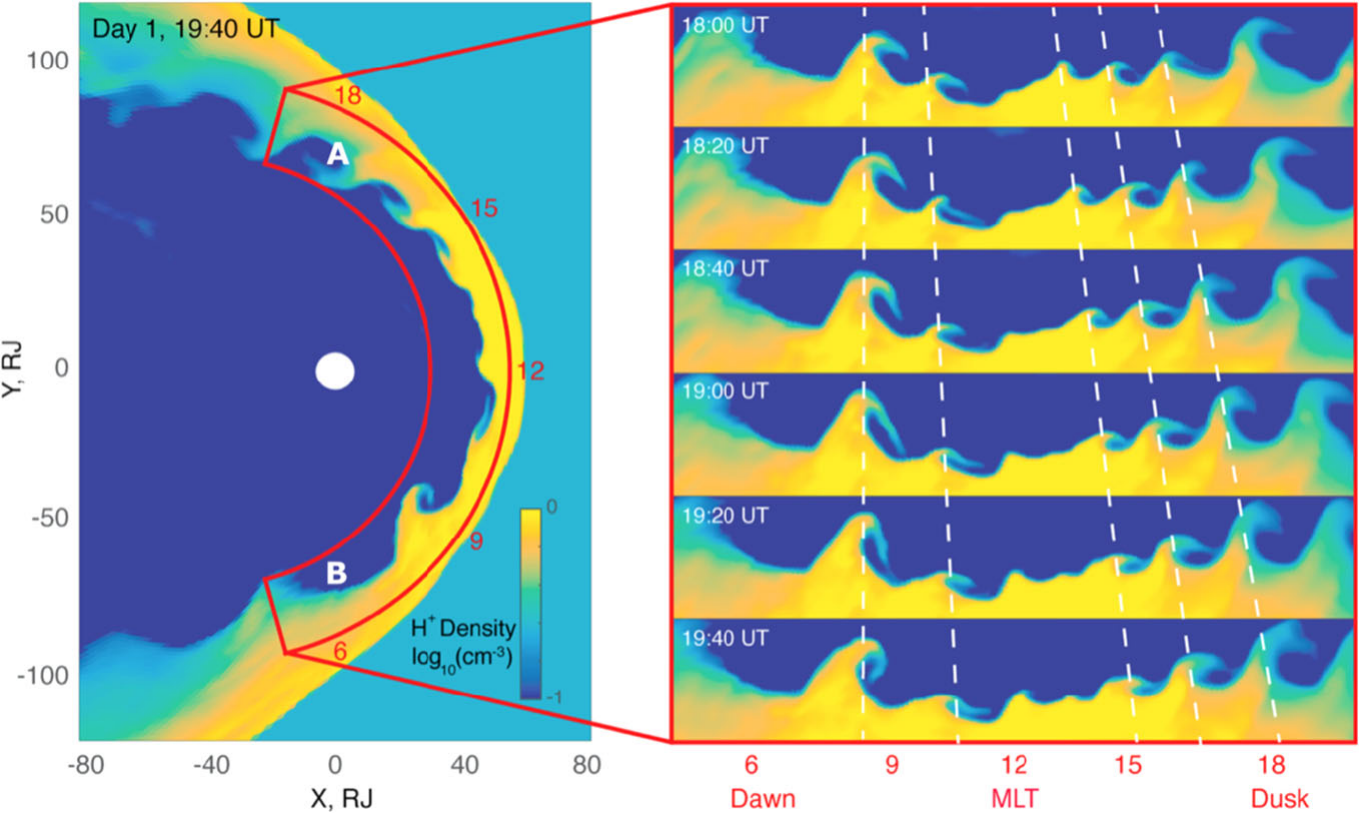
MFLFM simulation of Jupiter’s magnetosphere, showing the KH-unstable magnetopause boundary as well as dawn/dusk asymmetry (Zhang et al. 2018b)

There is abundant evidence that the KH instability is active at Saturn’s magnetopause boundary (Masters et al. 2009a, 2010; Wilson et al. 2012; Delamere et al. 2013). An initially surprising result was evidence for more KH activity in the post-noon sector where the sheared flow is minimized (i.e., magnetodisc corotation and sheath flows are both tailward). However, Ma et al. (2015), using a 2-D MHD simulation of Saturn’s dayside magnetopause, showed that KH vortices originating in the subsolar region (roughly from 11 to 14 local times) are transported to the postnoon sector and the wavelength is enlarged due to the gradient of sheared flow. Meanwhile, KH vortices formed in the prenoon sector (<10 LT) tend to diffuse rapidly and form a boundary layer, precluding the existence of well-defined vortex structures. As a result, vortex structures are more likely to be observed in the post-noon sector. To validate the simulation results, Ma et al. (2015) conducted a comprehensive boundary normal analysis (minimum variance analysis, MVA) and found that indeed the boundary normal directions fluctuate in a systematic manner consistent with the 2-D simulation results. Zhang et al. (2018a) also confirmed this result with global simulations (Fig. 52).

**Fig. 53:**
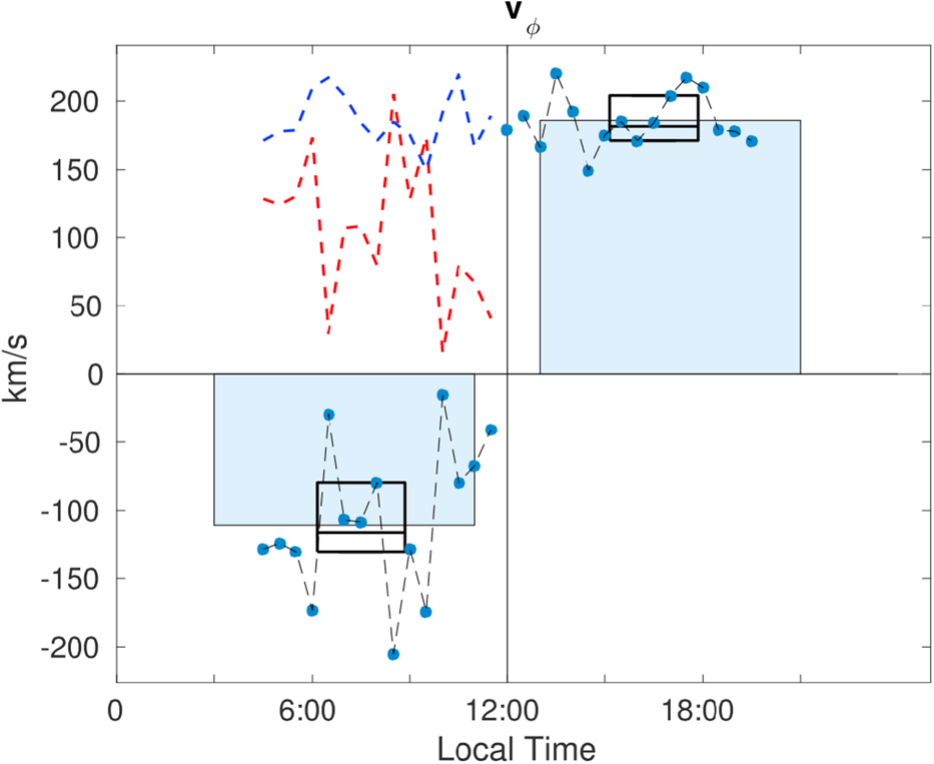
Dawn-dusk asymmetry in Saturn’s magnetosheath flows. The histogram is the dawn vs. dusk average, excluding 11:30 to 12:30 LT. (Burkholder et al. 2017)

Reduced dawn-side sheath flows at Saturn also provide compelling evidence for a viscous-like interaction (Burkholder et al. 2017). Numerically integrated moments from Cassini Plasma Spectrometer (CAPS) data (Thomsen and Delapp 2005; Thomsen et al. 2010) through 2011 were used to calculate average flow properties in the magnetosheath. Interestingly, there is a significant dawn-dusk asymmetry, with the dawn-side flows being substantially reduced from expectation. The asymptotic terminator flank value from MHD modeling by Desroche et al. (2013) for a polar flattened obstacle to flow is 200 km/s. Burkholder et al. (2017) found dusk flank values consistent with Desroche et al. (2013), but dawn flank values were ∼100 km/s. Figure 53 summarizes their result. The proximity to the magnetopause boundary and bow shock are critical for understanding the momentum transfer rates. The results are filtered based on the time elapsed from a boundary crossing (magnetopause and bow shock crossing were catalogued by Delamere et al. (2013)). Figure 53 is an average from data points within 500 minutes of the magnetopause boundary crossing (or roughly 3 $R_{\mathrm{S}}$ for a stationary boundary). Burkholder et al. (2017) conducted averages based on proximity to the magnetopause and the bow shock and found that asymmetry exists for roughly half of the sheath width.

**Fig. 54:**
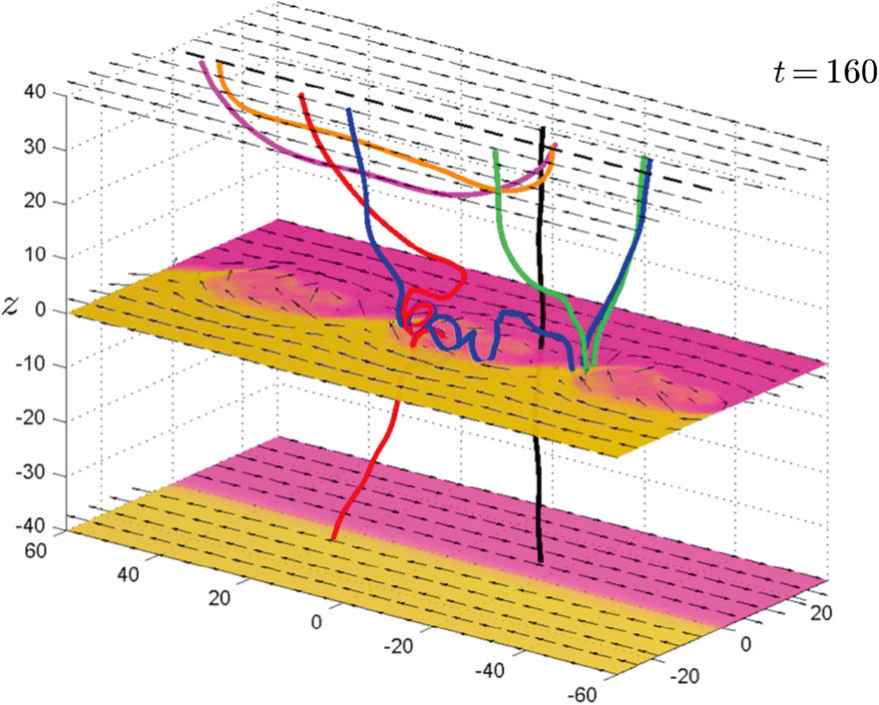
Selected field lines from a local MHD simulation of Saturn’s KH unstable magnetopause boundary. The red and black lines are closed field lines, and the green, blue, magenta, and orange lines are open field lines. (Ma et al. 2015)

Unlike the evidence for the KH instability at Saturn, magnetic reconnection has proved more elusive. Walker and Russell (1985) found 14 possible FTEs at Jupiter using the magnetic field observations from Pioneer 10 and 11 and Voyager 1 and 2. Indeed, the FTEs were associated with northward magnetosheath fields, but the electric fields were small in comparison to the magnetospheric corotation electric fields, leading to the conclusion that the FTEs were unlikely to influence magnetospheric flows. Lai et al. (2012) examined Saturn’s dayside magnetopause boundary using Cassini data and found no evidence for FTEs, but identified cases where the magnetic field threads the boundary, forming “magnetic bridges”. On the other hand, Jasinski et al. (2016) reported one FTE observed by the Cassini spacecraft at Saturn on 2 February 2007. Fuselier et al. (2014) showed that streaming electron distributions in the boundary layer are consistent with simple models predicting the location of reconnection. However, the situation could be more complicated because in three dimensions, the KH instability and reconnection interact (Ma et al. 2014a,b). Specifically, KH can thin the spine region connecting two vortices, triggering reconnection. Conversely, magnetic reconnection can thin a boundary in the presence of a sheared flow, triggering the KH instability. Recently, KH-associated reconnection was reported by Eriksson et al. (2016) and Li et al. (2016a) using MMS mission data at Earth. Figure 54 shows the complex magnetic field structure (with open and closed field lines) that can be generated by reconnection in a KH-unstable configuration (Ma et al. 2015; Walker et al. 2011).

**Fig. 55:**
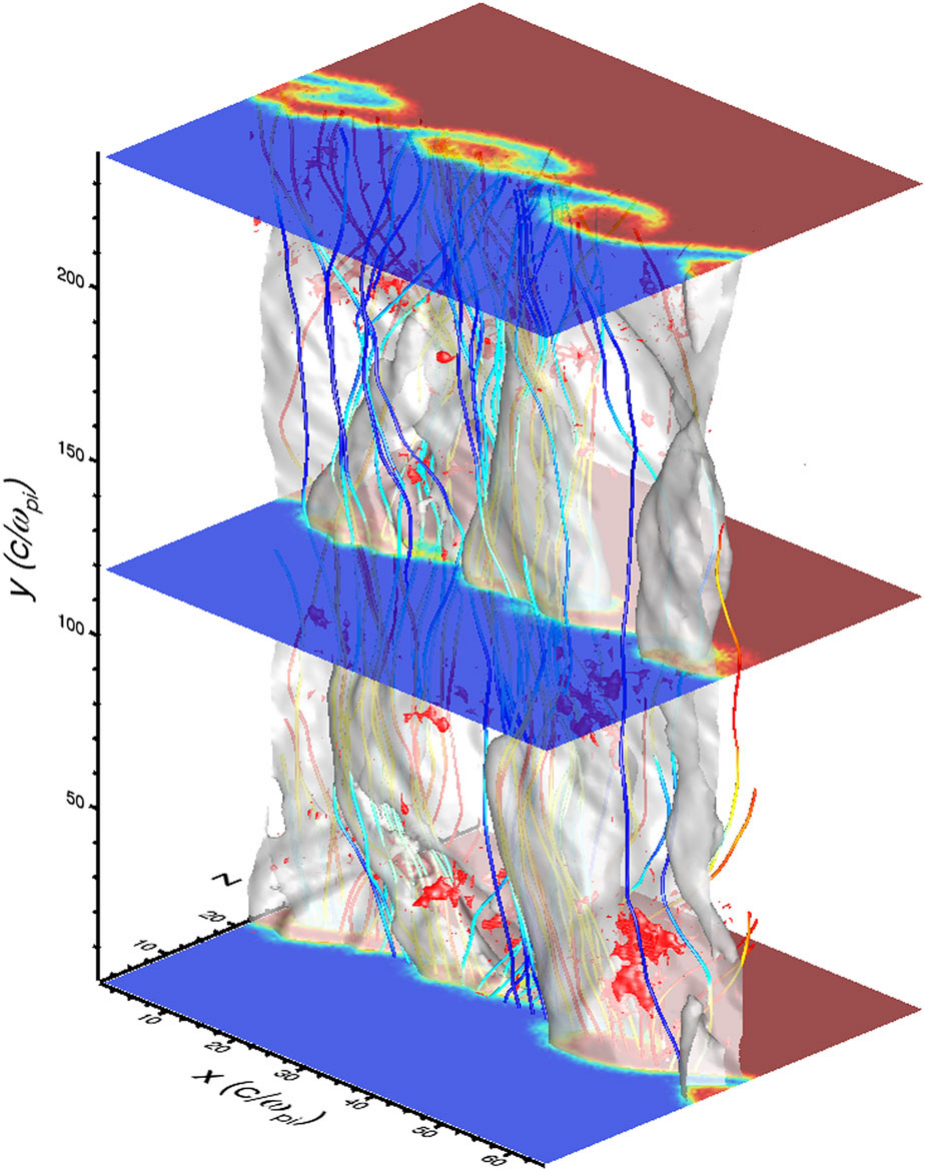
3-D Kelvin-Helmholtz hybrid simulation. The color slices show particle mixing, with grey representing the magnetopause boundary. Magnetic field lines are traced from the bottom to the top boundary. Red isosurfaces indicate parallel electric fields. (Delamere et al. 2018)

Delamere et al. (2018) conducted 3-D hybrid simulations of the KH instability for conditions appropriate for Saturn’s dawn-side magnetopause boundary (Fig. 55). In these simulations the phase of the surface waves can be different in the $y$ direction, allowing adjacent surface waves to twist the magnetic field into “candy wrapper” forms, promoting magnetic reconnection (Ma et al. 2016b, 2017). The Maxwell and Reynolds shear stresses generated at the boundary are a non-negligible fraction of the momentum flux density associated with the flow and Delamere et al. (2018) showed that the momentum transfer rates are consistent with estimates for the observed flow reduction in Saturn’s dawn-side sheath by Burkholder et al. (2017).

Magnetic reconnection under super-Alfvénic sheared flow conditions that are perpendicular to the reconnection plane adds further complexity to understanding the solar wind interaction with fast rotating magnetodiscs (Ma et al. 2016a). The KH instability can be suppressed for perpendicular flow shears in excess of the magnetosonic speed when reconnection operates first. When reconnection operates in sub-Alfvénic conditions, the evolution in the outflow region satisfies the Walén relation (i.e., $\Delta {\mathbf{v}} = \pm \Delta {\mathbf{v}}_{A}$). However, when reconnection operates in super-Alfvénic conditions, the outflow region acquires a significant magnetic field component along the sheared flow direction. The added magnetic pressure causes the outflow region to expand and the Walén relation is not satisfied. In addition, the expansion of the boundary will stabilize KH.

A critical aspect of the solar wind interaction is the role of dynamic pressure and the related variations in magnetospheric scale (Joy et al. 2002; Kanani et al. 2010; Pilkington et al. 2015). Compressions in the magnetosphere produced by, e.g., forward shocks can significantly modify azimuthal flows via conservation of angular momentum (Southwood and Kivelson 2001). Transitions from compressed to expanded configurations are poorly understood, particularly in terms of angular momentum transport within the magnetosphere.

Finally, magnetopause boundary processes can be the source of compressional modes in the magnetosphere, leading to resonant mode coupling (Glassmeier 1995). Both Jupiter and Saturn exhibit ultra-low-frequency, quasi-periodic behavior, which is likely a signature of resonant cavity eigenoscillations (Delamere 2016; Nichols et al. 2017).

The solar wind interaction with Jupiter and Saturn is fundamentally different from Earth. A complete understanding of the viscous-like interaction must combine flow shear (KH) instability with magnetic reconnection. Understanding the system-wide response to variations in solar wind conditions (e.g., dynamic pressure) is critical.

### Outstanding Questions

- A longstanding question of KH waves has been whether it operates symmetric at the dawn and dusk side flanks. While the typical Parker spiral configuration, properties of the magnetosheath, and kinetic physics imply that there should be an asymmetry, the question and the dominant physical mechanisms are not resolved.
- During periods of strongly northward IMF, the plasma sheet assumes a state called cold dense plasma sheet indicating the transport of cold magnetosheath material into the plasma sheet. The dominant candidates for this process are high latitude cusp reconnection or intermediate latitude KH induced reconnection at the boundary of the KH unstable flanks. Both processes have been demonstrated to be capable to generate sufficient mass transport and both processes have observational evidence for this transport. However, we do not know their relative importance and how this depends on magnetospheric and solar wind/IMF conditions.
- The specific entropy of the cold dense plasma sheet is more than an order of magnitude higher than in the magnetosheath. This amount of non-adiabatic heating represents a serious problem both for KH associated and cusp reconnection. In order to really understand the mass transport, we need to identify the mechanism that can explain the observed heating.
- An important aspect of the KH interaction with the magnetosphere is turbulence in the magnetosheath with somewhat controversial results in the past. Therefore, we need to identify the spatial distribution of turbulence in the magnetospheric flank, its temporal evolution, and relation to dense plasma distribution.
- Many papers in the past have attributed KH waves as one source of ULF waves (Pc 5 and Pc 4). However, much of this work uses correlation studies. Therefore, we need to identify the specific mechanism for the generation of these waves and their relation to the KHI/surface waves.
- As an initial value problem, the KHI depends strongly on the thickness of the initial velocity shear layer. However, KH at the flanks evolves as a result of its history further upstream. Can we define a thickness at some downstream location (based on other mechanisms) and how important is this for the evolution of the KH waves along the flanks of the magnetosphere?
- Global MHD simulations for both southward (Claudepierre et al. 2008) and northward IMF conditions (e.g., Li et al. 2012; Merkin et al. 2013) show that two KH modes may be simultaneously excited, the outer one that grows along the magnetopause and the inner one at a velocity shear layer somewhat earthward of the magnetopause. However, there is no evidence for such two KH modes in actual magnetospheres. Questions thus remain about whether the two KH modes exist in reality and, if so, whether there is any significant role played by the presence or interaction of the two modes.
- There has been some work on the interaction of KH waves and reconnection and it is highly likely that this interaction occurs under certain conditions. However, it not clear whether there is a connection between FTEs and KHI, and what the expected signatures for such events might be.
- Several studies have associated ionospheric (auroral and convection) signatures with KH waves at the magnetospheric boundaries. However, it is not clear how this coupling occurs and whether such signatures can uniquely be attributed to such waves. Can ionospheric signatures be used as a diagnostic for KH waves?
- Observations indicate that reconnection can have different scale sizes and modes. However, there is no simple picture of FTE formation emerging from observations. Therefore, it is highly important to determine the distribution of sizes and locations of FTE associated reconnection patches on the dayside magnetopause.
- There have been several observations of interlinked FTE flux ropes confirming that this situation can occur at least occasionally. We do not understand the associated flux and mass transport. How are FTE flux ropes convected toward the tail when the flux tubes are interlaced and have the “wrong” hemispheric connection? What are the properties and signatures of such interlinked flux tubes? What is the topological connection of flux ropes created by two longer X lines and how does it resolve into a simple north or southward connection?
- MMS results have shown the formation of secondary flux ropes at the magnetopause. Is there any significant role played by these flux ropes at the magnetopause? If that is the case, what is the role?
- Considering the different manifestations of FTE structures and sizes indicated by observations, it appears of major importance to identify what controls the stability, growth, and decay of FTEs.
- Early studies have focused to identify classical signatures of stationary reconnection. Often such signature and FTEs are considered as different modes of reconnection but this is by no means clear. Just based on convection any X line or reconnection patch can be expected to be blown off the dayside magnetopause within 10 to 20 minutes such that reconnection has to reform at least on this time scale. However, any newly formed reconnection site will develop at least initially a jet that should satisfy conditions of steady state models. Therefore, we need to resolve the relation between FTEs and so-called stationary reconnection.
- Early studies (e.g., Papamastorakis et al. 1989) have demonstrated that a number of FTEs satisfy the conditions of stationarity (de Hoffmann-Teller frame) and have an Alfvénic character (Walén relation) (see also Papamastorakis et al. 1989). This seems an important tool to study the character of FTEs and their evolution. Do all FTEs satisfy these conditions?
- As outlined, magnetosheath waves and other perturbations (KH, impulsive events, etc.) can couple to kinetic Alfvén waves at the magnetospheric boundary and these waves can cause mass transport and heating. However, it is not clear how important this effect is for instance compared to mass transport associated with reconnection.
- The same question applies to other kinetic or two fluid effects. For instance, the thin boundaries that develop in KH waves have been demonstrated to allow for enhanced diffusion. What are the conditions where this mass diffusion (or transport in filamentary structures) becomes the dominant aspect of the KH wave associated transport?

## Geoeffects of Dayside Transients

### Effects in the Magnetosphere

Transient solar wind structures associated with sudden changes (either positive or negative) in the dynamic pressure compress or inflate the Earth’s magnetosphere. The geomagnetic fields observed on the ground consequently increase or decrease. The magnetopause current intensifies when a positive impulse impinges on the magnetosphere. ULF waves can be excited in the inner magnetosphere as a result of the interaction between the Earth’s magnetic field and solar wind pressure impulses (Zong et al. 2009, 2017; Zong 2022; Zhang et al. 2010). Furthermore, these waves can accelerate energetic particles (Zong et al. 2007, 2009, 2012).

The pressure perturbations associated with foreshock transients typically have spatial scales of a few $R_{\mathrm{{E}}}$ but can be significantly larger in magnitude than the solar wind dynamic pressure pulses. They in turn cause large localized deformation of the magnetopause. For example, the dynamic pressure inside HFAs is lower than the ambient solar wind due to the density depletion and flow deflection. The passage of HFAs will therefore result in local negative pressure impulses. The depletion of the total pressure in HFAs leads to a local sunward expansion of the magnetopause that has been observed. In their comprehensive study of a single HFA event, Sibeck et al. (1999) reported that the magnetopause moved 5 $R_{\mathrm{{E}}}$ outward in response to the density decrease accompanying an HFA. Similarly, foreshock bubbles and foreshock cavities may also have significant impacts on the magnetosphere due to the dynamic pressure variations in these structures.

Foreshock transients such as HFAs impact the magnetosphere in several ways. They can transmit compressional fast magnetosonic waves into the magnetosphere that can excite resonant ULF waves (Eastwood et al. 2011; Zhao et al. 2017; Shen et al. 2018), lead to plasma flow vortex structures and reconfiguration of the magnetotail, cause particle acceleration or scattering into the loss cone and precipitating into the ionosphere, generate field-aligned currents in the magnetosphere that drive magnetic impulse events in the high-latitude ionosphere (Eastwood et al. 2008), and trigger transient auroral brightenings (Sibeck et al. 1999; Fillingim et al. 2011).

Magnetospheric ULF waves have received considerable attention recently because observations have shown that ULF waves are capable of controlling and accelerating radiation belt electrons (e.g., Elkington et al. 1999; Zong et al. 2007, 2008, 2009; Mann et al. 2013), ring current ions (e.g., Yang et al. 2011; Zong et al. 2012; Ren et al. 2016), and cold plasmaspheric electrons (e.g., Zong et al. 2017; Ren et al. 2017, 2018, 2019). The shear Alfvén mode ULF waves, with frequencies comparable to the eigenfrequencies of the geomagnetic field lines, develop into standing waves as a consequence of superposition of incident waves and reflected waves from the Earth’s ionosphere. A characteristic feature of standing waves is a 90^∘^ phase difference between the transverse components of the magnetic and electric fields in the local mean-field-aligned coordinate system (Singer et al. 1982; Takahashi et al. 2011; Le et al. 2017).

The magnetic gradient and curvature drift motion of charged particles is aligned with the azimuthal electric field component of poloidal mode ULF waves, allowing fast acceleration of energetic particles to occur in the magnetosphere (e.g., Zong et al. 2011, 2017). When a particle’s drift velocity equals the wave phase velocity in the azimuthal direction, it experiences a constant electric field and gains energy from or loses energy to waves (depending on the direction of the wave electric fields), which is a process known as drift resonance (Southwood and Kivelson 1981). Zong et al. (2009) found that electron fluxes in the 30-300 keV energy range enhanced several times within an hour due to continuous and dramatic acceleration through drift resonance with fundamental-mode ULF waves. Relativistic electrons can be accelerated by low-$m$ ULF waves. Hao et al. (2019) showed that ULF waves induced by an interplanetary shock impinge on the Earth’s magnetosphere can efficiently accelerate ultrarelativistic electrons up to 3.4 MeV in the outer radiation belt. Numerical simulations confirmed the adiabatic acceleration and transport processes of outer radiation belt electrons through drift resonance (Elkington et al. 1999; Wang et al. 2018d) and revealed the possibility of locally growing phase space density peaks produced by bursts of narrow-band ULF waves (Degeling et al. 2008).

For ring current ions and cold plasmaspheric electrons, their bounce motion should be taken into consideration during the wave-particle interactions because their bounce frequency is comparable to the wave frequency (Southwood and Kivelson 1981; Zong et al. 2017). A more complicated process, drift-bounce resonance, occurs under the resonant conditions associated with harmonic mode ULF waves (Ren et al. 2016, 2017; Liu et al. 2020g). Due to their mass difference, the drift-bounce resonance condition for ring current oxygen ions is much easier to satisfy than that for the protons (Zong et al. 2012; Ren et al. 2016; Zong et al. 2017), providing a potential explanation for the stronger O^+^ acceleration and flux enhancement during storms. Recently, interactions between ULF waves and cold plasmaspheric populations have been investigated. Observations have demonstrated that ULF waves play a crucial role not only in the energization and transport of cold electrons and ions (Yue et al. 2016; Ren et al. 2017, 2018, 2019; Liu et al. 2019d), but also in the spatial and temporal dynamics of various waves (e.g. hiss, chorus, and kinetic Alfvén waves), as well as the subsequent wave-particle interactions due to the modulation of the plasma density and the stronger anisotropy in energetic particle distributions caused by ULF waves (e.g., Breneman et al. 2015; Malaspina et al. 2015; Li et al. 2011).

#### Generation of ULF Waves in the Magnetosphere by Dayside Transients

**Fig. 56:**
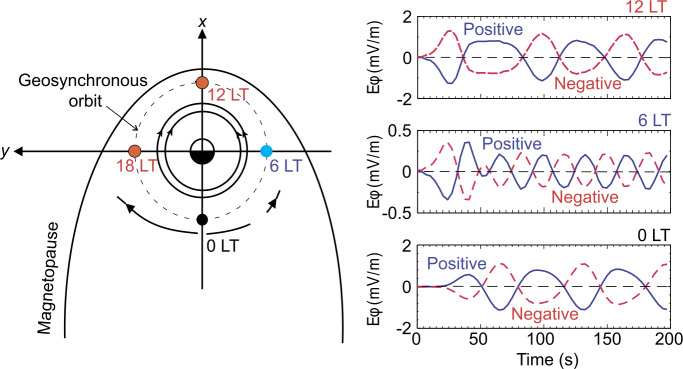
Illustration of the poloidal mode ULF waves excited by a positive/negative solar wind impulse at 12:00 LT, 6:00 LT, and 00:00 LT at geosynchronous orbit. The arrows schematically show the drift motion direction of ions (solid) and electrons (dashed) from midnight. (After Zhang et al. 2010)

Foreshock transients are potential energy sources for magnetospheric ULF waves because the localized pressure perturbations introduce seed fluctuations on the magnetopause boundary, which can trigger/generate various types of waves. It has long been known that solar wind pressure pulses excite ULF waves in the magnetosphere. For example, Fig. 56 illustrates poloidal mode waves excited by a positive (blue) and negative (red) solar wind impulse at different local times at geosynchronous orbit (Zhang et al. 2010). However, it has only been in the last decade or so the roles of foreshock transients in the generation of magnetospheric ULF waves started to gain attention. Several studies provided direct observational evidence that foreshock transients generated magnetospheric ULF waves, in either Pc 3-4 or Pc 5 bands. However, theoretical and numerical modeling work focused on foreshock transients as the wave source is still largely lacking. The wave generation mechanisms have not been fully explored.

It has long been well known that the main source of energy for magnetospheric Pc 3-4 waves are upstream waves generated by resonant interaction between backstreaming ions in the foreshock and the incoming solar wind. The causal relationship was established by the observed correlations between the wave occurrence and the IMF direction, as well as between the wave frequency and the IMF strength (e.g., Russell and Hoppe 1981; Le and Russell 1992, 1996). However, there is a subset of Pc 3-4 waves whose frequencies are not correlated with the IMF strength, implying other mechanisms are at work.

Eastwood et al. (2011) first reported observational evidence of transient Pc 3 waves generated by an HFA in the absence of significant upstream waves in the same frequency band. They suggested that the HFA created a localized perturbation of the magnetopause because of large variations in the dynamic pressure. Compressional waves could be generated as the perturbation interacted with the magnetopause and subsequently coupled into the magnetosphere.

**Fig. 57:**
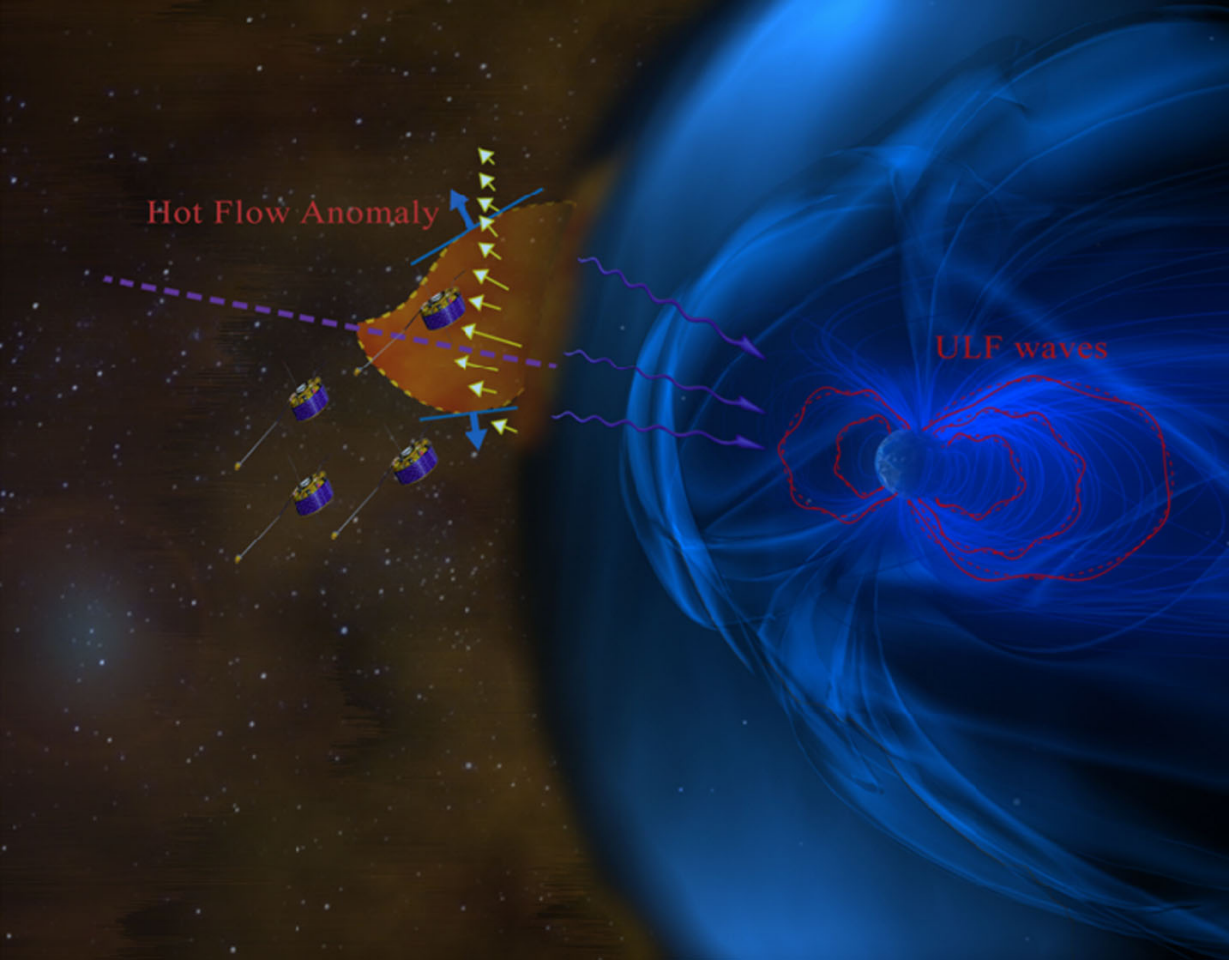
Schematic graph of an HFA generating global ULF waves in the magnetosphere (credit: Peking University)

Recent observations (Zhao et al. 2017) presented further evidence of Pc 3 waves generated by a large HFA. In their multiple spacecraft observations as shown in the schematic in Fig. 57, a large HFA was observed upstream from the bow shock on the dawn side, and long-lasting, nearly monochromatic Pc 3 waves were generated in the magnetosphere immediately after the HFA passage and propagated dawn-to-dusk. Coordinated observations from ground-based magnetometers in Canada (noon), Alaska (dawn), and Japan (nightside) confirmed the occurrence of the Pc 3 waves and their day-to-night propagation direction.

Several studies also show that foreshock transients can generate ULF waves in the Pc 5 band. Hartinger et al. (2013) presented the first observation of global Pc 5 wave activities in response to a high-speed solar wind interval associated with three types of foreshock transients, including HFAs, foreshock bubbles, and the motion of foreshock compressional boundaries. In their observations, global Pc 5 waves were excited, and the strongest waves occurred at the local time correlated with the location of the ion foreshock with amplitudes as large as 10 nT and comparable to storm-time wave activities. They concluded that foreshock transients could be an important energy source for global Pc 5 waves in the magnetosphere.

In a recent study by Shen et al. (2018) using both in-situ and ground-based observations, localized Pc 5 wave activities were reported in response to a foreshock transient event, in which there is a dawn-dusk asymmetry of the wave activity and Pc 5 waves were present only in the same local time sector as the foreshock transient was. Previous statistical studies have shown that the occurrence of Pc 5 waves exhibits a distinct dawn-dusk asymmetry, which was attributed to KH instabilities at the magnetopause (e.g., Chisham and Orr 1997). This new observation demonstrated that foreshock transients could also be an important energy source for localized Pc 5 wave activities with dawn-dusk asymmetry.

In addition to the aforementioned studies, Hietala et al. (2012) reported irregular ULF wave activities in response to the effects of pressure pulses associated with localized magnetosheath jets. These magnetosheath jets originated from the quasi-parallel bow shock under steady solar wind conditions. They found that the jets caused irregular pulsations at geosynchronous orbit. Additionally, Gillis et al. (1987) found that there was a correlation between FTEs and transversely polarized Pc 4 pulsations. A case study by Glasmeier et al. (1984) suggested that an FTE triggered Pc 5 pulsations. Recent observations by Bentley et al. (2018) also showed that FTEs can be a possible source of magnetospheric ULF waves.

Although there are many theoretical and numerical simulation studies attempting to understand the interaction of solar wind pressure pulses with the magnetosphere (e.g., Zhu and Kivelson 1988; Lee and Lysak 1989), mechanisms for wave excitation by localized pressure perturbations have not been fully explored. The pressure perturbations associated with foreshock transients are generally localized and moving along the magnetopause whereas solar wind pressure pulses interact with the entire magnetopause. Lin and Wang (2005) simulated the linkage between foreshock waves that involve self-consistently generated foreshock cavities and ULF waves in the magnetosphere using a 3D global-scale hybrid simulation for a case with the radial IMF. The simulation domain contains the dayside bow shock/foreshock, magnetosheath, and the magnetosphere, with the foreshock waves generated self-consistently in the interaction between the solar wind and the magnetosphere. Foreshock cavities are generated in the turbulent quasi-parallel shock, and they subsequently interact with the magnetopause as pressure pulses. The coupling between the pressure pulses/compressional waves and the magnetosphere leads to the mode conversion to shear Alfvén waves and kinetic Alfvén waves. As a result, field line resonances corresponding to the fundamental odd resonance wave number are found to be generated in the magnetosphere in this case in which the source of perturbations is near the nose of the magnetopause. Shi et al. (2021) illustrated the properties of self-consistently generated field line resonances through direct mode conversion in magnetospheric response to the foreshock disturbances (see Sect. 3.3.2 for details). In addition, based on the 3D global hybrid simulation, Wang et al. (2009) has shown a connection between the foreshock waves and the ULF waves in the cusp. Further simulations are required to fully understand the ULF waves in the magnetosphere associated with the foreshock structures.

#### Responses of Tail Plasma Sheet

FTEs can essentially transfer geomagnetic flux from dayside to nightside and contribute to accumulation of magnetic energy in the magnetotail, which forms the basis of activities in magnetospheric substorms or the Dungey cycle, and thus can be geoeffective. Their overall significance, however, depends largely on how FTEs are generated (Sect. 3.2) and how FTE flux tubes interact with each other during the course of their tailward transport. This is because what matters from the viewpoint of geoeffectiveness is the total amount per unit time of open flux transferred to the tail, which depends on the duration or continuity of magnetopause reconnection, extent and number of the relevant X-line, and magnetic topology of FTE flux tubes. All these factors are different for different models of FTE formation and interaction (e.g., Fear et al. 2017; Hasegawa et al. 2010; Øieroset et al. 2019), and it is not until recently that the interaction processes have been revealed using the state-of-the-art MMS observations (e.g., Kacem et al. 2018). Thus, the geoeffect of FTEs needs to be further explored in the future not only using high-resolution in situ measurements but also in conjunction with low-altitude, ground-based, and/or imaging observations, while FTEs at Mercury appear to be significant enough to drive the substorm cycle in the Hermean magnetosphere (Fear et al. 2019).

The magnetopause KHI could also be geoeffective through its role in the mass and momentum transport of the solar wind into the plasma sheet especially under northward IMF. In particular, Sorathia et al. (2019) demonstrated, based on a global MHD model combined with test particle simulation, that the particle transport rate ${\sim} 10^{26}\mbox{ s}^{-1}$ across the flank magnetopause resulting from the KHI is consistent with observations. They further showed that lower entropy flux tubes created by the KHI can be subject to interchange instability, a possible mechanism of filling the central plasma sheet with cold magnetosheath plasma. It should also be pointed out that weak sunward streams of cold and dense plasma in the plasma sheet, as observed by Geotail (Fujimoto et al. 1998), cannot be explained by high-latitude magnetopause reconnection and resultant convection alone (Song et al. 1999; Hasegawa 2012). They may result from KHI-induced interchange instability as reported by Sorathia et al. (2019) or turbulent flow as simulated by El-Alaoui et al. (2012).

We note that geomagnetic storms preceded by an extended period of northward IMF tend to be more intense than those with no such preceding northward IMF (Lavraud et al. 2006a), indicating the importance in the storm development of the cold-dense plasma sheet formed under northward IMF. Lavraud and Borovsky (2008) also show that coronal mass ejections, one of the drivers of storms, can produce magnetosheath conditions favorable for the KHI growth through their interaction with the magnetosphere. Thus, it is possible the KHI indirectly contributes to an intensification of one of the most geoeffective phenomena via the formation of the dense plasma sheet.

Sibeck (1990) proposed a model for the interaction between a dynamic pressure pulse and the magnetosphere based on the force balance between the transient solar wind and the magnetopause/magnetosphere, in which a single or double flow vortex inside the magnetosphere near the magnetopause was predicted. The ionospheric counterpart of the vortices was observed using SuperDARN radar and ground magnetometers (e.g., Sibeck et al. 2003). Global MHD simulations showed this single vortex in the dawn and dusk magnetosphere (Wang et al. 2010; Samsonov et al. 2010; Samsonov and Sibeck 2013). Samsonov and Sibeck (2013) interpreted this vortex formation as the compressional wave penetration and reflection in the magnetosphere boundaries. Shi et al. (2014) reported the first in-situ observations of a vortex related to a sudden increase of solar wind dynamic pressure. During this event, THEMIS-B, C, and D, located near the dusk flank of the magnetopause, observed an anti-clockwise rotating vortex. Equivalent ionospheric currents (EICs) derived from the THEMIS ground magnetometer array show a vortex with counterclockwise rotation around the foot points of THEMIS B, C, and D. Zhao et al. (2016b) reported an event during which a negative solar wind dynamic pressure change also induced a flow vortex inside the magnetosphere. By comparing observations from Zhao et al. (2016b) and Shi et al. (2014), one can find that the rotation sense of the vortex induced by a negative pressure pulse is opposite to that by a positive pressure pulse.

**Fig. 58:**
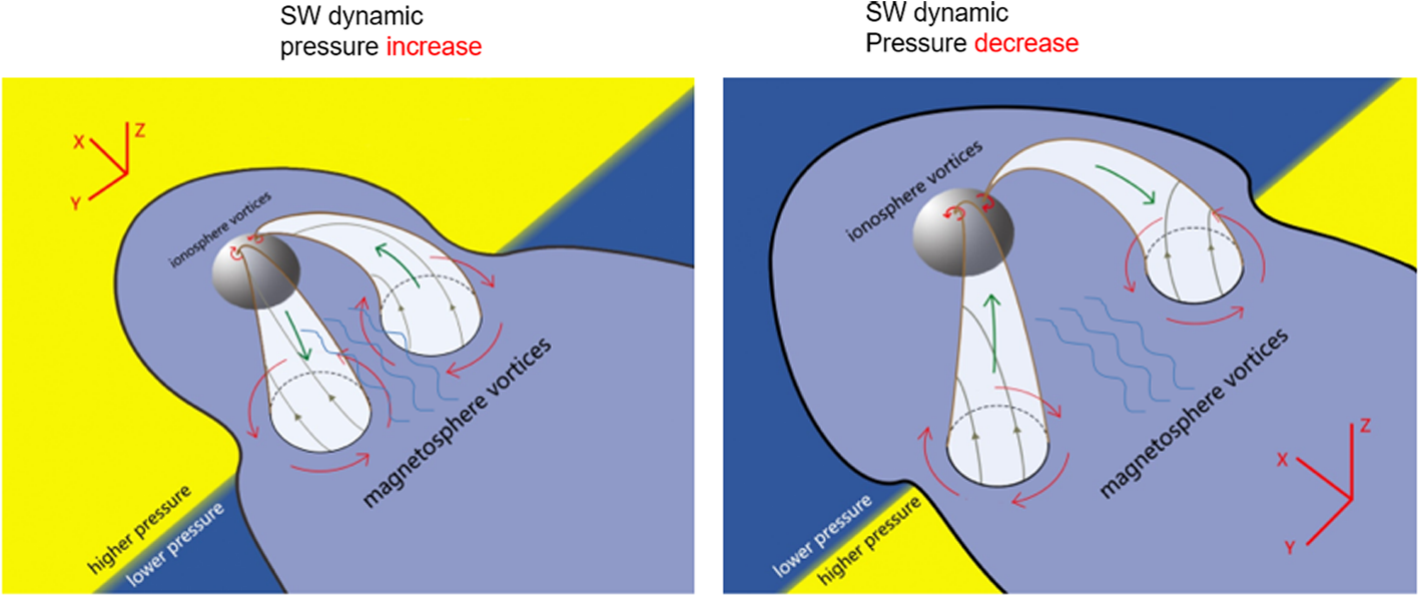
An illustration of the magnetospheric responses (vortex, ULF wave and FAC) for a) positive and b) negative pressure pulses (from Shi et al. 2020b)

A global picture is illustrated in Fig. 58. A change of the solar wind dynamic pressure generated two vortices, one on the dawn side and the other on the dusk, and these vortices propagate toward the tail in line with the pressure change front motion of the solar wind. The rotation sense is opposite for the dawn and dusk (clockwise on the dawn side and counterclockwise on the dusk for the pressure increase situation, and opposite for the pressure decrease situation). The two vortices can be connected to the ionospheric vortices by field-aligned currents (FACs). The FACs are in line with the region I FAC sense for the pressure increase situation (downward on the dawn side and upward on the dusk), and consistent with the Region II FAC sense for the pressure decrease situation (upward on the dawn side and downward on the dusk). The vortex is related to ULF waves and auroras sometimes. Considering the recent studies by Tian et al. (2016) and Ozturk et al. (2019), this global picture could also be revised and become complicated.

**Fig. 59:**
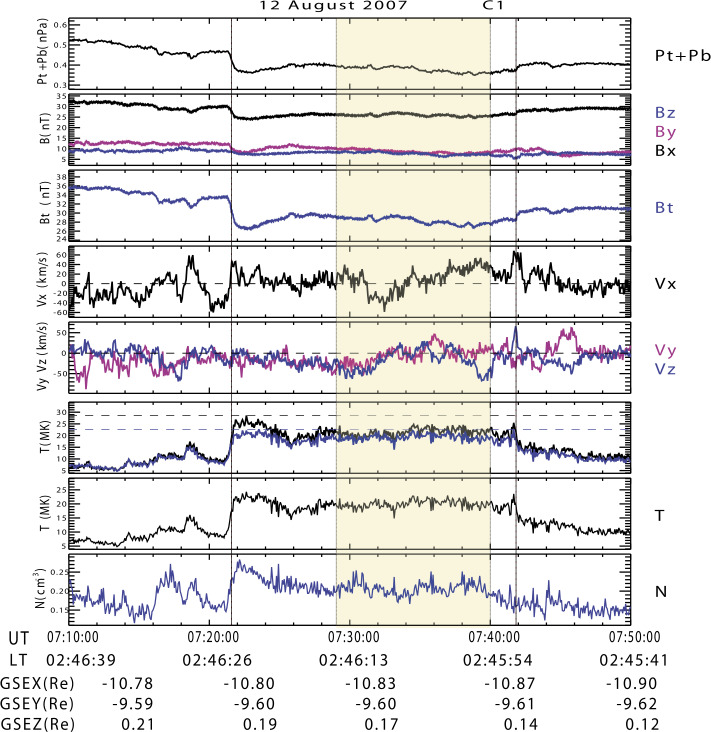
An overview plot of Cluster observations showing the magnetotail response to an extreme HFA. From top to bottom are the total pressure (the sum of the thermal pressure and the magnetic pressure), the magnetic field in GSE coordinates, the magnetic field strength, the plasma velocity in GSE coordinates, the perpendicular and parallel ion temperature, and the ion density. The time interval of the HFA observed by THEMIS is marked by two vertical lines, and the yellow shaded region indicates a vortex-like plasma structure. (Zhang and Liu, submitted to JGR)

The magnetotail response to foreshock transients is largely uncharted territory. Below we show an example of the magnetotail response to an extreme HFA reported by Zhang and Liu (submitted to JGR). The HFA with extremely low solar wind dynamic pressure inside caused the local bow shock to move outward, leading to the magnetopause outward motion by several $R_{\mathrm{{E}}}$ as observed by the THEMIS spacecraft (Zhang and Liu, submitted to JGR). The Cluster spacecraft in the magnetotail monitored the corresponding magnetotail response (Fig. 59).

Characterized by sharp changes in the plasma temperature, density, and magnetic field (Fig. 59), Cluster rapidly entered and exited the center of the magnetotail plasma sheet. The possible process is that due to the low dynamic pressure associated with the extreme HFA, the magnetopause moved outward significantly, resulting in the fast expansion of the magnetotail. As the HFA passed by, the magnetopause recovered/moved backward, and the magnetotail correspondingly contracted. As a result, Cluster, steadily located in the dawnside magnetotail, temporarily observed the magnetotail plasma sheet. Using the timing method with all four Cluster spacecraft observations, the expansion speed of the magnetotail was determined as ∼50 km/s.

Moreover, the Cluster spacecraft also observed a plasma flow vortex structure in the magnetotail plasma sheet as seen from the rotation of the plasma flow $V_{x}$ component (yellow shaded in Fig. 59). It could be generated by the inhomogeneous dynamic pressure associated with the extreme HFA impinging on the magnetosphere. Such a vortex structure as well as the expansion and contraction of the magnetotail could excite ULF waves contributing to further geoeffects. Therefore, it is important to understand the magnetotail response to foreshock transients. Additionally, foreshock transients can propagate to the midtail foreshock and disturb the local bow shock, magnetosheath, and magnetopause (Wang et al. 2018a, 2020b; Liu et al. 2020e), which can also potentially result in magnetotail disturbances.

### Effects in the Ionosphere and Thermosphere

#### Ground Magnetic Response

Long prior to the beginning of the space age, researchers employed ground magnetometers to infer the occasional occurrence of abrupt increases in the solar wind dynamic pressure that caused simple step-function enhancements of the magnetic field strength on the surface of the Earth on a global basis (Chapman and Ferraro 1931). Signatures at higher latitudes near the auroral oval were found to be more complicated, often indicating bipolar north/south variations (Matsushita 1957). The complication arises because the signatures at higher latitudes result from both fast and intermediate mode waves transmitted into the magnetosphere. The fast mode waves travel across magnetic field lines while the intermediate mode waves travel along magnetic field lines (Wilson and Sugiura 1961). With the help of extended arrays of ground magnetograms, it became clear that these magnetic impulse events could often be organized into azimuthally traveling convection vortices (Friis-Christensen et al. 1988).

Analytical models successfully predicted the organization of the magnetometer event signatures into single or pairs of vortices (Southwood and Kivelson 1990; Glassmeier and Heppner 1992; Sibeck et al. 2003). Zhu et al. (1997) simulated the ground magnetic field signatures of traveling convection vortices including both ionospheric conductivity enhancements and ground induction effects. They found that including the localized conductivity enhancement can cause a significant distortion of the current system and consequently the ground magnetic field disturbance. Events with enhanced hard precipitation are likely to exhibit this kind of distortion. As predicted by the models, events are conjugate in both hemispheres when there are no asymmetries in the ionospheric conductivity or magnetic field, yet asymmetries in the ground magnetic response can occur when these conditions are not met (Kim et al. 2015). Hartinger et al. (2017) compared the inter-hemispheric responses seen during one of the Kim et al. (2015) events with the predictions of a series of simulations with different conductivity profiles and magnetic field topologies. They found that asymmetries (e.g., in amplitude) varied with the conductivity/distortions in the magnetic field.

The dynamic pressure variations associated with transient events created by kinetic effects within the Earth’s foreshock are equal to or greater than those associated with intrinsic solar wind dynamic pressure variations, and far more common, albeit less spatially extended. Consequently, one might expect a large fraction of the largest amplitude perturbations seen in both high- and low-latitude ground magnetograms to be associated with foreshock events. In the absence of in situ measurements made within the foreshock itself, the occurrence patterns of the events seen in ground magnetometers should reflect those for foreshock transient events, i.e. they should occur during intervals of low solar wind density and dynamic pressure, high solar wind velocity, be associated with intervals of radial IMF orientation that place the foreshock upstream of the dayside magnetopause (Sibeck et al. 1989a,b; Fairfield et al. 1990) or in conjunction with abrupt changes in the IMF orientation that lead to the formation of hot flow anomalies. Case and statistical studies reveal the association between the ground magnetometer events with abrupt changes in the IMF orientation and the fact that they attain largest amplitudes and tend to occur prior to local noon (Sibeck and Korotova 1996). With the help of in situ measurements of the solar wind within the foreshock, it quickly became clear that perturbations associated with kinetic events in the foreshock drive the vast majority of traveling convection vortices (Murr and Hughes 2003), precisely as does the association of transient events at high latitudes on the ground with sudden impulse signatures at lower latitudes and geosynchronous orbit (Sibeck 1993).

#### Riometer Response

Transient compressions of the dayside magnetosphere such as those associated with foreshock transients adiabatically energize magnetospheric particle populations, resulting in distributions that peak more strongly at pitch angles nearly perpendicular to the magnetic field. Just as in the case of the compressions caused by intrinsic solar wind pressure variations (Perona 1972), the resulting ion and electron distributions are unstable to the growth of EMIC (for ions) or ELF/VLF (for electrons) plasma waves that can scatter the particles and cause their precipitation into the ionosphere. Precipitating electrons with energies greater 30-300 keV enhance ionospheric densities, making the ionosphere opaque to the arrival of cosmic noise. Consequently, we expect the transient events seen in high latitude dayside ground magnetograms associated with magnetospheric compressions produced by foreshock kinetic events to exhibit equally transient increases in cosmic noise absorption. Korotova et al. (1999) reported the results of a statistical study of cusp latitude South Pole Station observations indicating that this is precisely the case. About 80% of 153 magnetic impulse events were associated with a small enhancement in cosmic noise absorption. Like magnetic impulse events, the occurrence rate of events with enhanced absorption exhibits a large peak in the pre-noon ionosphere and a smaller peak in the post-noon ionosphere.

#### Aurora Signatures

*Auroras associated with foreshock transients*

Dayside transients associated with dynamic pressure changes can compress or expand the magnetosphere, which may enhance or weaken the scattering of magnetospheric particles into the ionosphere and thus modify the aurora intensifications. Large scale interplanetary transients like shocks or positive/negative dynamic pressure changes can invoke a global response of auroras (e.g., Tsurutani et al. 2001; Zhou et al. 2003; Sato et al. 2001; Liou et al. 2006). Unlike those large-scale solar wind changes, foreshock transients are localized and short-lived. Hence the aurora response to them is not frequently detected. Using Polar Ultraviolet Imager, Sibeck et al. (1999) reported that an aurora brightening following an HFA appeared first on the afternoon side and then on the morning side. The different auroral luminosity in the prenoon, local noon and postnoon sectors suggests the deformation of the dayside magnetosphere caused by the HFA. Observations of traveling convection vortices and pre-noon auroral brightening due to an HFA were also reported by Fillingim et al. (2011).

**Fig. 60:**
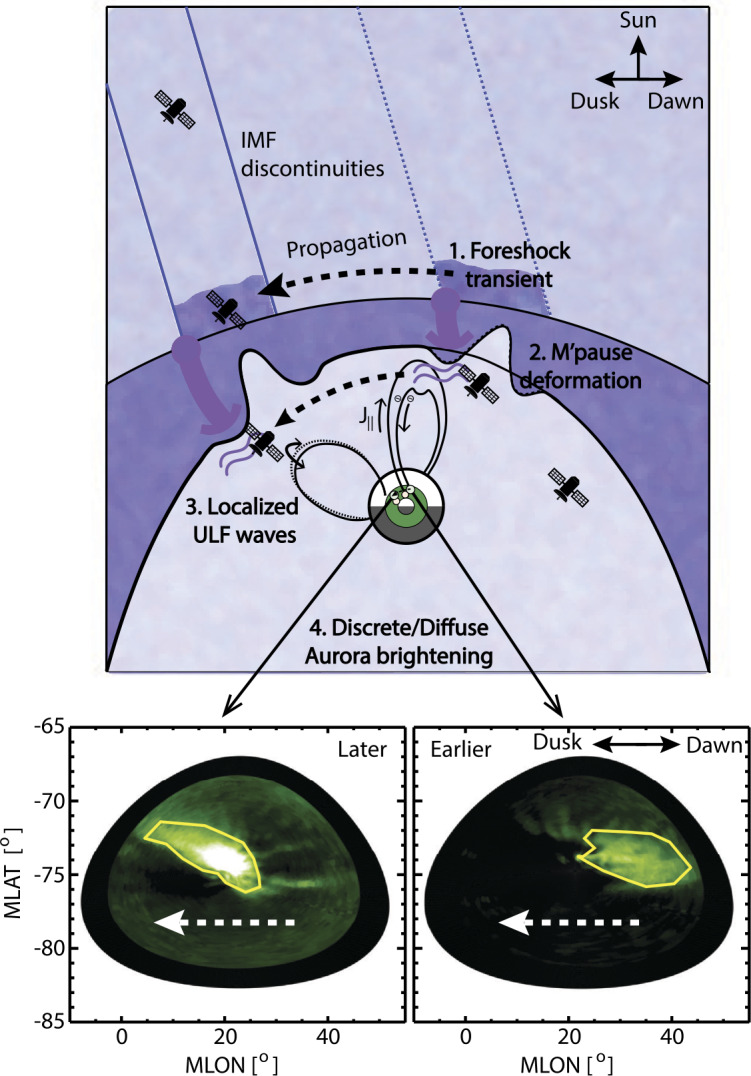
The impact of one foreshock transient on the Earth’s magnetosphere as seen by multi-satellites. Foreshock transients generated localized compressional waves with clear dawn-dusk asymmetry via disturbing the magnetopause within a small spatial size, generating localized field line resonances, field aligned current and moving aurora signatures. (After Shen et al. 2018; Wang et al. 2018c)

By using multi-satellite observations, Shen et al. (2018) studied an event of foreshock transient-generated ULF waves with a clear dawn-dusk asymmetry in the wave power. Wang et al. (2018c) presented duskward propagating auroras due to this foreshock transient from high-resolution 2D imaging of all-sky imager at South Pole on the dayside. A schematic plot is shown in Fig. 60. The field-aligned mapping of the aurora imaging into the magnetosphere was used to determine the size (a few $R_{\mathrm{{E}}}$ in $Y$) and propagation (duskward) of the magnetopause disturbance caused by the foreshock transient. Equivalent currents deduced from ground magnetometers show a pair of FACs and the duskward propagation is consistent with that of the auroras.

*Auroras associated with KH waves*

In general it is difficult to establish a 1 to 1 identification of an auroral signature with a corresponding event that is observed at the magnetospheric boundary because of the rather small size of the corresponding ionospheric footprint of the event, proper timing, and uncertainties in the mapping of the magnetic field from the magnetopause to the ionosphere. A well established theoretical effect of magnetopause processes is their influence on global magnetospheric convection which is identified through radar and low altitude satellite observations and measured in terms of a cross polar cap potential. This potential responds most strongly to magnetic reconnection for southward IMF $B_{z}$ with typical values of 50 to 100 kV while viscous interaction believed to be caused mostly by KH boundary waves is typically 10 to 20 kV for northward $B_{z}$. Here we focus on auroral signatures associated with KH waves. Auroras associated with FTEs are discussed in Sect. 4.2.4 since they are tied to ionospheric flow channels.

It has long been suspected that Pc 5 waves on the dayside might be related to KH waves at the magnetospheric boundary (Walker and Greenwald 1981). Lui et al. (1989) interpreted initial observations of bright auroral spots in the dayside afternoon sector as signatures of KH waves from the low latitude boundary layer, and Rostoker et al. (1992) provided further evidence of this interpretation through the associated observation of Pc pulsations and quantitative estimates. Similarly Farrugia et al. (1994) concluded that the most likely explanation of the auroral forms are KH waves. Based on numerical simulation Wei and Lee (1993) demonstrated quantitative consistency of these waves with KH waves and illustrated that the associated field-aligned current of the waves is upward in the afternoon sector consistent with downward electron precipitation. In the morning sector, spatially quasiperiodic plasma and electromagnetic signatures, as well as pulsating auroral arcs accompanied by pulsations mostly in the Pc 5 range also map to the flank boundary layer and have been associated with KH waves as the most likely source (Hirahara et al. 1998; Famigia et al. 2000). Morning side pulsations were also found by Ohtani et al. (1999) but they interpreted the origin as KH waves at the magnetopause rather than the inner edge of the boundary layer. For northward IMF, Polar UVI observations demonstrate that auroral intensity varies linearly with solar wind ram pressure indicating that viscous coupling likely through KH waves increases with solar wind velocity (Zhou and Tsurutani 2004). Similar properties were found when interplanetary shocks or solar wind pressure pulses cause fast moving auroral streaks or rays at velocities consistent with the propagation of KH waves at the magnetopause (Zhou et al. 2017b). In terms of ground magnetic field perturbations, Weigel et al. (2003) demonstrated that their amplitude is primarily determined by magnetopause reconnection while their time derivative is mostly caused by KH waves at the magnetospheric boundary.

*Throat aurora*

During a study on dayside diffuse aurora based on optical observations in the green line (557.7 nm), Han et al. (2015) noticed that, when a convection-aligned stripy diffuse aurora was contacting with the discrete aurora oval, a north-south-aligned discrete auroral form was often observed. This particular auroral form was named “throat aurora”, because it was only observed around the ionospheric convection throat region. It has been confirmed that throat auroras are caused by precipitation of magnetosheath particles (Han et al. 2016) and are the ionospheric signature of magnetopause indentations (Han et al. 2018). Han et al. (2017a) reported that the spatial scale of the magnetopause indentation can be as large as ∼2.0-3.0 $R_{\mathrm{{E}}}$ after mapping a throat aurora to the geomagnetic equatorial plane and the daily occurrence rate of throat aurora is higher than ∼50%, which implies that magnetopause indentations with considerable spatial scales should commonly exist on the subsolar magnetopause.

**Fig. 61:**
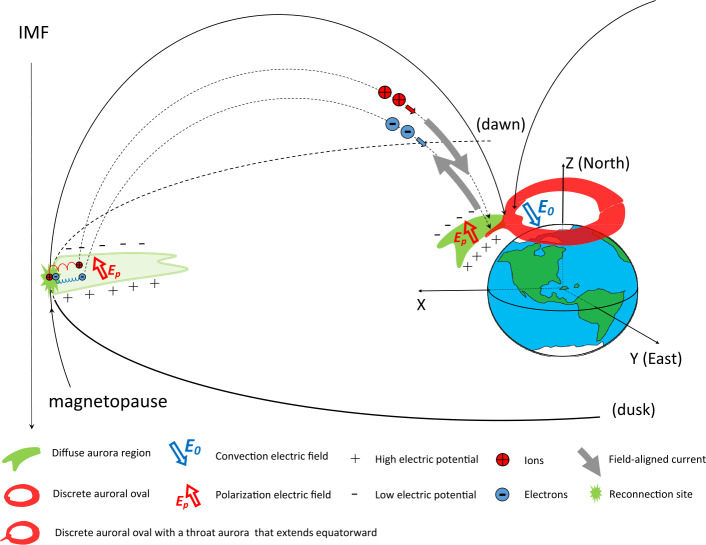
A conceptual model of throat aurora. The red circle over the north pole indicate the discrete aurora oval. A throat aurora in north-south direction bulge out from the discrete aurora oval toward low latitude near local noon. A stripy diffuse aurora is indicated by the green region, which will cause a polarization electric field $\mathbf{E}_{p}$. The model suggests that this electric field can map to the magnetosphere and affect the magnetopause reconnection to produce the throat aurora. (From Han 2019)

While considering the generation of throat auroras, Han et al. (2017a) noticed that throat auroras were closely involved with diffuse aurora and their orientations were convection-aligned. Because both diffuse aurora and convection are linked to magnetospheric internal phenomena, the occurrence of throat aurora was suggested to be affected by factors inside the magnetosphere, such as the ionospheric feedback effect. At the same time, Han et al. (2017a) noticed that the occurrence rate of throat aurora showed a clear dependence on the IMF cone angle, which suggested that some transient processes outside the magnetosphere, such as magnetosheath jets, might be a driver for throat auroras (Han et al. 2017a; Plaschke et al. 2018a). In order to explain why the generation of throat aurora depends on factors either inside or outside the magnetosphere, Han (2019) proposed a model that is schematically illustrated in Fig. 61, The model suggests that precipitation of a stripy diffuse aurora can lead to ionospheric conductivity enhancement and thus produce a polarization electric field in the ionosphere. After this electric field mapping to the magnetosphere along closed field lines, it may guide a magnetopause reconnection to develop inward the magnetosphere and result in a throat aurora. Some observational supports for the model were presented in Han (2019). This model can explain the occurrence dependence of throat aurora on either inside or outside factors, because two types of diffuse auroras existing near magnetic local noon are related with inside and outside factors, respectively (Han et al. 2017b). Furthermore, using coordinated observations from THEMIS and all-sky camera observations at South Pole station, Wang et al. (2018b) showed that magnetosheath jets were well associated with the localized diffuse auroral brightening observed at South Pole station near local noon. This provides further support to the model of Han (2019). According to this model, the outside factors (i.e., impulsive events in the magnetosheath like magnetosheath jets) may not drive throat aurora directly, but produce diffuse aurora first and then affect the magnetopause reconnection and result in throat aurora. The cause of throat aurora is likely strongly enhanced magnetopause reconnection forced by foreshock or magnetosheath transients.

#### Ionospheric Flow Response

*Ionospheric flow response to foreshock transients*

Dayside foreshock transients, such as HFAs, can also trigger ionospheric flow response. Sitar et al. (1998) identified traveling convection vortices (TCVs) in the ground magnetometer data that were associated with the field-aligned currents and propagating tailward. Meanwhile, incoherent scatter radar observed strong modulation in the ionospheric plasma flow strength and direction. POLAR UVI observed a localized intensification of aurora emissions due to an upward field-aligned current. DMSP observations indicated that the field-aligned currents that drove TCVs were mapped to the boundary plasma sheet-low latitude boundary layer interface. Based on the coordinated set of observations, Sitar et al. (1998) suggested that the driver was the magnetopause deformation caused by an HFA (Sibeck et al. 1998).

Later observations confirmed this scenario. Using multi-point THEMIS observations, Jacobsen et al. (2009) demonstrated significant magnetopause outward deformation caused by an HFA, which propagated tailward. TCVs were observed by ground magnetometers. Using THEMIS GMAG and POLAR UVI observations, Fillingim et al. (2011) showed a TCV and auroral brightening driven by an HFA that was detected by THEMIS spacecraft. Similarly, Wang et al. (2018c) also reported observations of TCVs and auroral brightening driven by a foreshock transient. Additionally, Liu et al. (2020e) showed a TCV which might be driven by an HFA in the nightside midtail foreshock.

*Ionospheric flow response to FTEs*

An important mechanism for transfer of flux from the solar wind to the magnetosphere is impulsive dayside reconnection and FTEs (Cowley and Lockwood 1992; Lockwood et al. 1995). FTEs were first discovered by Haerendel et al. (1978) and Russell and Elphic (1978, 1979). FTEs have a typical scale size of one $R_{\mathrm{{E}}}$ in the boundary normal direction at the magnetopause (Saunders et al. 1984). In the ionosphere that corresponds to around 100-200 km along the meridian (Southwood 1985, 1987). A reconnection burst propagates from the magnetopause to the ionosphere as an Alfvénic disturbance with a system of associated field-aligned currents (Glassmeier and Stellmacher 1996).

In the dayside aurora poleward-moving transients have often been interpreted as FTE signatures (Sandholt et al. 1990, 1993; Denig et al. 1993; Moen et al. 1995; Milan et al. 1999, 2000; Thorolfsson et al. 2000). The poleward moving auroral forms (PMAFs) can occur for both southward and northward IMF although FTEs are strongly related to southward IMF conditions, which indicates that FTEs may not have a simple 1 to 1 correspondence to PMAFs (Fasel 1995). In the northern hemisphere the PMAFs move west (east) for positive (negative) IMF $B_{y}$ (Sandholt et al. 1998, 2004a). Milan et al. (1999) and Thorolfsson et al. (2000) associated PMAFs with plasma flow. Later, Oksavik et al. (2004) used the EISCAT Svalbard Radar to identify the relative location of PMAFs and their associated flow channels.

The first ionospheric radar observations of FTE signatures were made several decades earlier by Van Eyken et al. (1984) and Goertz et al. (1985). In the following decade there were frequent reports of poleward-moving transients in literature, which depending on their specific characteristics, were called flow channel events (FCEs) (Pinnock et al. 1993, 1995; Chisham et al. 2000; Neudegg et al. 1999, 2000), pulsed ionospheric flows (PIFs) (Provan et al. 1998, 2002; Provan and Yeoman 1999; McWilliams et al. 2000), or poleward-moving radar auroral forms (PMRAFs) (Milan et al. 2000; Davies et al. 2002; Rae et al. 2004). These dayside transients are often related to each other (Wild et al. 2001) and show repetition rates (e.g., Milan et al. 1999) that are comparable to FTEs at the magnetopause. Neudegg et al. (2000) pointed out a good correspondence between FTEs at the magnetopause and discrete flow channels in the ionosphere. Provan et al. (1998, 2002) investigated the pulsed plasma flows poleward of the convection reversal boundary.

In an effort to summarize the different categories of plasma flow, Sandholt et al. (2004b) listed three key flow channels in the daytime aurora: (1) enhanced sunward return flow on closed field lines (Lockwood et al. 1993; Moen et al. 1995, 2006), (2) enhanced flow on newly open flux containing FTEs (FCEs and PIFs), and (3) enhanced flow on old open field lines due to the solar wind magnetosphere dynamo in the high-latitude boundary layer (Stern 1984; Sandholt et al. 2004b; Farrugia et al. 2004; Sandholt and Farrugia 2007). All three categories give enhanced flow in the same direction as the anti-sunward large-scale plasma flow in the polar cap.

Afterwards, a fourth category of plasma flow was added (Rinne et al. 2007; Moen et al. 2008; Oksavik et al. 2011). Rinne et al. (2007) named it a reversed flow event (RFE), because the enhanced flow was observed in the opposite direction of the general background convection, i.e. it had the effect of reversing the flow. This work was based on a series of publications that used the European Incoherent Scatter (EISCAT) Svalbard Radar (ESR) in fast scan modes to investigate flow channels in the cusp region (Carlson et al. 2004; Oksavik et al. 2004, 2005; Rinne et al. 2007, 2010; Moen et al. 2008). Rinne et al. (2007) defined the flow speed inside the RFE to be $>250$ m/s for the event to qualify as an RFE. The rationale was to highlight events that clearly stood out from the background convection. Moen et al. (2008) reported that RFEs can last for 10-20 min. Rinne et al. (2007) observed RFEs 40% of the time within one hour of magnetic noon, with no apparent preference for IMF $B_{y}$ or $B_{z}$ polarity, but with a clear preference for clock angles between $40^{\circ }$ and $240^{\circ }$ (i.e. $|B_{y}| > |B_{z}|$). Oksavik et al. (2004, 2005) pointed out that clockwise vorticity is consistent with an upward Birkeland current (i.e., intense aurora) on one side of a flow channel, while counterclockwise vorticity is consistent with a downward Birkeland current (i.e., weak aurora) on the other side of the flow channel. Moen et al. (2008) pointed out that the RFE phenomenon is not uniquely related to PMAFs but appears to be a specific feature of Birkeland Current Arcs (BCAs). Moen et al. (2008) suggested that the RFEs may be generated in two ways: (1) the RFE channel can be a region where two magnetosphere-ionosphere (MI) current loops, forced by independent voltage generators, couple through a poorly conducting ionosphere, or (2) the RFE channel can be the ionospheric footprint of an inverted-V-type coupling region. It remains unclear if one dominates, or if both mechanisms are closely related.

Dayside transients are known to cause significant space weather effects (Moen et al. 2013). Moen et al. (2004) presented a one-to-one relationship between PMAFs and ion upflow events in the cusp ionosphere. For southward IMF there are also frequent reports of polar cap patches in the polar ionosphere (Crowley 1996; Tsunoda 1988; Basu and Valladares 1999; Dandekar and Bullett 1999). Polar cap patches are 100-1000 km wide islands in the horizontal direction (Weber et al. 1984), where the local F-region electron density can be 2-20 times larger than the surrounding plasma (Buchau et al. 1983; Weber et al. 1984; Crowley et al. 2000).

Most patches originate at lower latitudes, where photoionization from solar EUV radiation increases the plasma density (Foster 1993). Three groups of mechanisms have been suggested for formation and structuring of polar cap patches (Moen et al. 2006). The first one is formation via transient reconnection or FTEs (Lockwood and Carlson 1992; Carlson et al. 2002, 2004, 2006), where the open-closed polar cap boundary leaps equatorward to a lower latitude high-density plasma reservoir, followed by a relaxation of the boundary, which transports high-density plasma into the poleward flow. The second one is enhanced plasma depletion between patches due to enhanced recombination of plasma from enhanced ion-frictional or Joule heating due to rapid plasma drift in short-lived east-west flow channels in the cusp ionosphere (Valladares et al. 1996). The third one is changes in the plasma convection pattern, especially due to IMF $B_{y}$ reversals, that alternate the intake of high density (postnoon) and low density (prenoon) plasma and create isolated polar cap patches that drift into the dayside polar cap (Anderson et al. 1988; Sojka et al. 1993; Rodger et al. 1994). Walker et al. (1999) suggested that the soft particle precipitation in the cusp region may also cause significant ionization that can result in F-region plasma that drift into the polar cap. Oksavik et al. (2006) added that particle precipitation inside lobe convection cells may also lead to the formation of polar cap patches in the polar cap during northward IMF.

For southward IMF the polar cap patches can travel all the way across the polar cap with the large-scale ionospheric convection (Foster 2005; Oksavik et al. 2010; Van Der Meeren et al. 2014). While in transit, the polar cap patches are known to disrupt radio communication and navigation systems (Basu et al. 1990, 1998). Intense PMAF activity can also cause significant phase scintillation and loss of signal lock for satellite navigation signals (Oksavik et al. 2015). Recombination of the high-density plasma leads to weak 630.0 nm emissions that can be observed as airglow patches (Weber et al. 1984; Hosokawa et al. 2014, 2016, 2019). On the nightside the airglow patches leave the polar cap and enter the nightside auroral oval (Lorentzen et al. 2004; Moen et al. 2007; Van Der Meeren et al. 2015). Nishimura et al. (2014) also followed a PMAF/airglow patch that moved across the polar cap from the dayside to the nightside, where it resulted in a poleward boundary intensification (PBI). It shows that dayside transients may ultimately also play a key role in triggering localized nightside reconnection and flow bursts in the nightside plasma sheet.

An open question for all FTE models is the length of the X lines and the amount of magnetic flux actually transported in FTEs. Milan et al. (2016) used optical and SuperDARN data to examine two events to show that their ionosphere extent was very different (2 vs. 7 hours local time). Oksavik et al. (2005) presented a comprehensive set of ground-based, ionospheric, and low-altitude satellite observations surrounding a PMAF, and confirmed that narrow flow channels consistent with small-scale twin convection cells and associated field-aligned currents were observed along with the PMAF. They estimated that the open magnetic flux transferred by the poleward flow channel is of the order of 1 MWb, which is consistent with a narrow flux bundle created by localized and transient reconnection. Other ground-based observations (Fear et al. (2017); and references therein), however, show that in very extreme cases the flux content of each FTE can be as large as 50 MWb, that is about 10% of the open flux in the polar cap. These observations indicate that the reconnection line responsible for some FTEs is long, and no single FTE formation mechanism can account for a variety of FTEs as observed.

Fear et al. (2017) also argue that when the reconnection line associated with FTEs is extended, the magnetic flux transferred by each FTE should not be estimated as the product of the field component along the FTE flux tube/rope axis and the cross sectional area of the FTE, as often done in the literature (e.g., Slavin et al. 2010). An accurate estimate is in fact given by the product of the field component normal to the magnetopause ($B_{n}$) within the FTE, the radius of the flux rope ($r$), and the length of the flux rope ($L$): $\Phi =B_{n}rL$. Fear et al. (2012a) analyzed the axial direction of a series of FTEs observed by the Cluster spacecraft during a magnetopause crossing of high magnetic shear in order to constrain the possible formation mechanisms. They found that the FTE axes are all oriented in the dawn-dusk direction, consistent with the processes involving relatively long reconnection lines (extended single or multiple X line reconnection) rather than localized, transient reconnection.

#### Thermospheric Heating

As noted in Sects. 4.2.1 and 4.2.4, transient compressions of the dayside magnetopause and magnetosphere launch field-aligned currents that carry transient electric fields along the magnetic field lines to the high-latitude dayside ionosphere. Here the currents and electric fields drive traveling convection vortices associated with currents and plasma flow in the ionosphere. The compressions can create or enhance anisotropies in magnetospheric plasma populations that lead to the growth of plasma waves which can in turn scatter some of the magnetospheric particles into the loss cone, causing them to precipitate into the high latitude ionosphere. The transient magnetopause compression can also heat and expand the high-latitude thermosphere. Enhanced particle precipitation ionizes neutral atmosphere, increasing the ionospheric plasma density. As the result, more ionospheric plasmas experience the fast ${\mathbf{E}} \times {\mathbf{B}}$ drift at a speed up to several km/s and collide with the slow thermospheric neutrals at a typical speed of ∼100 m/s. The neutrals and plasmas exchange energy and momentum through this collisional process, resulting in the enhancements of the neutral wind and the high-altitude thermospheric density. Some, perhaps most, of the frequently observed transient magnetopause compressions and traveling compression vortices are associated with kinetic structures generated within the foreshock. Consequently, there is a need to examine the potential effect of these transients upon the ionosphere and the thermosphere.

Schunk et al. (1994) employed the Utah State University ionospheric model to study the response of the ionosphere to representative traveling convection twin vortices for a range of background conditions. They found local, transient, ion and electron temperature enhancements, O^+^ density depletions and NO^+^ density increases, non-Maxwellian ion distributions including enhanced temperature anisotropies, elevated plasma scale heights, and plasma upwelling events. The effects were evident at all altitudes, but largest in the E and lower F regions, and somewhat larger in summer than winter.

Recent studies (Connor et al. 2014, 2016; Shi et al. 2017b; Ozturk et al. 2018) investigated the impact of the sudden magnetopause compression on the ionosphere-thermosphere system using a global magnetosphere-ionosphere-thermosphere coupled model. They found that the sudden magnetopause compression leads to enhancement of dayside aurora precipitation, modification of ionospheric convection pattern, temperature increase of the ionosphere and thermosphere, and neutral density increase at 400 km altitude.

### Outstanding Questions

Dayside transients are frequently observed throughout geospace, but there are still many open questions. Upstream transients are localized and short-lived, just like those in the ionosphere. But it is not always clear which event in the ionosphere is linked to which specific transient in the foreshock, bow shock, magnetosheath, or at the magnetopause. Observations are scarce and typically constrained by point measurements (in-situ spacecraft) or limited field-of-view (riometers, magnetometers, radars, and ground-based imagers). Consequently, a complete view of the whole system is missing, because simultaneous global observations are unavailable.

The topology of Earth’s magnetic field, and especially how open magnetic field lines map through a dynamic and disturbed magnetopause, is not understood beyond a very basic conceptual level. Consequently, it is not straightforward to trace events on open magnetic field lines between the magnetosheath and the polar ionosphere. For the same reason, more work is needed in order to make robust predictions that allow transients to be followed and tracked. The Earth’s magnetosphere is also a barrier that may block smaller transients from giving any detectable signatures in the ionosphere/thermosphere.

In the last 10-15 years a new understanding has emerged that geospace is asymmetric (Laundal and Østgaard 2009), which plays a key role in how the northern and southern hemispheres respond differently to solar-wind driving. Differences in sunlight exposure, magnetic field strength, and pole offsets lead to observed asymmetries in ionospheric convection, thermospheric winds, currents and magnetic field perturbations, ion outflow, electron density, and auroral emissions (Laundal et al. 2017). The relative importance of each process is still debated, including the role of asymmetric feedback. However, asymmetries should play a significant role in redistribution of ionospheric plasma, formation of ionospheric irregularities, and energetic particle precipitation.

In the polar ionosphere there are many open questions related to plasma circulation, as more instrumentation leads to better data coverage in remote locations of the Earth. A topic currently under active investigation is ionospheric flow channels (Reistad et al. 2019a,b; Herlingshaw et al. 2019, 2020). What is the role of the Earth’s dipole tilt in plasma circulation? What are the size and time constraints for meso-scale ionospheric flow channels to develop? Under what IMF conditions do different types of flow channels occur? Do all flow channels transit the polar cap (Nishimura et al. 2014), or does it only apply to a subset (e.g. only the largest transients)? Consequently, it is still very difficult to predict the dynamics and implications of transients in the polar ionosphere and thermosphere.

An important method to identify boundary processes and their relative location in the magnetosphere is the mapping of ionospheric signatures into the magnetosphere and its boundary layer. However, although there is much auroral activity for higher latitudes in the giant magnetospheres, there is an open debate on where the actual open-closed boundary is located in the ionosphere. Therefore it is not well established whether auroral events map to the actual boundary or much deeper into the magnetosphere.

In summary, quite little is known about the overall geo-effective impact of dayside transients, both at Earth and at other planets. Open questions remain on properties of ULF waves generated by dayside transients; which fraction is generated by KH waves, pressure pulses, and foreshock transients? Nevertheless, it is evident that dayside transients must play a key role in space weather disruptions both at Earth and at other planets. A better understanding of dayside transients is therefore fundamental for society, as everyone in their daily life becomes more dependent on advanced technology, and human activity is expected to grow, both in space and in polar regions.

## Conclusions and Outstanding Questions

A veritable zoo of transient phenomena has been identified upstream from the bow shock. These phenomena include HFAs, SHFAs, foreshock bubbles, density holes, foreshock cavities, foreshock cavitons, foreshock compressional boundaries, and SLAMs. They show both similar and different characteristics (Table 1). Among these phenomena, HFAs and SHFAs have been studied extensively. HFAs and SHFAs exhibit core regions with low field strength, low density, plasma heating, and plasma deflection bounded by one or two compressional boundaries. Their typical spatial scales are several $R_{\mathrm{{E}}}$. Occurrence patterns for the two phenomena do not show clear statistical differences except that HFAs require a TD to form. HFAs are universal phenomena which have been observed at many planets. On average, their occurrence rate is about several per day. Conditions favoring HFA formation are fast solar wind, high Mach number, low IMF strength, small IMF cone angles, large magnetic shear angles across TDs, and small TD transit speed along the bow shock surface. HFAs are very dynamic. As HFAs evolve from “young” to “mature”, foreshock ions and solar wind ions gradually merge into one diffuse population. As the plasma and magnetic field perturbations associated with HFAs strengthen, wave activity within them intensifies. HFAs are also efficient particle accelerators with multiple acceleration mechanisms proposed. Magnetic reconnection and flux ropes have also been observed inside HFAs. Foreshock bubbles, density holes, foreshock cavities, and foreshock cavitons are all characterized by core regions with low field strength and low density, but they exhibit different degrees of plasma heating and deflection and have very different formation mechanisms. Foreshock compressional boundaries can be identified as regions of enhanced field strength and density that separate the solar wind from the foreshock. Inside the foreshock, there are also ULF waves, which can further steepen into shocklets and SLAMS with enhanced fluctuations. Solar wind discontinuities or local, transient bow shock deformations, enable the occurrence of localized, transient foreshock regions.

Multiple types of transient structures have been observed in the magnetosheath. Magnetosheath jets are localized structures with enhanced dynamic pressure and typical spatial scales of less than one $R_{\mathrm{{E}}}$. They mainly occur downstream from the quasi-parallel bow shock. They form due to bow shock ripples, solar wind discontinuities, or foreshock transients. They are associated with enhanced dynamic pressures that can compress the magnetopause, which can in turn disturb the magnetosphere and ionosphere, trigger magnetic reconnection, and result in plasma penetration across the magnetopause. Due to their fast motion, they can also drive a bow wave that can accelerate particles. In the magnetosheath, there are also magnetic holes and magnetic peaks with local field strength decreases and increases, respectively, from MHD scales to kinetic scales. They form due to mirror mode or solitary waves. Particle acceleration, deceleration, and various waves have been observed inside them.

These transient phenomena have been observed and simulated at various planets (Table 4). In principle, they very likely exist at all planets because their physical background is similar to those at Earth. Observations are needed to fill the empty slots in Table 4. Hence, Table 4 gives the future direction of research on the transient events. To study these events at various planets and compare their features with their terrestrial counterpart will dramatically improve our understanding of them.

Surface waves at the magnetopause are excited by solar wind pressure variations, transient phenomena generated near the bow shock, or the KHI on the magnetopause, and have been observed at Earth, Mercury, Venus, Mars, and Saturn. They may play a significant role in viscous or turbulent transport of solar wind plasmas into the magnetotail and in driving magnetospheric ULF waves. A recent extensive survey using THEMIS data has shown that the terrestrial KH waves are present approximately 19% of the time and the occurrence rate increases with the solar wind speed, Alfvén Mach number, and number density. The KH wavelength increases as they propagate toward the tail. KH waves have been observed not only on the flanks, but also in the high latitude cusp region during strongly dawnward IMF conditions and even close to the subsolar point when dense plume plasma is present in the magnetosphere. During southward IMF, magnetic reconnection and KHI can occur simultaneously and interact with each other. Recent MMS observations show unambiguous evidence of magnetic reconnection and properties of turbulence/waves associated with KHI.

FTEs, with a typical duration of 1 min and spacing of 10 min, are frequently observed at Earth during southward IMF. MMS observations revealed structures and evolution of both typical and ion-scale FTEs. FTEs are very common at Mercury’s magnetopause with a typical duration of ∼1 s and spacing of 4 s. They have been observed during both northward and southward IMF conditions. At giant planets, in contrast, observations of FTEs are rare. The solar wind interaction with giant planets is fundamentally different from Earth and Mercury, where IMF is important in coupling solar wind to the magnetosphere. A complete understanding of the viscous-like interaction must combine KH instability with magnetic reconnection.

Dynamic pressure variations associated with dayside transients deform the magnetopause and launch field-aligned currents that drive traveling convection vortices and plasma flow in the high-latitude ionosphere. Dayside transients can also transmit compressional waves into the magnetosphere that can excite resonant ULF waves locally or globally. Analytical theories, numerical simulations, and in-situ observations have found that foreshock compressional waves can directly mode convert to shear Alfvén waves when the compressional wave frequency matches the local Alfvén resonance condition due to the nonuniformity of the magnetosphere, providing an effective mechanism of ULF wave generation. These ULF waves can interact with particles in the radiation belts, ring current, and the plasmasphere. Transient compressions by dayside transients can create or enhance plasma anisotropies that lead to the growth of plasma waves which can scatter particles into the loss cone and precipitate into the ionosphere, resulting in transient auroral brightenings. Enhanced ionospheric densities due to particle precipitation can heat and expand the high-latitude thermosphere and increase cosmic noise absorption. Dayside transients can impact not only the dayside but also the nightside magnetosphere and ionosphere since dayside transients and the compressional waves excited by them can propagate to the nightside. For example, FTEs and KH waves can transport mass and magnetic flux from dayside to the magnetotail plasma sheet. The magnetotail plasma sheet expands and contracts in response to the magnetopause perturbations driven by foreshock transients.

The forthcoming joint European Space Agency and Chinese Academy of Sciences spacecraft mission Solar wind Magnetosphere Ionosphere Link Explorer (SMILE), which is presently scheduled for launch in November 2023 will offer new opportunities to observe the effects of foreshock transients upon the Earth’s magnetosphere (Branduardi-Raymont and Wang 2017). Operating from a highly inclined ($70-98^{\circ }$) and highly elliptical orbit with an apogee of 20 $R_{\mathrm{{E}}}$, the spacecraft will carry a magnetometer and top-hap ion analyzer capable of observing the solar wind input from a near-Earth vantage point, a wide field-of-view soft X-ray telescope capable of identifying the location of the subsolar magnetosheath, and a far ultraviolet camera capable of observing the auroral oval. SMILE’s soft X-ray telescope is a novel instrument designed to measure the emissions generated when high charge state solar wind ions exchange charges with exospheric neutrals in the dayside magnetosheath and cusps. Since high charge solar wind ions do not penetrate the dayside magnetopause, there are no emissions from the outer dayside magnetosphere. Numerical simulations have shown that the telescope is capable of distinguishing the low emission dayside magnetosphere from the high emission magnetosheath and therefore identifying the magnetopause in line-of-sight integrated images. Whether or not the cadence of the images suffices to image foreshock transient events or their effects on the magnetopause remains an open question. The typical solar wind density is 5 cm^−3^ and the typical solar wind velocity is 400 km s^−1^. As noted earlier in this article, foreshock events tend to occur for low solar wind densities and high solar wind velocities, perhaps densities of 2.5 cm^−3^ and velocities of 800 km s^−1^. In either case, the solar wind ion flux is $2.0 \times 10^{8}\mbox{ cm}^{-2}$ s^−1^. For fluxes like these, the integration time required for the instrument to construct an image and identify the subsolar magnetopause is on the order of 25 min, longer than the 5 to 15 min required for solar wind features to sweep past the entire dayside magnetosphere. The situation with the far ultraviolet imager on SMILE is far more favorable. The imager will distinguish the dayside auroral emissions driven by transient events at the bow shock and magnetopause from Earth’s albedo and nominal dayglow by imaging with a resolution of 150 km and a cadence of 60 s in the 160-180 nm Lyman-Birge-Hopfield Long band. This will be able to detect the occurrence, extent, and propagation of the auroral brightenings associated with transient compressions of the magnetosphere driven by foreshock events.

Although significant progress has been made on dayside transients and their geoeffects, there are still many open questions. We list below some outstanding questions (in addition to the specific outstanding questions at the end of each section):

- What are the physical differences and relationships among transient phenomena upstream from the bow shock (such as hot flow anomaly, foreshock bubbles, foreshock cavities, and density holes)?
- How much energy is transported to the foreshock region and how fast is the energy coupled to the solar wind?
- How much do foreshock transients contribute to particle acceleration at shocks?
- How do they evolve with time and transition through the magnetosheath?
- Do foreshock events trigger transient features (magnetic reconnection/FTEs, surface waves etc.) at the magnetopause?
- How does the ionosphere respond to transient phenomena generated at the magnetopause and bow shock?
- How can we quantify the energy and momentum transport during the dayside transient phenomena? How deterministic is the magnetosphere?
- How much do dayside transients contribute to magnetospheric dawn-dusk asymmetry?
- Are dayside transients equally important for different magnetospheres (e.g., Saturn and Jupiter’s magnetospheres)?
